# Small mammals and associated infections in China: a systematic review and spatial modelling analysis

**DOI:** 10.1016/j.lanwpc.2024.101264

**Published:** 2024-12-18

**Authors:** Jin-Jin Chen, Chen-Long Lv, Tao Wang, Yan-He Wang, Tian-Le Che, Qiang Xu, Xue-Geng Hong, Ai-Ying Teng, Shen Tian, Yuan-Yuan Zhang, Mei-Chen Liu, Li-Ping Wang, Simon I. Hay, Yang Yang, Li-Qun Fang, Wei Liu

**Affiliations:** a State Key Laboratory of Pathogen and Biosecurity, Academy of Military Medical Science, Beijing, PR China; b Department of Clinical Laboratory, The Second Affiliated Hospital of Anhui Medical University, Hefei, PR China; c The 949th Hospital of Chinese PLA, Altay, Xinjiang, 836300, PR China; d The 968th Hospital of Chinese PLA, Jinzhou, Liaoning, 121000, PR China; e Guangzhou Center for Disease Control and Prevention, Guangzhou, PR China; f Division of Infectious Disease, Key Laboratory of Surveillance and Early-warning on Infectious Disease, Chinese Center for Disease Control and Prevention, Beijing, PR China; g Department of Health Metrics Sciences, School of Medicine, University of Washington, USA; h Institute for Health Metrics and Evaluation, University of Washington, USA; i Department of Statistics, Franklin College of Arts and Sciences, University of Georgia, Athens, GA, USA

**Keywords:** Small mammals, Rodent, Spatial modelling analysis

## Abstract

**Background:**

As natural reservoirs of diverse pathogens, small mammals are considered a key interface for guarding public health due to their wide geographic distribution, high density and frequent interaction with humans.

**Methods:**

All formally recorded natural occurrences of small mammals (Order: Rodentia, Eulipotyphla, Lagomorpha, and Scandentia) and their associated microbial infections in China were searched in the English and Chinese literature spanning from 1950 to 2021 and geolocated. Machine learning models were applied to determine ecological drivers for the distributions of 45 major small mammal species and two common rodent-borne diseases (RBDs), and model-predicted potential risk locations were mapped.

**Findings:**

A total of 364 small mammal species collectively carrying 155 small mammal-associated microbes (SMAMs) combined with 215,791 human cases of eight RBDs were reported in 2484 counties in China. Murid rodents (Family: Muridae) including the brown rat (*Rattus norvegicus*), the house mouse (*Mus musculus*), and the striped field mouse (*Apodemus agrarius*) are the most widespread species, while *Rattus norvegicus* harbored the highest variety of SMAMs (75 species), followed by the tanezumi rat (*Rattus tanezumi*) (68 species). The top three SMAMs that infest the highest variety of small mammal species are *Yersinia pestis* (58 small mammal species), *Bartonella grahamii* (36 species), and *Orientia tsutsugamushi* (33 species). The 45 major species of small mammals were grouped into six ecological clusters based on their ecological niche, mainly driven by annual mean temperature, temperature seasonality, total precipitation, and elevation. Model-predicted presence areas for the 45 major small mammal species and two RBDs were 1–499% larger in geographic size than observed.

**Interpretation:**

The extensive intersection between small mammals and microbes with pathogenic potential in humans poses imminent threats to public health. Active field surveillance should be prioritized for potential high-risk areas identified in this study to prevent zoonotic transmission of SMAMs.

**Funding:**

10.13039/501100012166National Key Research and Development Program of China; Natural Science Foundation of China; The U.S. 10.13039/100000030Centers for Disease Control and Prevention.

**Research in context**

**Evidence before this study**

We searched PubMed, Web of Science, MedRvix, and BioRvix for peer-reviewed English and Chinese research publications and reports. The search keywords were as follows: (“*rodent*” [All Fields] OR “*shrew*” [All Fields] OR “*mole*” [All Fields] OR “*hedgehog*” [All Fields] OR “*rabbit*” [All Fields] OR “*pika*” [All Fields] OR “*tree shrew*” [All Fields] OR “*mammal*” [All Fields]) AND (“*spatial*” [All Fields] OR “*distribution*” [All Fields] OR “*geographical*” [All Fields] OR “*map*” [All Fields]) AND (“*China*” [All Fields]) without any date or language restrictions. A total of 490 relevant papers were retrieved, of which 340 papers focused on animal models, drugs, vaccines, and molecular mechanisms, 73 papers reported field studies of small mammal-associated microbes, 62 papers conducted spatial analyses of rodent-borne diseases (RBDs), and 15 papers described small mammal-associated arthropod vectors (i.e., ticks, mites, and fleas). Among the 62 papers involving spatial analyses, the geospatial distribution of one specific rodent-borne pathogen (RBP) or RBD was investigated at either the local city level (58 papers) or the national level (4 papers). No study has explored the geographic distributions and ecological niches of multiple small mammal species or RBPs at the national scale.

**Added value of this study**

The current study represents the first systematic review and geospatial modelling analysis of small mammal species and RBPs in China. We evaluated the richness, geographic distributions, and ecological drivers of small mammals and RBPs, as well as the biological linkage between small mammals and RBPs. We determined six clusters of small mammal species with similar ecological niches and geographic distributions. We used 52 socio-environmental factors to establish ecological models that yielded ≥90% accuracy in predicting the distributions of 45 dominant small mammal species. The effects of these ecological factors on the distribution and cluster patterns of the small mammals were determined and compared. The incidence and distribution of hemorrhagic fever with renal syndrome and leptospirosis, two RBDs associated with a high disease burden, were examined and predicted by the model. Compared to previous reports, the model predicted 1–499% wider geographic ranges for the small mammal species and an 8–66% higher incidence for the RBDs.

**Implications of all the available evidence**

The intersection between small mammals and microbes with pathogenic potential in humans is extensive, and the geographic regions that are ecologically suitable for small mammals and associated RBDs are broad. The risk maps produced by this study can inform targeted field surveillance of RBPs and allocation of public health resources for preventing and controlling RBDs.

## Introduction

With strong ecological adaptability and high reproductive potential, small mammals (Order: Rodentia, Eulipotyphla, Lagomorpha, and Scandentia), especially rodents (Order: Rodentia), constitute the most abundant and diverse groups of living mammals. Rodents represent nearly 42% of mammalian species and cover a wide variety of biotopes globally.^1^ The richness, mobility, opportunism, and synanthropy of rodent species make them efficient amplifiers and spreaders of zoonotic diseases.^2^ Rodents serve as an important reservoir of many zoonotic diseases that can cross species barriers to infect humans and other animal hosts such as birds, pangolins, and swine.^3^, ^4^, ^5^ This is exemplified by the 31 mammalian virus families reported in the DRodVir online database, as of 21 July 2021.^6^ The increasing prevalence of certain generalist small mammal (SM) species, which thrive in a variety of environments and consume a wide range of food resources, raises concerns about their potential role in the spillover and outbreaks of emerging and re-emerging pathogens such as Langya henipavirus and monkeypox virus.^7^^,^^8^ These generalist and invasive species pose a heightened risk for disease transmission across various ecosystems and hosts.

Rodent-borne pathogens (RBPs) are transmitted through multiple routes: direct biting; food, water, or even air carrying viral particles from rodent feces, urine, or decomposed bodies; or via arthropod ectoparasites and vectors such as ticks, mites, and fleas.^9^^,^^10^ For example, *Yersinia pestis* can be transmitted to humans through rodent fleas, causing plague. Rodents can serve as amplifying hosts of RBPs and help sustain pathogen transmission cycles in diverse environments including human and wild habitats. The geographic range expansion of rodent species may be jointly driven by climatic factors, environmental changes, and human-related activities.^2^^,^^11^, ^12^, ^13^ For example, anthropogenic changes in land-use, trade across regions, and agricultural activities have been associated with rodent invasion and spillover of RBPs. Flooding can force rodents to migrate from their burrows near water sources to build environments closer to humans. The brown rat (*Rattus norvegicus*), a host for *orthohantavirus*, *Leptospira*, and *Yersinia pestis*,^14^ was first discovered in northern China and Mongolia, became established in Europe by the 1500s, was introduced to North America by the 1750s, and now occupies almost all major landmasses outside polar regions.^1^^,^^10^ In contrast, the house mouse (*Mus musculus*) is believed to have spread through nearly all of Eurasia during prehistoric human migrations.^15^ The global expansion of some rodent species is considered a potential threat to public health and animal health, given their capability of carrying and transmitting hundreds of known zoonoses.^2^^,^^11^^,^^12^

In the last three decades, high throughput sequencing and microbiome studies of SMs have revealed a variety of novel small mammal-associated microbes (SMAMs) in China.^16^ Other SMs from different orders, such as lagomorphs, shrews, and tree shrews, are less studied. A comprehensive understanding of the microbiological community associated with SMs including knowledge of microbial prevalence, genetic diversity, and geographic distribution is valuable for risk assessment and early warning of transmission across species barriers. However, it is costly to monitor all SMs and infections with SMAMs on a regular basis at the national level. Mathematical modelling and novel risk-assessment approaches have been used to predict undetected infections in SMs and potential pandemics.^2^^,^^12^ Here we assembled a comprehensive and up-to-date database on the distributions of SMs and associated SMAMs in China between 1950 and 2021 and conducted ecological modelling for major SMs and two rodent-borne diseases (RBDs) with high disease burdens, hemorrhagic fever with renal syndrome (HFRS) and leptospirosis. We hypothesized that the ecological niches of these SMs and related infectious diseases are highly predictable using socio-environmental variables.

## Methods

### Data sources

In the field of zoology, SMs are typically defined as a group of animals that are relatively small compared to larger mammals such as elephants, cows, or humans. SMs generally include a wide range of species from different taxonomic groups. We extracted from multiple sources of records describing the presence of four major orders of SMs including Rodentia (235 rodent species), Eulipotyphla (92 shrew, mole, and hedgehog species), Lagomorpha (36 rabbit, hare, and pika species), and Scandentia (one tree shrew) and the detection of SMAMs in human cases of RBDs, rodents, and other mammalian orders (but excluding arthropod vectors) in China. These data sources include: (1) literature in English and Chinese; (2) Figshare,^17^ GBIF, IUCN, and GenBank databases; (3) the China Information System for Disease Control and Prevention (CISDCP); and (4) surveillance reports on key infectious diseases and vectors in China. For the literature review, we searched four major online databases of publications between January, 1950 and December, 2021, including Web of Science (https://access.clarivate.com/), PubMed (https://pubmed.ncbi.nlm.nih.gov/), China National Knowledge Infrastructure (CNKI, https://www.cnki.net/), and WanFang database (https://www.wanfangdata.com.cn/), the latter two mainly dedicated to Chinese literature. Articles reporting SMAMs were retrieved using the following keywords to search titles, abstracts, and keywords: (“*mammal*” and “*pathogens*” or “*virus*” or “*bacterial*” or “*microbiology*” or “*diseases*” or “*infections*”) and “*China*”. The inclusion criterion for this screening stage consisted of field studies that reported the presence of SM species or SMAMs, with clear information on the study time, location, and methods. Studies were excluded if they: (i) were unrelated to the detection of SM species or SMAMs; (ii) did not provide geographic information for the detection; or (iii) focused on the development of drugs or vaccines, systematic reviews, meta-analyses, opinions and perspectives, or conference posters and presentations. The titles and abstracts of all studies were independently screened for inclusion and cross-checked by two authors (JJC, TW). For all eligible studies, the full text was retrieved and assessed for eligibility by the same authors. Inconsistent opinions on study selection, data extraction, and quality assessment processes were resolved through discussions with WL and LQF.

**Fig. 1:**
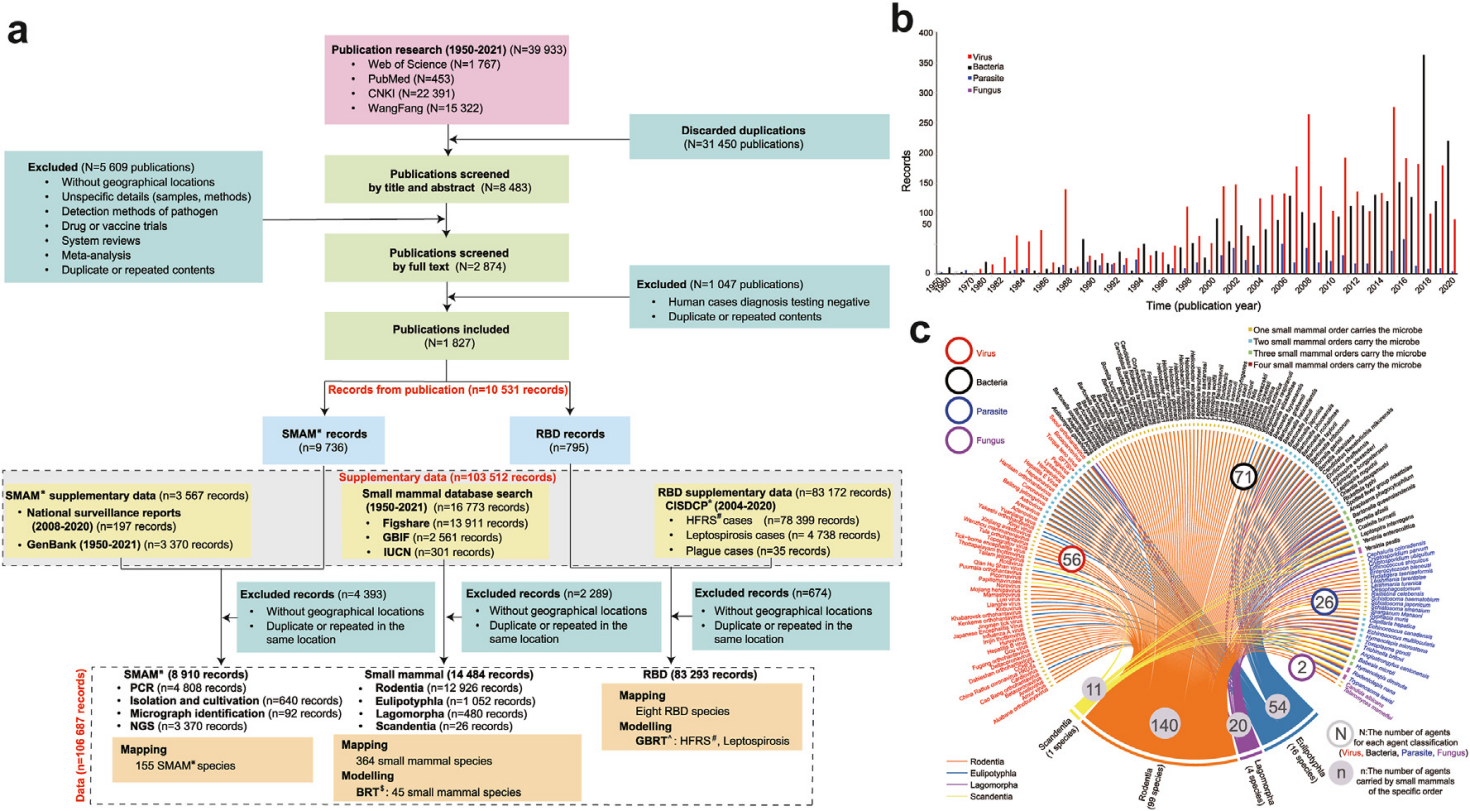
**Flow chart of literature review, number of records over time, and chord diagram.** (a)This flow chart summarizes the collection and sorting of records of small mammal families and associated microbes for each analysis in this study. ∗China Information System for Disease Control and Prevention (CISDCP), a national surveillance system serving the general population of the entire country; ^#^Hemorrhagic fever with renal syndrome (HFRS); ^&^Boosted regression tree (BRT); ^ˆ^Generalized boosted regression tree (GBRT); ^※^Small mammal-associated microbes (SMAMs); (b)Annual number of records on small mammals from 1950 to 2020, stratified by SMAM type; (c)Chord diagram of recorded host–microbe relationships between small mammal orders and SMAM species including viruses (red), bacteria (black), parasites (blue) and fungi (purple).

From eligible peer-reviewed publications, reports and online databases, the species or family of identified SMs and detected SMAMs, as well as the total number of tests and prevalence wherever available, were extracted (see Appendix 1 pp 2 and 12 for details). Additional extracted data included the first author, year of publication, geographic location, and study period, description of study groups, and diagnostic tests or laboratory methods used. The annual numbers of reported cases of HFRS, leptospirosis and plague at the county level in mainland China between 2004 and 2020 were extracted from CISDCP. We geolocated patients based on their residential addresses at the time of disease onset. For travel-related patients recorded in the CISDCP or literature, we assigned their geolocation to the places of potential exposure, and cases with missing potential exposure locations were excluded. For all SMAMs detected in SMs, the sampling sites were geolocated. Serological detections were not considered conclusive due to low test specificity and potential cross-reactivity between SMAMs and were classified as “suspected reservoirs” unless the serologic detection was followed by a confirmatory test. The confirmatory tests include polymerase chain reaction (PCR) and next generation sequencing (NGS) for obtaining genetic evidence, isolation and cultivation of the microbes, and micrograph identification of the microbes. We use species names of SMAM whenever available; otherwise the genus or family name is used, followed a note in square brackets, e.g., Anellovirus [genus], when it first appears. All the extracted data were integrated to form a single database at the county level for the final analyses (Fig. 1a).

Mapping and spatial analysis were performed for the SMs and SMAMs at both the family and species levels, while ecological modeling analysis was performed at the species level for the major SMs as well as for two SMAMs associated with significant human disease burden, *orthohantavirus* and *Leptospira*, which are the causative agents of HFRS and leptospirosis respectively. A record of a SMAM was defined as one or more detections of the SMAM in either SMs or human cases of the associated RBD (laboratory-confirmed or clinically diagnosed) in a unique administrative area. A record of a SM species was defined as one or more documented observations of the given species in a unique administrative area. If a SM species or SMAM was reported more than once in the same administrative area (e.g., through periodic survey or by different investigators), only one of the records was counted. If more than one microbe were found in the same study or isolated from the same SM, a record was created for each microbe. The laboratory tests used to detect SMAMs are described in Appendix 1 (p12).

We collected 24 environmental, nine socioeconomic, and 19 bioclimatic variables that are potentially associated with the ecology of SMs or RBD-associated SMAMs. Environmental data at a resolution of one square kilometer in China during 1985–2015 were obtained from the National Earth System Science Data Sharing Infrastructure (http://www.geodata.cn). Monthly climatic data from 1981 to 2018, including average temperature, average maximum temperature, average minimum temperature, average relative humidity, and accumulated rainfall, were collected from 2006 weather surveillance stations in the mainland of China, covering 74.0% of 1228 surveyed counties (http://cdc.nmic.cn/home.do). For the 877 counties without meteorological stations, the mean values of meteorological variables at the nearest five surveillance stations were used as a proxy. From these routine meteorological variables, 19 cross-sectional bioclimatic variables (BIO01−19, also called bioclimatic variables recommended by the U.S. Geological Survey) were calculated and their yearly averages were used as predictors in our ecological models. Demographic and socioeconomic data at the county level were derived from the Sixth National Census of China in 2010 (the National Bureau of Statistics), including the numbers of specialized hospitals, general hospitals, health centers, clinics, and emergency centers, human population density, gross domestic product, the percentage of females, and the percentage of residents ≥60 years old. Data extraction, cleaning and reorganization were performed using the ArcGIS Desktop 10.7 software (ESRI Inc., Redlands, CA, USA) and the statistical software R v4.0.3 (Appendix 1 pp 3, 13).

### Ecological modeling and clustering of major small mammal species

Boosted Regression Tree (BRT) models were built at the county level to assess driving factors for the ecological suitability of each of the 45 major SM species, where the outcome of each model is the presence or absence of a given species in each county based on all survey records. A species was considered major if its occurrence was recorded in ≥50 counties. To achieve robust predictions, BRT modeling was conducted on 100 resampled data sets obtained by a random split of the data into 75% training and 25% test sets. Results are based on average and percentile values across the 100 BRT models (Appendix 1 pp 4–5). Using the fitted models, we predicted the potential ecological niches for each of them.^18^, ^19^, ^20^ The area under the curve (AUC) of the receiver operating characteristic was used to measure the discriminatory performance of the ecological models in terms of a balance between sensitivity and specificity. To explore the similarity of ecological niches among the major SM species, a hierarchical cluster analysis based on the weighted-average linkage method was performed using three indexes,^18^^,^^19^ including: (1) the average relative contribution (RC) of each significant predictor across the 100 resampled data sets, where RC is an indicator of the importance of a predictive factor to the ecological suitability; (2) a measure for the difference in each significant predictor between presence counties and all counties; and (3) the linear correlation between each significant predictor and model-predicted presence probabilities of the given SM species among all counties. A predictor is considered a significant contributor to ecological suitability if its average RC across the 100 BRT models was above 5 (Appendix 1 p 6). These three indexes reflect ecological similarity among the major SM species (Appendix 1 p 6). A dendrogram was created to demonstrate the clustering pattern of the major SM species, together with a thematic matrix illustrating the features.

### Ecological modeling of two RBD-associated SMAMs

A generalized boosted regression tree (GBRT) approach was used to assess the potential drivers for the ecology of the *orthohantavirus* and *Leptospira*, which cause HFRS and leptospirosis respectively.^21^ In addition to the 43 variables used in the ecological modelling of SMs, nine socioeconomic factors and the predicted presence probabilities associated with seven SM hosts were also included as potential predictors for the two pathogens (Appendix 1 pp 14−16). For each pathogen, a two-step GBRT modelling approach was implemented using the R package “*xgboost*” (Appendix 1 p 7).^19^ In the first step, a logistic GBRT model was fitted to the presence/absence of the pathogen on each of 100 resampled data sets.^21^ In this step, all counties with either reported human cases during 2004–2020 or detected infections in SMs by PCR or isolation during 1950–2021 were classified as “presence” locations while those without were classified as “absence” locations. For *orthohantavirus*, a sensitivity analysis was performed to include serological detection in SMs as evidence for presence. In the second step, a different GBRT model was developed to fit the average annual incidence rate of reported human cases during 2004–2020, assuming a gamma distribution for the outcome. This analysis was limited to counties with an average predicted occurrence probability above 0.5, as computed by the first-step model.^22^ Similarly, the gamma model was fitted to 100 resampled data sets.

### Role of the funding source

The funders of the study had no role in the design and conduct of the study; collection, management, analysis, and interpretation of data; preparation, review, or approval of the manuscript; and decision to submit the manuscript for publication.

## Results

A total of 10,531 records were retrieved from 39,333 peer-reviewed articles in Chinese and English from the literature search and 103,512 records were retrieved from Figshare, GBIF, IUCN, GenBank, CISDCP and surveillance reports. After screening using the inclusion and exclusion criteria and removing duplicated data, 106,687 records were included in this study, with 14,484 records for SMs (comprising 364 SM species belonging to four orders and 18 families), 8910 records for SMAMs (6040 on viruses, 2276 on bacteria, 588 on parasites, and six on fungi), and 83,293 records for RBDs (Fig. 1a). The full list of SMAMs and corresponding publications are provided in Appendix 1 (pp 17−20, 32−105). The annual number of records of SMAMs (by publication year) increased since the end of the 1990s, especially for those describing viral and bacterial zoonotic agents (Fig. 1b). Four major orders of SMs collectively carry a total of 155 SMAMs which include 56 viruses, 71 bacteria, 26 parasites, and two fungi at the species level. The highest number of recorded SM species was found for the order Rodentia (140 species), followed by Eulipotyphla (54 species), Lagomorpha (20 species), and Scandentia (11 species) (Fig. 1c).

### Distribution and risk mapping for small mammal species

All 364 SM species are mapped at the county level in Appendix 2 (pp 2−4), and the corresponding data sets and their descriptions are given in Supplementary data 1 and 2 and Appendix 1 (p 8). The Muridae (present in 1503 counties), Cricetidae (823 counties), Sciuridae (801 counties), Spalacidae (408 counties), and Pteromyidae (307 counties) were the most widely distributed SM families, and *Rattus* (1304 counties), *Mus* (1178 counties), *Apodemus* (900 counties), and *Cricetulus* (510 counties) were the most widely distributed genera. At the species level, *Mus musculus*, *Rattus norvegicus*, *Apodemus agrarius*, and the tanezumi rat (*Rattus tanezumi*) were recorded in >500 counties, and the white-bellied rat (*Niviventer niviventer*), the chinese striped hamster (*Cricetulus barabensis*), and the Himalayan field rat (*Rattus nitidus*) were detected in 300–500 counties.

The diversity and richness of SM species varied markedly across the seven biogeographic zones (Northeast, North China, Inner Mongolia-Xinjiang, Qinghai-Tibet, Southwest, Central China and South China) which differed climatically and ecologically, with a notably higher abundance near borders of neighboring biogeographic zones (Appendix 2 p 5). Shannon index scores ranked the zones from the highest to the lowest species diversity as follows: Southwest (4.88), Inner Mongolia-Xinjiang (4.76), North China (4.19), South China (4.09), Central China (3.95), Qinghai-Tibet (3.83), and Northeast (3.69). The regions with relatively high richness, as indicated by Chao1 index scores, were Southwest China (191.52, hosting 166 species), Inner Mongolia-Xinjiang (127.69, hosting 126 species), and North China (63.58, hosting 63 species). The families Muridae, Sciuridae, and Cricetidae included the highest numbers of recorded species, with the former two more frequently recorded in South China and Central China and Cricetidae in Central China and Inner Mongolia-Xinjiang (Appendix 2 p 5).

We identified 45 major species of SMs that were recorded in ≥50 counties. The ecological models for these 45 major species showed adequate predictive power with testing AUC ranging from 0.78 to 0.97 (Table 1). The model-predicted geographic distributions of these SM species are substantially broader than the recorded species distributions. Compared to the recorded distributions, the predicted distributions were 42–438% greater in the number of counties, 1−499% wider in area, and 35−436% larger in residential human population size (Appendix 2 pp 6−9). *Rattus norvegicus* was predicted to be the most widespread SM species, affecting 839 million people residing in 1686 counties, followed by *Mus musculus* (815 million people in 1656 counties) and *Apodemus agrarius* (629 million people in 1268 counties). More than 300 million people reside in regions suitable for *Rattus tanezumi*, *Cricetulus barabensis,* and the white-bellied rat (*Niviventer niviventer*). In addition, the grey dwarf hamster (*Cricetulus migratorius*), the Siberian jerboa (*Orientallactaga sibirica*), the midday jird (*Meriones meridianus*), the Himalayan marmot (*Marmota himalayana*), and *Mus musculus* are the top five species inhabiting the largest areas, ranging from 3.1 to 4.3 million km^2^ (Table 1).

**Table 1:** The average testing areas under curve (AUC) for testing sets of the boosted regression tree (BRT) models and predicted vs. observed numbers, coverage areas, and human population sizes of affected counties for the 45 prevalent small mammal species in China.

Small mammal species	Average AUC (2.5–97.5% percentiles)	Predicted/observed (relative difference %)		
		Number of counties	Coverage area (10,000 km^2^)	Huamn population size (million)
*Mus musculus*^a^^,^^b^^,^^c^	0.920 (0.893, 0.947)	1656/1169 (41.7)	307.3/303.3 (1.3)	815.0/521.6 (56.2)
*Rattus norvegicus*^a^^,^^c^	0.925 (0.899, 0.947)	1686/1161 (45.2)	290.9/279.9 (3.9)	839.1/531.2 (58.0)
*Apodemus agrarius*^a^^,^^c^	0.930 (0.901, 0.952)	1268/771 (64.5)	204.7/148.4 (37.9)	629.9/366.9 (71.7)
*Rattus tanezumi*^a^^,^^c^	0.947 (0.923, 0.964)	837/534 (56.7)	173.1/125.7 (37.7)	435.5/257.6 (69.1)
*Niviventer niviventer*	0.897 (0.864, 0.930)	701/432 (62.3)	165.4/106.4 (55.5)	316.8/204.5 (54.9)
*Cricetulus barabensis*^a^^,^^c^	0.964 (0.936, 0.982)	739/393 (88.0)	106.9/66.3 (61.2)	393.2/183.7 (114.0)
*Rattus nitidus*	0.935 (0.904, 0.961)	513/360 (42.5)	140.6/98.4 (42.9)	217.5/161.4 (34.8)
*Sciurotamias davidianus*	0.933 (0.898, 0.966)	431/250 (72.4)	90.8/60.6 (49.8)	166.1/97.7 (70.0)
*Tamias sibiricus*	0.937 (0.909, 0.962)	473/248 (90.7)	178.2/106.6 (67.2)	139.9/82.8 (69.0)
*Tscherskia triton*	0.900 (0.859, 0.934)	624/232 (169.0)	138.2/67.9 (103.5)	296.5/106.4 (178.7)
*Spermophilus dauricus*	0.936 (0.907, 0.963)	407/208 (95.7)	93.6/64.1 (46.0)	176.1/91.3 (92.9)
*Apodemus peninsulae*	0.886 (0.822, 0.935)	427/200 (113.5)	177.1/91.8 (92.9)	125.9/64.2 (96.1)
*Callosciurus erythraeus*	0.896 (0.857, 0.930)	494/192 (157.3)	119.1/53.5 (122.6)	216.4/86.5 (150.2)
*Eospalax fontanierii*	0.962 (0.930, 0.987)	330/192 (71.9)	126.7/84.7 (49.6)	105.0/66.8 (57.2)
*Niviventer fulvescens*	0.888 (0.856, 0.926)	486/185 (162.7)	134.1/58.1 (130.8)	181.3/74.4 (143.7)
*Leopoldamys edwardsi*	0.896 (0.863, 0.936)	500/185 (170.3)	127.1/56.6 (124.6)	207.4/70.5 (194.2)
*Tamiops swinhoei*	0.911 (0.875, 0.940)	404/184 (119.6)	117.4/58.1 (102.1)	128.5/61.5 (108.9)
*Trogopterus xanthipes*	0.926 (0.882, 0.955)	336/182 (84.6)	80.2/47.0 (70.6)	125.8/72.6 (73.3)
*Micromys minutus*	0.832 (0.759, 0.889)	388/146 (165.8)	151.5/57.9 (161.7)	126.0/54.6 (130.8)
*Dremomys pernyi*	0.903 (0.856, 0.939)	344/131 (162.6)	101.5/39.5 (157.0)	110.5/42.6 (159.4)
*Rhizomys sinensis*	0.907 (0.847, 0.960)	288/131 (119.8)	71.2/33.8 (110.7)	100.4/44.1 (127.7)
*Crocidura suaveolens*	0.942 (0.865, 0.991)	198/128 (54.7)	33.3/21.0 (58.6)	92.3/60.3 (52.1)
*Rattus rattus*	0.871 (0.814, 0.950)	504/120 (320.0)	136.1/37.4 (263.9)	256.2/66.5 (285.3)
*Cricetulus longicaudatus*	0.938 (0.892, 0.972)	285/115 (147.8)	172.8/88.6 (95.0)	74.5/29.9 (149.2)
*Apodemus chevrieri*	0.958 (0.929, 0.980)	246/110 (123.6)	80.9/39.0 (107.4)	77.9/33.6 (131.8)
*Meriones meridianus*^b^	0.965 (0.948, 0.983)	313/104 (201.0)	356.0/160.1 (122.4)	77.7/22.9 (239.3)
*Marmota himalayana*^b^	0.972 (0.927, 0.991)	208/100 (108.0)	355.6/163.4 (117.6)	20.3/11.0 (84.5)
*Alexandromys fortis*	0.879 (0.797, 0.948)	306/98 (212.2)	104.4/39.0 (167.7)	116.3/39.0 (198.2)
*Rattus losea*	0.935 (0.875, 0.980)	282/94 (200.0)	44.8/16.5 (171.5)	132.5/43.7 (203.2)
*Orientallactaga sibirica*^b^	0.942 (0.909, 0.967)	342/92 (271.7)	388.3/112.5 (245.2)	98.0/24.4 (301.6)
*Eothenomys melanogaster*	0.911 (0.851, 0.955)	159/90 (76.7)	42.1/25.3 (66.4)	53.1/32.4 (63.9)
*Apodemus draco*	0.899 (0.845, 0.942)	241/89 (170.8)	93.3/35.7 (161.3)	67.6/28.4 (138.0)
*Pteromys volans*	0.857 (0.767, 0.944)	350/80 (337.5)	164.0/39.2 (318.4)	86.5/23.6 (266.5)
*Craseomys rufocanus*	0.944 (0.889, 0.983)	238/79 (201.3)	91.8/36.5 (151.5)	80.1/38.8 (106.4)
*Petaurista alborufus*	0.921 (0.879, 0.954)	226/78 (189.7)	62.6/24.9 (151.4)	81.8/31.4 (160.5)
*Eothenomys miletus*	0.934 (0.864, 0.980)	232/75 (209.3)	80.3/25.9 (210.0)	69.0/24.0 (187.5)
*Myospalax psilurus*	0.885 (0.806, 0.947)	311/69 (350.7)	187.6/37.9 (395.0)	110.2/31.0 (255.5)
*Berylmys bowersi*	0.908 (0.845, 0.950)	297/67 (343.3)	72.8/19.6 (271.4)	107.8/26.1 (313.0)
*Bandicota indica*	0.962 (0.938, 0.979)	339/63 (438.1)	66.5/16.2 (310.5)	195.6/36.5 (435.9)
*Niviventer confucianus*	0.784 (0.609, 0.906)	321/62 (417.7)	108.8/19.3 (463.7)	95.1/20.8 (357.2)
*Ochotona thibetana*	0.944 (0.896, 0.979)	213/61 (249.2)	258.2/52.4 (392.7)	27.4/7.7 (255.8)
*Cricetulus migratorius*^b^	0.951 (0.907, 0.988)	289/60 (381.7)	427.5/71.4 (498.7)	59.3/11.7 (406.8)
*Anourosorex squamipes*	0.901 (0.818, 0.957)	252/55 (358.2)	82.5/16.8 (391.1)	76.1/17.2 (342.4)
*Meriones unguiculatus*	0.950 (0.892, 0.990)	190/55 (245.5)	178.4/59.9 (197.8)	51.3/13.5 (280.0)
*Ochotona dauurica*	0.968 (0.900, 0.993)	134/54 (148.1)	125.8/41.6 (202.4)	29.3/12.5 (134.4)

The predicted numbers are based on counties with predicted probability of presence >0.5. The relative differences (%) between predicted and observed values are given in parentheses.

a Top five small mammal species affecting the greatest numbers of counties.

b Top five small mammal species affecting the widest areas.

c Top five small mammal species affecting the largest population sizes.

Among all the considered variables, temperature seasonality (measured as a standard deviation of monthly temperature) and annual mean temperature are the two most important ecological drivers, with average RCs ≥5% for the distributions of 35 and 30 SM species, respectively, followed by total precipitation and elevation each contributing significantly to 25 species (Appendix 1 pp 22−30; Appendix 2 pp 10−12). However, the directions of the effects of temperature seasonality differ among SM species. For instance, higher temperature seasonality (higher variability in temperature throughout the year) was an important driver for the presence of the Himalayan field rat (*Rattus nitidus*) and *Rattus tanezumi*, while less temperature seasonality was favored by *Rattus rattus* and *Rattus norvegicus*.

### Ecological clustering of SM species

**Fig. 2:**
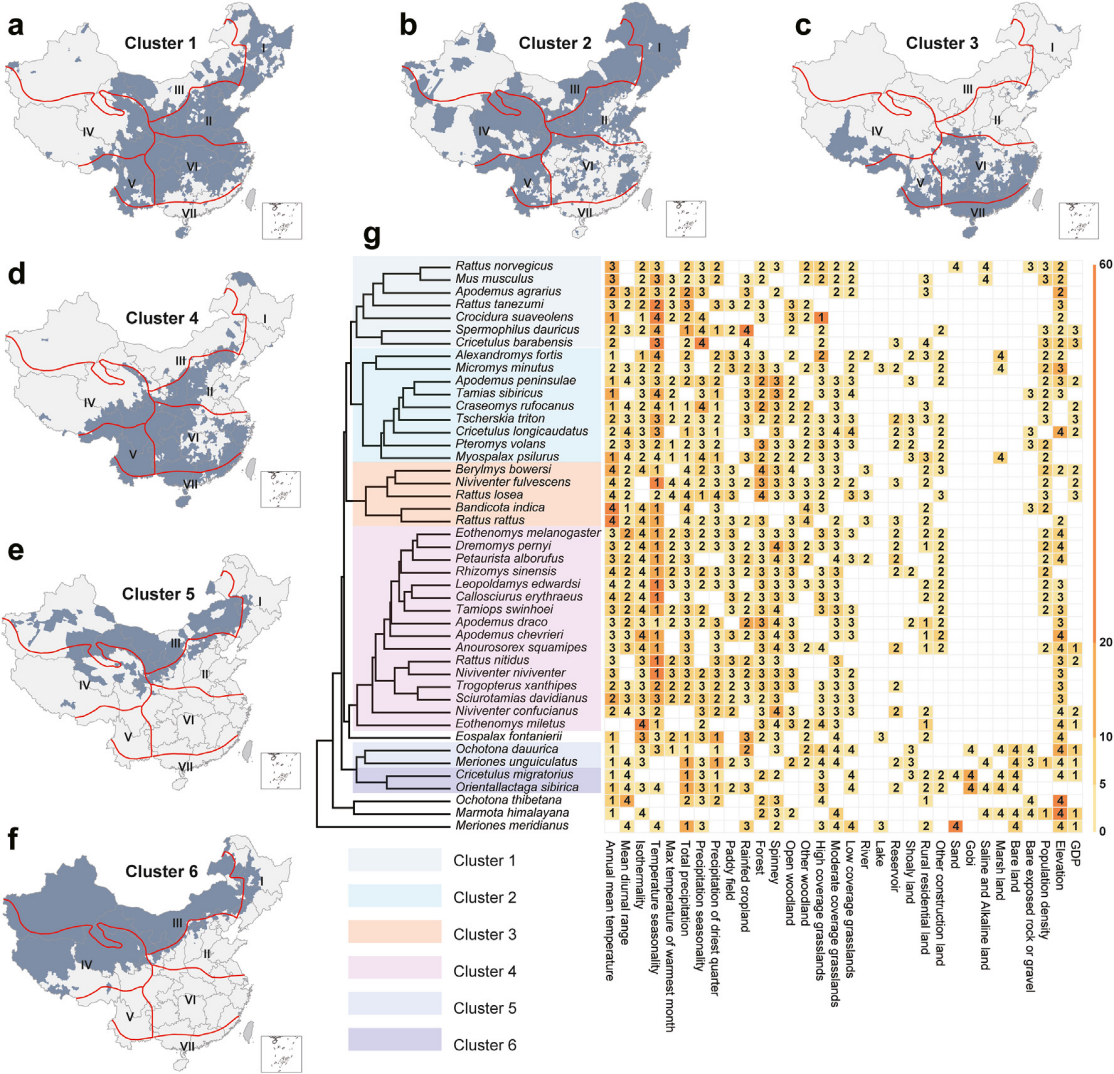
**Clustering of small mammal species based on their ecological features and the geographic distributions of these clusters at the county level.** Panels a–f indicate the geographic distributions of the six clusters (clusters 1–6). The red solid lines indicate the boundaries of the seven biogeographic zones: I = Northeast district, II = North China district, III = Inner Mongolia-Xinjiang district, IV = Qinghai-Tibet district, V = Southwest district, VI = Central China district, and VII = South China district. Panel g illustrates the dendrogram of small mammal species, with the colors indicating clusters 1–6. The features used for clustering are three quantities associated with each predictor of the BRT model for each species. Two of the three quantities are displayed in panel g: the relative contribution of each predictor (colored in ascending order from yellow to red) and the standardized median value of each predictor among counties with small mammal occurrence (numbers 1–4 indicate the position of this median in reference to the quartiles of this predictor among all counties).

Six clusters were identified from the 45 major SMs according to their similarity in the statistically significant ecological predictors. A clear geographic aggregation of the clusters is apparent based on the model-predicted distribution of the SM species in each cluster (Fig. 2). Cluster 1 ecologically fits the Northeast (I), North China (II), Southwest (V), and Central China (VI) biogeographic zones, characterized by intermediate levels of annual mean temperature, high percent coverage of grasslands, and intermediate elevations. Cluster 2 covered the vast region from northeastern to southwestern China (biogeographic zones I–V), which was characterized by high temperature seasonality. Clusters 3 and 4 were associated with low temperature seasonality that is common in the Southwest (V), Central China (VI), and South China (VII) biogeographic zones. Both clusters 5 and 6 were primarily found in Northern China, covering the Inner Mongolia-Xinjiang (III) and Qinghai-Tibet (IV) biogeographic zones. Cluster 5 was associated with high coverage of shoaly land (a type of land located in the shallow water, it may be considered marshy or sandy depending on its specific location) and intermediate coverage of grasslands. Cluster 6 was associated with low coverages of rural residential land and other construction land and a high percent coverage of gobi. Four SM species (*Eospalax fontanierii*, *Ochotona thibetana*, *Marmota himalayana* and *Meriones meridianus*) did not group into any of the clusters due to their distinct ecological niches.

### Distribution and prediction mapping for SMAMs

A total of 8910 records of SMAMs were obtained from SMs, identified by PCR (4808 records), isolation and cultivation (640 records), micrograph identification (92 records), and NGS (3370 records, results from papers) (Fig. 1a). Details on the distribution of SMAMs are provided in Appendix 1 (p 9). Of the 364 SM species, 120 (33.0%) were found to carry a total of 155 SMAMs at the species level, which were identified by non-NGS detection methods, comprising 56 viruses, 71 bacteria, 26 parasites, and two fungi (Fig. 1c; raw data in Supplementary data 3). Three SM families were hyper-reservoirs each carrying over 50 species of SMAMs, with the highest number of species carried by the family Muridae (115 species), followed by the families Cricetidae (72 species) and Soricidae (54 species). Within the family Muridae, *Rattus norvegicus* harbored the highest variety of SMAMs (75 species that included 35 bacteria, 24 viruses and 16 parasites). Other SM species that carried >40 SMAMs were *Rattus tanezumi* (68 species), the lesser ricefield rat (*Rattus losea*) (52 species), *Apodemus agrarius* (50 species), and the Asian house shrew (*Suncus murinus*) (42 species). The top three families of SMAMs that parasitize the highest variety of SM species were the Yersiniaceae (identified in 58 SM species), Bartonellaceae (identified in 52 species), and Hantaviridae (identified 51 species).

SM-associated viruses that were identified in more than ten SM species are Seoul *orthohantavirus* (infesting 30 rodent species), Hantaan *orthohantavirus* (25), Anellovirus [genus] (12), Hepatitis E virus (12), Coronavirus [subfamily *Orthocoronavirinae*] (11), and Jingmen tick virus (11).

SM-associated bacteria that were identified in more than ten SM species are *Yersinia pestis* (infesting 56 species), followed by *Bartonella grahamii* (36), *Orientia tsutsugamushi* (33), *Anaplasma phagocytophilum* (31), *Bartonella taylorii* (29)*, Bartonella tribocorum* (29)*, Leptospira interrogans* (25)*, Bartonella japonica* (23)*, Bartonella elizabethae* (22)*, Rickettsia typhi* (22)*, Bartonella queenslandensis* (16)*, Bartonella rochalimae* (16)*, Borrelia garinii* (16)*, Borrelia afzelii* (13)*, Ca. Neoehrlichia mikurensis* (13)*, Rickettsia prowazekii* (13)*, Rickettsia sibirica* (13)*, Coxiella burnetiid* (12)*,* and *Bartonella fuyuanensis* (11).

**Fig. 3:**
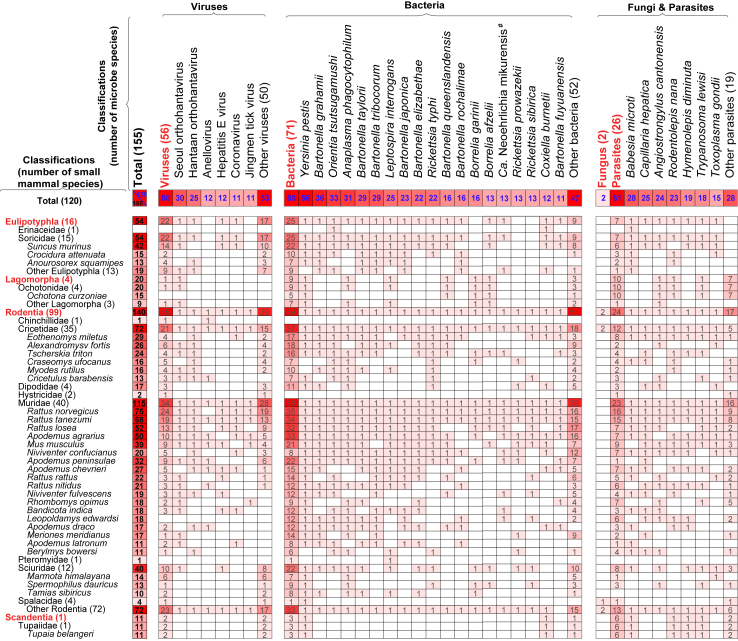
**Types of microbes detected in small mammal species in China from 1950 to 2021.** Data sources are provided in Supplementary 1 and 2. Bold blue numbers in the top row indicate the number of small mammal species each microbe infects, and bold black numbers in the first column indicate the number of microbes carried by each small mammal species. Records of small mammal-associated microbes include human cases and detections in small mammals, excluding those in arthropod vectors.

SM-associated fungi and parasites that were identified in more than ten SM species are *Babesia microti* (infesting 28 species), *Capillaria hepatica* (25), *Angiostrongylus cantonensis* (24), *Rodentolepis nana* (23), *Hymenolepis diminuta* (19), *Trypanosoma lewisi* (18), and *Toxoplasma gondii* (15) (Fig. 3; raw data are listed in Supplementary data 3 and 4).

A total of 3370 NGS records were found for 76 virus families (46 RNA virus families and 30 DNA virus families) that were carried by 61 SM species. The most frequently identified virus families were *Parvoviridae*, *Picobirnaviridae*, *Picornaviridae*, and *Herpesviridae*. There were 59 virus families exclusively identified from the NGS data, none of which can be found in the amplicon-sequencing data (Appendix 3 pp 2−4; raw data are listed in Supplementary data 3). The SMAMs detected from SMs were spatially scattered over the whole country (Appendix 3 pp 5−7), and the specific data are listed in Supplementary data 4 and 5.

**Fig. 4:**
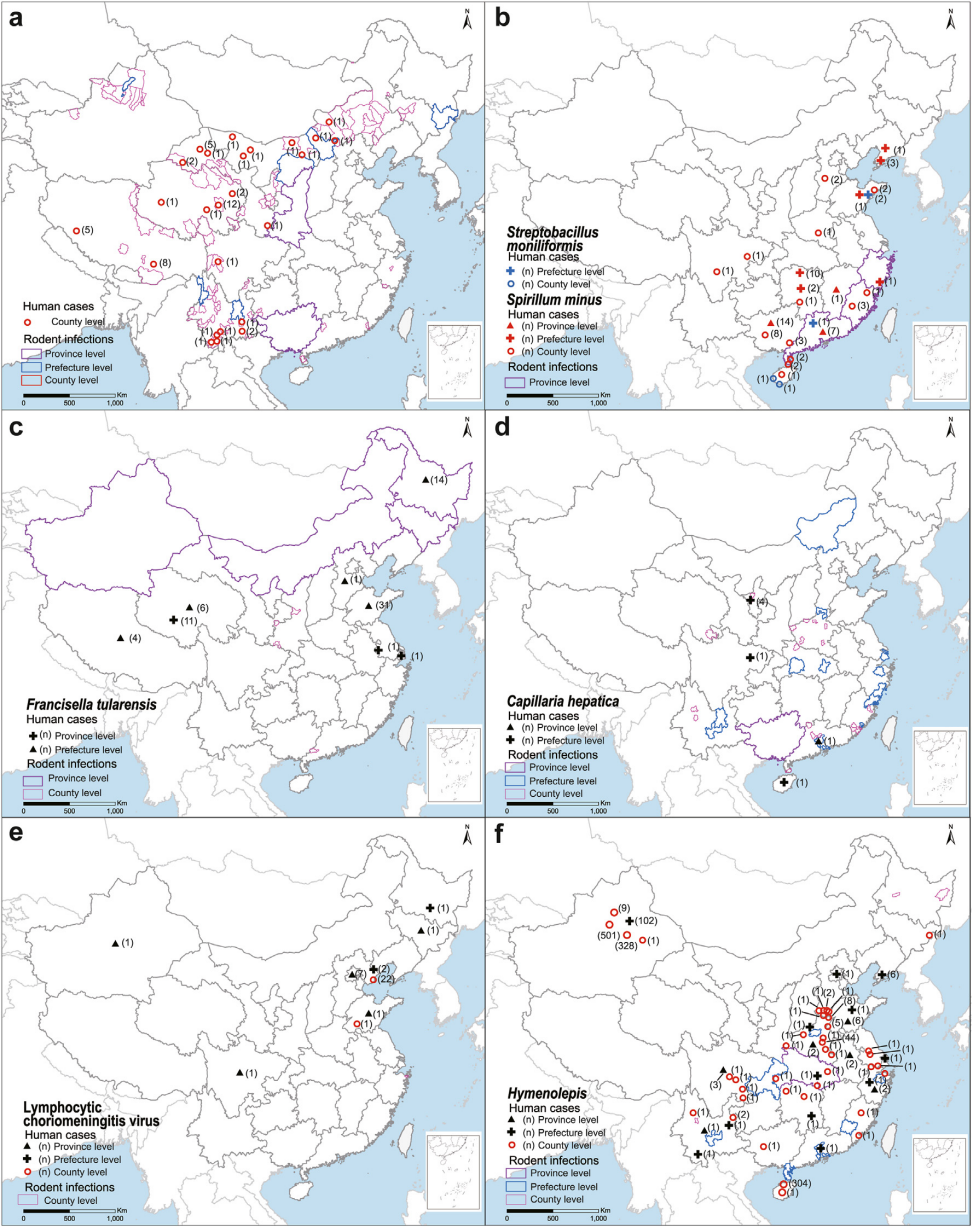
**Geographic distributions of eight small mammal-borne infections in humans and in small mammals in China.** (a) *Yersinia pestis*; (b) *Streptobacillus moniliformis* and *Spirillum minus*; (c) *Francisella tularensis*; (d) *Capillaria hepatica*; (e) Lymphocytic choriomeningitis virus; and (f) *Hymenolepis*.

**Fig. 5:**
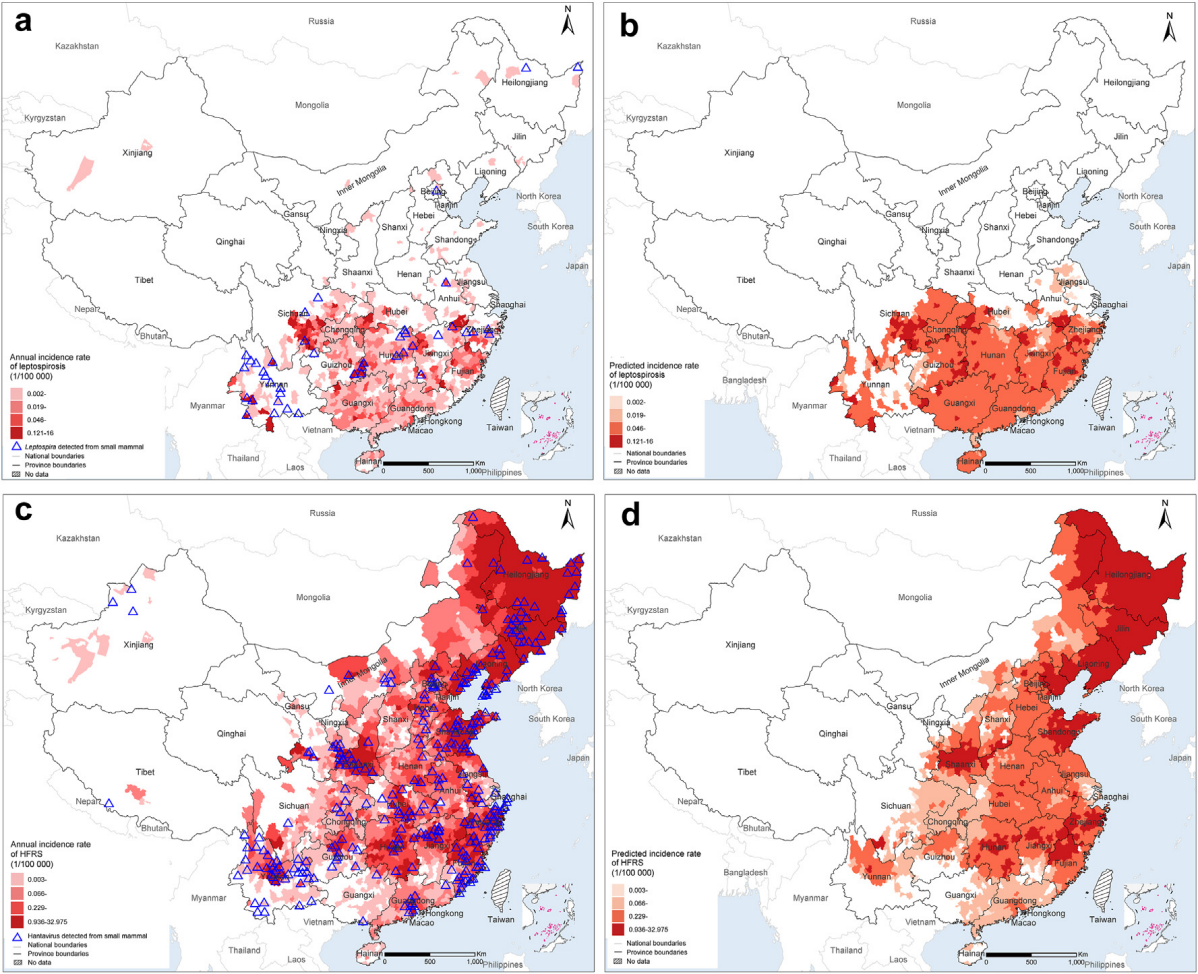
**Observed and model-predicted distributions of leptospirosis and hemorrhagic fever with renal syndrome (HFRS) at the county level in China.** (a) Reported average annual incidence rates of human leptospirosis during 2004–2020 and locations of *Leptospira* detected from small mammals during 1950–2021; (b) Spatial distribution of model-predicted incidence rate of leptospirosis; (c) Reported average annual incidence rate of human HFRS during 2004–2020 and locations of hantavirus detected by PCR and isolation from small mammals during 1950–2021; (d) Spatial distribution of model-predicted incidence rate of HFRS. Model-predicted incidence rate is calculated as he product of the presence probability predicted by the step-1 logistic GBRT model and the incidence rate predicted by the step-2 gamma GBRT model (which is set as 0 if the predicted presence probability is < 0.5).

Eight RBDs that were prevalent in China were mapped (Fig. 4; 5a and c), including plague caused by *Yersinia pestis*, rat-bite fever caused by *Streptobacillus moniliformis* or *Spirillum minus*, tularemia caused by *Francisella tularensis*, capillariasis caused by *Capillaria hepatica*, lymphocytic choriomeningitis (LCM) caused by lymphocytic choriomeningitis virus, hymenolepiasis caused by *Hymenolepis*, leptospirosis caused by *Leptospira*, and HFRS caused by *orthohantaviruses*. There are 215,791 reported cases of these eight RBDs in humans, and additional information regarding the distribution and incidence of these diseases can be found in Appendix 1 (p 10).

A total of 9582 leptospirosis cases were reported during 2004–2020, mainly from Southern and Central China, consistent with the potential high-risk regions for human cases of leptospirosis or detection of *Leptospira* in SMs predicted by the step-1 logistic GBRT model (Fig. 5a and b). Approximately 465.97 million people reside in the potential high-risk regions with a total area of 1.592 million km^2^. The step-2 gamma GBRT model predicted a high annual incidence (≥0.046/100,000 person-years, the median of the average annual incidence rates of all counties in China) of human leptospirosis cases in 540 counties, slightly more than the 501 counties with a high incidence observed. The presence of leptospirosis cases was associated with high annual temperature, medium temperature seasonality, and medium total precipitation, all contributing to the model with a RC ≥5%. Among the counties at risk of *Leptospira* occurrence, a higher case incidence was associated with low elevation (RC = 17.8%), a high model-predicted probability of *Apodemus peninsulae* (one of the most dominant reservoirs of *Leptospira*) (RC = 12.0%), and low coverage of rural residential land (RC = 10.1%). Other predictive variables with RC ≥5% were low population density (RC = 8.5%), high maximum temperature of the warmest month (RC = 6.6%), and low temperature seasonality (RC = 5.1%) (Table 2; Appendix 3 p 8).

**Table 2:** Relative contributions of major ecological factors to the presence and incidence rate of leptospirosis and hemorrhagic fever with renal syndrome (HFRS), estimated by two-step generalized boosted regression tree (GBRT) models.

Category	Variable	Leptospirosis (relative contributions %)^a^		HFRS (relative contributions %)^a^	
		Step 1 (Presence)	Step 2 (Incidence Rate)	Step 1 (Presence)	Step 2 (Incidence Rate)
Environmental	Rainfed cropland (%)	**–**	2.41 (0.58)	3.51 (0.68)	**–**
	Forest (%)	**–**	3.34 (1.10)	**–**	**–**
	Open woodland (%)	**–**	2.76 (0.59)	3.62 (0.76)	3.66 (0.49)
	Other woodland (%)	**–**	–	**–**	2.57 (0.41)
	Moderate coverage grasslands (%)	**–**	**–**	**–**	2.56 (0.54)
	Low coverage grasslands (%)	**–**	**–**	3.12 (0.77)	5.39 (1.27)
	River (%)	**–**	3.02 (0.73)	**–**	**–**
	Lake (%)	2.39 (1.01)	**–**	**–**	**–**
	Rural residential land (%)	6.14 (0.77)	10.09 (4.87)	**–**	**–**
	Other construction land (%)	**–**	3.11 (1.40)	**–**	**–**
	Bare exposed rock or gravel (%)	**–**	**–**	42.03 (3.28)	**–**
	Elevation (m)	**–**	17.78 (6.77)	10.51 (2.79)	4.10 (0.65)
Bioclimatic	Annual mean temperature (°C)	60.14 (3.47)	3.53 (1.01)	3.30 (0.81)	18.28 (2.77)
	Isothermality	**–**	**–**	4.55 (2.30)	2.58 (0.40)
	Temperature seasonality	7.08 (1.35)	5.10 (1.57)	7.02 (1.01)	9.09 (2.77)
	Mean diurnal range (°C)	3.68 (0.74)	**–**	**–**	4.41 (0.83)
	Max temperature of warmest month (°C)	4.83 (0.67)	6.55 (1.37)	**–**	2.04 (0.32)
	Total precipitation (mm)	11.55 (2.86)	3.05 (0.77)	7.93 (1.32)	8.53 (1.48)
	Precipitation seasonality (mm)	**–**	**–**	**–**	2.63 (0.42)
	Precipitation of driest quarter (mm)	**–**	3.13 (0.57)	–	12.20 (1.30)
Social	Gross domestic product GDP (CNY)	**–**	1.92 (0.72)	4.53 (2.05)	3.12 (0.43)
	Population density (persons per km^2^)	**–**	8.49 (2.67)	–	2.63 (0.29)
	Proportion of ≥60 years old	**–**	–	–	2.44 (0.37)
	Number of clinics hospitals	**–**	3.14 (0.72)	–	–
Biological	presence of *Apodemus peninsulae*^b^	**–**	12.02 (5.68)	5.04 (0.93)	5.78 (1.42)
	presence of *Tscherskia triton*^b^	**–**	3.06 (0.86)	–	2.21 (0.30)
	presence of *Rattus losea*^b^	4.19 (0.55)	**–**	**–**	**–**
	presence of *Rattus tanezumi*^b^	**–**	4.54 (1.40)	**–**	2.74 (0.79)
	presence of *Apodemus agrarius*^b^	**–**	2.95 (1.22)	4.84 (1.12)	3.06 (0.42)

a The relative contributions were shown as mean (standard deviation) across 100 resampled data sets. Step 1 logistic GBRT model was fitted to the presence/absence of any reported human case or pathogen detection in small mammals. Step 2 gamma GBRT model was fitted to the average annual average incidences from 2004 to 2021 counties with predicted presence probability >0.5. “–” indicates the factors was not included in the whole model.

b The presence probability predicted by the BRT models for major small mammal species.

A total of 204,601 HFRS cases were reported in China during 2004–2020, mainly identified between the longitudes of 98–135° East (Fig. 5c). Based on the step-1 logistic GBRT model, predicted high-risk areas largely resembled those associated with actually recorded cases. However, the step-2 gamma GBRT model predicted a high incidence (≥0.229/100,000 person-years, the median of the average annual incidence rate of all counties in China) in 1477 counties, substantially more (66%) than the 892 counties that reported a high incidence (Fig. 5d). The step-1 GBRT model showed statistically significant associations between a high risk of *orthohantavirus* occurrence and low coverage of bare exposed rock or gravel (RC = 42.0%), low elevation, medium to high temperature seasonality, medium to high temperature seasonality, and a high model-predicted probability of *Apodemus peninsulae* which is a major animal reservoir of *orthohantavirus* (Table 2). Among counties at potential risk of hantavirus occurrence, the step-2 gamma GBRT model revealed that a low to medium annual mean temperature was the leading contributing factor to the incidence of HFRS (RC = 18.3%), followed by relatively high precipitation of the driest quarter (RC = 12.2%), high temperature seasonality (RC = 9.1%), medium to high total precipitation (RC = 8.5%), a high model-predicted probability of *Apodemus peninsulae* presence (RC = 5.8%), and low coverage of grasslands (RC = 5.4%) (Table 2; Appendix 3 p 9). The results were similar in the sensitivity analysis where serological detection of *orthohantavirus* in SMs was also considered evidence of presence in the step-1 GBRT model, except that the model-predicted probability of *Apodemus peninsulae* was no longer an influential contributor (Appendix 1 p 31 and Appendix 3 pp 10−11).

## Discussion

SMs are thought to be the most speciose group of animals and comprise a disproportionate number of globally important reservoir species for zoonotic pathogens.^2^^,^^3^^,^^21^ In this study, we performed an exhaustive search of the literature in both English and Chinese as well as in the GenBank and NGS databases to assemble an up-to-date, comprehensive dataset on county-level detections of 120 SM species (out of all 364 SM species recorded in mainland China) and 155 associated SMAMs in China at both family and species levels, spanning a period of over 70 years. Based on this dataset, the ecological drivers of the dominant SM species and two RBDs associated with high disease burden in China were evaluated and potential high-risk areas were mapped. A recent global study described 95 pathogens detected in 285 SM species up to the year 2020.^2^ Our study can be considered a valuable extension focusing on China and based on more comprehensive literature search. Our study characterized the biodiversity, spatial distribution, and ecological niches of SMs and associated SMAMs in China at an unprecedented level of detail and can inform and guide global risk assessments of RBDs and targeted field surveillance of SMAMs.

Leveraging the unprecedented data volume, many of our ecological modeling results for SMs corroborate with previous research and offer new insights. For example, Tian^23^ investigated ecological niches of *Spermophilus dauricus* in China from 2000 to 2015 and found that it was primarily distributed in arid and semi-arid regions of North China featuring moderate temperature, low precipitation, and sparse vegetation, which aligned with our findings (Fig. 2, Appendix p 28). Our BRT model discovered temperature and precipitation seasonality as additional drivers and provided a higher test AUC (0.93) than Tian's MaxEnt model (0.91). Lu, et al.^24^ found that *Marmota himalayana's* ecological niche was mainly determined by temperature, elevation, vegetation coverage and soil type, using survey data from a single county and a single year. Based on nationwide data form multiple years, our study identified elevation as the most influential driver, followed by coverage of moderate-density grassland, GDP, population density, coverage of marshland, and coverage of forest. Though not appearing as a top driver, temperature was associated with an RC >3%.

SMs often share their habitats with farm animals, enabling ample opportunities for transmission of SMAMs to the farm animals. Additionally, SM habitats, particularly those in urban areas, play a crucial role in connecting rural and urban disease hotspots. This connection greatly increases the risk of emerging zoonotic pathogen spillover, which in turn can lead to sporadic human cases.^5^^,^^12^^,^^25^, ^26^, ^27^ The diversity and disease burden of RBDs has remained understudied because most field investigations target notifiable infectious diseases. The variety of SMAMs that can cause human infection and their distributions are poorly understood due to non-specific clinical manifestations and the lack of etiological testing in clinical practice.^28^^,^^29^ Remarkably, there was a clear distinction between the geographic distributions of human cases and the associated SMAMs detected in SMs for five neglected RBDs that were not included in the list of the notifiable disease surveillance system (CISDCP), including rat-bite fever, tularemia, capillariasis, LCM, and hymenolepiasis. For example, rat-bite fever cases had been reported in 12 provinces, while small mammal infections with *Spirillum minus* and *Streptobacilus moniliformis* were only recorded in three coastal provinces, possibly a result of insufficient field investigation. In addition, five rodent-borne microbes with pathogenic potential in humans, including Amur virus, Dobrava-Belgrade virus, Tula virus, *Rickettsia conorii* and *Bartonella bacilliformis*, have not been found to cause humans cases in China.^30^, ^31^, ^32^ However, it should be noted that *Rickettsia conorii* is pathogenic in humans and tick-borne. Despite the increasing knowledge about neglected RBPs, the natural history of disease associated with these infections has yet to be elucidated, making it difficult to effectively assess the risk of outbreaks and develop prevention strategies.

Many SMAMs identified in our study, such as murine *picobirnaviruses*, parvoviruses, and herpesviruses, are not regarded as zoonotic, nor do they pose apparent threats to companion animals or livestock. However, we recommend strengthening surveillance of the SMs hosting these SMAMs, especially those with habitats near human communities and farms, to promptly identify potential events of crossing species barrier that could imply new threats to human or animal health. Such a surveillance system will demand close collaboration among different stakeholders including but not limited to public health agencies, agricultural departments, farmers, and veterinary institutes.^33^

Our findings highlight the crucial role of meteorological factors in the ecology of SMs and SMAMs. Temperature seasonality and annual mean temperature were influential predictors for the ecological niches of ≥30 of the 45 identified SM species, especially for the hyper-reservoirs of SMAMs such as *Rattus norvegicus*, *Mus musculus*, and *Apodemus agrarius.* These two variables also contribute significantly to the ecology of *Leptospira* and *orthohantavirus*, even after adjusting for the dominant reservoirs. Climate change, together with changes in land use and human mobility, have directly and indirectly reshaped biodiversity. There are increasing opportunities for pathogens to cross-infect previously geographically-isolated species of wildlife and an increasing potential for human-wildlife contact resulting in pathogen spillover to humans.^11^^,^^25^^,^^27^^,^^34^, ^35^, ^36^, ^37^, ^38^, ^39^ Recent studies have indicated that certain taxa of SMs are more prone to zoonotic transmission than others, and these animals often thrive in human-dominated landscapes and facilitate the emergence of RBDs.^27^^,^^40^^,^^41^ Intriguingly, these hyper-reservoirs tend to be even more capable of zoonotic transmission when biodiversity is low,^27^^,^^40^^,^^41^ suggesting that host biodiversity and the prevalence of zoonotic transmission is a dynamic process and their relationship is not necessarily linear.

We identified six clusters of SM species that share similar ecological niches and geographic distributions. Substantial geographic overlap was seen among these clusters, likely a result of (1) diverse but overlapping climate and landcover conditions in China providing resources for different SMs to flourish, and (2) flexible ecological adaption of some SM species to environmental variation. These clustering patterns could inform risk assessments. For instance, *Rattus norvegicus* and *Apodemus agrarius,* the two rodent species in Cluster 1, harbor 75 and 50 SMAMs respectively and act as efficient vectors for multiple human pathogens such as *orthohantavirus*, *Yersinia pestis*, and *Leptospira*. Other SMs, especially rodents, in this cluster should be closely monitored for possible cross-species transmission of microbes that can be pathogenic in humans. In future research, the ecological determinants of each cluster as a whole should be investigated, and ecological modeling should be conducted at different spatial scales. The relevance of bioclimatic factors could be even more prominent at larger scales such as prefecture and province, compared to the county-level in our analysis. This phenomenon can be explained by the scale effect,^42^ which means that the characteristics of environmental factors are manifested differently at various spatial scales. Nevertheless, looking at large scales alone can subject results to ecological fallacy.^43^ Conversely, if we move down to scales finer than county, bioclimatic conditions may be less relevant, and other factors such as vegetation cover may become more dominant. Future research should explore this multi-scale approach in greater detail.

In China, there is no indication that a rodent species would have disappeared entirely from a county after its detection in the early years, although this scenario cannot be ruled out. A more common trend is geographic expansion via either ecological adaption or improvement in detection efforts, especially the latter, which justifies our use of cumulative occurrence data for ecological modeling. Among the 45 major SM species, 38 (84%) had 40% records on or before year 2000, where 31 (69%) had 40% records after 2000 (Appendix 1 p 32). For these species, separate modeling for the two periods, 1950–2000 and 2001–2021, would be difficult as data in one of the periods are sparse. For species with relative balanced and adequate numbers in both periods, we can perform separate modeling as a sensitivity analysis. For example, we did so for *Mus musculus* and *Rattus norvegicus*, as the two species have the most abundant data (Appendix 1 p 32). We compared the model-predicted distributions using all data (1950–2021) vs. using early data (1950–2000), and we do not see qualitative difference in geographic distribution (Appendix 3 pp 12−13). In addition, the top important factors for ecological niches also largely overlap between the all-data model and the early-data model, e.g., top three predictors fully overlap for both *Mus musculus* and *Rattus norvegicus* (Appendix 1 p 33). These results confirm the validity of our modeling analyses.

Our study had a few limitations. The survey locations of SMs were unlikely sampled randomly, and a certain level of bias may exist in the modeling results, despite the use of inverse probabilities of survey for weighting individual counties in the ecological models. On the other hand, the sampling bias is unlikely to be large because the 1660 surveyed counties accounted for 57% of all counties in mainland China and provide a representative coverage of all biogeographical zones. Another possible source of bias may be the lack of adequate laboratory testing and reporting capacities in remote and rural areas which can affect the quality of field detection of SMAMs and reporting of RBD cases leading to an underestimation of risks. In addition, in our ecological modeling for SM, the definition of a “case” as a county with ≥1 recorded occurrences could be overly loose, as the detection could be due to transient migration rather than ecological establishment. We conducted a sensitivity analysis using ≥2 recorded occurrences as the definition of a “case” county for *Mus musculus* (Appendix 3 p 14) and *Cricetulus barabensis* (Appendix 3 p 15). The observed and model-predicted distributions are qualitatively similar between the two definitions, though the original definition was associated with a slightly wider range of high-risk areas for *Cricetulus barabensis*. The identified risk factors of high importance were largely similar between the two definitions. Finally, for RBD patients reported by the national surveillance system CISDCP, the residential address at symptom onset was used as a proxy for the exposure site, which could lead to erroneous geolocations for traveled patients if the incubation period was relatively long, e.g., the incubation period of leptospirosis and HFRS may last for weeks. For patients identified via literature search, the reported exposure site was used for geolocation which could be subject to recall bias.

Despite the caveats mentioned above, our systematic review and modelling analyses provide a timely and comprehensive update describing the geographic distributions and ecological drivers of SM species, associated SMAMs and important RBDs in China. The results of the BRT and GBRT models confirmed our hypotheses that the ecological niches of SMs and related infectious diseases are highly predictable using environmental variables and that the predicted distributions are larger than the observed ones. The risk maps can aid the monitoring of existing and predicted hotspots for novel SMAMs, cross-species transmission events and zoonotic spillovers. This study highlights the need for policymakers to consider a much wider geographic scope and the increasing variety of animal hosts when developing strategies for disease prevention and public awareness campaigns. Surveillance of SMAMs with pathogenic potential in humans should be supplemented with research in rapid diagnostic tools, effective vaccines and therapeutic treatments. On the clinical side, our results can help clinicians better screen for etiologic pathogens based on exposure information provided by patients. We call for more field surveys that are carefully designed, well documented, and openly accessible to facilitate risk assessments and guide the prevention and control of zoonotic diseases associated with small mammals.

## Contributors

LQF, WL, YY and SIH conceived, designed, and supervised the study. JJC, CLL, TW, YHW, TLC, QX, XGH, AYT, ST, YYZ, MCL, and LPW performed the literature review, data collection, and integration. WL and LQF verified the data and had access to the raw data. JJC, CLL, TW, and YHW conducted the analyses under the supervision of LQF, WL, YY and SIH. LCL, TW and YHW helped with the analyses. JJC, CLL, and LQF drafted of the manuscript. LQF, WL, YY and SIH interpreted the findings and revised the manuscript. All authors read the manuscript, provided feedback, and approved the final version. LQF, WL, YY and SIH had final responsibility for the decision to submit for publication.

## Data sharing statement

Data will be made available upon request made to the corresponding author.

## Editor note

The Lancet Group takes a neutral position with respect to territorial claims in published maps and institutional affiliations.

## Declaration of interests

We declare no competing interests.

## Appendix A Supplementary data

Supplementary data 5

Supplementatry Appendix 1

Supplementatry Appendix 2

Supplementatry Appendix 3
