# Supplementary material for: Small mammals and associated infections in China: a systematic review and spatial modelling analysis
Source: Lancet Reg Health West Pac. 2024 Dec 18;54:101264. doi: 10.1016/j.lanwpc.2024.101264 (PMC11728903; doi:10.1016/j.lanwpc.2024.101264)
Supplement: Supplementatry Appendix 1 [file mmc6.pdf]

Supplementary appendix 1

Supplement to: Small mammals and associated infections in China: a systematic review and spatial modelling analysis

Table of Contents 1

Appendix Text S1: Data sources and dataset pooling 2
Appendix Text S2: Ecological modeling...4
Appendix Text S3: Clustering analysis of small mammals with similar ecological niches and their spatial distribution 6
Appendix Text S4: Population at risk for main rodent-borne diseases (RBDs) with high disease burden 7
Appendix Text S5: The integrated database and distribution of small mammal species 8
Appendix Text S6: The distribution of small mammal-associated microbes (SMAMs) detected from small mammals in China 9
Appendix Text S7: The distribution of rodent-borne diseases (RBDs) in China 10
Appendix Table S1: The laboratory tests used to detect small mammal-associated microbes (SMAMs) in the reviewed studies...12
Appendix Table S2: Information of data sources in this study..13
Appendix Table S3: Potential risk factors for small mammal species, hemorrhagic fever with renal syndrome (HFRS) and leptospirosis. 14
Appendix Table S4: The list of small mammal-associated microbes and corresponding references. 17
Appendix Table S5: Clustering analysis of eco-climatic predictors at the county level based on pairwise Pearson correlation coefficients...21
Appendix Table S6: BRT-model-estimated relative contributions to the spatial distribution of Cricetidae family...22
Appendix Table S7: BRT-model-estimated relative contributions to the spatial distribution of Muridae family...24
Appendix Table S8: BRT-model-estimated relative contributions to the spatial distribution of Muridae family...26
Appendix Table S9: BRT-model-estimated relative contributions to the spatial distribution of Sciuridae family. 28
Appendix Table S10: BRT-model-estimated relative contributions to the spatial distribution of other family. 29
Appendix Table S11: Relative contributions of major factors to the spatial distributions of hantavirus detected by PCR, isolation, and serological methods..31
Appendix Table S12: The number of 45 species of small mammals in the counties of 1950–2000 and 2001–2021...32
Appendix Table S13: Relative contributions of the top six predictors in the ecological models for *Mus musculus* and *Rattus norvegicus* based on data from 1950-2021 and 1950-2000..................33
Appendix References 34

Appendix Text S1: Data sources and dataset pooling

Data on small mammal, small mammal-associated agents and rodent-borne diseases
We assembled a comprehensive database of small mammal-associated microbes (SMAMs) from a variety of sources, including (1) literature reporting the occurrence of rodent-borne diseases (RBDs) published between January 1950 and December 2021; (2) the reported incidences of notifiable RBDs from the China Information System for Disease Control and Prevention (CISDCP); (3) location data of small mammals from Figshare, GBIF (https://www.gbif.org/), IUCN (https://www.iucn.org/), and the location data of SMAMs from GenBank that is the National Institutes of Health (NIH) genetic sequence database, an annotated collection of all publicly available DNA sequences;[1] (4) and surveillance reports on key infectious diseases and vectors in China. Clinically diagnosed or laboratory-confirmed haemorrhagic fever with renal syndrome (HFRS), leptospirosis and plague cases at clinics and hospitals are reported, as mandated by the Ministry of Health, to CISDCP. The county-level data of human cases of identified origin of HFRS and leptospirosis during 2004–2020 were collected from the Chinese Scientific Data Center for Public Health (http://www.phsciencedata.cn) and were used in the ecological models for RBDs, and excluded cases with a history of travel when modeling for leptospirosis and HFRS.

For the small mammal, we collected the Figshare, GBIF, and IUCN database to our database of small mammal, not including those located in museums in the both databases. For the literature review, four main electronic databases (Web of Science, PubMed, China National Knowledge Infrastructure (CNKI), and China WanFang database) were searched for studies published between January 1950 and December 2021, using the following key-words: ("*mammal*" or "*pathogens*" or "*virus*" or "*bacterial*" or "*microbiology*" or "*diseases*" or "*infections*") and "China" in English and Chinese. We also checked the references in retrieved articles to reach more relevant articles. Each article was carefully reviewed by two team members independently to collect the following information using a standard form: time and location of investigation, type of samples, spatial resolution, small mammal species identified, number of small mammal samples for test, laboratory test methods applied and test results for SMAMs. Any disagreement between the two staff members was resolved by discussion and consensus among the reviewers and other co-authors. Only studies with exact data and clear description were included in our database. For articles containing ambiguous data, the original authors were contacted for clarification; if the ambiguity was not clarified, the data in question were excluded from the study. If SMAM was reported more than once in the same county (e.g., through seasonal collections or by different study groups, or in more than one township within a county) during the study period, it was counted only once at the county level.

The SMAMs included in the study were detected from small mammals by any of the following laboratory tests: real-time polymerase chain reaction (RT-PCR), PCR, next-generation sequencing, isolation or culture, or smear microscope. For all SMAMs, positive serological detection in a reservoir was not considered conclusive, due to poor test specificity and cross-reactivity with other SMAMs. Such reservoirs were classified as "suspected reservoirs" unless serologic detection was followed by a confirmatory test. For infection in humans, those displaying a four-fold increase in titer of specific antibodies, a seroconversion of specific antibodies determined from the acute and convalescent disease, or specific IgM antibodies determined from the acute phase of illness, were also included. From GenBank, in addition to the above-mentioned information, the accession number, the sequencing and genotypes if available of the tested SMAMs were additionally extracted. The above data on RBDs were used for the mapping and modelling in this study. The data from the literature and GenBank were integrated to form one database at the county level for mapping. In addition, the data on the cross-sectional survey of specific antibodies of RBDs in humans were also extracted only for displaying their probable distribution of them, which was not included in the modeling analysis.

Data on environmental, social, bioeclimatic and biological variables

We collected a variety of environmental, social, and bioclimatic variables that are commonly used in ecological studies on small mammal species and RBDs. The choice of variables is mainly based on empirical ecological evidence in the literature and their spatial variability. In addition, we focus on environmental variables that are potentially shared by multiple species so that the results can be compared across species. The 38 years (from 1981 to 2018) of climatic data were collected from 2006 weather surveillance stations in the mainland of China, covering 74.0% of 1228 surveyed counties (http://cdc.nmic.cn/home.do). The bioeclimatic data include average monthly meteorological variables such as temperature, maximum temperature, minimum temperature, relative humidity, and rainfall during the 38 years. For the 877 counties without meteorological stations, the mean values of the nearest five surveillance stations were used as a proxy for their meteorological variables. From these longitudinal meteorological variables, 19 cross-sectional ecoclimatic variables (BIO01−19, also called bioclimatic variables recommended by the U.S. Geological Survey) were calculated and their yearly averages were used as predictors in our machine learning models. These bioecoclimatic variables better capture the seasonal trends of different species related to their physiological constraints than traditional meteorological variables and have been widely used in ecological studies.[2]

The environmental data in China were regularly updated every 5–10 years since 1980. Raster-type land cover data of China during 1985–2015 with a resolution of one square kilometer were obtained from the National Earth System Science Data Sharing Infrastructure (http://www.geodata.cn). We used the average value of land cover data from 1980–2015 for modelling the distributions of small mammal species and RBDs. The population data at the county level were derived from the Sixth National Census of China in 2010. Social data including the numbers of hospitals, clinics, emergency center, the percentage of the female population, and the percentage of residents ≥60 years old were obtained from the national bureau of statistics.

In total, 52 environmental (24), social (nine), bioclimatic (19), and biological variables (seven) at the county level were extracted from these data using the ArcGIS Desktop 10.7.0.10450 software (ESRI Inc., Redlands, CA, USA). Data cleaning and reorganization were performed by the statistical software R Program Version v4.0.3.

Appendix Text S2: Ecological modeling

We built Boosted Regression Tree (BRT) models at the county level to identify environmental, social, bioclimatic and biological determinants for the geographic distribution of small mammal species, as well as to predict high-risk areas where the small mammals might exist.

To avoid overfitting and to improve interpretability of the models, we screened for multicollinearity among candidate predictors, due to their potential correlation with each other. We performed a clustering analysis on these predictors based on their pairwise correlations using the R package "NbClust" (https://cran.r-project.org/web/packages/NbClust/index.html/). Specifically, a binary distance matrix was formed with the distance between any pair of eco-climatic variables being 0 if the absolute value of the correlation coefficient is bigger than 0.8 and 1 otherwise. The best number of clusters was chosen by the Krzanowski and Lai index.[3-5] This clustering analysis found six clusters of the bioclimatic. A continuous distance matrix where the distance is one minus the absolute value of the correlation coefficient also identified the same clusters. Only one predictor from each group was used for model-fitting.

At the first step, we excluded records that were determined only in prefectures or provinces. Then based on whether all referred small mammals collected were identified at the collection location, each record was characterized by field investigation types (complete investigation or incomplete investigation). A "case-control" (presence-absence) study design was utilized to construct predictive boosted regression tree (BRT) models at the county level for the 45 major small mammal species with recorded occurrence in ≥50 counties (comprising of 19 species in Muridae, eight in Cricetidae, seven in Sciuridae, three in Pteromyidae, three in Spalacidae, two in Ochotonidae, two in Soricidae, and one in Dipodidae). For each given small mammal species, counties with at least one record of occurrence were considered as "cases", and those conducting complete investigation yet lacking any evidence of occurrence were considered as "controls".[6] The numbers of "cases" and "controls" for each small mammal species were listed in Supplementary Data 1. The remaining counties where small mammal surveys have not been conducted or have not yielded conclusive findings were excluded from the model building but were included for risk mapping.

A Boosted Regression Trees (BRT) model at the county level have been used to fit to the training set to assess the contributions of bioclimatic, environmental and biological predictors to the geographic distribution of the given small mammal species. The BRT model is a popular approach to ecological studies and has been widely used for risk mapping of infectious diseases such as avian influenza, rabies, and helminth.[4,5,7-10] The BRT model couples the advantages of two algorithms, regression trees, and machine learning techniques, and allows nonlinear relationships between outcomes and covariates and multicollinearity among covariates.[11] A BRT model was fitted to the training set to analyze the environmental, social, bioclimatic, and biological predictors for their contribution in determining the presence of each given small mammal species. The fitted model was used to project risk levels in counties without small mammal surveys.

To counterbalance the potential sampling bias of the surveyed counties, we built a logistic regression model with "surveyed" vs. "not surveyed" as the binary outcome and all environmental, social, and bioclimatic variables as predictors. The response of this model was one for all small mammal-surveyed counties and zero for unsurveyed counties. The predictors were chosen using a backward procedure at the significance level of 0.05. The reciprocals of predicted sampling probabilities of all surveyed counties were first rescaled to have a mean of one and then used as weights in the BRT models for the 45 major small mammal species.

BRT modelling was accomplished with the R (v4.0.3) packages "*dismo*" and "*gbm*", and predictive performance was evaluated with packages "*ROCR*" and "*Proc*". Multicollinearity among candidate predictors was first screened to reduce overfitting and improve interpretability of the models (appendix 1 table S5).[5,12] A tree complexity of five, a learning rate of 0.005 and a bagging fraction of 75% were used for the primary analysis based on their satisfactory performance in our previous research. [4,5,13] Bagging is a procedure that resamples data points to fit sequential trees so to improve predictive performance. A 10-fold cross validation was used to identify the optimal number of trees using the gbm step function in the R package "dismo". The output of a BRT model consists of both predicted probabilities of occurrence and relative contributions (or influences) of predictors. The relative contribution is calculated based on how many times a predictor is chosen for splitting and how much each split improves the objective function, averaging over all trees. These relative contributions of all predictors are standardized so that they sum to one. [4,5]

A two-stage resampling procedure was employed to provide a more robust and parsimonious estimation of model parameters. In each stage, the following split-and-fit step was repeated for a certain number of times. A training set with 75% of data points was randomly selected by resampling

without replacement, and the remaining 25% served as a test set. A BRT model was built using the training set, and then applied to the test set for validation if needed. The first stage is a screening stage, in which the split-and-fitting step was repeated ten times to screen important predictors. Validation of the trained model using the test set was not performed in this step. Predictors that had a relative contribution <2% for all bootstrap training sets were excluded from the next stage. In the second stage, the split-and-fitting step was repeated for 100 times using the remaining predictors. As no variable selection was performed in this step, all 100 models had the same predictors but yielded different contribution estimates. The relative contributions of the predictors were averaged over the 100 BRT models to represent their final relative contributions. The receiver operating characteristic curve (ROC) curves and areas under the curve (AUC) based on the test sets were also averaged to represent the final predictive performance. The standard deviations and 95% percentiles of the relative contributions and AUCs across the 100 models were used to quantify the uncertainty in the estimation. Considering that there could be false negative and false positive counties in the observed data, we also calculated partial area AUC with a tolerance level of 0.2 for omission error. For partial area AUC, the horizontal axis is the total rate of positives rather than false positives. We presented the ratio of the partial AUC to the area under the random selection line (diagonal line) as suggested by Peterson et al. Finally, the predicted probabilities were averaged over the 100 models to represent the final estimates of the county-specific probabilities of presence, which were mapped for the 45 main small mammal species. BRT Modeling was conducted using the R packages *dismo* and *gbm*, and predictive performance was assessed using ROCR and pROC in the R v4.0.3 environment. We also performed a sensitivity analysis using a learning rate of 0.01 for selected small mammal species but found no substantial difference in the contribution estimates. Due to both the data size and the number of models runs ([45 small mammals] × 100), we cannot afford a full cross-validation optimization for all model configuration parameters.

To define model-predicted high-risk counties for each small mammal species, a cut-off value was chosen to maximizes sensitivity + specificity along the average ROC curve over the 100 BRT models.[5,12] Counties with predicted probabilities above the cut-off value for a given model were considered as having a high risk of distribution range for the corresponding small mammal species. For each small mammal species, the number, area, and population size of model-predicted high-risk counties were compared to the quantities of counties with observed occurrence (table 1).

Appendix Text S3: Clustering analysis of small mammals with similar ecological niches and their spatial distribution

To explore similarity in ecological niches among the 45 major small mammal species, a hierarchical cluster analysis based on the weighted-average linkage method was performed.[14]

Features used for clustering were formed as the following. We first excluded predictors that are not influential (excluded from final models) for all 45 small mammals. For each small mammal species, three indexes associated with each remaining ecological predictor were calculated as features for clustering. (1) The average relative contribution (RC) of each significant predictor in the 100 BRT models based on 100 resampled data sets, where RC is an indicator for the importance of a predictive factor to the ecological suitability; (2) A measure for the difference in each significant predictor between presence counties and all counties. We first calculated the median value of this predictor among all case counties and quartile intervals of the predictor among all counties in the nation. For each of the significant predictors, we calculated the median value of the predictor among all “case” counties and quartile intervals of the predictor among all counties in the nation. We then assigned one of the numbers 1–4 according to which quartile interval the median lies in, e.g., assign 1 (4) if the median lies in the lowest (highest) quartile. (3) The linear correlation between each significant predictor and model-predicted presence probabilities of the given rodent species among all counties. These three quantities of all ecological predictors jointly serve as features for clustering. A predictor is considered a significant contributor to ecological suitability if its average RC across the 100 BRT models was above 5.

A dendrogram was created to demonstrate the clustering pattern of these 45 small mammal species, together with a thematic matrix illustrating the features (figure 2). This matrix has small mammal species as rows and predictors as columns, and color of each cell in the matrix shows the average relative contribution and the number shows the quartile (1–4 for 1st–4th quartiles) location of the median of cases. To map geographic distributions of the identified clusters of small mammal species at the county level, we define the presence of a cluster in a given county as the presence of any small mammal species in that cluster. We then mapped geographic distributions of the identified clusters of small mammal species at the county level, where a cluster is considered present in a given county if any small mammal species in that cluster is present.

Appendix Text S4: Population at risk for main rodent-borne diseases (RBDs) with high disease burden

Generalized Boosted Regression Tree (GBRT) models were built at the county level to explore potential risk drivers for the presence and incidence (in terms of human case incidence) of hantavirus and *leptospira*. GBRT is the generalized version of BRT to handle outcomes with special distributions such as binomial, Poisson, Gamma, etc. in which the loss function is related to the likelihood, and we used the version implemented in the R package "*xgboost*" (R Core Team, 2013).[15] For presence and absence of disease, all counties where the viruses were detected in small mammals according to literature or human cases of the associated RBDs were reported by surveillance were regarded as presence. For each agent, in addition to the same 45 potential predictors, additional predictors, including nine social factors and presence possibility of seven small mammal species (*Apodemus peninsulae*, *Tscherskia triton*, *Rattus losea*, *Rattus tanezumi*, *Mus musculus*, *Apodemus agrarius*, *Rattus norvegicus*), were used for GBRT model for the presence and incidence of disease.

We fitted two independent GBRT models to predict the presence as well as incidence of disease. At the first step, a logistic model was used to fit the presence/absence of viruses by county. This step accounts for the excessive amount of zero case numbers in the majority of the counties. In this step, similar to the model of BRT model for small mammals, the cut-off values based on maximum sensitivity + specificity were calculated to estimate the presence of agents. In the second step, a gamma distribution was fitted to the non-zero annual average incidences from 2004 to 2020. The gamma distribution was chosen because it best fits the observed non-zero annual incidences. In both seps, the models were run with a learning rate of 0.05, a max tree depth of 8, and a bag fraction of 0.75. The best number of trees (nrounds option in Xgboost) was chosen by 5-fold cross-validation. Similar to the ecological models of small mammals, in each step, we first screened predictors with 10 resampled data sets (random split into 75% training and 25% testing but only the training set was used), and we excluded predictors with average relative contributions <1·5% from further analyses. The remaining predictors entered the second stage and the GBRT model was fitted on 100 resampled data sets (random split into 75% training and 25% testing), and the final results were averaged over the 100 models.

This two-step modeling approach is similar to the traditional hurdle model and the zero-inflated model in the realm of generalized linear models which are also designed to account for excessive zero counts. However, the two steps in our approach are independent in terms of separate optimizations, whereas both the hurdle model and zero-inflated model are likelihood-based and their components are tied by a unified probability structure with a single optimization step. On the other hand, the two steps in our approach are not entirely independent in the sense that the second step is performed on counties with occurrence probabilities >0.5 predicted by the first step.

The model-fitted incidence for each county was calculated as the following: if the step-1 logistic-model predicted a probability of presence less than the cut-off value, then the predicted incidence was set to 0; otherwise, the predicted incidence was set to the mean incidence predicted by the step-2 gamma model multiplied by the predicted probability of presence by the step-1 logistic model. Considering that hantavirus can also be detected by serological methods, we performed a sensitivity analysis of HFRS by including counties with only serological detection in small mammals (no PCR- or isolation-confirmed detection or human HFRS cases) as presence counties.

Appendix Text S5: The integrated database and distribution of small mammal species

After pooling the data from all sources (literature review, database searching, CISDCP, and surveillance reports), we obtained an integrated database of 14 484 records of small mammal species records. The final dataset part of small mammal contained 364 small mammals and shrews in 4 orders, 18 families, 114 genera and 364 species. For small mammal genus, we assembled 12 926, 1052, 480, and 26 records for Rodentia, Eulipotyphla, Lagomorpha, and Scandentia order, respectively. Supplementary data 1−2 shows the numbers of province, city, county level used in our study. Among them, Muridae family (7 099 records, present in 1 503 counties), Cricetidae family (2 089 records, present in 823 counties), Sciuridae family (1 955 records, present in 801 counties), Soricidae family (791 records, present in 304 counties), Pteromyidae family (666 records, present in 307 counties), and Spalacidae family (639 records, present in 408 counties) were the most widespread small mammal families, and *Rattus* genus (2 526 records, present in 1 304 counties), *Apodemus* genus (1 419 records, present in 900 counties), *Mus* genus (1 327 records, present in 1 178 counties), *Niviventer* genus (900 records, present in 508 counties), and *Cricetulus* genus (680 records, present in 515 counties) were the most widely distributed genus.

At the species level, the house mouse (*Mus musculus*), the brown rat (*Rattus norvegicus*), the striped field mouse (*Apodemus agrarius*), and the tanezumi rat (*Rattus tanezumi*) were recorded in >500 counties, and the white-bellied rat (*Niviventer niviventer*), the chinese striped hamster (*Cricetulus barabensi*)*s*, and the himalayan field rat (*Rattus nitidus*) were detected in 300–500 counties. The abundance of small mammal species varied markedly across seven biogeographic zones which differed in climatical and ecological conditions, notably with higher abundance in adjacent areas of neighboring biogeographic zones.

In general, high diversity of small mammal species was noticed in Southwestern China (hosting 166 species in total), Northwestern China (126 species), and Northern China (63 species). Higher species abundance of the families of Muridae and Sciuridae were shown in southern and eastern China provinces than those in western or northern China, and Cricetidae was shown a reversed pattern. Four provinces reported >100 small mammal species, including Yunnan (162 species), Sichuan (152 species), Gansu (122 species), and Shaanxi (106 species). Eight prefectures reported >40 small mammal species, four in the Sichuan province of southwestern China, three in the Yunnan province of southwestern China, and one in the Gansu province of northwestern. Higher species abundance of the families of Muridae and Sciuridae were shown in southern and eastern China provinces than those in western or northern China, and Cricetidae was shown a reversed pattern (appendix 2 figure S2−5, supplementary data S1−2).

Appendix Text S6: The distribution of small mammal-associated microbes (SMAMs) detected from small mammals in China

After pooling the data from all sources (literature review, database searching, CISDCP, and surveillance reports), we obtained an integrated database of 92 203 records of SMAMs (small mammal-association agents and rodent-borne diseases) records. We assembled 8910 and 83 293 records of small mammal-association agents, and rodent-borne diseases respectively.

155 small mammal-associated microbes (SMAM) were detected in small mammals, and *Yersinia pestis* were found inhabiting all four types of small mammals (figure 1C). We georeferenced and mapped locations of positive detections of 55 families of SMAMs in China, including 21 families of viruses, 14 families of bacteria, 18 families of parasites, and two families of fungus.

A total of 21 species in the Hantaviridae were detected from small mammals, the Hantaviridae viruses were widely distributed in almost all provinces except for Qinghai provinces at the county level (appendix 3 figure S13A). At the county level, Wenzhou mammarenavirus were detected in Xinjiang, Yunnan, and Guangdong province. The distributions of other virus agent species were mostly focal, for example, Arenavirus was found in the county of Inner Mongolia Autonomous Region, and Jingmen tick virus was detected in the small mammal from Xinjiang province (appendix 3 figure S13).

Altogether, the distributions of agent species were mostly focal, 71 species of bacteria were detected from small mammals, with Bartonellaceae being the most common species (21 species) detected from small mammals in north, Inner Mongolia–Xinjiang district, southwest, and southeast China, followed by Rickettsiaceae (11 species) mainly distributed in northwestern, southwest, and south China. In addition, the other species of agent were detected as relatively scattered, of which *Yersinia pestis* was distributed in plague focus (appendix3 figure S14−15).

Appendix Text S7: The distribution of rodent-borne diseases (RBDs) in China

We mapped the distributions of eight RBDs that were prevalent in China, including plague caused by *Yersinia pestis*, rat-bite fever caused by *Streptobacillus moniliformis* or *Spirillum minus*, tularemia caused by *Francisella tularensis*, *capillariasis* caused by *Capillaria hepatica*, lymphocytic choriomeningitis (LCM) caused by lymphocytic choriomeningitis virus, hymenolepiasis caused by *Hymenolepis*, leptospirosis caused by *leptospira* and HFRS caused by hantaviruses.

A total of 54 human plague cases were reported during the period from 2004 to 2020, which were in Qinghai (16 cases), Tibet (13 cases), Gansu (nine cases), Inner Mongolia (eight cases), Yunnan (seven cases) and Sichuan (one case), while small mammal infection with *Yersinia pestis* was distributed more widely than that in humans (figure 4A).

Totally 75 human cases were infected with rat-bite fever (70 with S*pirillum minus* and five with *Streptobacilus moniliformis*) and most of them were in southeast China (figure 4B).

Since the first human case of tularemia was report in Heilongjiang Province in 1959, altogether 72 tularemia cases have been reported from 1959 to 2021, who were mainly located in Shandong, Qinghai, and Heilongjiang provinces, while small mammal microbes were reported in provinces of northern China (figure 4C).

From 1950 to 2021, only seven capillariasis cases were reported in Ningxia, Guangdong, Sichuan, and Hainan provinces, while small mammal infection with *Capillaria hepatica* was distributed more widely (figure 4D).

In addition, 37 LCM cases were reported and most of them (24/37) were in Hebei province, while small mammal infections were only reported in Zhejiang province (figure 4E).

By the end of 2021, a total of 1363 human hymenolepsis cases were recorded who were located broadly across China and were mainly distributed in Hainan province and Xinjiang Autonomous Region (figure 4F).

A total of 9 582 leptospirosis cases were reported, mainly from southern and central China and A total of 204 601 HFRS cases were reported, mainly identified in the 98−135 degrees east longitude of China. The majority of HFRS cases were diagnosed in the northeastern (Heilongjiang, Jilin, Liaoning), north (Hebei), northwest (Shaanxi), central China (Hunan, Jiangxi, Henan, Hubei and Anhui) and Shandong, Fujian and Guangdong provinces along the eastern coast.

Appendix Text References

1. Sayers EW, Beck J, Bolton EE, et al. Database resources of the National Center for Biotechnology Information. *Nucleic Acids Res* 2021; **49**(D1): D10-d7.
2. O'Donnell MS, Ignizio DAJUgsds. Bioclimatic predictors for supporting ecological applications in the conterminous United States. 2012; **691**(10): 4-9.
3. Miao D, Liu MJ, Wang YX, et al. Epidemiology and Ecology of Severe Fever With Thrombocytopenia Syndrome in China, 2010‒2018. *Clin Infect Dis* 2021; **73**(11): e3851-e8.
4. Wang T, Meng F, Che T, et al. Mapping the distributions of blood-sucking mites and mite-borne agents in China: a modeling study. *Infect Dis Poverty* 2022; **11**(1): 41.
5. Wang T, Fan ZW, Ji Y, et al. Mapping the Distributions of Mosquitoes and Mosquito-Borne Arboviruses in China. *Viruses* 2022; **14**(4).
6. Fang LQ, de Vlas SJ, Liang S, et al. Environmental factors contributing to the spread of H5N1 avian influenza in mainland China. *PLoS One* 2008; **3**(5): e2268.
7. Fang LQ, Li XL, Liu K, et al. Mapping spread and risk of avian influenza A (H7N9) in China. *Sci Rep* 2013; **3**: 2722.
8. Dallas T, Park AW, Drake JM. Predictability of helminth parasite host range using information on geography, host traits and parasite community structure. *Parasitology* 2017; **144**(2): 200-5.
9. Martin V, Pfeiffer DU, Zhou X, et al. Spatial distribution and risk factors of highly pathogenic avian influenza (HPAI) H5N1 in China. *PLoS Pathog* 2011; **7**(3): e1001308.
10. Tarantola A, Blanchi S, Cappelle J, et al. Rabies Postexposure Prophylaxis Noncompletion After Dog Bites: Estimating the Unseen to Meet the Needs of the Underserved. *Am J Epidemiol* 2018; **187**(2): 306-15.
11. Elith J, Leathwick JR, Hastie T. A working guide to boosted regression trees. *J Anim Ecol* 2008; **77**(4): 802-13.
12. Zhang YY, Sun YQ, Chen JJ, et al. Mapping the global distribution of spotted fever group rickettsiae: a systematic review with modelling analysis. *Lancet Digit Health* 2023; **5**(1): e5-e15.
13. Zhao GP, Wang YX, Fan ZW, et al. Mapping ticks and tick-borne pathogens in China. *Nat Commun* 2021; **12**(1): 1075.
14. Hamilton LC. Statistics with Stata: version 12: Cengage Learning; 2012.
15. Chen T, Guestrin C. Xgboost: A scalable tree boosting system. Proceedings of the 22nd acm sigkdd international conference on knowledge discovery and data mining; 2016; 2016. p. 785-94.

Appendix Table S1: The laboratory tests used to detect small mammal-associated microbes (SMAMs) in the reviewed studies.

Infection types	Detection methods
**Infection in small mammals**	(1) Molecular detection with polymerase chain reaction (PCR) or next generation sequencing (NGS);<br>(2) Isolation and cultivation of pathogens from samples;<br>(3) Light or electronic micrograph identification.
**Infection in humans**	(1) Molecular detection with PCR or sequencing;<br>(2) Molecular detection and sequence determination;<br>(3) Isolation and cultivation of pathogens from samples;<br>(4) Light or electronic micrograph identification;<br>(5) A four-fold increase in titer of specific antibodies in blood sera collected from the acute and convalescent stages of illness, or a seroconversion of specific antibodies.

Appendix Table S2: Information of data sources in this study.

Variable	Source	Note	Data period
Climate data	http://data.cma.cn/data/cdcdetail/dataCode/SURF_CLI_CHN_MUL_MMON_19812010.html	The climate data are accessible in this website from 1981 to 2010. Data from 2010 to 2018 was accessible in past yet not open now, which can be accessible by contact the data source organization.	1981–2018
Land cover	https://www.resdc.cn/Datalist1.aspx?FieldTyepID=1,3	A raster digital map with a resolution of 1km.	1985–2015
Population density and proportion of older and female	http://www.stats.gov.cn/tjzs/tjsj/tjcb/tjzl/201303/t20130318_44794.html	The number of populations, older and female at the county level.	2010

Appendix Table S3: Potential risk factors for small mammal species, hemorrhagic fever with renal syndrome (HFRS) and leptospirosis. Potential risk factors at the county level used in the BRT model for small mammal species and two-step GBRT model for HFRS and Leptospirosis.

Category	Variables	Description (Unit)	Small mammal	HFRS	Leptospirosis
Environmental	Paddy field	Percent coverage of paddy field (%)	√	√	√
	Rainfed cropland	Percent coverage of rainfed cropland (%)	√	√	√
	Forest	Percent coverage of forest (%)	√	√	√
	Spinney	Percent coverage of spinney (%)	√	√	√
	Open woodland	Percent coverage of open woodland (%)	√	√	√
	Other woodland	Percent coverage of open other woodland (orchard, mulberry garden, tea garden, etc.) (%)	√	√	√
	High-density grasslands	Percent coverage of high-density grasslands (>50% area covered by grass) (%)	√	√	√
	Moderate-density grasslands	Percent coverage of moderate-density grasslands (50%> area covered by grass >20%) (%)	√	√	√
	Low-density grasslands	Percent coverage of low-density grasslands (20%> area covered by grass >5%) (%)	√	√	√
	River	Percent coverage of river (%)	√	√	√
	Lake	Percent coverage of lake (%)	√	√	√
	Reservoir	Percent coverage of reservoir (%)	√	√	√
	Permanent glacial snow	Percent coverage of permanent glacial snow (%)	√	√	√
	Mud flat	Percent coverage of permanent mud flat (%)	√	√	√
	Shoaly land	Percent coverage of permanent shoaly land (%)	√	√	√
	Rural residential land	Percent coverage of permanent rural residential land (%)	√	√	√
	Other construction land	Percent coverage of permanent other construction land (roads, mines, airports, etc.) (%)	√	√	√
	Sand	Percent coverage of permanent sand (%)	√	√	√
	Gobi	Percent coverage of permanent gobi (%)	√	√	√
	Saline and alkaline land	Percent coverage of permanent saline and alkaline land (%)	√	√	√
	Marsh land	Percent coverage of permanent marsh land (%)	√	√	√
	Bare land	Percent coverage of permanent bare land (%)	√	√	√
	Bare exposed rock or gravel	Percent coverage of permanent bare exposed rock or gravel (%)	√	√	√
	Elevation	Average elevation (m)		√	√
Social	Proportion of female	Proportion of women population (%)		√	√

	Proportion of the old	Proportion of ≥60 years old (%)		√	√
	Specialized hospital	Number of specialized hospitals		√	√
	General hospital	Number of general hospitals		√	√
	Health center	Number of health centers		√	√
	Clinic hospitals	Number of clinics hospitals		√	√
	Emergency center	Number of emergency center		√	√
	Population density	Human population density (persons per km^2)		√	√
	GDP	Gross domestic product (CNY)		√	√
Bioclimatic	Bio1	Annual mean temperature (℃)	√	√	√
	Bio2	Mean diurnal range (Mean of monthly (max temp-min temp)) (℃)	√	√	√
	Bio3	Isothermality (BIO02/BIO07) (*100)	√	√	√
	Bio4	Temperature seasonality (standard deviation*100)	√	√	√
	Bio5	Max temperature of warmest month (℃)	√	√	√
	Bio6	Min temperature of coldest month (℃)			
	Bio7	Annual range of temperature (BIO05-BIO06) (℃)			
	Bio8	Mean temperature of wettest quarter (℃)			
	Bio9	Mean temperature of driest quarter (℃)			
	Bio10	Mean temperature of warmest quarter (℃)			
	Bio11	Mean temperature of coldest quarter (℃)			
	Bio12	Annual precipitation (mm)	√	√	√
	Bio13	Precipitation of wettest month (mm)			
	Bio14	Precipitation of driest month (mm)			
	Bio15	Precipitation seasonality (coefficient of variation)	√	√	√
	Bio16	Precipitation of wettest quarter (mm)			
	Bio17	Precipitation of driest quarter (mm)	√	√	√
	Bio18	Precipitation of warmest quarter (mm)			
	Bio19	Precipitation of coldest quarter (mm)			
Biological	*Apodemus peninsulae*	Model-predicted probability of *Apodemus peninsulae* presence		√	

Tscherskia triton	Model-predicted probability of *Tscherskia triton* presence	√	
Rattus losea	Model-predicted probability of *Rattus losea* presence	√	√
Rattus tanezumi	Model-predicted probability of *Rattus tanezumi* presence	√	√
Mus musculus	Model-predicted probability of *Mus musculus* presence	√	
Apodemus agrarius	Model-predicted probability of *Apodemus agrarius* presence	√	√
Rattus norvegicus	Model-predicted probability of *Rattus norvegicus* presence	√	√

Appendix Table S4: The list of small mammal-associated microbes and corresponding references.

NO.	Agent type	Agent family	Agent genus	Agent species	Microbe types	References
1	Virus	Adenoviridae	Mastadenovirus	Adenovirus	Zoonotic pathogens	1-4
2	Virus	Anelloviridae	Alphatorquevirus	Torque teno virus	Zoonotic pathogens	4-7
3	Virus	Anelloviridae		Anellovirus	Zoonotic pathogens	8-10
4	Virus	Arenaviridae	Mammarenavirus	Wenzhou mammarenavirus	Zoonotic pathogens	11-22
5	Virus	Arenaviridae		Arenavirus	Zoonotic potential	14,17
6	Virus	Astroviridae	Avastrovirus	Xinjiang avastrovirus	Zoonotic pathogens	23
7	Virus	Astroviridae	Mamastrovirus	Mamastrovirus	Zoonotic potential	1
8	Virus	Astroviridae		Astrovirus	Zoonotic potential	24-27
9	Virus	Caliciviridae	Norovirus	Norovirus	Zoonotic potential	28
10	Virus	Coronaviridae	Betacoronavirus	Betacoronavirus	Zoonotic potential	29,30
11	Virus	Coronaviridae	Betacoronavirus	China Rattus coronavirus HKU24	Zoonotic potential	31
12	Virus	Coronaviridae	Deltacoronavirus	Deltacoronavirus	Zoonotic potential	30
13	Virus	Coronaviridae		Coronavirus	Zoonotic potential	32-34
14	Virus	Flaviviridae	Flavivirus	Japanese Encephalitis Virus	Zoonotic potential	35
15	Virus	Flaviviridae	Flavivirus	Tick-borne encephalitis virus	Zoonotic pathogens	36-44
16	Virus	Flaviviridae	Pegivirus	Pegivirus	Zoonotic potential	4,45,46
17	Virus	Flaviviridae		Jingmen tick virus	Zoonotic pathogens	47-49
18	Virus	Hantaviridae	Orthohantavirus	Amur virus	Zoonotic pathogens	50-56
19	Virus	Hantaviridae	Orthohantavirus	Cao Bang orthohantavirus	Zoonotic potential	57-60
20	Virus	Hantaviridae	Orthohantavirus	Dabieshan orthohantavirus	Zoonotic pathogens	61-69
21	Virus	Hantaviridae	Orthohantavirus	Fugong orthohantavirus	Zoonotic potential	70-73
22	Virus	Hantaviridae	Orthohantavirus	Gou virus	Zoonotic potential	74
23	Virus	Hantaviridae	Orthohantavirus	Hantaan orthohantavirus	Zoonotic pathogens	51,61,65,66,68,72,74-261262-501
24	Virus	Hantaviridae	Orthohantavirus	Kenkeme orthohantavirus	Zoonotic potential	77
25	Virus	Hantaviridae	Orthohantavirus	Khabarovsk orthohantavirus	Zoonotic potential	72,73,77,142,502
26	Virus	Hantaviridae	Orthohantavirus	Lianghe virus	Zoonotic potential	59,503,504
27	Virus	Hantaviridae	Orthohantavirus	Luxi virus	Zoonotic potential	73,505-507
28	Virus	Hantaviridae	Orthohantavirus	Puumala orthohantavirus	Zoonotic potential	100,173,508-546
29	Virus	Hantaviridae	Orthohantavirus	Qian Hu Shan virus	Zoonotic potential	58,547
30	Virus	Hantaviridae	Orthohantavirus	Seoul orthohantavirus	Zoonotic pathogens	53,65,66,72,80,82,85,86,91,92,94,95,100-102,106,107,114,116-119,124-126,129,132-134,136,141,143,150,151,153,154,166,170,195,197,267,507,546,548-687262-294,298-404,406-409,688-716410-500,717-7195,14,51,56,59,62,66,68,70,73-75,77,78,80,82,83,85-87,91,93-95,98,101,103,105,106,118,135,141-143,150,154,158,166,170,171,202,503,505,507,542,547,558,559,567,569,583,584,592,593,617-623,625,629,631-

						633,635-640,643,645-650,720-814
31	Virus	Hantaviridae	Orthohantavirus	Topografov virus	Zoonotic potential	72,73
32	Virus	Hantaviridae	Orthohantavirus	Tula orthohantavirus	Zoonotic potential	106,506,805,811,815-822
33	Virus	Hantaviridae	Orthohantavirus	Xinyi virus	Zoonotic potential	57
34	Virus	Hantaviridae	Orthohantavirus	Yakeshi orthohantavirus	Zoonotic potential	59,823
35	Virus	Hantaviridae	Orthohantavirus	Yuanjiang virus	Zoonotic potential	72
36	Virus	Hantaviridae	Thottimvirus	Imjin thottimvirus	Zoonotic pathogens	91,771,824-828
37	Virus	Hantaviridae	Thottimvirus	Thottapalayam thottimvirus	Zoonotic potential	547,824,829-832
38	Virus	Hepadnaviridae	Orthohepadnavirus	Hepatitis B virus	Zoonotic pathogens	833-835
39	Virus	Hepadnaviridae		Hepadnavirus	Zoonotic potential	558,836-838
40	Virus	Hepeviridae	Paslahepevirus	Hepatitis E Virus	Zoonotic pathogens	558,837,839-847
41	Virus	Herpesviridae		Herpesvirus	Zoonotic potential	4,846
42	Virus	Nairoviridae	Orthonairovirus	Crimean-Congo hemorrhagic fever orthonairovirus	Zoonotic pathogens	848-851
43	Virus	Orthomyxoviridae	Alphainfluenzavirus	Influenza A virus	Zoonotic pathogens	4
44	Virus	Papillomaviridae		Papillomaviruses	Zoonotic potential	852
45	Virus	Paramyxoviridae	Henipavirus	Mojiang henipavirus	Zoonotic potential	853
46	Virus	Paramyxoviridae	Jeilongvirus	Beilong jeilongvirus	Zoonotic potential	623,854-857
47	Virus	Paramyxoviridae	Jeilongvirus	Tailam jeilongvirus	Zoonotic potential	623,858
48	Virus	Parvoviridae	Bocaparvovirus	Bocaparvovirus	#N/A	859-870
49	Virus	Peribunyaviridae	Orthobunyavirus	Akabane orthobunyavirus	Zoonotic potential	871
50	Virus	Phenuiviridae	Bandavirus	Severe fever with thrombocytopenia syndrome bunyavirus	Zoonotic pathogens	872-881
51	Virus	Picornaviridae	Cardiovirus	Cardiovirus	Zoonotic potential	882-884
52	Virus	Picornaviridae	Hunnivirus	Hunnivirus	Zoonotic potential	885-887
53	Virus	Picornaviridae	Kobuvirus	Kobuvirus	Zoonotic potential	887-889
54	Virus	Picornaviridae		Picornavirus	Zoonotic potential	41,890-892
55	Virus	Rhabdoviridae	Lyssavirus	Lyssavirus	Zoonotic potential	893-897
56	Virus	Sedoreoviridae	Rotavirus	Rotavirus	Zoonotic potential	898
57	Bacteria	Actinomycetaceae	Actinomyces	*Actinomyces gaoshouyii*	Zoonotic pathogens	899-904
58	Bacteria	Actinomycetaceae	Boudabousia	*Boudabousia liubingyangii*	Zoonotic potential	905-910
59	Bacteria	Anaplasmataceae	Anaplasma	*Anaplasma bovis*	Zoonotic pathogens	911-915
60	Bacteria	Anaplasmataceae	Anaplasma	*Anaplasma phagocytophilum*	Zoonotic pathogens	916-933
61	Bacteria	Anaplasmataceae	Ehrlichia	*Candidatus Neoehrlichia mikurensis*	Zoonotic pathogens	934-942
62	Bacteria	Anaplasmataceae	Ehrlichia	*Ehrlichia chaffeensis*	Zoonotic pathogens	38,943-961
63	Bacteria	Bartonellaceae	Bartonella	*Bartonella coopersplainsensis*	Zoonotic potential	962-976
64	Bacteria	Bartonellaceae	Bartonella	*Bartonella doshiae*	Zoonotic pathogens	962,977-984
65	Bacteria	Bartonellaceae	Bartonella	*Bartonella elizabethae*	Zoonotic potential	963,985-1004
66	Bacteria	Bartonellaceae	Bartonella	*Bartonella fuyuanensis*	Zoonotic potential	963,1005-1010
67	Bacteria	Bartonellaceae	Bartonella	*Bartonella grahamii*	Zoonotic potential	962,963,969,989,990,993,999,1000,1007,1008,1011-1019
68	Bacteria	Bartonellaceae	Bartonella	*Bartonella heixiaziensis*	Zoonotic potential	549,962,1009,1011,1015,1020-1027
69	Bacteria	Bartonellaceae	Bartonella	*Bartonella henselae*	Zoonotic pathogens	1028-1041
70	Bacteria	Bartonellaceae	Bartonella	*Bartonella jaculi*	Zoonotic potential	1012,1015,1020
71	Bacteria	Bartonellaceae	Bartonella	*Bartonella japonica*	Zoonotic potential	962,964,1000,1010,1011,1018,1020,1042-1048
72	Bacteria	Bartonellaceae	Bartonella	*Bartonella krasnovii*	Zoonotic potential	1015,1049,1050
73	Bacteria	Bartonellaceae	Bartonella	*Bartonella phoceensis*	Zoonotic potential	964,985,989,1008,1010,1015,1030,1051-1053

**74**	Bacteria	Bartonellaceae	Bartonella	*Bartonella queenslandensis*	Zoonotic pathogens	963,964,987,999,1000,1009,1010,1022,1026,1053-1067
**75**	Bacteria	Bartonellaceae	Bartonella	*Bartonella rattimassiliensis*	Zoonotic potential	964,1000,1009,1026,1051,1053,1056,1068
**76**	Bacteria	Bartonellaceae	Bartonella	*Bartonella rochalimae*	Zoonotic potential	987,1016,1018,1069,1070
**77**	Bacteria	Bartonellaceae	Bartonella	*Bartonella rudakovii*	Zoonotic potential	1016,1071,1072
**78**	Bacteria	Bartonellaceae	Bartonella	*Bartonella silvatica*	Zoonotic pathogens	989,993,999,1000,1006,1011,1016,1018,1073-1081
**79**	Bacteria	Bartonellaceae	Bartonella	*Bartonella taylorii*	Zoonotic potential	962,989,993,999,1000,1006,1007,1011,1013,1016,1046
**80**	Bacteria	Bartonellaceae	Bartonella	*Bartonella tribocorum*	Zoonotic pathogens	548,987,999,1009,1017,1046,1054-1056,1061,1075,1082-1089
**81**	Bacteria	Bartonellaceae	Bartonella	*Bartonella vinsonii*	Zoonotic potential	962,990,1012,1019,1071,1081,1089-1098
**82**	Bacteria	Bartonellaceae	Bartonella	*Bartonella washoeensis*	Zoonotic potential	962,1011,1012,1099,1100
**83**	Bacteria	Bartonellaceae	Bartonella	*Bartonella bacilliformis*	Zoonotic potential	1015,1023,1086
**84**	Bacteria	Borreliaceae	Borrelia	*Borrelia afzelii*	Zoonotic pathogens	1060,1101-1105
**85**	Bacteria	Borreliaceae	Borrelia	*Borrelia burgdorferi sensu lato*	Zoonotic pathogens	38,926,1103,1106-1177
**86**	Bacteria	Borreliaceae	Borrelia	*Borrelia caucasica*	Zoonotic potential	1178
**87**	Bacteria	Borreliaceae	Borrelia	*Borrelia garinii*	Zoonotic pathogens	926,1101-1105,1126,1127,1131,1179-1182
**88**	Bacteria	Borreliaceae	Borrelia	*Borrelia valaisiana*	Zoonotic pathogens	1106,1179,1183-1189
**89**	Bacteria	Corynebacteriaceae	Corynebacterium	*Corynebacterium lizhenjunii*	Zoonotic potential	1190
**90**	Bacteria	Coxiellaceae	Coxiella	*Coxiella burnetii*	Zoonotic pathogens	869,947,1191-1193
**91**	Bacteria	Enterobacteriaceae	Escherichia	*Escherichia marmotae*	Zoonotic potential	1194
**92**	Bacteria	Enterobacteriaceae	Salmonella	*Salmonella enterica*	Zoonotic potential	1195
**93**	Bacteria	Francisellaceae	Francisella	*Francisella tularensis*	Zoonotic pathogens	1196
**94**	Bacteria	Helicobacteraceae	Helicobacter	*Helicobacter pylori*	Zoonotic potential	1197
**95**	Bacteria	Helicobacteraceae	Helicobacter	*Helicobacter apodemus*	Zoonotic potential	1197
**96**	Bacteria	Helicobacteraceae	Helicobacter	*Helicobacter canadensis*	Zoonotic potential	1197
**97**	Bacteria	Helicobacteraceae	Helicobacter	*Helicobacter ganmani*	Zoonotic potential	1197
**98**	Bacteria	Helicobacteraceae	Helicobacter	*Helicobacter hepaticus*	Zoonotic potential	1197
**99**	Bacteria	Helicobacteraceae	Helicobacter	*Helicobacter himalayensis*	Zoonotic potential	1198
**100**	Bacteria	Helicobacteraceae	Helicobacter	*Helicobacter pametensis*	Zoonotic potential	1197
**101**	Bacteria	Helicobacteraceae	Helicobacter	*Helicobacter winghamensis*	Zoonotic potential	1197
**102**	Bacteria	Leptospiraceae	Leptospira	*Leptospira alexanderi*	Zoonotic potential	1199-1205
**103**	Bacteria	Leptospiraceae	Leptospira	*Leptospira borgpetersenii*	Zoonotic potential	1199-1201,1206-1211
**104**	Bacteria	Leptospiraceae	Leptospira	*Leptospira interrogans*	Zoonotic pathogens	548,549,1200,1201,1206,1208,1210,1212-1258
**105**	Bacteria	Leptospiraceae	Leptospira	*Leptospira kirschneri*	Zoonotic potential	1206,1230,1231,1259,1260
**106**	Bacteria	Leptospiraceae	Leptospira	*Leptospira noguchii*	Zoonotic potential	1201,1230,1259,1261-1264
**107**	Bacteria	Leptospiraceae	Leptospira	*Leptospira santarosai*	Zoonotic potential	1206
**108**	Bacteria	Leptospiraceae	Leptospira	*Leptospira weilii*	Zoonotic potential	1201
**109**	Bacteria	Leptospiraceae	Leptospira	*Leptospira wolffii*	Zoonotic potential	1201,1212
**110**	Bacteria	Listeriaceae	Listeria	*Listeria fleischmannii*	Zoonotic potential	1265
**111**	Bacteria	Listeriaceae	Listeria	*Listeria floridensis*	Zoonotic potential	1265

112	Bacteria	Listeriaceae	Listeria	*Listeria innocua*	Zoonotic potential	1265
113	Bacteria	Listeriaceae	Listeria	*Listeria ivanovii*	Zoonotic potential	1265
114	Bacteria	Listeriaceae	Listeria	*Listeria monocytogenes*	Zoonotic potential	1265
115	Bacteria	Rickettsiaceae	Orientia	*Orientia tsutsugamushi*	Zoonotic pathogens	297,932,1266-1277
116	Bacteria	Rickettsiaceae	Rickettsia	*Candidatus Rickettsia tarasevichiae*	Zoonotic pathogens	1212,1278-1288
117	Bacteria	Rickettsiaceae	Rickettsia	*Rickettsia akari*	Zoonotic pathogens	797,1289-1295
118	Bacteria	Rickettsiaceae	Rickettsia	*Rickettsia conorii*	Zoonotic pathogens	1287,1290,1291,1296-1307
119	Bacteria	Rickettsiaceae	Rickettsia	*Rickettsia felis*	Zoonotic pathogens	947,1033,1308-1311
120	Bacteria	Rickettsiaceae	Rickettsia	*Rickettsia prowazekii*	Zoonotic pathogens	861,869,1312-1318
121	Bacteria	Rickettsiaceae	Rickettsia	*Rickettsia rickettsii*	Zoonotic pathogens	1300,1305,1319-1326
122	Bacteria	Rickettsiaceae	Rickettsia	*Rickettsia sibirica*	Zoonotic pathogens	947,1037,1041,1279,1296,1297,1300,1302,1306,1327-1342
123	Bacteria	Rickettsiaceae	Rickettsia	*Rickettsia typhi*	Zoonotic pathogens	861,947,1040,1041,1314,1316,1343-1354
124	Bacteria	Rickettsiaceae	Rickettsia	*Spotted fever group rickettsiae*	Zoonotic pathogens	861,947,1105,1192,1296,1314,1355-1377
125	Bacteria	Streptococcaceae	Streptococcus	*Streptococcus respiraculi*	Zoonotic potential	1378-1381
126	Bacteria	Yersiniaceae	Yersinia	*Yersinia enterocolitica*	Zoonotic pathogens	1382-1385
127	Bacteria	Yersiniaceae	Yersinia	*Yersinia pestis*	Zoonotic pathogens	302,693,916,1060,1198,1254,1385-1608
128	Bacteria			*Listeria innocua*	Zoonotic potential	1265
129	Fungus	Debaryomycetaceae	Candida	*Candida albicans*	Zoonotic pathogens	1609
130	Fungus	Trichocomaceae	Talaromyces	*Talaromyces marneffei*	Zoonotic potential	1609-1616
131	Parasite	Angiostrongylidae	Angiostrongylus	*Angiostrongylus cantonensis*	Zoonotic pathogens	38,1617-1685
132	Parasite	Babesiidae	Babesia	*Babesia microti*	Zoonotic pathogens	38,921,1686-1739
133	Parasite	Capillariidae	Capillaria	*Capillaria hepatica*	Zoonotic pathogens	1621,1633,1740-1768
134	Parasite	Chabertiidae	Oesophagostomum	*Oesophagostomum*	Zoonotic potential	1769
135	Parasite	Cryptosporidiidae	Cryptosporidium	*Cryptosporidium parvum*	Zoonotic pathogens	1749,1770
136	Parasite	Cryptosporidiidae	Cryptosporidium	*Cryptosporidium ubiquitum*	Zoonotic pathogens	1771
137	Parasite	Davaineidae	Raillietina	*Raillietina celebensis*	Zoonotic potential	1740
138	Parasite	Diphyllobothriidae	Sparganum	*Sparganum Mansoni*	Zoonotic potential	1772
139	Parasite	Enterocytozoonidae	Enterocytozoon	*Enterocytozoon bieneusi*	Zoonotic potential	1771,1773-1775
140	Parasite	Heteroxynematidae	Cephaluris	*Cephaluris coloradensis*	Zoonotic potential	1769
141	Parasite	Hymenolepididae	Hymenolepis	*Hymenolepis microstoma*	Zoonotic pathogens	1776-1780
142	Parasite	Hymenolepididae	Hymenolepis	*Hymenolepis diminuta*	Zoonotic pathogens	1740,1775,1778,1780-1792
143	Parasite	Hymenolepididae	Rodentolepis	*Rodentolepis nana*	Zoonotic pathogens	1740,1769,1778,1780,1781,1783,1785-1787,1790,1793,1794
144	Parasite	Oxyuridae	Syphacia	*Syphacia muris*	Zoonotic potential	1795
145	Parasite	Sarcocystidae	Toxoplasma	*Toxoplasma gondii*	Zoonotic pathogens	1617,1625,1769,1796-1799
146	Parasite	Schistosomatidae	Schistosoma	*Schistosoma haematobium*	Zoonotic potential	1778
147	Parasite	Schistosomatidae	Schistosoma	*Schistosoma japonicum*	Zoonotic pathogens	1625,1800-1806
148	Parasite	Schistosomatidae	Schistosoma	*Schistosoma sinensium*	Zoonotic potential	1807,1808
149	Parasite	Taeniidae	Echinococcus	*Echinococcus multilocularis*	Zoonotic pathogens	1769,1809-1812
150	Parasite	Taeniidae	Echinococcus	*Echinococcus shiquicus*	Zoonotic pathogens	1769,1809,1813
151	Parasite	Taeniidae	Hydatigera	*Hydatigera taeniaeformis*	Zoonotic pathogens	1787,1814
152	Parasite	Trichinellidae	Trichinella	*Trichinella britovi*	Zoonotic pathogens	1633,1794,1795,1815-1818
153	Parasite	Trypanosomatidae	Leishmania	*Leishmania tarentolae*	Zoonotic pathogens	1819
154	Parasite	Trypanosomatidae	Leishmania	*Leishmania turanica*	Zoonotic pathogens	1820
155	Parasite	Trypanosomatidae	Trypanosoma	*Trypanosoma lewisi*	Zoonotic pathogens	1618,1821-1826

Appendix Table S5: Clustering analysis of eco-climatic predictors at the county level based on pairwise Pearson correlation coefficients.

	bio1*	bio2*	bio3*	bio4*	bio5*	bio6	bio7	bio8	bio9	bio10	bio11	bio12*	bio13	bio14	bio15*	bio16	bio17*	bio18	bio19
bio1						0·967			0·962		0·965								
bio2																			
bio3																			
bio4							0·967												
bio5								0·815		0·880									
bio6									0·965		0·992								
bio7																			
bio8										0·934									
bio9											0·975								
bio10																			
bio11																			
bio12													0·961			0·973		0·893	
bio13																0·993		0·950	
bio14																	0·970		0·924
bio15																			
bio16																		0·955	
bio17																			0·981
bio18																			
bio19																			

Pairwise correlations above 0·8 are shown, and blank off-diagonal cells all have correlations < 0·8. Predictors grouped to the same cluster are colored the same. From each cluster, only one predictor (marked with *) is chosen to be used in county-level BRT models to avoid multicollinearity.

Appendix Table S6: BRT-model-estimated relative contributions to the spatial distribution of Cricetidae family. BRT-model-estimated mean (standard deviation) relative contributions of top factors (RC≥5%) to the spatial distribution of eight most prevalent small mammal species in the Cricetidae family.

Category	Variable	*Cricetulus migratorius*	*Craseomys rufocanus*	*Tscherskia triton*	*Cricetulus longicaudatus*	*Eothenomys melanogaster*	*Alexandromys fortis*	*Cricetulus barabensis*	*Eothenomys miletus*
Bioclimatic	Annual mean temperature		9·10 (1·58)	9·71 (1·18)				9·29 (1·58)	
	Mean diurnal range				5·65 (1·47)	10·22 (2·30)			
	Isothermality					5·67 (1·48)	6·35 (2·02)		34·18 (3·98)
	Temperature seasonality		8·37 (1·49)	13·09 (1·20)	14·46 (1·87)	13·08 (1·64)	16·58 (2·03)	30·91 (3·98)	8·38 (3·28)
	Total precipitation	19·80 (5·86)			6·70 (1·16)	6·77 (1·62)	9·53 (2·92)		
	Precipitation seasonality		11·52 (2·35)					22·04 (4·21)	
	Precipitation of driest quarter						5·55 (0·97)		
Environmental	Paddy field					8·15 (1·48)	5·77 (1·58)		
	Rainfed cropland			5·80 (0·82)					
	Forest	5·83 (2·75)	15·03 (2·10)						8·48 (1·64)
	Spinney		9·79 (2·20)	6·17 (1·08)	6·36 (1·32)				
	Open woodland								6·29 (1·55)
	Other woodland		8·01 (2·43)						
	High-density grasslands					5·18 (1·26)	11·25 (2·40)		5·43 (2·11)
	Low-density grasslands				8·09 (2·23)				
	Rural residential land								9·21 (2·66)
	Gobi	13·44 (6·58)							
	Marsh land						5·67 (1·40)		
	Bare land	5·10 (1·95)							
	Elevation				12·52 (2·05)	5·28 (0·92)		8·42 (1·48)	9·19 (1·86)
Social	Population density							5·07 (2·06)	
	GDP							5·16 (1·70)	
AUC	Train	0·991 (0·982, 0·998)	0·997 (0·992, 0·999)	0·994 (0·985, 0·999)	0·998 (0·995, 1·000)	0·998 (0·994, 1·000)	0·985 (0·972, 0·996)	0·996 (0·991, 0·999)	0·987 (0·978, 0·997)

	Test	0·925 (0·862, 0·975)	0·925 (0·862, 0·975)	0·874 (0·833, 0·904)	0·924 (0·889, 0·951)	0·910 (0·863, 0·959)	0·877 (0·807, 0·937)	0·957 (0·934, 0·973)	0·930 (0·875, 0·974)
Partial AUC Ratio	Train	1·94	1·93	1·80	1·89	1·92	1·91	1·68	1·93
	Test	1·89	1·92	1·78	1·87	1·91	1·82	1·67	1·87

Mean AUCs (95% percentiles) and partial area AUC ratio (calculated at tolerance level of 0.2) are given.

Appendix Table S7: BRT-model-estimated relative contributions to the spatial distribution of Muridae family. BRT-model-estimated mean (standard deviation) relative contributions of top factors (RC≥5%) to the spatial distribution of ten most prevalent small mammal species in the Muridae family.

Category	Variable	*Rattus rattus*	*Berylmys bowersi*	*Rattus norvegicus*	*Nniviventer fulvescens*	*Apodemus chevrieri*	*Niviventer confucianus*	*Leopoldamys edwardsi*	*Micromys minutus*	*Bandicota indica*	*Rattus nitidus*
Bioclimatic	Annual mean temperature	23·64 (5·99)	10·23 (2·69)	16·08 (1·96)	5·45 (1·19)		5·38 (2·12)			37·72 (5·39)	
	Isothermality	6·01 (1·58)	5·57 (1·75)	6·22 (0·95)		10·36 (2·83)				7·35 (1·74)	5·09 (1·02)
	Temperature seasonality	12·43 (2·87)	8·16 (1·64)	9·26 (1·03)	20·23 (1·64)	10·38 (2·56)	6·26 (2·65)	24·76 (2·23)	6·57 (1·34)	18·15 (3·87)	33·67 (1·62)
	Max temperature of warmest month										5·25 (1·22)
	Total precipitation			8·31 (1·71)		7·73 (2·18)				8·02 (1·70)	5·34 (0·92)
	Precipitation seasonality		6·08 (1·47)								
	Precipitation of driest quarter			7·63 (0·97)			7·27 (2·29)				9·76 (1·40)
Environ-mental	Paddy field					5·39 (1·70)	6·21 (3·25)		7·39 (1·35)		5·70 (1·09)
	Rainfed cropland								6·68 (1·39)		7·46 (1·62)
	Forest	5·66 (1·42)	13·47 (2·32)		15·60 (2·09)	5·26 (1·19)		9·90 (1·37)	6·82 (1·19)		
	Spinney		6·31 (1·30)		6·23 (1·09)		17·93 (2·82)				
	Open woodland					9·60 (2·44)					
	Other woodland	7·59 (2·12)		5·87 (0·85)						5·22 (1·80)	
	High-density grasslands			8·96 (1·46)							
	Moderate-density grasslands										5·06 (0·79)
	Other construction land					7·31 (1·39)					
	Elevation			7·45 (0·85)		18·16 (2·24)			11·83 (2·77)		7·58 (1·13)
Social	Population density		8·68 (1·90)						9·21 (1·73)	5·12 (1·57)	
AUC	Train	0·973 (0·933, 0·994)	0·995 (0·988, 0·999)	0·992 (0·990, 0·993)	0·984 (0·975, 0·993)	0·997 (0·994, 0·999)	0·963 (0·941, 0·983)	0·988 (0·979, 0·997)	0·984 (0·971, 0·995)	0·997 (0·994, 1·000)	0·993 (0·986, 0·997)
	Test	0·853 (0·790, 0·916)	0·908 (0·868, 0·947)	0·918 (0·897, 0·938)	0·875 (0·846, 0·904)	0·951 (0·924, 0·975)	0·798 (0·702, 0·883)	0·889 (0·857, 0·917)	0·822 (0·770, 0·873)	0·964 (0·940, 0·983)	0·931 (0·909, 0·950)
Partial AUC Ratio	Train	1·89	1·94	1·26	1·83	1·90	1·94	1·83	1·87	1·94	1·70

Test	1·81	1·93	1·25	1·78	1·90	1·85	1·78	1·86	1·94	1·66

Mean AUCs (95% percentiles) and partial area AUC ratio (calculated at tolerance level of 0.2) are given.

Appendix Table S8: BRT-model-estimated relative contributions to the spatial distribution of Muridae family. BRT-model-estimated mean (standard deviation) relative contributions of top factors (RC≥5%) to the spatial distribution of nine most prevalent small mammal species in the Muridae family.

Category	Variable	*Mus musculus*	*Apodemus peninsulae*	*Rattus losea*	*Meriones meridianus*	*Meriones unguiculatus*	*Rattus tanezumi*	*Apodemus draco*	*Apodemus agrarius*	*Nniviventer niviventer*
Bioclimatic	Annual mean temperature	18·99 (1·97)	8·21 (1·45)	6·45 (1·71)			9·84 (1·88)		15·60 (1·65)	5·41 (0·88)
	Mean diurnal range							7·93 (2·01)	5·62 (0·98)	
	Isothermality						6·13 (0·80)		9·95 (1·82)	
	Temperature seasonality	12·06 (1·77)	6·44 (1·06)				27·02 (2·02)		9·67 (2·07)	23·96 (1·41)
	Max temperature of warmest month									5·61 (0·90)
	Total precipitation		5·11 (1·04)	5·12 (1·34)	19·77 (5·19)	10·48 (2·78)	16·68 (2·55)		11·16 (1·71)	5·89 (0·90)
	Precipitation seasonality		7·13 (1·10)	9·74 (2·15)					5·85 (1·25)	5·20 (0·76)
	Precipitation of driest quarter	7·39 (0·79)	8·08 (1·18)	9·45 (2·31)		17·38 (3·30)	7·26 (0·89)	5·21 (1·27)		6·52 (0·83)
Environ-mental	Paddy field			6·37 (1·72)						
	Rainfed cropland				6·72 (1·67)			10·64 (2·19)	5·07 (1·88)	6·00 (1·00)
	Forest		11·92 (1·67)	10·71 (2·14)				10·98 (1·72)		6·64 (0·92)
	Spinney		10·40 (1·38)					9·64 (1·82)		6·22 (0·87)
	Open woodland					6·53 (1·60)	5·79 (0·72)			7·14 (0·80)
	High-density grasslands	8·26 (1·24)								
	Low-density grasslands			7·21 (1·65)	5·50 (1·90)					
	Rural residential land							5·97 (1·47)		
	Sand				22·88 (6·78)					
	Bare land				5·76 (1·73)	6·15 (2·15)				
	Elevation	6·05 (0·78)	5·17 (1·11)		7·47 (1·95)		5·97 (0·76)	9·56 (1·65)	15·55 (1·92)	5·34 (0·71)
Social	Population density	5·83 (1·63)				5·31 (1·76)				
AUC	Train	0·991 (0·989, 0·993)	0·980 (0·965, 0·991)	0·997 (0·989, 1·000)	0·995 (0·990, 0·999)	0·998 (0·995, 1·000)	0·991 (0·986, 0·994)	0·989 (0·979, 0·996)	0·989 (0·983, 0·992)	0·987 (0·974, 0·993)
	Test	0·911 (0·883, 0·937)	0·874 (0·828, 0·917)	0·903 (0·840, 0·948)	0·953 (0·927, 0·972)	0·956 (0·908, 0·985)	0·937 (0·917, 0·953)	0·884 (0·814, 0·935)	0·926 (0·903, 0·945)	0·893 (0·863, 0·923)

Partial AUC Ratio	Train	1·25	1·82	1·91	1·91	1·95	1·59	1·92	1·45	1·65
	Test	1·24	1·81	1·85	1·88	1·95	1·57	1·91	1·44	1·60

Mean AUCs (95% percentiles) and partial area AUC ratio (calculated at tolerance level of 0.2) are given.

Appendix Table S9: BRT-model-estimated relative contributions to the spatial distribution of Sciuridae family. BRT-model-estimated mean (standard deviation) relative contributions of top factors (RC≥5%) to the spatial distribution of seven most prevalent small mammal species in the Sciuridae family.

Category	Variable	*Spermophilus dauricus*	*Marmota himalayana*	*Callosciurus erythraeus*	*Sciurotamias davidianus*	*Dremomys pernyi*	*Tamias sibiricus*	*Tamiops swinhoei*
Bioclimatic	Annual mean temperature	5·58 (1·00)		7·54 (1·28)	10·81 (1·21)	9·05 (1·62)	14·75 (1·89)	6·36 (1·12)
	Mean diurnal range			5·60 (1·03)	6·54 (1·00)			5·18 (1·05)
	Isothermality				7·18 (0·94)	5·15 (1·40)		8·13 (1·63)
	Temperature seasonality	10·85 (1·59)		30·92 (2·92)	10·14 (1·16)	10·47 (2·10)	14·56 (1·36)	10·54 (2·21)
	Max temperature of warmest month				7·22 (1·12)			
	Total precipitation	10·66 (2·33)			6·26 (0·78)			6·18 (1·26)
	Precipitation seasonality	8·25 (2·20)			6·96 (1·13)			5·21 (0·96)
	Precipitation of driest quarter	8·66 (1·75)			6·24 (0·98)		9·41 (1·27)	
Environ-mental	Paddy field			5·68 (0·92)				
	Rainfed cropland	22·25 (2·38)					5·74 (0·68)	
	Forest		5·25 (1·44)				6·82 (1·06)	7·01 (1·27)
	Spinney			6·25 (1·23)	7·31 (1·18)	10·32 (1·68)	10·32 (1·33)	9·35 (1·74)
	Open woodland					5·12 (1·07)		
	High-density grasslands			5·38 (0·94)				7·01 (1·10)
	Moderate-density grasslands		7·61 (1·77)					8·16 (1·25)
	Marsh land		6·21 (2·09)					
	Elevation		44·01 (4·20)		9·20 (1·16)	7·13 (1·57)		6·10 (1·15)
Social	Population density		7·20 (2·19)			6·16 (1·69)		
	GDP		7·24 (2·41)					
AUC	Train	0·995 (0·989, 0·999)	0·999 (0·997, 1·000)	0·971 (0·953, 0·987)	0·997 (0·993, 0·999)	0·986 (0·976, 0·996)	0·995 (0·990, 0·999)	0·990 (0·981, 0·997)
	Test	0·931 (0·907, 0·955)	0·974 (0·948, 0·988)	0·871 (0·832, 0·912)	0·925 (0·897, 0·949)	0·878 (0·824, 0·922)	0·924 (0·893, 0·946)	0·913 (0·882, 0·941)
Partial AUC Ratio	Train	1·82	1·91	1·83	1·78	1·88	1·79	1·84
	Test	1·80	1·91	1·74	1·78	1·76	1·78	1·83

Mean AUCs (95% percentiles) and partial area AUC ratio (calculated at tolerance level of 0.2) are given.

Appendix Table S10: BRT-model-estimated relative contributions to the spatial distribution of other family. BRT-model-estimated mean (standard deviation) relative contributions of top factors (RC≥5%) to the spatial distribution of 11 most prevalent small mammal species in the other family.

Category	Variable	*Rhizomys sinensis*	*Eospalax fontanierii*	*Trogopterus xanthipes*	*Ochotona dauurica*	*Myospalax psilurus*	*Anourosorex squamipes*	*Ochotona thibetana*	*Petaurista alborufus*	*Pteromys volans*	*Orientallactaga sibirica*	*Crocidura suaveolens*
Bioclimatic	Annual mean temperature	5·14 (0·94)	6·93 (1·79)	6·84 (1·34)		13·57 (2·65)		5·33 (1·70)	5·84 (1·44)		9·55 (2·20)	11·12 (2·13)
	Mean diurnal range	8·77 (1·71)					5·86 (1·83)	10·64 (2·73)	6·40 (1·54)	5·60 (1·64)		
	Isothermality		10·74 (1·84)				9·68 (3·74)					5·99 (2·42)
	Temperature seasonality	13·17 (1·42)		10·69 (1·65)	6·30 (2·25)		14·48 (5·40)		13·80 (1·53)	7·06 (1·91)		22·52 (2·51)
	Max temperature of warmest month	5·53 (1·47)	6·05 (1·24)	6·34 (1·03)								
	Total precipitation		5·19 (1·42)	6·80 (1·29)			5·48 (2·23)	6·46 (2·14)	6·11 (1·33)		10·98 (3·11)	5·37 (1·82)
	Precipitation seasonality	6·35 (1·37)	9·51 (1·77)			7·11 (1·58)					5·64 (1·30)	5·22 (1·61)
	Precipitation of driest quarter		15·56 (2·16)	5·44 (1·16)		8·19 (1·69)		5·32 (1·83)			8·70 (1·55)	
Environ-mental	Rainfed cropland		10·22 (1·49)		12·53 (3·46)	8·66 (2·09)			6·35 (1·30)		6·83 (1·99)	
	Forest	5·06 (0·94)		5·19 (0·91)		5·17 (1·21)	6·06 (1·68)	8·82 (2·51)		14·66 (1·58)		
	Spinney			7·61 (1·15)			5·89 (1·47)	5·08 (1·26)	6·24 (1·35)			
	Open woodland	6·48 (1·42)		8·01 (0·93)								5·09 (1·76)
	Other woodland				6·91 (2·03)				7·24 (1·51)			
	High-density grasslands				6·65 (2·09)					8·27 (1·65)	5·17 (1·11)	33·21 (3·59)
	Moderate-density grasslands	5·39 (1·26)										
	Rural residential Land					5·18 (1·49)						
	Gobi										10·79 (2·79)	
	Marsh land					5·15 (2·20)					7·55 (1·35)	
	Bare land				6·91 (1·74)							
	Elevation		9·96 (1·77)	8·30 (1·34)	11·63 (1·41)		9·13 (2·87)	42·23 (2·97)	5·94 (1·63)			
Social	Population density	6·77 (1·26)								9·58 (1·66)		
	GDP				9·66 (2·21)		8·26 (2·16)					

AUC	Train	0·991 (0·981, 0·998)	0·999 (0·997, 1·000)	0·997 (0·993, 0·999)	0·998 (0·996, 1)	0·989 (0·977, 0·998)	0·981 (0·965, 0·996)	0·995 (0·989, 0·999)	0·998 (0·994, 1·000)	0·986 (0·974, 0·996)	0·993 (0·986, 0·999)	0·998 (0·995, 1·000)
	Test	0·902 (0·866, 0·937)	0·964 (0·944, 0·982)	0·918 (0·888, 0·951)	0·959 (0·923, 0·988)	0·882 (0·813, 0·934)	0·892 (0·820, 0·950)	0·952 (0·916, 0·980)	0·920 (0·882, 0·956)	0·859 (0·798, 0·916)	0·935 (0·909, 0·960)	0·929 (0·864, 0·979)
Partial AUC Ratio	Train	1·88	1·83	1·84	1·95	1·93	1·95	1·94	1·93	1·93	1·91	1·88
	Test	1·87	1·83	1·84	1·95	1·82	1·77	1·93	1·86	1·86	1·90	1·86

Mean AUCs (95% percentiles) and partial area AUC ratio (calculated at tolerance level of 0.2) are given.

Appendix Table S11: Relative contributions of major factors to the spatial distributions of hantavirus detected by PCR, isolation, and serological methods.

Category	Variable	hantavirus (relative contributions %) #	
		Step 1 (Presence)	Step 2 (Incidence Rate)
**Environmental**	Forest (%)	-	1·96 (0·59)
	Spinney (%)	-	2·06 (0·40)
	Open woodland (%)	-	3·58 (0·52)
	Other woodland (%)	-	2·50 (0·42)
	Moderate-density grasslands (%)	-	2·11 (0·46)
	Low-density grasslands (%)	4·48 (1·39)	5·33 (1·31)
	Rural residential land (%)	-	2·35 (0·49)
	Bare exposed rock or gravel (%)	48·21 (2·81)	-
	Elevation (m)	5·93 (2·11)	4·27 (0·74)
**Bioclimatic**	Annual mean temperature (℃)	3·24 (0·66)	19·51 (2·62)
	Mean diurnal range	-	4·37 (0·83)
	Isothermality	6·62 (2·07)	2·47 (0·37)
	Temperature seasonality	5·90 (1·12)	9·33 (2·73)
	Max temperature of warmest month (℃)	-	1·95 (0·35)
	Total precipitation (mm)	9·87 (1·61)	8·74 (1·64)
	Precipitation seasonality (mm)	5·11 (0·81)	2·92 (0·42)
	Precipitation of driest quarter (mm)	-	12·46 (1·29)
**Social**	Gross domestic product GDP (CNY)	3·42 (1·07)	3·18 (0·41)
	Population density (persons per km^2)	3·48 (0·81)	2·60 (0·33)
	Proportion of ≥60 years old	-	2·36 (0·37)
**Biological**	Presence of *Rattus tanezumi* &	-	2·74 (0·80)
	Presence of *Apodemus agrarius* &	3·75 (0·99)	3·21 (0·48)

&The presence of predominant small mammal species indicated the occurrence probability of each species predicted by model.

Appendix Table S12: The number of 45 species of small mammals in the counties of 1950–2000 and 2001–2021.

Species	1950-2021	1950-2000	1950-2000/ 1951-2021	2001-2021	2001-2021/ 1951-2021
Mus musculus	1169	1083	93%	831	71%
Rattus norvegicus	1161	1074	93%	774	67%
Apodemus agrarius	771	456	59%	406	53%
Rattus tanezumi	534	440	82%	114	21%
Niviventer niviventer	432	369	85%	148	34%
Cricetulus barabensis	393	181	46%	271	69%
Rattus nitidus	360	312	87%	92	26%
Sciurotamias davidianus	250	140	56%	120	48%
Tamias sibiricus	248	128	52%	167	67%
Tscherskia triton	232	136	59%	204	88%
Spermophilus dauricus	208	65	31%	189	91%
Apodemus peninsulae	200	78	39%	170	85%
Callosciurus erythraeus	192	119	62%	102	53%
Eospalax fontanierii	192	122	64%	77	40%
Niviventer fulvescens	185	127	69%	88	48%
Leopoldamys edwardsi	185	10	5%	11	6%
Tamiops swinhoei	184	111	60%	117	64%
Trogopterus xanthipes	182	70	38%	90	49%
Micromys minutus	146	102	70%	85	58%
Dremomys pernyi	131	80	61%	59	45%
Rhizomys sinensis	131	98	75%	58	44%
Crocidura suaveolens	128	114	89%	20	16%
Rattus rattus	120	85	71%	100	83%
Cricetulus longicaudatus	115	60	52%	88	77%
Apodemus chevrieri	110	56	51%	66	60%
Meriones meridianus	104	38	37%	87	84%
Marmota himalayana	100	84	84%	36	36%
Alexandromys fortis	98	81	83%	15	15%
Rattus losea	94	66	70%	26	28%
Orientallactaga sibirica	92	79	86%	12	13%
Eothenomys melanogaster	90	60	67%	53	59%
Apodemus draco	89	54	61%	28	31%
Pteromys volans	80	51	64%	65	81%
Craseomys rufocanus	79	39	49%	39	49%
Petaurista alborufus	78	48	62%	36	46%
Eothenomys miletus	75	44	59%	37	49%
Myospalax psilurus	69	57	83%	61	88%
Berylmys bowersi	67	52	78%	39	58%
Bandicota indica	63	50	79%	53	84%
Niviventer confucianus	62	11	18%	40	65%
Ochotona thibetana	61	37	61%	20	33%
Cricetulus migratorius	60	52	87%	7	12%
Anourosorex squamipes	55	13	24%	15	27%
Meriones unguiculatus	55	40	73%	45	82%
Ochotona dauurica	54	42	78%	13	24%

Appendix Table S13: Relative contributions of the top six predictors in the ecological models for *Mus musculus* and *Rattus norvegicus* based on data from 1950-2021 and 1950-2000.

1950-2021	*Mus musculus*	RC (%)
1	Annual Mean Temperature	18.99
2	Temperature Seasonality	12.06
3	High Coverage Grasslands	8.26
4	Precipitation of Driest Quarter	7.39
5	Elevation	6.05
6	Population Density	5.83
1950-2021	*Rattus norvegicus*	RC (%)
1	Annual Mean Temperature	16.08
2	Temperature Seasonality	9.26
3	High Coverage Grasslands	8.96
4	Total precipitation	8.31
5	Precipitation of Driest Quarter	7.63
6	Elevation	7.45

1950-2000	*Mus musculus*	RC (%)
1	Annual Mean Temperature	14.27
2	High Coverage Grasslands	13.39
3	Temperature Seasonality	11.67
4	Elevation	9.97
5	Isothermality	9.87
6	Precipitation Seasonality	6.89
1950-2000	*Rattus norvegicus*	RC (%)
1	High Coverage Grasslands	16.06
2	Annual Mean Temperature	10.74
3	Temperature Seasonality	9.87
4	Elevation	9.43
5	Total precipitation	7.31
6	Other Woodland	7.24

Appendix References

1. Ao YY, Yu JM, Li LL, et al. Diverse novel astroviruses identified in wild Himalayan marmots. *J Gen Virol* 2017; **98**(4): 612-23.
2. Xiong YQ, Zhang MY, Zhou JH, et al. A Molecular Epidemiological Investigation of Carriage of the Adeno-Associated Virus in Murine Rodents and House Shrews in China. *Intervirology* 2018; **61**(3): 143-8.
3. Zheng XY, Qiu M, Ke XM, et al. Detection of novel adenoviruses in fecal specimens from rodents and shrews in southern China. *Virus Genes* 2016; **52**(3): 417-21.
4. He W, Gao Y, Wen Y, et al. Detection of Virus-Related Sequences Associated With Potential Etiologies of Hepatitis in Liver Tissue Samples From Rats, Mice, Shrews, and Bats. *Front Microbiol* 2021; **12**: 653873.
5. Wu Y, Wang S, Chen Y, et al. Complete Genome Sequence of a Rodent Torque Teno Virus in Hainan Island, China. *Microbiol Resour Announc* 2018; **7**(19).
6. Xiong YQ, Mo Y, Chen MJ, Cai W, He WQ, Chen Q. Detection and phylogenetic analysis of torque teno virus (TTV) carried by murine rodents and house shrews in China. *Virology* 2018; **516**: 189-95.
7. Yongxin L, Xuhua B. Research progress on the prevalence and diversity of Torque Teno Virus. *Chinese Bulletin of Life Sciences* 2017; **29**(11): 5.
8. Du J, Li Y, Lu L, et al. Biodiversity of rodent anelloviruses in China. *Emerg Microbes Infect* 2018; **7**(1): 38.
9. Ning SY, Zhou MM, Yang J, Zeng J, Wang JP. Viral metagenomics reveals two novel anelloviruses in feces of experimental rats. *Virol J* 2021; **18**(1): 252.
10. Liu Y, Sun L, Tu Z, et al. Virome Profiling of an Amur leopard cat Reveals Multiple Anelloviruses and a Bocaparvovirus. *Vet Sci* 2022; **9**(11).
11. Li K, Lin X, Li M, Wang M, Sun X, Zhang YJ. Genomic analysis of Wenzhou virus in rodents from Zhejiang province. 2017; **38**(3): 384-7.
12. Zhang Y, Rao L, Lv G, et al. Complete Genome Sequence of a Mammarenavirus Harbored by Rodents on Hainan Island, China. *Genome Announc* 2018; **6**(10).
13. Liu MM, Li LL, Wang XF, Duan ZJ. Complete Genome Sequence of a Novel Variant of Wenzhou Mammarenavirus. *Genome Announc* 2017; **5**(47).
14. Wu Z, Du J, Lu L, et al. Detection of Hantaviruses and Arenaviruzses in three-toed jerboas from the Inner Mongolia Autonomous Region, China. *Emerg Microbes Infect* 2018; **7**(1): 35.
15. Blasdell KR, Duong V, Eloit M, et al. Evidence of human infection by a new mammarenavirus endemic to Southeastern Asia. *Elife* 2016; **5**.
16. Wu JY, Guo C, Xia Y, et al. Genomic characterization of Wenzhou mammarenavirus detected in wild rodents in Guangzhou City, China. *One Health* 2021; **13**: 100273.
17. Li K, Lin XD, Wang W, et al. Isolation and characterization of a novel arenavirus harbored by Rodents and Shrews in Zhejiang province, China. *Virology* 2015; **476**: 37-42.
18. Wang J, Yang X, Liu H, et al. Prevalence of Wēnzhōu virus in small mammals in Yunnan Province, China. *PLoS Negl Trop Dis* 2019; **13**(2): e0007049.
19. Guo L, Liu S, Song J, et al. Seroprevalence of Wenzhou virus in China. *Biosaf Health* 2020; **2**(3): 152-6.
20. Tan Z, Yu H, Xu L, et al. Virome profiling of rodents in Xinjiang Uygur Autonomous Region, China: Isolation and characterization of a new strain of Wenzhou virus. *Virology* 2019; **529**: 122-34.
21. Liu M-m, Li L-l, Wang X-f, Duan Z-jJGa. Complete genome sequence of a novel variant of Wenzhou Mammarenavirus. 2017; **5**(47): e01303-17.
22. Wang N, Yang L, Li G, et al. Molecular detection and genetic characterization of Wenzhou virus in rodents in Guangzhou, China. *BMC Vet Res* 2021; **17**(1): 301.
23. Long Zhimei LJ, Huang Jialiang, Yu Xinglong, Zhao Chunsheng, Jiang Kanghua, Lu Zhenfu, Yan Aiwu. A prelimiry study on the relationship between rat like animals and human diseases in the south chi sea. *Chin J Zoonoses* 1996; **12**(03): 0-.
24. To KKW, Chan WM, Li KSM, et al. High prevalence of four novel astrovirus genotype species identified from rodents in China. *J Gen Virol* 2017; **98**(5): 1004-15.
25. Hu B, Chmura AA, Li J, et al. Detection of diverse novel astroviruses from small mammals in China. *J Gen Virol* 2014; **95**(Pt 11): 2442-9.
26. Chu DK, Chin AW, Smith GJ, et al. Detection of novel astroviruses in urban brown rats and previously known astroviruses in humans. *J Gen Virol* 2010; **91**(Pt 10): 2457-62.
27. Min Q. Investigation and Gene Analysis of Asterovirus Carried by Rodents and Shrews in Guangzhou and Xiamen: Southern Medical University; 2017.
28. Tse H, Chan WM, Lam CS, Lau SK, Woo PC, Yuen KY. Complete genome sequences of novel rat noroviruses in Hong Kong. *J Virol* 2012; **86**(22): 12435-6.

29. Ge XY, Yang WH, Zhou JH, et al. Detection of alpha- and betacoronaviruses in rodents from Yunnan, China. *Virol J* 2017; **14**(1): 98.
30. Zhu W, Yang J, Lu S, et al. Beta- and Novel Delta-Coronaviruses Are Identified from Wild Animals in the Qinghai-Tibetan Plateau, China. *Virol Sin* 2021; **36**(3): 402-11.
31. Lau SK, Woo PC, Li KS, et al. Discovery of a novel coronavirus, China Rattus coronavirus HKU24, from Norway rats supports the murine origin of Betacoronavirus 1 and has implications for the ancestor of Betacoronavirus lineage A. *J Virol* 2015; **89**(6): 3076-92.
32. Wang W, Lin XD, Liao Y, et al. Discovery of a Highly Divergent Coronavirus in the Asian House Shrew from China Illuminates the Origin of the Alphacoronaviruses. *J Virol* 2017; **91**(17).
33. Wang W, Lin XD, Guo WP, et al. Discovery, diversity and evolution of novel coronaviruses sampled from rodents in China. *Virology* 2015; **474**: 19-27.
34. Wang W, Lin XD, Zhang HL, et al. Extensive genetic diversity and host range of rodent-borne coronaviruses. *Virus Evol* 2020; **6**(2): veaa078.
35. Chen SW, Jiang LN, Zhong XS, et al. Serological Prevalence Against Japanese Encephalitis Virus-Serocomplex Flaviviruses in Commensal and Field Rodents in South China. *Vector Borne Zoonotic Dis* 2016; **16**(12): 777-80.
36. Dai X, Shang G, Lu S, Yang J, Xu J. A new subtype of eastern tick-borne encephalitis virus discovered in Qinghai-Tibet Plateau, China. *Emerg Microbes Infect* 2018; **7**(1): 74.
37. Cai Zenglin LZ, Hu Lingmei, Zhao Zhanlin, Jin Xiantao, He Yixiang. Epidemiological investigation on tural foci of forest encephalitis in Hunchun area. *Public Health in China* 1995; **14**(1): 2.
38. Hu Manxia ZJ, Fan Donghui, Fu Weiming, Sun Xiufeng, Yang Liwei. Detection of rodent infected by new tick pathogens in Suifenhe and Dongning ports. *Chinese Jourl of Vector Biology and Control* 2009; **20**(6): 4.
39. Wang Zhuo WQ, Yu Miao, Zhang Qingbo, Guo Zunyuan, Feng Li, Wang Xiuhong, Wu Yimin. Serological investigation of several important tick borne pathogens in rodents in forest areas of Chi DPRK border areas. *J Microbiol* 2017; **37**(06): 41-4.
40. Zhan L, Cao WC, Chu CY, et al. Tick-borne agents in rodents, China, 2004–2006. 2009; **15**(12): 1904.
41. Shasha L. Investigation on Serological Prevalence of Wenzhou Virus: Peking Union Medical College.
42. Zhang GL, Liu R, Sun X, et al. [Investigation on the endemic foci of new emerged tick-borne encephalitis in Charles Hilary, Xinjiang]. *Zhonghua Liu Xing Bing Xue Za Zhi* 2013; **34**(5): 438-42.
43. Wang J, Zhang H, Fu S, et al. Isolation of kyasanur forest disease virus from febrile patient, yunnan, china. *Emerg Infect Dis* 2009; **15**(2): 326-8.
44. Chen LF, Liu YC, Chen SH, Hui S, Li JH, Xu J. [Characteristic analysis of E protein genes of new strains of tick-borne encephalitis virus isolated from China]. *Bing Du Xue Bao* 2008; **24**(3): 202-7.
45. Gao YW, Wan ZW, Wu Y, Li XF, Tang SX. PCR-based screening and phylogenetic analysis of rat pegivirus (RPgV) carried by rodents in China. *J Vet Med Sci* 2020; **82**(10): 1464-71.
46. Dujiang. Study on Viral Groups and Evolution of 7 Families of Rodents, including Sarviviridae and Xanthoviridae: Peking Union Medical College; 2017.
47. Qin XC, Shi M, Tian JH, et al. A tick-borne segmented RNA virus contains genome segments derived from unsegmented viral ancestors. *Proc Natl Acad Sci U S A* 2014; **111**(18): 6744-9.
48. Guo JJ, Lin XD, Chen YM, et al. Diversity and circulation of Jingmen tick virus in ticks and mammals. *Virus Evol* 2020; **6**(2): veaa051.
49. Yu ZM, Chen JT, Qin J, et al. Identification and characterization of Jingmen tick virus in rodents from Xinjiang, China. *Infect Genet Evol* 2020; **84**: 104411.
50. Guo Weidong ZW, Jiang Jiafu, Chi Haiyi, Wang Wenrui, Wang Zhongyuan, Jin Ying, Yang Hong, Fang Liqun, Cao Wuchun. Complete sequence of the M segment of Amur virus in rodent from China. *Chinese Jourl of Epidemiology* 2009; (10): 4.
51. Yao LS, Zhao H, Shao LJ, et al. Complete genome sequence of an amur virus isolated from Apodemus peninsulae in Northeastern China. *J Virol* 2012; **86**(24): 13816-7.
52. Wang Ying FZ, Li Ying, Gao Yanfei, Liu Yingying, Zhang Xiaolong, Cao Xiaomei. Investigation and gene characteristics of hantavirus in rats at Changbai port in 2019. *Chinese sanitary insecticide* 2020; **26**(6): 3.
53. Yao Lisi SL, Wang Gang. Amur like virus nucleic acid was detected in Apodemus agrarius in Changbai County, Jilin Province. *Jourl of Parasites and Medical Insects* 2013; (2): 5.
54. Zhang WY, Jiang JF, Yao K, et al. [Identification of Amur like virus in Apodemus peninsulae and its molecular characteristics in China]. *Zhonghua Liu Xing Bing Xue Za Zhi* 2007; **28**(5): 482-6.
55. Jiang JF, Zhang WY, Wu XM, Zhang PH, Cao WC. Soochong virus and Amur virus might be the same entities of hantavirus. *J Med Virol* 2007; **79**(11): 1792-5.

56. Guo WD, Zhang WY, Jiang JF, et al. [Complete sequence of the M segment of Amur virus in rodent from China]. *Zhonghua Liu Xing Bing Xue Za Zhi* 2009; **30**(10): 1051-4.
57. Gu SH, Arai S, Yu HT, Lim BK, Kang HJ, Yanagihara R. Genetic variants of Cao Bang hantavirus in the Chinese mole shrew (Anourosorex squamipes) and Taiwanese mole shrew (Anourosorex yamashinai). *Infect Genet Evol* 2016; **40**: 113-8.
58. Zuo SQ, Gong ZD, Fang LQ, et al. A new hantavirus from the stripe-backed shrew (Sorex cylindricauda) in the People's Republic of China. *Virus Res* 2014; **184**: 82-6.
59. Guo WP, Lin XD, Wang W, et al. Phylogeny and origins of hantaviruses harbored by bats, insectivores, and rodents. *PLoS Pathog* 2013; **9**(2): e1003159.
60. Lokugamage K, Kariwa H, Lokugamage N, et al. Genetic and antigenic characterization of the Amur virus associated with hemorrhagic fever with renal syndrome. *Virus Res* 2004; **101**(2): 127-34.
61. Li Hongbing FH, Hu Kan, Hu Xiaoqian, Deng Feng, Zhang Kejian, Gao Juan, Yan Chuanyuan, Tian Hui, Zhang Yaning. Survey on host animals of hemorrhagic fever with rel syndrome in Baoji City of Shaanxi Province from 2014 to 2018. *Chinese sanitary insecticide* 2020.
62. Cao ZW, Zuo SQ, Gong ZD, et al. Genetic analysis of a hantavirus strain carried by Niviventer confucianus in Yunnan province, China. *Virus Res* 2010; **153**(1): 157-60.
63. Zhiwei C. Hantavirus infection and gene variation in rodents in some areas of China: PLA Academy of Military Medical Sciences; 2010.
64. Liu Shiwen XG, Gong Tian, Shi Yong, Xiao Fang, Li Jianxiong, Zhang Yanni, Zhou Jun, Liu Xiaoqing, Xiong Ying. Detection and gene characteristics of Dabie Mountain virus in 537 rat lung specimens in Jiangxi Province. *Modern Preventive Medicine* 2018; **45**(20): 4.
65. Wang H, Yoshimatsu K, Ebihara H, et al. Genetic diversity of hantaviruses isolated in china and characterization of novel hantaviruses isolated from Niviventer confucianus and Rattus rattus. *Virology* 2000; **278**(2): 332-45.
66. Lin XD, Wang W, Guo WP, et al. Cross-species transmission in the speciation of the currently known murinae-associated hantaviruses. *J Virol* 2012; **86**(20): 11171-82.
67. Hu Qun ZC, Ma Sijie, Tong Shumei, Mei Yong. Comparison and alysis of S gene sequences of hantavirus strains isolated from 5 mouse samples. *Chinese Jourl of Zoonoses* 2016; **32**(5): 4.
68. Tian H, Tie WF, Li H, et al. Orthohantaviruses infections in humans and rodents in Baoji, China. *PLoS Negl Trop Dis* 2020; **14**(10): e0008778.
69. Luo Zhaozhuang LY, Liu Hong, Wang Yiyin, Zhao Yueping. Discovery of a new subtype of Hantavirus in a small epidemic focus of hemorrhagic fever with rel syndrome in mountain forest areas of Anhui Province. *Chinese Jourl of Epidemiology* 2002; **23**(5): 363-5.
70. Ge XY, Yang WH, Pan H, et al. Fugong virus, a novel hantavirus harbored by the small oriental vole (Eothenomys eleusis) in China. *Virol J* 2016; **13**: 27.
71. Li Wenjuan ZH, Zhang Yunzhi, Yang Weihong, Zhou Jihua, Duan Chunli, Zhang Qiong. Epidemiological characteristics of hemorrhagic fever with rel syndromein in Xiangyun County，Yunn Province. *Chinese Tropical Medicine* 2013; (11): 4.
72. Zou Y, Wang JB, Gaowa HS, et al. Isolation and genetic characterization of hantaviruses carried by Microtus voles in China. *J Med Virol* 2008; **80**(4): 680-8.
73. Zou Y, Xiao QY, Dong X, et al. Genetic analysis of hantaviruses carried by reed voles Microtus fortis in China. *Virus Res* 2008; **137**(1): 122-8.
74. Wang W, Wang MR, Lin XD, et al. Ongoing spillover of Hantaan and Gou hantaviruses from rodents is associated with hemorrhagic fever with renal syndrome (HFRS) in China. *PLoS Negl Trop Dis* 2013; **7**(10): e2484.
75. Li N, Li A, Liu Y, et al. Genetic diversity and evolution of Hantaan virus in China and its neighbors. *PLoS Negl Trop Dis* 2020; **14**(8): e0008090.
76. Jiang JF, Zhang WY, Yao K, et al. A new Hantaan-like virus in rodents (Apodemus peninsulae) from Northeastern China. *Virus Res* 2007; **130**(1-2): 292-5.
77. Wang CQ, Gao JH, Li M, et al. Co-circulation of Hantaan, Kenkeme, and Khabarovsk Hantaviruses in Bolshoy Ussuriysky Island, China. *Virus Res* 2014; **191**: 51-8.
78. Zou Y, Hu J, Wang ZX, et al. Molecular diversity and phylogeny of Hantaan virus in Guizhou, China: evidence for Guizhou as a radiation center of the present Hantaan virus. *J Gen Virol* 2008; **89**(Pt 8): 1987-97.
79. Yi J, Xu Z, Zhuang R, et al. Hantaan virus RNA load in patients having hemorrhagic fever with renal syndrome: correlation with disease severity. *J Infect Dis* 2013; **207**(9): 1457-61.
80. Zhang YZ, Zou Y, Fu ZF, Plyusnin A. Hantavirus infections in humans and animals, China. *Emerg Infect Dis* 2010; **16**(8): 1195-203.
81. Zhang X, Chen HY, Zhu LY, et al. Comparison of Hantaan and Seoul viral infections among patients with hemorrhagic fever with renal syndrome (HFRS) in Heilongjiang, China. *Scand J Infect Dis* 2011;

43(8): 632-41.
82. Shang C, Sun Y, Yin Q, et al. Hemorrhagic Fever with Renal Syndrome - Liaoning Province, China, 1999-2018. *China CDC Wkly* 2020; **2**(20): 350-4.
83. Li JL, Ling JX, Liu DY, et al. Genetic characterization of a new subtype of Hantaan virus isolated from a hemorrhagic fever with renal syndrome (HFRS) epidemic area in Hubei Province, China. *Arch Virol* 2012; **157**(10): 1981-7.
84. Song G. Epidemiological progresses of hemorrhagic fever with renal syndrome in China. *Chin Med J (Engl)* 1999; **112**(5): 472-7.
85. Zhang S, Wang S, Yin W, et al. Epidemic characteristics of hemorrhagic fever with renal syndrome in China, 2006-2012. *BMC Infect Dis* 2014; **14**: 384.
86. Wang L, Wang T, Cui F, et al. Hemorrhagic Fever with Renal Syndrome, Zibo City, China, 2006-2014. *Emerg Infect Dis* 2016; **22**(2): 274-6.
87. Dai X, Jian C, Li N, Li D. Characterization of the L genome segment of an orthohantavirus isolated from Niviventer confucianus. *Arch Virol* 2019; **164**(2): 613-6.
88. Tang K, Zhang Y, Zhang C, et al. Hantaan virus-induced elevation of plasma osteoprotegerin and its clinical implications in hemorrhagic fever with renal syndrome. *Int J Infect Dis* 2022; **126**: 14-21.
89. Xu FL, Yang ZQ, Yang CC, Xiao SY, Xiao H, Wen L. Serological characterization of a hantavirus from Hubei, China. *Acta Virol* 2004; **48**(1): 5-8.
90. Chen H, Li Y, Zhang P, Wang Y. A case report of empty Sella syndrome secondary to Hantaan virus infection and review of the literature. *Medicine (Baltimore)* 2020; **99**(14): e19734.
91. Sun XF, Zhao L, Zhang ZT, et al. Detection of Imjin Virus and Seoul Virus in Crocidurine Shrews in Shandong Province, China. *Vector Borne Zoonotic Dis* 2017; **17**(6): 425-31.
92. Zuo SQ, Li XJ, Wang ZQ, et al. Genetic Diversity and the Spatio-Temporal Analyses of Hantaviruses in Shandong Province, China. *Front Microbiol* 2018; **9**: 2771.
93. Zhang Y, Zhang H, Dong X, et al. Hantavirus outbreak associated with laboratory rats in Yunnan, China. *Infect Genet Evol* 2010; **10**(5): 638-44.
94. Ma C, Yu P, Nawaz M, et al. Hantaviruses in rodents and humans, Xi'an, PR China. *J Gen Virol* 2012; **93**(Pt 10): 2227-36.
95. Liu J, Liu DY, Chen W, et al. Genetic analysis of hantaviruses and their rodent hosts in central-south China. *Virus Res* 2012; **163**(2): 439-47.
96. Sui X, Zhang X, Fei D, Zhang Z, Ma M. Simultaneous rapid detection of Hantaan virus and Seoul virus using RT-LAMP in rats. *PeerJ* 2019; **6**: e6068.
97. Wang T, Liu J, Zhou Y, et al. Prevalence of hemorrhagic fever with renal syndrome in Yiyuan County, China, 2005-2014. *BMC Infect Dis* 2016; **16**: 69.
98. Wu G, Xia Z, Wang F, et al. Investigation on risk factors of haemorrhagic fever with renal syndrome (HFRS) in Xuancheng City in Anhui Province, Mainland China. *Epidemiol Infect* 2020; **148**: e248.
99. Yao PP, Chen G, Xu F, et al. [Genotype and evolution of hantavirus in Tiantai of Zhejiang province, 2011-2018]. *Zhonghua Liu Xing Bing Xue Za Zhi* 2019; **40**(10): 1285-90.
100. Zhang YZ, Zou Y, Yan YZ, et al. Detection of phylogenetically distinct Puumala-like viruses from red-grey vole Clethrionomys rufocanus in China. *J Med Virol* 2007; **79**(8): 1208-18.
101. Cao S, Ma J, Cheng C, Ju W, Wang Y. Genetic characterization of hantaviruses isolated from rodents in the port cities of Heilongjiang, China, in 2014. *BMC Vet Res* 2016; **12**: 69.
102. Shortridge KF, Lee HW, Le Duc JW, Wong TW, Chau GW, Rosen L. Serological evidence of Hantaan-related viruses in Hong Kong. *Trans R Soc Trop Med Hyg* 1987; **81**(3): 400-2.
103. Xiao H, Lin X, Gao L, et al. Ecology and geography of hemorrhagic fever with renal syndrome in Changsha, China. *BMC Infect Dis* 2013; **13**: 305.
104. Fan X, Deng H, Sang J, et al. High Serum Procalcitonin Concentrations in Patients With Hemorrhagic Fever With Renal Syndrome Caused by Hantaan Virus. *Front Cell Infect Microbiol* 2018; **8**: 129.
105. Tian HY, Yu PB, Luis AD, et al. Changes in rodent abundance and weather conditions potentially drive hemorrhagic fever with renal syndrome outbreaks in Xi'an, China, 2005-2012. *PLoS Negl Trop Dis* 2015; **9**(3): e0003530.
106. Zou Y, Hu J, Wang ZX, et al. Genetic characterization of hantaviruses isolated from Guizhou, China: evidence for spillover and reassortment in nature. *J Med Virol* 2008; **80**(6): 1033-41.
107. Li J, Zhao ZT, Wang ZQ, Liu YX, Hu MH. Nucleotide sequence characterization and phylogenetic analysis of hantaviruses isolated in Shandong Province, China. *Chin Med J (Engl)* 2007; **120**(9): 825-30.
108. Ma Xuemin MJ, Li Haijun, Yan Yi, Zhan Jun, Zhang Zheng. Alysis of host animal surveillance of rel syndrome hemorrhagic fever in Jingyuan County, Ningxia, 2005-2012. *Jourl of Ningxia Medical University* 2014; **36**(2): 187-90.
109. Wu Rui YP, Li Jing, Du Quanli, Chen Hailong, Liu Jifeng, Li Hengxin, Ma Chaofeng. Complete

genome sequencing and alysis of hantavirus Xi'an isolate. *Shaanxi Medical Jourl* 2013; (12): 15-6+9.
110. Yan YC, Liu XL, Yang ZB, Li ZL. [Propagation and characterization of the etiologic agent of epidemic hemorrhagic fever in cultured A-549 cells]. *Zhongguo Yi Xue Ke Xue Yuan Xue Bao* 1982; **4**(2): 67-72.
111. Zhou Jihua ZH, Yang Weihong, Zhangyu Earthquake. Surveillance of hemorrhagic fever with rel syndrome in Yunn Province in 2008. *Medical animal control* 2009.
112. Yao Zhihui DG, Zhang Jiake, Yu Yongxin, Liu Xuecheng, Liu Wenxue, Zhang Linglin, Yan Dongyou. Genetic characteristics of hantavirus strain s85-46 isolated from Sichuan. *Chinese Jourl of Epidemiology* 2001; **22**(6): 449-51.
113. Yao Zhihui DG, Yu Yongxin, Zhang Jiake, Liu Xuecheng, Zhang Linglin, Yan Dongyou. A new subtype of hantavirus sn7-HTN isolated from Sichuan. *Chinese Jourl of Infectious Diseases* 2002; **20**(2): 4.
114. Liu Fuqiang GL, Dai Defang, Zhang Hong, Zeng Ge, Guo Shouheng, Wu Zigui, Hu Shixiong. Surveillance of hemorrhagic fever with rel syndrome in Hun Province in 2006. *Chinese Jourl of Preventive Medicine* 2008; **9**(6): 4.
115. Zhang Yongzhen XQ, Li Minghui, Zou Yang, Lv Wei, Dai Defang, Chen Huaxin. An epjdemiologic inVestigation Of hantaViruses carried by rOdent hoSts in Hun province. *Chinese Jourl of Epidemiology* 2007; **28**(1): 5.
116. Liu Tian XS, Mei Fangsheng, Hao Haibo, Nie Xiaopei, Huang Jigui, Yao Menglei, Liu Li, Yang Rui, Hu Bing, Wu Yang, Tong Yeqing, Chen Qi. Surveillance results of host animals of hemorrhagic fever with rel syndrome in Jingzhou， Hubei province， Chi from 2017 to 2018. *Chinese Jourl of Vector Biology and Control* 2020; **31**(4): 5.
117. Jing L. Genetic evolution of hantavirus and its host in hubei province: WuHan University; 2011.
118. Kang YJ, Zhou DJ, Tian JH, et al. Dynamics of hantavirus infections in humans and animals in Wuhan city, Hubei, China. *Infect Genet Evol* 2012; **12**(8): 1614-21.
119. Liu Shiwen XG, Gong Tian, Xiong Ying, Shi Yong, Li Jianxiong, Liu Xiaoqing, Xiao Fang, Zhang Yanni, Zhou Jun. Characteristics of hantavirus gene carried by rodents in Jiangxi Province. *Chinese Jourl of Vector Biology and Control* 2015; **26**(5): 5.
120. Shiwen L, Ying X, Tian G, et al. Sequence Analysis of the Whole Genome of Hantaan Virus Isolate AYW89-15 in Jiangxi Province. *Chinese Journal of Zoonoses* 2017; **33**(12): 5.
121. Xu Fang ZH, Yao Pingping, Hu Dan, Zhang Yun, Xie Ronghui, Yang Zhangnu, Qian Lei, Zhu Jin. Isolation and identification of hantavirus from rodents in Zhejiang Province from 2008 to 2011. *Chinese Jourl of Vector Biology and Control* 2013; **24**(4): 4.
122. Xu Fang YP, Zhu Hanping, Xie Ronghui. Isolation and identification of three strains of hemorrhagic fever with rel syndrome virus. *Chinese Jourl of Vector Biology and Control* 2008; **19**(6): 2.
123. Hu Qun TS, Guo Liping. Genotyping and sequence alysis of hantavirus carried by rodents in Daxie port area. *Chinese Jourl of Frontier Health and Quarantine* 2010; **33**(4): 5.
124. Cui Beijin YH, Wu Bingyao, He Jiang, Hu Shuangshuang, Yang Qinggui, Sun Lixin. Investigation and alysis of pathogens carried by rodents at Jiangsu port from 2014 to 2015. *Chinese sanitary insecticide* 2016; (1): 4.
125. Hou Yong LD, Liu Yupeng, Ju Wendong, Cheng Cheng, Gao Danning, Fu Yingqun, Zhao Yashuang. Investigation and alysis of hantavirus molecular prevalence in rodents at Heilongjiang Chi Russia border port from 2009 to 2010. *Chinese Jourl of Frontier Health and Quarantine* 2012; **35**(3): 4.
126. Suya C. Molecular Epidemiological Study on Hantavirus Infection of Rodents in Heilongjiang Port Cities: Northeast Forestry University; 2016.
127. Chen Shuhong CL, Liu Yancheng, Li Jihong, Hui Shan. Isolation and gene identification of a Hantaan virus of Hantaan type in Heilongjiang Province. *Chinese Jourl of tural Medicine* 2008.
128. Yao Kun JJ, Zhang Wenyi, Zhao Qiumen, Jiang Xining, Zuo Shuqing, Wu Xiaoming, Zhan Lin, Chu Chenyi, Zhang Panhe, Yang Hong, Cao Wuchun. Molecular epidemiological investigation of hantavirus infection in host animals in some areas of Northeast Chi. *Chinese Jourl of Pathogenic Biology* 2007; **2**(1): 4.
129. Xian C. Isolation and evolutionary analysis of hantavirus in Jilin Province: Sichuan University; 2005.
130. Qingli Y, Pengfei Y, Lijun S, et al. Study on S Gene Characteristics of Hantavirus Carried by Apodemus agrarius in Changbai County. *Journal of Virology* 2013; **29**(4): 4.
131. Shen Bo PD, Wu Donglin, Xu Shuang, Wu Yanping, Yang Xianda, Zhang Min, Hou Mei. Isolation and identification of hantavirus in Fusong are. *Chi Health Engineering* 2012; **11**(2): 4.
132. Liang M, Li D, Xiao SY, Hang C, Rossi CA, Schmaljohn CS. Antigenic and molecular characterization of hantavirus isolates from China. *Virus Res* 1994; **31**(2): 219-33.
133. Duan Zhengxiu LM, Yu Juan, Wang Wen, Guo Wenping, Zhang Yongzhen. Comparison on the

difference of virulence between Hantaan virus and Seoul virus isolated both from Rattus norvegicus. *Chinese Jourl of Epidemiology* 2009; (10): 4.
134. Liu Jing CY, Lin Daihua, Xiao Fangzhen, Lin Wen, Wang Jiaxiong, Han Tengwei, Liu Weijun. Epidemiological characteristics and host animal monitoring of hemorrhagic fever with rel syndrome in Fujian province, China, 2016-2018. *Chinese Jourl of Vector Biology and Control* 2021.
135. Yan QL, Yang PF, Shao LJ, et al. [The analysis of hantavirus S gene in Apodemus agrarius in Changbai area]. *Bing Du Xue Bao* 2013; **29**(4): 382-5.
136. Wang Yulan CD, Xu Yan, Zhang Jin, Fu Rukun, Shi Shulu. Surveillance on rats and hantavirus detection in Rizhao plain area. *Chinese Jourl of Frontier Health and Quarantine* 2016; **39**(6): 4.
137. Geng Yingzhi TJ, Liu Yun, Wang Bo, Sun Yingwei, Li Xin, Yao Wenqing. Genetic features and distribution of Hantaan virus in Liaoning province, Chi. *Chinese Jourl of Vector Biology and Control* 2012; **23**(5): 4.
138. Ma Chao LM, Zhang Fengxian, Zhang Yongzhen. Genotyping of hantavirus in rodents in hulunbeier city. *Jourl of shihezi university* 2007; (6): 5.
139. Sun Yanfeng HK, Wu Yongping. Alysis of serum diagnosis results of hemorrhagic fever with rel syndrome cases in Baoji City. *Medical animal control* 2016; (9): 3.
140. Peng Yan ZW, Li Guoming. Genetic Characteristics of Hantavirus Carried by Rodents in Xiantao District, Hubei Province in 2012. *Laboratory Medicine and Clinical* 2015; **12**(18): 2.
141. Fang LZ, Zhao L, Wen HL, et al. Reservoir host expansion of hantavirus, China. *Emerg Infect Dis* 2015; **21**(1): 170-1.
142. Lu T, Fu Y, Hou Y, et al. Hantavirus RNA Prevalence in Myomorph Rodents on Bolshoy Ussuriysky Island at the Sino-Russian Border. *Vector Borne Zoonotic Dis* 2017; **17**(8): 588-95.
143. Fang LQ, Wang XJ, Liang S, et al. Spatiotemporal trends and climatic factors of hemorrhagic fever with renal syndrome epidemic in Shandong Province, China. *PLoS Negl Trop Dis* 2010; **4**(8): e789.
144. Tian Z, Yao N, Wu Y, Wang F, Zhao Y. Serum superoxide dismutase level is a potential biomarker of disease prognosis in patients with hemorrhagic fever with renal syndrome caused by the Hantaan virus. *BMC Infect Dis* 2022; **22**(1): 446.
145. Li R, Sun J, Chen Y, et al. Clinical and laboratory features and factors predicting disease severity in pediatric patients with hemorrhagic fever with renal syndrome caused by Hantaan virus. *J Med Virol* 2022.
146. Kariwa H, Zhong CB, Araki K, et al. Epizootiological survey of hantavirus among rodent species in Ningxia Hui Autonomous Province, China. *Jpn J Vet Res* 2001; **49**(2): 105-14.
147. Xiao SY, Liang M, Schmaljohn CS. Molecular and antigenic characterization of HV114, a hantavirus isolated from a patient with haemorrhagic fever with renal syndrome in China. *J Gen Virol* 1993; **74 (Pt 8)**: 1657-9.
148. Cai YN, Han X, Wei YM, et al. [Spatial-temporal cluster of hemorrhagic fever with renal syndrome in Hebei province, 2005-2016]. *Zhonghua Liu Xing Bing Xue Za Zhi* 2019; **40**(8): 930-5.
149. Fang LQ, Li CY, Yang H, et al. [Using geographic information system to study on the association between epidemic areas and main animal hosts of hemorrhagic fever with renal syndrome in China]. *Zhonghua Liu Xing Bing Xue Za Zhi* 2004; **25**(11): 929-33.
150. Liu DY, Liu J, Li JL, et al. [Genetic analysis on S segment of hantaviruses in rodent hosts from Wuhan area, Hubei province]. *Zhonghua Liu Xing Bing Xue Za Zhi* 2012; **33**(8): 828-31.
151. Li YL, Ruo SL, Tong Z, et al. A serotypic study of hemorrhagic fever with renal syndrome in rural China. *Am J Trop Med Hyg* 1995; **52**(3): 247-51.
152. Xu XA, Ruo SL, Tang YW, Fisher-Hoch SP, McCormick JB. Molecular characterization and expression of glycoprotein gene of Hantavirus R22 strain isolated from Rattus norvegicus in China. *Virus Res* 1991; **21**(1): 35-52.
153. Tang YW, Ruo SL, Xu X, et al. Hantavirus strains isolated from rodentia and insectivora in rural China differentiated by polymerase chain reaction assay. *Arch Virol* 1990; **115**(1-2): 37-46.
154. Wang W, Lin XD, Guo WP, Li MH, Zhang YZ. [Genetic analysis of hantaviruses carried by Rattus norvegicus collected from Henan and Neimenggu provinces, China]. *Zhonghua Liu Xing Bing Xue Za Zhi* 2010; **31**(9): 1030-4.
155. Yang Z, Miao Z, Liu Y, et al. [A study on the epidemiology and preventive measure on epidemic haemorrhage fever in army barrack area in Shandong]. *Zhonghua Liu Xing Bing Xue Za Zhi* 2000; **21**(1): 30-3.
156. Chen HX, Qiu FX. Epidemiologic surveillance on the hemorrhagic fever with renal syndrome in China. *Chin Med J (Engl)* 1993; **106**(11): 857-63.
157. Wang JJ, Wei ZZ, Wei J, et al. [Long term epidemiological effects of vaccination on hemorrhagical fever with renal syndrome (HFRS) in Shaanxi provincial HFRS epidemic areas]. *Zhonghua Liu Xing Bing Xue Za Zhi* 2012; **33**(3): 309-12.

158. Chen LF, Chen SH, Wang KL, Zhang J, Li JH. [Characterization of S gene of a strain of hantavirus isolated from Apodemus peninsulae in Heilongjiang Province]. *Bing Du Xue Bao* 2012; **28**(5): 517-21.
159. Wang ZQ, Wang YL, Fu JH, et al. [Molecular analysis of hantavirus isolated from Shandong province]. *Zhonghua Shi Yan He Lin Chuang Bing Du Xue Za Zhi* 2003; **17**(2): 121-3.
160. Li Q, Chen W, Yang ZQ. [The reactivity of sera from hemorrhagic fever in patients with renal syndromes to the recombination nucleotide proteins from European hantaviruses in Hubei province]. *Zhonghua Liu Xing Bing Xue Za Zhi* 2008; **29**(6): 577-80.
161. Zhang ZR, Zhao HR, Li Q. [Study on the geographic distribution and serological typing of HFRS in Hebei Province]. *Zhonghua Liu Xing Bing Xue Za Zhi* 1996; **17**(2): 87-90.
162. Liu JW, Lee BJ, Ko WC, Chuang YC. Hemorrhagic fever with renal syndrome: first imported case of hantavirus infection in Taiwan. *J Formos Med Assoc* 1996; **95**(6): 480-3.
163. Yang S, Liu J, Song J. [Seroepidemiologic study on hemorrhagic fever with renal syndrome virus in the migration areas of the Three-Gorges Reservoir Chongqing, Region, Yangtze River]. *Zhonghua Liu Xing Bing Xue Za Zhi* 2001; **22**(4): 284-6.
164. Luo Z, Liu Y, Liu H, Wang Y, Zhao Y. [Discovery of two sub-types of Hantanvirus in Anhui mountain areas]. *Zhonghua Liu Xing Bing Xue Za Zhi* 2002; **23**(5): 363-5.
165. Lu S, Zhu N, Guo W, et al. RNA-Seq Revealed a Circular RNA-microRNA-mRNA Regulatory Network in Hantaan Virus Infection. *Front Cell Infect Microbiol* 2020; **10**: 97.
166. Hansen A, Cameron S, Liu Q, et al. Transmission of haemorrhagic fever with renal syndrome in china and the role of climate factors: a review. *Int J Infect Dis* 2015; **33**: 212-8.
167. Yao ZO, Yang WS, Zhang WB, Bai XF. The distribution and duration of hantaan virus in the body fluids of patients with hemorrhagic fever with renal syndrome. *J Infect Dis* 1989; **160**(2): 218-24.
168. Lu DH, Jiang H, Lian JQ. Hantavirus Infection during Pregnancy. *Virol Sin* 2021; **36**(3): 345-53.
169. Jiang H, Du H, Wang LM, Wang PZ, Bai XF. Hemorrhagic Fever with Renal Syndrome: Pathogenesis and Clinical Picture. *Front Cell Infect Microbiol* 2016; **6**: 1.
170. Jiang F, Zhang Z, Dong L, et al. Prevalence of hemorrhagic fever with renal syndrome in Qingdao City, China, 2010-2014. *Sci Rep* 2016; **6**: 36081.
171. Zhang R, Mao Z, Yang J, et al. The changing epidemiology of hemorrhagic fever with renal syndrome in Southeastern China during 1963-2020: A retrospective analysis of surveillance data. *PLoS Negl Trop Dis* 2021; **15**(8): e0009673.
172. Jiang W, Yu HT, Zhao K, et al. Quantification of Hantaan virus with a SYBR green I-based one-step qRT-PCR assay. *PLoS One* 2013; **8**(11): e81525.
173. Niklasson BS. Haemorrhagic fever with renal syndrome, virological and epidemiological aspects. *Pediatr Nephrol* 1992; **6**(2): 201-4.
174. Jiang W, Wang PZ, Yu HT, et al. Development of a SYBR Green I based one-step real-time PCR assay for the detection of Hantaan virus. *J Virol Methods* 2014; **196**: 145-51.
175. Wang ML, Lai JH, Zhu Y, et al. Genetic susceptibility to haemorrhagic fever with renal syndrome caused by Hantaan virus in Chinese Han population. *Int J Immunogenet* 2009; **36**(4): 227-9.
176. Ting G. Epidemic status of hemorrhagic fever with renal syndrome and investigation of host animals in some areas in Yunnan from 2014 to 2015: Dali University; 2008.
177. Dongjing Y. Molecular Epidemiological Analysis of Hantavirus in Tianjin and Study on Construction and Expression of GP Multiple Epitope Antigen Gene: Tianjin Medical University; 2009.
178. Kun Y. Epidemiological study on hantavirus infection in animals in some areas of China. *PLA Academy of Military Medical Sciences* 2008.
179. You Z. Molecular epidemiology of viruses carried by important animal hosts in hainan island: Hainan Medical College; 2018.
180. Mengying J. Preliminary study on the infection of a kind of sand grain virus in host animals and febrile patients in yunnan province. *Dali University* 2019.
181. Liyong H. Epidemic characteristics and periodicity of hemorrhagic fever with renal syndrome in China: Chinese Center for Disease Control and Prevention; 2012.
182. Ivanov A, Dekonenko A, Dzagurova T, et al. Outbreak of hemorrhagic fever with kidney syndrome in Egor'ev district of Moscow region. 2000; **45**(4): 33-6.
183. Dekonenko A, Tkachenko E, GIu L, et al. Genetic differentiation of hantaviruses using the polymerase chain reaction and sequencing. 1996; **41**(1): 24-7.
184. Yao P, Zhu H, Xu F, et al. Molecular characterization and sequence comparison of the M and S segments of Hantavirus ZJ5 strain. 2008; **22**(6): 434-6.
185. Antic D, Lim B-U, Kang CYJVr. Molecular characterization of the M genomic segment of the Seoul 80-39 virus; nucleotide and amino acid sequence comparisons with other hantaviruses reveal the evolutionary pathway. 1991; **19**(1): 47-58.
186. Lee S-H, Hong SP, Shin Y-C, Noh K-S, Kim H-S, Kim S-OJDS. Nucleotide sequence of

nucleocapsid protein (N) of Hantaan virus isolated from a Korean hemorrhagic fever patient. 1997; **7**(6): 349-52.
187. Reip A, Haring B, Sibold C, et al. Coding strategy of the S and M genomic segments of a hantavirus representing a new subtype of the Puumala serotype. 1995; **140**(11): 2011-26.
188. Yoo D, Kang CYJNar. Nucleotide sequence of the M segment of the genomic RNA of Hantaan virus 76-118. 1987; **15**(15): 6299.
189. Yashina L, Mishin V, Zdanovskaya N, Schmaljohn C, Ivanov LJEID. A newly discovered variant of a hantavirus in Apodemus peninsulae, far Eastern Russia. 2001; **7**(5): 912.
190. Lloyd G, Jones NJJoI. Infection of laboratory workers with hantavirus acquired from immunocytomas propagated in laboratory rats. 1986; **12**(2): 117-25.
191. Xiao S-Y, Leduc JW, Chu YK, Schmaljohn CSJV. Phylogenetic analyses of virus isolates in the genus Hantavirus, family Bunyaviridae. 1994; **198**(1): 205-17.
192. Kim HC, Kim WK, Klein TA, et al. Hantavirus surveillance and genetic diversity targeting small mammals at Camp Humphreys, a US military installation and new expansion site, Republic of Korea. *PLoS One* 2017; **12**(4): e0176514.
193. Yadav PD, Vincent MJ, Nichol ST. Thottapalayam virus is genetically distant to the rodent-borne hantaviruses, consistent with its isolation from the Asian house shrew (Suncus murinus). *Virol J* 2007; **4**: 80.
194. Kariwa H, Yoshimatsu K, Sawabe J, et al. Genetic diversities of hantaviruses among rodents in Hokkaido, Japan and Far East Russia. *Virus Res* 1999; **59**(2): 219-28.
195. Shi X, Liang M, Hang C, Song G, McCaughey C, Elliott RM. Nucleotide sequence and phylogenetic analysis of the medium (M) genomic RNA segments of three hantaviruses isolated in China. *Virus Res* 1998; **56**(1): 69-76.
196. Schmaljohn CS. Nucleotide sequence of the L genome segment of Hantaan virus. *Nucleic Acids Res* 1990; **18**(22): 6728.
197. Qu YG, Yang GQ, Zou Y, Yan GQ, Chen HX, Zhang YZ. [Isolation and characterization of Hantavirus carried by rodents in Huludao, Liaoning province]. *Zhonghua Liu Xing Bing Xue Za Zhi* 2006; **27**(6): 513-7.
198. Yashina LN, Patrushev NA, Ivanov LI, et al. Genetic diversity of hantaviruses associated with hemorrhagic fever with renal syndrome in the far east of Russia. *Virus Res* 2000; **70**(1-2): 31-44.
199. Vapalahti O, Lundkvist A, Fedorov V, et al. Isolation and characterization of a hantavirus from Lemmus sibiricus: evidence for host switch during hantavirus evolution. *J Virol* 1999; **73**(7): 5586-92.
200. Razzauti M, Plyusnina A, Henttonen H, Plyusnin A. Accumulation of point mutations and reassortment of genomic RNA segments are involved in the microevolution of Puumala hantavirus in a bank vole (Myodes glareolus) population. *J Gen Virol* 2008; **89**(Pt 7): 1649-60.
201. Schmaljohn CS, Schmaljohn AL, Dalrymple JM. Hantaan virus M RNA: coding strategy, nucleotide sequence, and gene order. *Virology* 1987; **157**(1): 31-9.
202. Klein TA, Kang HJ, Gu SH, et al. Hantaan virus surveillance targeting small mammals at Dagmar North Training Area, Gyeonggi Province, Republic of Korea, 2001-2005. *J Vector Ecol* 2011; **36**(2): 373-81.
203. Park K, Lee SH, Kim J, et al. A Portable Diagnostic Assay, Genetic Diversity, and Isolation of Seoul Virus from Rattus norvegicus Collected in Gangwon Province, Republic of Korea. *Pathogens* 2022; **11**(9).
204. Arai S, Ohdachi SD, Asakawa M, et al. Molecular phylogeny of a newfound hantavirus in the Japanese shrew mole (Urotrichus talpoides). *Proc Natl Acad Sci U S A* 2008; **105**(42): 16296-301.
205. Song JW, Baek LJ, Kim SH, et al. Genetic diversity of Apodemus agrarius-borne hantaan virus in Korea. *Virus Genes* 2000; **21**(3): 227-32.
206. Kariwa H, Tkachenko EA, Morozov VG, et al. Epidemiological study of hantavirus infection in the Samara Region of European Russia. *J Vet Med Sci* 2009; **71**(12): 1569-78.
207. Noh JY, Cheong HJ, Song JY, et al. Clinical and molecular epidemiological features of hemorrhagic fever with renal syndrome in Korea over a 10-year period. *J Clin Virol* 2013; **58**(1): 11-7.
208. Zhu Yuefen ZQ. HFRS monitoring report of Xishan City from 1995 to 1998. *Jiangsu Health Care* 2000; **2**(1): 1.
209. Wang Fukun LJ, Xu Qihua. Surveillance and alysis of hemorrhagic fever from 1993 to 2002. *Medicine Forum Magazine* 2005; **26**(19): 2.
210. Yuanling L. Epidemic development alysis of hemorrhagic fever with rel syndrome from 1992 to 2006 in Tai'an City. *Jourl of Preventive Medicine Information* 2008; **24**(6): 3.
211. Sun Liping HY. Investigation on hemorrhagic fever with rel syndrome in Hulunbuir league from 1990 to 1999. *Jourl of Disease Control* 2002; **018**(004): 471-.
212. Luo Qinghong LF, Wen Jianhua, Hao Ruifeng, Huang Mantao. Investigation on epidemic foci of epidemic hemorrhagic fever in seven cities and counties of Guangdong Province in 1984. *Hygiene and*

epidemic prevention in Guangdong 1987; (3).
213. Guo Jianhua ZB, Liu Li, Zhang Shuangzhai, Zhou Jikun. Alysis of epidemic characteristics of hemorrhagic fever with rel syndrome in Shijiazhuang, Hebei Province from 1984 to 2008. *Disease surveillance* 2009; (10): 4.
214. Wei Shuhui WJ, Chu Hong, Shang Jing, Zhao Guoliang, Zeng Qiang. Epidemiological investigation on hemorrhagic fever with rel syndrome in Chengde city from 1984 to 2008. *Chinese Jourl of Vector Biology and Control* 2010; (6): 3.
215. Li Xinglong YY, Zhao Shumei, Li Yuanhong. Surveillance of hemorrhagic fever with rel syndrome in Taiyuan from 1984 to 2003. *Disease surveillance* 2007; **22**(7): 3.
216. Liu Xuecheng ZJ, Chen Danlin, Lai Wenhong, Wu Haiyan, Yu Jia, Xie Shihong. Surveillance of hemorrhagic fever with rel syndrome in Sichuan Province from 1984 to 2000. *Jourl of Preventive Medicine Information* 2002; **18**(6): 3.
217. Wang Tingzhe CW, Liu Mingzhong. Alysis of surveillance results of epidemic hemorrhagic fever in Shenzhen from 1984 to 1999. *Public Health in China* 2000; **16**(5): 1.
218. Hong L. Epidemiological investigation and alysis of EHF in Sishui county from 1982 to 2008. *Medical animal control* 2009; **25**(7): 3.
219. Gao Mingqi LH. Epidemiological investigation of epidemic hemorrhagic fever in Sishui county from 1982 to 2002. *Jourl of Strait Preventive Medicine* 2003; **9**(4): 2.
220. Zhang Zonghui LJ, Wu Peng, Tian Hongrui, Jiang Beiping, Huang Shunhe, Luo Chengyuan. HFRS Surveillance in nchong，1961－2010. *Jourl of Preventive Medicine Information* 2012; **28**(4): 4.
221. Zhang Wuzhuang ZF, Liu Jingrong. Surveillance and alysis of hemorrhagic fever with rel syndrome in Zhouning County in 1998. *Public Health in China* 1999; **15**(7): 1.
222. Chen Renqiang SB, Wang ishuo, Chang Jinliang, Sun Jianmin. Epidemiological investigation of epidemic hemorrhagic fever in Yuncheng County in 1998. *Health of urban and rural enterprises in Chi* 1999; (4): 1.
223. Li Shiqing CR, He Simi, Zhang Wuzhuang, Liu Jingrong, Xiao Shusheng, Zheng Dingxiang, Huang Lianzhi, Zheng Qingjiang. Alysis of HFRS monitoring in Fujian Province in 1998. *Public Health in China* 2009; **15**(5): 3.
224. Wei L. Epidemic characteristics of hemorrhagic fever with rel syndrome in Shaodong county from 1998 to 2000. *Practical Preventive Medicine* 2002.
225. Hore Z. Report on surveillance results of HFRS among rats in Changping District from 1997 to 2001. *Disease surveillance* 2002; **17**(11): 1.
226. Chen Lifeng LX, Chen Jia. Alysis of monitoring results of hemorrhagic fever with rel syndrome in Yuyao City from 1996 to 2015. *Preventive Medicine Forum* 2016; **28**(7): 3.
227. Chen Lianping ZY. Surveillance and alysis of hemorrhagic fever with rel syndrome in Xianju County from 1996 to 2006. *Chinese Jourl of Vector Biology and Control* 2007; **18**(4): 345-.
228. Xie Shuyun WZ, Zhang Lei, Ling Feng, Gong Zhenyu, Weng Jingqing. Surveillance of hemorrhagic fever with rel syndrome in Zhejiang Province from 1995 to 1996. *Disease surveillance* 1998; **9**(4): 299-.
229. Zhang Minglei WL. Surveillance of hemorrhagic fever with rel syndrome in Ganyu，1995-2014. *Modern Preventive Medicine* 2016; **43**(7): 5.
230. Chang Yancun JX, Wang Chenxia. Surveillance and alysis of hemorrhagic fever with rel syndrome in LUANN COUNTY, Hebei Province from 1995 to 2005. *Chinese Jourl of Vector Biology and Control* 2009; **20**(5): 1.
231. Lv Jing WS, Zeng Qiang, Ji Wenbo. Surveillance and alysis of hemorrhagic fever with rel syndrome in Chengde summer resort from 2003 to 2005. *Modern Jourl of Integrated Chinese and Western Medicine* 2003; **19**(2): 3.
232. Jiang Jianxiang YD, Dang Qinghui, Liu Xuezhen, and Gai Yongzhi. Alysis of surveillance results of hemorrhagic fever with rel syndrome in Gansu Province in 2002. *Medical animal control* 2003; **19**(2): 3.
233. Fang Chunfu YZ, Wang Wei. Surveillance and alysis of hemorrhagic fever with rel syndrome in Quzhou City from 2002 to 2004. *Disease surveillance* 2005; **20**(10): 513-7.
234. Li Dahan GR. Alysis of surveillance data of hemorrhagic fever with rel syndrome in Ganyu County in 2001. *Preventive medicine literature information* 2003; **009**(001): 112-3.
235. Bai Jingjing WX, Liu Li, Guan Xuhua, Xing Xuesen, Wu Yang, Liu Man, Guo Yanping. Epidemiological characteristics of hemorrhagic fever with rel syndrome in Hubei Province from 2001 to 2015. *Modern Preventive Medicine* 2016; **43**(20): 6.
236. Jing Qinlong LL, Xiao Xincai, Xu Yang, Song Shaofang, Di Biao, Wang Ming, Lu Jiahai, Yang Zhicong. Surveillance on hemorrhagic fever with rel syndrome in Guangzhou，2001－2010. *Jourl of Tropical Medicine* 2012; **12**(1): 5.
237. Zhu Liye WJ, Liu Qiquan, Zhou Huatao, Ding Zhentao. Alysis of HFRS surveillance in Fuyang city

from 2000 to 2006. *Anhui Jourl of Preventive Medicine* 2007; **13**(4): 4.
238. Zhang Yingchao LD, Li Dejun, Zhang Hengqian, Zheng Li, Sun Maoli. Alysis of surveillance results of hemorrhagic fever with rel syndrome in Dalian from 2000 to 2006. *Disease surveillance* 2007; **22**(6): 2.
239. Fang Chunfu WC, Yu Zhangyou, Gao Yi. Surveillance of epidemic hemorrhagic fever in Quzhou City in 1999. *Chin J Zoonoses* 2001.
240. Wang Jinquan TA, Peng Langtao, Gu Ti, Song Zongbi, Liu Fenglan, You Zhongqiong, Chen Yunze. Surveillance of Hantaan virus infection in small animals and healthy people in hemorrhagic fever with rel syndrome epidemic area in Guang'an District in 1999. *Chinese Jourl of Vector Biology and Control* 2001; **12**(2): 96-.
241. Yao Pingping XF, Zhu Hanping, Xie Ronghui, Cheng Yinkai, Mei Lingling, Zhu Zhiyong, Deng Xiaozhao, Zhang Yun, Wang Zhongcan. Surveillance and isolation of hantavirus infection in rodents in Zhejiang Province in 2007. *Chinese Jourl of Vector Biology and Control* 2009; **20**(4): 3.
242. Li Junhua CL, Gao Lidong, Liu Yunzhi, Hu Shixiong, Liu Fuqiang, Zeng Ge, Liu Jiahui, Zhang Hong. Epidemiological alysis of hemorrhagic fever with rel syndrome in Hun province from 2007 to 2010. *Chinese Jourl of Preventive Medicine* 2012; **13**(10): 5.
243. Yuan Wei LX, Zhang Jiake, Lin Shihua, Chen Danlin. Surveillance of Hemorrhagic Fever With RelSyndrome in Sichuan Province, 2007 -2009. *Jourl of Preventive Medicine Information* 2010; (10): 4.
244. Chen Shuhong CL, Liu Yancheng, Li Jihong, Hui Shan. Surveillance of hemorrhagic fever with rel syndrome in Heilongjiang Province in 2006. *Chinese Jourl of Vector Biology and Control* 2008; (6): 564-6.
245. Wang Ling LF, Jiang Xiaolin, Cao Haixia, Sun Tao, Zhang Ling, Zhang Yunxiao, Cui Feng, Wang Zhiqiang. Alysis on epidemiological characteristics of hemorrhagic fever with rel syndrome in Zibo city from 2006 to 2019. *Modern Preventive Medicine* 2021.
246. Jiang Jianxiang YD, Zhao Hai. Surveillance of hemorrhagic fever with rel syndrome in Gansu, 2006 − 2011. *Disease surveillance* 2013; (2): 4.
247. Zhang Yunzhi ZH, Mi Zhuqing, Yang Weihong, Ya Hongxiang, Yuan Qinghong, Feng Yun, Zhang Yuzhen. Monitoring of hemorrhagic fever with rel syndrome in Yunn province, Chi, 2005. *Chinese Jourl of Vector Biology and Control* 2008; **19**(2): 3.
248. Wei Xueqian ZZ. Alysis of the Surveillance of Hemorrhagic Fever with Rel Syndrome in Tangshan City in 2005. *Preventive Medicine Forum* 2008; **14**(3): 2.
249. Ma Weisheng WZ, Wang Yulu, Wang Mei, Wang Xianjun, Bi Zhenqiang. Alysis on the Surveillance of Hemorrhagic Fever with Rel Syndrome in Shandong in2005. *Preventive Medicine Forum* 2007; **13**(3): 2.
250. Yunxia X. 2005-2009 Jintan City rel syndrome hemorrhagic fever surveillance alysis. *Jiangsu Preventive Medicine* 2011; **22**(2): 3.
251. Yu Ping WX, Chen Zhao, Zhu Yefei. Surveillance and alysis of hemorrhagic fever with rel syndrome in Gao'an City, Jiangxi Province from 2005 to 2016. *Disease surveillance* 2017; **32**(10): 4.
252. Gao Lifen WX, Hu Haimei, Luo Qiongmei, Yan Yong, Qian Fabao, Hu Qiuling, Li Rui. Alysis on epidemiological features of hemorrhagic fever with rel syndrome from 2006 to 2014 in Chuxiong prefecture. *Modern Preventive Medicine* 2017; **44**(4): 5.
253. Tao Xu PX, Jia Chunhui, Luo Yongsong, Shi Mingkun, Zheng Lixin. Surveillance on host animals of hemorrhagic fever with rel syndrome in Qinhuangdao City from 2005-2013. *Occupation and health* 2015; (21): 3.
254. Cui Qingrong PW, Ge Junhua, Dong Yin. Alysis of the surveillance data on hemorrhagic fever with rel syndrome in Tiantai county, Zhejiang province from 2005 to 2009. *Chinese Jourl of Vector Biology and Control* 2011; **22**(1): 2.
255. Han Zhanying ZY, Yu Qiuli, Wei Yamei, Zhang Wenzeng, Xu Yonggang, Qi Shunxiang, Li Qi. Alysis for surveillance of host animals of hemorrhagic fever with rel syndrome in Hebei province from 2005 − 2006. *Chinese Jourl of Vector Biology and Control* 2007; **18**(4): 312-4.
256. Yang Weihong ZH, Mi Zhuqing, Zhang Yunzhi, Zhang Yuzhen, Ya Hongxiang, Feng Yun, Yuan Qinghong. Surveillance of Hemorrhagic Fever with Rel Syndrome in Yunn Province , Chi, in 2004. *Chinese Jourl of Vector Biology and Control* 2006; **17**(2): 3.
257. Liang Xiaolei ZT, Xu Kailing. Epidemiological alysis of hemorrhagic fever with rel syndrome in Lianyungang between 2004 and 2015. *Modern Preventive Medicine* 2016; **43**(22): 4.
258. Gao Yan ZH, Yuan Luliang, Zhu Baogen. Alysis of epidemic situation of hemorrhagic fever with rel syndrome in Baoding city from 2004 to 2014. *Chinese Jourl of Pathogenic Biology* 2016.
259. Xiaotao C. Epidemiological characteristics of hemorrhagic fever with rel syndrome in Linyi City

from 2004 to 2012. *Occupation and health* 2013; (19): 3.
260. Ya Hongxiang ZH, Zhang Yunzhi, Mi Zhuqing, Yang Weihong, Zhang Yuzhen, Yuan Qinghong, Feng Yun. Investigation on hemorrhagic fever with rel syndrome in western Yunn from 2004 to 2005. *Endemic Diseases Bulletin* 2007.
261. Wu Dajin LJ, Tang Baochai, Zhang Changhao. Surveillance and alysis of hemorrhagic fever with rel syndrome in Zhouning County in 2004. *Jourl of Strait Preventive Medicine* 2006; **12**(2): 2.
262. Han Xu HZ, Wei Yamei, Zhang Yanbo, Qi Shunxiang, Li Qi. Alysis of the surveillance data on host animals in tiol monitoring sites of hemorrhagic fever with rel syndrome in Hebei province in 2011. *Chinese Jourl of Health Inspection* 2013; (15): 3109-11.
263. Pang Weilong GJ, Zhou Xuebing. A surveillance alysis of hemorrhagic fever surveillance of rel syndrome in Tiantai County, Zhejiang province from 2011 to 2016. *Chi Rural Health Administration* 2018; **38**(7): 3.
264. Shi Xuguang SJ, Xu Fang, Lin Junfen, Chai Chengliang, Ling Feng, Wang Wei. Alysis of surveillance data of hemorrhagic fever with rel syndrome in Zhejiang Province in 2010. *Disease surveillance* 2011; **26**(6): 3.
265. Tao Xu PX, Jia Chunhui, Luo Yongsong, Shi Mingkun, Zheng Lixin. Surveillance and epidemic alysis of hemorrhagic fever with rel syndrome in Qinhuangdao City in 2013. *Occupation and health* 2014; (18): 3.
266. Wang Tao WL, Cui Feng, Zhou Yunping. Surveillance and alysis of hemorrhagic fever with rel syndrome in Zibo, 2013-2014. *Modern Preventive Medicine* 2016; **43**(3): 3.
267. Liu Dapeng WQ, Qu Rongwei, Liu Danhong, Sun n, Liu Yingli, Zhang Hengqian, Zhou Yi, Bo Zhijian. Alysis of hantavirus carrying status and gene characteristics in rats in Dalian in 2012. *Preventive Medicine Forum* 2013; **19**(12): 4.
268. Hu Quanbo CS, Hua Hua, Yang Ming. Investigation on the host animals of hemorrhagic fever with rel syndrome in Heilongjiang Province in 2017. *Chinese sanitary insecticide* 2020; **26**(4): 4.
269. Teng Xindong ZK, Zhang Juan, Zhang Jin, Chen Xiaoguang, Xu Hefei, Xu Ying, Zhang Qi. Molecular identification of common rodents at Shandong Port and investigation of Hantavirus carriers in 2016. *Chinese Jourl of Frontier Health and Quarantine* 2017; **40**(3): 3.
270. Peng Yuehua WY, Lei Yujing. Epidemiological characteristics of epidemic hemorrhagic fever in Jilin Province in 2016. *Chinese Jourl of Biological Products* 2017; **30**(12): 5.
271. Li Kelun ZZ, Xie Yiwen. Investigation on rodents and their pathogens in Guotong Logistics City, Chencun, Foshan in 2016. *Chinese Jourl of Frontier Health and Quarantine* 2017; **40**(2): 2.
272. Zhang Tao ZG, Chu Xiang. 2016-2019 Rat Monitoring at Huangdao Port. *Chinese Jourl of Frontier Health and Quarantine* 2020; **43**(5): 2.
273. Teng Xindong ZK, Zhang Juan, Zhang Jin, Chen Xiaoguang, Xu Hefei, Xu Ying, Zhang Qi. Molecular identification and hantavirus detection of common rats at Shandong port. *Chinese Jourl of Frontier Health and Quarantine* 2017; **40**(3): 3.
274. Li Tao WX, Zhang Hualin, Chen Zhao. Surveillance alysis of hemorrhagic fever with rel syndrome and evaluation of vaccine immunization effect in Fengcheng City, Jiangxi Province in 2014. *Preventive Medicine in South China* 2017; **43**(2): 3.
275. Zhou Xiaofeng PW, Zhang Renli, Chen Hongbiao, Cao He. Investigation on rodents and hantavirus in Longhua District, Shenzhen from 2014 to 2019. *Chinese Jourl of Frontier Health and Quarantine* 2020; **43**(2): 3.
276. Li Hongbing FH, Hu Kan, Hu Xiaoqian, Deng Feng, Zhang Kejian, Gao Juan, Yan Chuanyuan, Tian Hui, Zhang Yaning. Surveillance and Alysis of Host Animals of Hemorrhagic Fever with Rel Syndrome in Baoji City from 2014 to 2018. *Chinese sanitary insecticide* 2020; **26**(6): 4.
277. Wang Zheng XZ, Sun Liang, Liang Changliu, Ma Bing. Alysis of monitoring results of hemorrhagic fever with rel syndrome in Yingshang County, Anhui Province from 2010 to 2015. *Disease monitoring and control* 2016.
278. Liu Hong RZ, Wang Yiyin, Yu Zhengchu, Zhang Darong, Gu Lili, Li Furong, Luo Zhaozhuang. Species composition of small mammals and host animals of hemorrhagic fever with rel syndrome in mountainous and forest areas of Anhui Province. *Chinese Jourl of Vector Biology and Control* 1998; **9**(2): 145-7.
279. Sun Chengzhai WZ, Zhai Jinkui, Yang Yuzhi, Jiang Lanqing, Zhu Liye, Liu Yaya, He Suqin, Si Xiaofei, Wang Dongfeng. Distribution of EHF host animals in Fuyang, Anhui Province. *Chin J Zoonoses* 1987; **3**(04): 58-.
280. Liu Yan WS, Wang Jun, Zhang Zhiping, Zhang Fu'an, Wang Yulin, Liu Hong. Investigation and Alysis of Foci Epidemic Areas of HFRS(Hemorrhage Fever with Rel Syndrome)in Dabie Mountain in Anhui Province. *Anhui Jourl of Preventive Medicine* 2003; **9**(5): 3.
281. Wang Jun GL, Zhang Yonggen, Li Qing, Liu Hong. Surveillance of hemorrhagic fever with rel

syndrome in Anhui province，2005 － 2010. *Anhui Jourl of Preventive Medicine* 2012; **18**(2): 4.
282. Pang Zhenqing PR, Lu Shuheng, Qi Yemin, Zheng Zhenwang. Monitoring and alysis of host animals in epidemic focus of hemorrhagic fever with rel syndrome in Cangzhou City. *Medical animal control* 2010.
283. Chaoyong L. Surveillance and magement of epidemic situation of epidemic hemorrhagic fever in Cangzhou City. *Medical Theory and Practice* 2011.
284. Ding Yixin RQ, Zhu Xiangxiu, Zhao Yueping, Xu Shuguang, Li Maochao, Zhang Yonggen, Liu Yan, Lu Zhongqiang, Liu Kun, Gao Cong, Hong Jiahao. Isolation and Identification of Epidemic Hemorrhagic Fever Virus in Different Animals. *Anhui Medicine* 1986.
285. Zhang Jixiang WS, Liu Hengtai. Investigation and alysis of epidemic hemorrhagic fever in Binzhou City in 2000. *Modern Preventive Medicine* 2002.
286. Zhao Rui QJ, Xia Defeng. Alysis of serological monitoring results of hemorrhagic fever in rodent interrel syndrome at Beilun port. *Chinese Jourl of Frontier Health and Quarantine* 2004.
287. Zhang Xiuchun GZ, Zhou Shaolian, Hu Jingyu. The Distribution and Hantavirus Infection Situation of Rodent in Yanshan Mountain of Beijin. *Chinese Jourl of Vector Biology and Control* 2006.
288. Chen Yongliang YY, Dou Xiangfeng, Geng Libin, Jia Lili, Wang Yaqiong, Wang Huayong. Investigation on tural infection of hantavirus among rats in Miyun District of Beijing from 2005 to 2015. *Chinese Jourl of Preventive Medicine* 2017.
289. Zuo Shuqing ZP, Wu Xiaoming, Zhan Lin, Jiang Jiafu, Tang Fang, Sun Peiyuan, Wang Bingcai, Cai Wei, Zhang Jiusong, Yang Hong, Cao Wuchun. Isolation and Prelimiry Identification of Hantavirus from Rattus norvegicus in Beijing. *Chin J Microbiol Immunol* 2005.
290. Cai Wei DZ, Wang Lei, Wang Bingcai, Su Xuan, Ding Jie. Investigation on the tural infectious status of Hantaviruses in rodents in Haidian district, Beijing. *Chinese Jourl of Vector Biology and Control* 2009.
291. Wang Ruiqin TY, Li Sa, Cai Xu, Liu Chongcheng, Jin Yingying. Alysis of rel syndrome hemorrhagic fever surveillance in Changping District, Beijing. *Disease prevention and control notification* 2003.
292. Zuo Shuqing WX, Sun Peiyuan, Zhang Panhe, Wang Bingcai, Tang Fang, Dun Zhe, Cai Wei, Liu Qi, Zeng Fanxin, Jiang Jiafu, Liu Wei, Cao Wuchun. Molecular epidemiology of hantavirus infection in host animals in the northern suburbs of Beijing. *Chinese Jourl of Epidemiology* 2004.
293. Zuo Shuqing WX, Sun Peiyuan, Zhang Panhe, Wang Bingcai, Tang Fang, Dun Zhe, Cai Wei, Liu Qi, Jiang Jiafu, Cao Wuchun. Study on risk factors of hantaviruse infection in rodents from Haidian District of Bejjing. *Chinese Tropical Medicine* 2004.
294. Hu Jingshe LX, Xia Hong, Guan Zengzhi. Investigation on epidemic focus and virus isolation of hemorrhagic fever with rel syndrome in Beijing. *Beijing Medicine* 1990; **12**(6): 4.
295. Yan Qixin YG, Wang Lishun. Isolation of Epidemic Hemorrhagic Fever Virus from Rattus norvegicus. *Hun Medicine* 1988; (2).
296. Yin Liqun WX, Hou Yinghui, Chen Guo, Wang Zhongge. First detection of hemorrhagic fever with rel syndrome virus antigen in the lungs of Rattus norvegicus in Chifeng City. *Medical animal control* 2002; **18**(9): 2.
297. Guo Hengbin XM, Yu Mingming, Wu Guanghua. Rickettsia tsutsutsugamushi isolated from musk shrew. *Chin J Zoonoses* 1992; **8**(006): 51-2.
298. Wang Ying YC, Zhang Hongwei, Shao Bo, Geng Jinpei, Liu Mingjie, Fang Shaoqing, Wang Liguang. Alysis of cases of EHFantibody detected from norvegicus norvegicus. *Chinese Jourl of Frontier Health and Quarantine* 2011; **34**(2): 2.
299. Wang Baoshan HM, Zhu Liping, Zhang Jingqi, Li Qiujuan, Xu Ju. Prelimiry report on the isolation of epidemic hemorrhagic fever virus from brown house mouse. *Chinese Jourl of Epidemiology* 2020.
300. Li Shiqing LX, Pan Liang, Song Liyu. Isolation of epidemic hemorrhagic fever virus from Rattus norvegicus in Fuzhou suburb. *Chinese Jourl of Rodent Control* 1987; (2).
301. Zhou Jihua YW, Pan Hong, Han Qian, Feng Yun, Zhang Yunzhi. Surveillance of hemorrhagic fever with rel syndrome in Yunnan Province, Chi, 2013. *Disease prevention and control notification* 2015; (3): 5.
302. Zhang Ji LY, Yao Lisi, Zhang Lijian, Chen Lei. Alysis on Monitoring Results of Rodents at Changbai Port, Jilin Province, 2014-2015. *Chinese Jourl of Frontier Health and Quarantine* 2015; (S1): 3.
303. Zhou Hui QG, Zhou Xiaoping, Li Ziqiang, Chen Yinong. Alysis of monitoring results of field rodents in Beijing capital airport area. *Chinese Jourl of Frontier Health and Quarantine* 1995.
304. Zhai Wei CY, Li Guoqiang, Pang Liwei, Li Shengfu, Wang Jie. Benxi city 1999-2003 rel syndrome hemorrhagic fever surveillance alysis. *Public Health in China* 2005.
305. Liu Peiqin LH, Fu Jianlin, Hang Changshou, Zhou Jingyi, Wang Jiaobiao, Song Jieyi, Min Weiping,

Qiu Huiling, Zhang Quanfu, Gao Guangzhong, Xiong Shouli, Li Haoran, Zou Xingui, Song Gan. Isolation and antigenicity identification of epidemic hemorrhagic fever virus from rosemary mouse and social mouse. *Acta Acad Med Jiangxi* 1984; (03): 3-8+102-3.
306. Pan Jingtang PS, Yang Renyi, Wang Shibai, He Shaolin, Li Delin. Isolation of Epidemic Hemorrhagic Fever Virus from Apodemus agrarius in Jingzhou Area. *Public Health in China* 1986; (6): 3.
307. Liu Hong LH, Jiang Mingzhou. Simultaneous isolation of Leptospira and hemorrhagic fever with rel syndrome virus from Apodemus agrarius. *Chinese Jourl of Vector Biology and Control* 2002; **13**(4): 294-5.
308. Li Qi LW, Zhang Zuoru, Zhao Hongru, Meng Zongda, Gao Qiuju, Wang Hua, Chen Huaxin, Ye Qing. Isolation of ehf virus from hamster lung. *Chin J Zoonoses* 1995; **11**(002): 0-.
309. Liu Fuqiang CL, Gao Lidong, Zeng Ge, Cai Liang. Alysis of surveillance results of hemorrhagic fever with rel syndrome in Hun Province in 2007. *Practical Preventive Medicine* 2008.
310. Liu Fuqiang GL, Dai Defang, Zhang Hong, Zeng Ge, Guo Shouheng, Wu Zigui, Hu Shixiong. Alysis of the results of surveillance of rel syndrome hemorrhagic fever in Hun Province in 2007. *Chinese Jourl of tural Medicine* 2008.
311. Peng Jinping LF, Dai Defang, Gao Lidong, Zhang Hong, Hu Shixiong, Liu Yunzhi, Li Wei, Wu Zigui. Surveillance of hemorrhagic fever with rel syndrome in Hun Province in 2005. *Practical Preventive Medicine* 2007.
312. Xiang Jinmin YG, Yang Mingrui, Zhu Guangqi, Ni Dashi, Qiu Xuezhao. Immunofluorescence detection of epidemic hemorrhagic fever specific antigen in lung tissue of Apodemus agrarius in epidemic area of Hubei Province. *Jourl of Hubei Medical College* 1998.
313. Yang Mingrui ZG, Yuan Guangming, Zhang Qianhu, Xiao Wen, Zhu Hanrong, Chen Deli, Zhou Tianjie, Xiang Jinmin. Detection and prelimiry detection of hemorrhagic fever antigen in lung tissue of Rattus norvegicus in epidemic area of Hubei Province. *Jourl of Hubei Medical College* 1985.
314. Fanghua W. Study on virus carrying status of host animals of hemorrhagic fever with rel syndrome in Hubei Province. *Jourl of Xiamen University (tural Science Edition)* 2006.
315. Yang Mingrui ZG, Yuan Guangming, Zhang Qianhu, Xiao Wen, Li Zongshan, Zhu Hanrong, Zhou Xinchun, Xiang Jinmin. Investigation on tural host of epidemic hemorrhagic fever virus in Hubei Province. *Jourl of Hubei Medical College* 1985.
316. Zhao Mingjiang LF. Alysis of the results of hemorrhagic fever surveillance for rel syndrome in 2001 in Hubei Province. *Disease surveillance* 2002.
317. Li Junjie YC, Lu Shuheng, Zhao Junxian, Li Huicong, Yang Gang. Research on surveillance of host animals of hemorrhagic fever with rel syndrome in the Hutuo river area of Xian xian *Medical animal control* 2013.
318. Control EDoHLIoEPa. Brief introduction of epidemic situation of epidemic hemorrhagic fever in Hohhot League since 1980. *Chinese Jourl of Epidemiology* 1987.
319. Zhang Fengxian WD, Xu Liangwen, Liu Wenjun. Alysis of hemorrhagic fever surveillance in Hohhot city with rel syndrome. *Chinese Jourl of Vector Biology and Control* 2004.
320. Zhou Jie ZX, Chen Mengshi, Huang Xin, Liu Aizhong, Yang Tubao, Tan Hongzhuan. Epidemiological study on hemorrhagic fever with rel syndrome in flood areas. *Jourl of Central South University (Medical Edition)* 2011.
321. Ruoping J. Surveillance results of epidemic situation of epidemic hemorrhagic fever in Hengshui City from 2004 to 2009. *Occupation and health* 2010; (19): 3.
322. Guan Cheng LF, Li Baolin, Hu Xijing. Seroepidemiological investigation of Apodemus agrarius turally infected with epidemic hemorrhagic fever virus. *Chinese Jourl of Rodent Control* 1986; (4).
323. Jin Tiezhi ZH, Li Jinsong, Ma Chaofeng, Wu Rui, Mi Bao, Wang Kaifeng. Correlation between population structure of Apodemus agrarius and Hantavirus. *Jourl of Biology* 2012; **29**(2): 3.
324. Chen Lufei XJ, Liu Zhongwei, Liu Yancheng, Li Jihong, Sun Rongfang, Sun Chengqun. Alysis of monitoring results of hemorrhagic fever with rel syndrome after flood in Heilongjiang Province. *Chinese Jourl of Vector Biology and Control* 2001; **12**(4): 3.
325. Chengqun S, Lufei C, Yuhua W, et al. Molecular Biological Evidence for the First Discovery of Hantavirus in Heilongjiang Province. *Jourl of Disease Control* 2000; **4**(004): 313-6.
326. Chen Lufei XJ, Liu Zhongwei, Liu Yancheng, Sun Chengqun, Li Jihong, Sun Rongfang. First isolation of domestic rat type hemorrhagic fever with rel syndrome virus from rat lungs in Heilongjiang Province. *Public Health in China* 2001; **17**(4): 1.
327. Wang Fuxing LD, Wang Hua, Yu Tao, Luo Chengwang, Zhao Xiuqin, Zhang Yonghua, Douzhi, Chen Huaxin. Observation on immune effect of ictivated vaccine of hemorrhagic fever with rel syndrome type I in Nongken area of Heilongjiang Province. *Chinese Jourl of Vector Biology and Control* 1996.
328. Chen Lufei CS, Wang Kaili, Zhang Jing, Li Jihong. Isolation and sequence alysis of hantavirus S

gene from Apodemus agrarius in Heilongjiang Province. *Jourl of Virology* 2012; **28**(5): 5.
329. Hu Quanbo CS, Sun Wei, Xu Jun, Hua Hua, Yang Ming, Li Jihong, Zhou Guangen. Monitoring of epidemic situation of host animals of hemorrhagic fever with rel syndrome in Heilongjiang province, Chi, in 2014-2016. *Chinese Jourl of Vector Biology and Control* 2019.
330. Liu Zhongwei XJ, Chen Lufei, Sun Rongfang, Sun Chengqun, Li Jihong, Liu Yancheng. Surveillance and epidemic alysis of host animals of hemorrhagic fever with rel syndrome in disaster and non disaster areas in Heilongjiang Province in 1998. *Disease surveillance* 2000; **15**(003): 100-3.
331. Zuo Shuangyan TK, Li Ying, Yu Jihong, Zhang Yuan, Ni Xuebing, Zheng Yuanchun, Huo Qiubao, Song Yudong, Zeng Xiaomin. DNA detection and sequence alysis of Borrelia burgorferi sensu lato in rodents from Helongjiang forest region. *Chinese Jourl of Epidemiology* 2012; **33**(6): 2.
332. Qu Shuxian HJ, Ji Yusheng. Epidemic alysis and Countermeasures of hemorrhagic fever in Heihe City. *Chinese Jourl of Vector Biology and Control* 2007; **18**(3): 2.
333. Liu Qinglin TS, Zhang Bo. Investigation report on mouse species and EHF Virus in Heihe City in 1991. *Chinese Jourl of Epidemiology* 1992.
334. Gao Peng LH, Liu Qinglin, Yuan Decai. Investigation on mouse species and hemorrhagic fever with rel syndrome virus in Heihe area. *Chinese Jourl of Vector Biology and Control* 1998.
335. Zhao Tieqiang GZ, Gao Yurong, Wang Wenjing, Cui Guoyuan, Liu Yun, Wang Yingjun, Sun Qingzhang, Yu Li, Kang Yingyuan. Prelimiry report on the investigation of norvegicus norvegicus turally carrying EHF Virus Antigen and antibody. *Public Health Magement in Chi* 1987.
336. Wang Wen LX, Guo Wenping, Li Minghui, Zhang Yongzhen. Genetic alysis of hantaviruses carried by Rattus norvegicus collected from Hen and Neimenggu provinces, China. *Chinese Jourl of Epidemiology* 2010; (9): 5.
337. Jiang Jiafu WX, Zuo Shuqing, Wang Riming, Chen Liquan, Wang Bingcai, Dun Zhe, Zhang Panhe, Guo Tianyu, Cao Wuchun. Study on the association between hantavirus infection and Rattus norvegicus. *Chinese Jourl of Epidemiology* 2006.
338. Chen Shuhong PY, Chen Lufei, Liu Di, Liu Yancheng, Yang Ming, Wang Kaili, Zhang Jing, Li Jihong. Alysis of S Gene Characteristics of Rattus norvegicus Hantavirus Isolated from Heilongjiang SC106. *Chinese Jourl of Preventive Veteriry Medicine* 2010.
339. Guo Shuxing WC, Wang Guohan, Wei Rongtai, Ru Zhijian, Li Chunlan, Ma Jiuming, Wang Zhenwu. Serological examition of rat lung antigen and patients in epidemiological epidemic area of Xinye County, Hen Province. *Hen Jourl of Preventive Medicine* 1983.
340. Zeng Guijin WJ, Li Zizhao, Yang Like, Lei Daosheng. Investigation on HFRSV antigen carried by rodents in Hen Province. *Hen Jourl of Preventive Medicine* 1989.
341. Li Linhong WZ, Zhang Yanping, Zeng Guijin, Yu Fuxun, Ren Fang, Shang Siyuan, Zheng Guiming. Comparative study on surveillance of different types of hemorrhagic fever with rel syndrome in Hen Province. *Chinese Jourl of Vector Biology and Control* 2002.
342. Zhang Aimei SJ, Wang Haifeng, Liang Minghui, Wei Haiyan, Li Linhong, Zhang Yanping, Li Xinmin, Zhang Yongzhen, Hao Zongyu. Immunological investigation of hemorrhagic fever with rel syndrome in rodents in hen province. *Chinese Jourl of Health Inspection* 2010.
343. Zhang Zuoru MZ, Lun Jizong, Wang Mingyi, Mo Zhenhai, Yin Yiju, Niu Jianzhang, Ji Tongwen, Gao Huimin, Pang Bozhuang, Yin Wenming. Prelimiry report on epidemiological investigation of hemorrhagic fexueqing with rel syndrome in Hebei Province. *Chinese Jourl of Epidemiology* 1983.
344. Gao GJ NJ, Qi SX, Lu XD, Meng ZD, Zhang ZR. Study on antigen carrying status of epidemic hemorrhagic fever among rats in Hebei Province. *Medical animal control* 1989.
345. Li Qi ZZ, Zhao Hongru, Li Wei, Wang Hua, Chen Huaxin. Study on syndrome typing of hemorrhagic fever with rel syndrome in Hebei Province. *Chinese Jourl of Public Health* 445.
346. Zhang Zuoru ZH, Li Qi, Li Wei, Gao Qiuju, Shan Lijuan. Distribution and serological typing of hemorrhagic fever with rel syndrome in Hebei Province. *Chinese Jourl of Epidemiology* 1996.
347. Zhang Yanbo HZ, Wei Yamei, Xu Yonggang, Qi Shunxiang, Li Qi. The monitoring investigation of hemorrhagic fever with rel syndrome from tural focus in tiol monitoring site of Hebei province. *Chinese Jourl of Vector Biology and Control* 2008.
348. Han Zhanying ZY, Wei Yamei, Han Xu, Xu Yonggang, Qi Shunxiang, Li Qi. Alysis of the surveillance data on host animals in the tiol monitoring sites of hemorrhagic fever with rel syndrome in Hebei province. *Chinese Jourl of Vector Biology and Control* 2011.
349. Lin Z. Epidemiological investigation of epidemic hemorrhagic fever in Xihu District, Hangzhou. *Zhejiang Preventive Medicine* 1999; **11**(9): 3.
350. Liping G. Surveillance and Alysis of Epidemic Hemorrhagic Fever in Handan City from 1998 to 2000. *Disease surveillance* 2002; **17**(5): 2.
351. Huang Haozhuang CY, Kuang Jishen, Wang Min, Chen Chuanqing, Chen Wenzhou, Chen Huaxin, Wang Hua, Tang Qing, Zhang Yonghua, Feng Chonghui, Wu Qinghe, Fu Zhecai. Epidemiological,

serological and etiological investigation of hemorrhagic fever with rel syndrome in Hain Province. *Hain Medicine* 1997; (1): 3.
352. Li Hongyuan ZK, Xing Chengliang, Tong Kuiming, Liu Dianli, Yuan Youhe, Liu Ruizhang. Investigation on storage hosts of epidemic hemorrhagic fever in the suburbs of Harbin. *J Harbin Med Univ* 1984; (3).
353. Gang W. Alysis of species composition and virus carrying rate of rats in hemorrhagic fever with rel syndrome epidemic area in Acheng District, Harbin. *Chinese Jourl of Vector Biology and Control* 2008; **19**(6): 570-1.
354. Huang Xuegui YC, Zhang Anjun, Le Wusheng, Tang Chaozhong, Wang Jianmin, Fu Deqing, Wang Zhaoxiao. Surveillance of host animals in hemorrhagic fever with rel syndrome epidemic area in Zunyi County, Guizhou Province from 1985 to 2000. *Jourl of Disease Control* 2003; **14**(005): 384-5.
355. Huang Xuegui YC, Zhang Anjun, Le Wusheng, Tang Chaozhong, Wang Jianmin, Fu Deqing, Wang Zhaoxiao. Surveillance of host animals of hemorrhagic f ever with rel syndrome in epidemic areas in Zunyi, Guizhou, 1985-1999. *Jourl of Disease Control* 2002.
356. Wang Zhaoxiao FD, Bai Heng, Wang Jianmin, Zhang Xiancai, Zhou Huating, Yang Xiuzhen. Surveillance of host animals of hemorrhagic fever with rel syndrome in Guizhou Province. *Chinese Jourl of Vector Biology and Control* 1996.
357. Wang Zhuan L, Yang Jingyuan, Huang Yan, Tang Guangpeng. Alysis of epidemic situation of hemorrhagic fever with rel syndrome in Guizhou Province from 2005 to 2013. *Medical animal control* 2015.
358. Cai Xinghe WD, Liu Ming, Hu Lijuan, Huang Yanping, Wang Zhaoxiao. Surveillance and alysis of hemorrhagic fever with rel syndrome in Guizhou Province in 1999. *Chinese Jourl of Vector Biology and Control* 2001; **12**(4): 3.
359. Tong Yibing WD, Liu Ming, Hu Lijuan, Wang Zhaoxiao, Zhang Xiancai, Tang Guangpeng, Yang Desheng, Wang Jianmin, Tang Chaozhong. Alysis of hemorrhagic fever surveillance of rel syndrome in Guizhou Province in 1997. *Preventive medicine literature information* 1999.
360. Wang Dingming WZ, Tong Yibing, Liu Ming, Cai Xinghe, Hu Lijuan, Huang Yanping. Surveillance on hemorrhagic fever with rel syndrome in Guizhou during 1984-2000. *Chinese Jourl of Epidemiology* 2003; **24**(8): 3.
361. Yang Xiuzhen KX, Wang Zhaoxiao, Li Chunju, Lu Daqi. Investigation on tural foci of epidemic hemorrhagic fever in Southeast Guizhou. *Chinese Jourl of Rodent Control* 1989; **5**(1): 35-8.
362. Luo Yuexue JQ, Liu Mingqiang, Cai Xinghe. Surveillance and epidemic alysis of host animals of hemorrhagic fever with rel syndrome in Guiyang. *Guangxi Preventive Medicine* 2002; **8**(005): 297-9.
363. Jiang Guowei HY, Wang Baoping, Tan Guoxiang. Investigation on epidemic situation of epidemic hemorrhagic fever in Guiyang. *Chinese Jourl of Vector Biology and Control* 1990; **1**(4): 1.
364. Qiu Jichun LC, Lin Xiangyang, Luo Jianbo, Pan Zhiming, Yang Zhicong, Qiu Guoxiong. Surveillance on hemorrhagic fever with rel syndrome in Guangzhou city from 2001 to 2002. *Chinese Tropical Medicine* 2003; **3**(6): 2.
365. Chen Fang QJ, Lu Enjie, Xiong Yuan, Liang Caiyun, Di Biao. Surveillance on hemorrhagic fever with rel syndrome in Guangzhou city from 2000 to 2003. *Public Health in China* 2004.
366. Xiong Yuan QJ, Liang Caiyun, Gao Yufan, Guo Rongtong, Lin Xiangyang. Alysis of the results of the surveillance of hemorrhagic fever with rel syndrome in Guangzhou in 1999. *Public Health in China* 2002; **18**(3): 2.
367. Qiu Jichun XY, Pan Zhimin, Gao Wenhao, Sun Shuhua, Tang Ximei. Surveillance of hemorrhagic fever with rel syndrome in Guangzhou from 1991 to 1993. *Chinese Jourl of Vector Biology and Control* 1995; **6**(2): 5.
368. Liu Shuguo HG, Zong Yigui. Epidemiological investigation of epidemic hemorrhagic fever in Guangzhou from 1983 to 1984 - Liu Shuguo. *Information on health and epidemic prevention in Guangdong* 1985.
369. Liu Yuan XY, Li Yilan, Jing Qinlong, Jiang Liyun, Cao Yimin, Di Biao, Yang Zhicong. Surveillance and alysis of hemorrhagic fever with rel syndrome in Guangzhou from 2009 to 2012. *Disease monitoring and control* 2013; (11): 2.
370. Zhixiong H, Yinghui C, Chrysolite, Quanming A, Xiaochun H. Report on the First Hemorrhagic Fever with Rel Syndrome in Guangxi. *Shaanxi Medical Jourl* 2020.
371. Hao Ruifeng MQ, Huang Mantao, Song Yigui, Yuan Wenwei, Chen Huaxin. First Investigation Report on Epidemic Foci of Epidemic Hemorrhagic Fever in Guangdong Province. *Guangzhou Pharmaceutical* 1984; (2).
372. Zeng Yanwen XY, Xiong Ying, Gong Tian, Zhou Jun, Shi Yong, Liu Liping. Alysis of surveillance results of hemorrhagic fever with rel syndrome in Gao'an city from 2006 to 2007. *Modern Preventive Medicine* 2009; **36**(16): 3.

373. Wen Jialiang XG. Surveillance of hemorrhagic fever with rel syndrome in Gao'an city in 2000. *Chinese Jourl of Vector Biology and Control* 2002; **13**(1): 74-.
374. Xu Mintian ZF, Zhang Minglei, Feng Xiaochen. Surveillance of hemorrhagic fever with rel syndrome in Ganyu County in 2006. *Jourl of Disease Control* 2008; **12**(3): 292-3.
375. Zhang Minglei WL. Alysis of surveillance results of hemorrhagic fever with rel syndrome in Ganyu County in 2005. *Primary Health Care in China* 2007; **21**(2): 2.
376. Yu Deshan DQ. Prevention and monitoring of rel syndrome hemorrhagic fever in Gansu Province. *Chinese Jourl of Vector Biology and Control* 2003; **14**(1): 2.
377. Yang Shumin BL, Yu Deshan, Dang Qinghui. Investigation and alysis of hemorrhagic fever with rel syndrome in Gansu Province for 11 years. *Chinese Jourl of Vector Biology and Control* 1995; **6**(2): 6.
378. Shi Yanlong GX, Liang Xiaocheng, Yao Chengxiang, Ding Xueliang, Xi Jinxiao, Peng Changjia, Zhang Hong, Zhou Qin, Qu Qingquan. Investigation and alysis of epidemic focus of epidemic hemorrhagic fever in Minxian County, Gansu Province. *Endemic Diseases Bulletin* 1997; **012**(002): 67-9.
379. See Hai Hui WJ, Zhu Liye, Jiang Tao, Tian Yazhen. Alysis of surveillance results of hemorrhagic fever with rel syndrome in Fuyang City from 2006 to 2010. *Anhui Jourl of Preventive Medicine* 2012; **18**(1): 3.
380. Li Zhiyue ZL, Zhang Lijun, Li Hao, Wang Zhenhai. Alysis of surveillance results of hemorrhagic fever with rel syndrome in Fuyang City from 1994 to 1999. *Anhui Jourl of Preventive Medicine* 2000; **6**(3): 2.
381. Zhou Wenwei YG, Huang Lixiong, Xu Suyun, Sun Baochang, Xiao Shusheng, Shi Xiaoqin. It is the first time that social mice and yellow haired mice carry epidemic hemorrhagic fever virus antigen in Zhouning County, Fujian Province. *Public Health in China* 1986; (2): 2.
382. He Simi CY, Wang Linglan, Lin Daihua, Li Shuyang, Chen Liang, Zhou Shuyu, Li Shiqing. Epidemic situation of hemorrhagic fever with rel syndrome and monitoring of host animals in Fujian Province. *Jourl of Strait Preventive Medicine* 2011; (2): 3.
383. Li Xianfeng PL, Li Shiqing, Zong Liyu. Epidemiological investigation of epidemic hemorrhagic fever in Fujian Province. *Chinese Jourl of Rodent Control* 1986.
384. Chen Yang HS, Li Shiqing, Lin Daihua, Li Shuyang, Zhou Shuyu, Chen Liang, Wang Linglan. Surveillance and isolation virus of hemorrhagic fever with rel syndrome at different epidemic areas in Fujian. *Chinese Jourl of Vector Biology and Control* 2009; **20**(4): 3.
385. Chen Yang LD, Chen Liang, Wang Jiaxiong, Han Tengwei, Liu Weijun, Xiao Fangzhen, Lin Wen, Li Shuyang, Deng Yanqin. Surveillance of epidemic and host animals of hemorrhagic fever with rel syndrome in 2013-2015 in Fujian province, China. *Chinese Jourl of Vector Biology and Control* 2016; **27**(6): 3.
386. Chen Yang LD, Chen Liang, Lin Wen, Wang Jiaxiong, Han Tengwei, Niu Jing, Li Shuyang, He Simian, Deng Yanqin. Epidemiological surveillance of hemorrhagic fever with rel syndrome and associated reservoir hosts during 2012 in Fujian province, Chi. *Chinese Jourl of Vector Biology and Control* 2014; **25**(2): 3.
387. He Mei CY, Lin Wen, Wang Jiaxiong, Li Shuyang, Deng Yanqin. Alysis of monitoring results of hemorrhagic fever with rel syndrome in Fujian Province in 2010. *Jourl of Strait Preventive Medicine* 2012; **18**(3): 2.
388. Chen Yang HX, Li Shiqing, Wang Linglan. Epidemiological investigation and alysis of hemorrhagic fever with rel syndrome in Fujian Province in 2005. *Jourl of Strait Preventive Medicine* 2007.
389. Chen Yang HX, Lin Wen, Li Shuyang, Chen Liang, Deng Yanqin. Epidemiological survey alysis of rel syndrome hemorrhagic fever in Fujian Province in 2002. *Chinese Jourl of Vector Biology and Control* 2003; **18**(11): 3.
390. Zhu Changfu YC, Sheng Shaoqin, Jiang Chuanmei. Surveillance and alysis of hemorrhagic fever with rel syndrome in Fengtai County from 1982 to 1993. *Chinese Jourl of Vector Biology and Control* 1995; (2 期): 100-4.
391. Gu Shengquan ZM, Chen Junjie, Dong Bijun, Zou Songting, Xiong Honghe. Investigation of rodents carrying epidemic hemorrhagic fever pathogen in Fengcheng County. *Chinese Jourl of Epidemiology* 2020.
392. Zhang Jinbo WS, Chen Ying. Alysis of epidemic situation of epidemic hemorrhagic fever in Fei County from 1975 to 2000. *Jourl of Strait Preventive Medicine* 2002; **8**(6): 2.
393. Tang Liuying CH, Zou Xueyi, Luo Chengwang, Yu Tao, Dong Junjie, Kang Fengchun, Zhao Linying. Investigation on the ture of tural foci of hemorrhagic fever with rel syndrome in Dayangshu Town, Oroqen Autonomous Banner. *Chin J Zoonoses* 2000; **16**(6): 98-9.
394. Huang Peng YZ, Liu Yuan, Yao Pingping, Hu Jianli, Wang Xiaochen, Yu Jianjia, Li Jun, Han Yaping, Jin Ke, Yang Long, Zhang Yun, Yue Ming. The molecular characteristics and epidemiological alysis of

Hantavirus in southeast coastal area of China from 1980 to 2015. *Chinese Jourl of Vector Biology and Control* 2017; **28**(4): 5.
395. Gao Yu YS, Zhang Ping, Chen Jun, Hong Jiang. Hemorrhagic fever with rel syndrome in Dandong Fengcheng city and rodent poison case investigation. *Medical animal control* 2012.
396. Wang Jianbo B, Li Chunfu, Zhu Jianhua. Surveillance alysis of rel syndrome hemorrhagic fever epidemic in Daxinganling forest area in 1998. *Public Health in China* 1999; **15**(007): 644-.
397. Wang Hongxia YL, Wang Yu, Chen Huaxin, Zhang Yongzhen, Xu Jianguo. Isolation of Hantavirus from Apodemus Peninsulae and Alysis of Its S Fragment. *Chinese Jourl of Vector Biology and Control* 2005; **16**(2): 103-5.
398. Mi Zhuqing ZH, Zhang Yunzhi, Yang Weihong, Wang Jinglin, Zhang Yuzhen, Yuan Qinghong. Investigation on rodents turally infected with hemorrhagic fever with rel syndrome virus in Dali City in 2001. *Medical animal control* 2003; **19**(10): 2.
399. Longgen W. Alysis of 10-year surveillance of hemorrhagic fever with rel syndrome in Dafeng City. *Jiangsu Preventive Medicine* 2001; **12**(1): 2.
400. Jingang H. Alysis of surveillance results of hemorrhagic fever with rel syndrome in Dafeng City in 1998. *Public Health in China* 1999; **15**(7).
401. Su Chengqin CY, Wu Yilun, Zhang Ronggan, He Li, Zi Dengyun, Zhang Hailin, Shi Huafang, Zhu Zhuqing. Epidemic hemorrhagic fever virus antigen was found in the body of Yunn velvet mouse in the west of Yunn Province. *Anhui Medicine* 1986; (01): 40-2.
402. Lu Mingrui YJ, Xu Li, Zhu Hanping, Wang Shensong, Zhu Xiande, Jiang Qingchun. Investigation on the first outbreak of epidemic hemorrhagic fever in Dabie Mountain Pass. *Chinese Jourl of Epidemiology* 1987.
403. Yan Dongyou XY, Zhang Chuan'an, Zheng Guoying, Zhang Shanggui. Hantavirus was isolated from Rattus norvegicus and Rattus flavipectus in Sichuan Province for the first time. *Chinese Jourl of Epidemiology* 2020.
404. Zhang Chengwen JK. Epidemic hemorrhagic fever virus isolated from Rattus norvegicus in Hanzhong, Shaanxi Province. *Shaanxi Medical Jourl* 1991; **20**(5): 4.
405. Liu Xueli FE, Han Qi. Epidemic Hemorrhagic Fever Epidemic Area in Heilongjiang Province. *Chinese Jourl of Epidemiology* 2020.
406. Gao Yunxia LX, Fang Shengfan, Yan Jie, Huang Li, Zhang Xianguang, Fang Shuchun, Deng Jing, Ding Guoyun, Huang Jicheng. Investigation on Yersinia pestis and hantavirus infection among rodent animal in Guangdong port. *Chinese Jourl of Vector Biology and Control* 2016; **27**(2): 4.
407. Yun Z, Faqing L, Jianzhong S, et al. Discovery and Identification of the Yuanjiang Type of Leptospira Sero Group. *Practical Preventive Medicine* 1985.
408. Zhang Yun LF, Shen Jianzhong, Bao Mingrong, Deng Xiaozhao, Zhao Xuezhong, Wu Guanghua. Investigation on tural infection of epidemic hemorrhagic fever virus by gamasid mites. *Jiangsu Medicine* 1985.
409. Xie Wenqiu YG, Chen Jianhua, Xu Suyun. Comparison of EHF focus types and detection rates of mouse antigens and antibodies in Ningde area, Fujian Province. *Chin J Zoonoses* 1987; **3**(04): 57-.
410. Lu Aitao BX, Song Zhuangzhi, Han Song, Huo Lixia, Liu Baolong. Surveillance alysis of epidemic hemorrhagic fever among rats in Inner Mongolia from 2008 to 2009. *Disease monitoring and control* 2010; (12): 2.
411. Song Bingjian MZ, Yang Benxing, Dai Ruiqun, Cheng Chuanji, Gui Fengxin. Investigation on species composition and trichinella spiralis infection of residential rats in nyang city. *Medical animal control* 1992.
412. key H. Surveillance and alysis of epidemic situation of hemorrhagic fever with rel syndrome in nchong City in 1998. *Public Health in China* 1999; **15**(7): 652-3.
413. Fu Renlong CSe, Xia Jinlian, Liu Xiaoqing, Wu Yayun, Zheng Weiqing. An alysis of surveillance results of host animals of hemorrhagic fever with rel syndrome from 2009 to 2016 in Anyi county, nchang, Jiangxi province, Chi. *Chinese Jourl of Vector Biology and Control* 2019; (2): 3.
414. Liu Jiusheng WG, Yuan Ziping, Lu Chengming. Investigation on mouse species and epidemic hemorrhagic fever virus at Lijiazhuang port, nchang. *Chinese Jourl of Frontier Health and Quarantine* 1996; **19**(6): 3.
415. Cai Qiuyun ZX, Zhang Xin. Investigation on host animals of epidemic hemorrhagic fever in Mudanjiang City. *Medical animal control* 1999.
416. Qu Qingquan ZY, Zhou Qin. Surveillance report of epidemic hemorrhagic fever in Min county from 1992 to 1995. *Disease surveillance* 1993.
417. Xue Zhenya LY. Epidemiological investigation on local outbreak of epidemic hemorrhagic fever in Guodian Township, Mengcheng County. *Anhui Medicine* 1985.
418. Liu Hong ZY. Investigation on host animals of epidemic hemorrhagic fever in Meihekou City.

Modern drug application in China 2009.
419. Li Xiaosong PQ, Fan Rusheng, Huang Qin, Zhang You. Alysis of surveillance results of epidemic hemorrhagic fever in Maoming port area. *Chinese Jourl of Frontier Health and Quarantine* 2005.
420. You Chuanxin XZ. Ten year epidemiological surveillance and control of house mouse hemorrhagic fever in Luoyang area. *Hen Jourl of Preventive Medicine* 1992.
421. Mei Shenghua WM, Li Zhaomei, Zhong Xiujuan. Longquan City 2008-2010 rel syndrome hemorrhagic fever surveillance alysis. *Zhejiang Preventive Medicine* 2012.
422. Gu Xianshi MG, Song Zongbi, Peng Langtao, You Zhongqiong, Su Qiang, Gu Ti, Yang Shiquan. Five year investigation of hantavirus infection in small mammals and healthy people in epidemic hemorrhagic fever area. *Jourl of Preventive Medicine Information* 1994; **10**(4): 211-3.
423. Lan Mingyang MY, Zhou Hongfu, Han Zhixin, Tao Buzhi. Investigation on the toxicity of mice and mites in epidemic area of epidemic hemorrhagic fever. *Acta Acad Med Suzhou* 2020.
424. Qu Jingyi LB, Sheng Mid Autumn Festival. Study on the toxicity of mice in epidemic and non epidemic areas of epidemic hemorrhagic fever. *Shandong Medicine* 1983.
425. Li Xianfeng PL, Sun Baochang, Yuan Gaolin, Huang Lixiong, Zhou Wenwei, Huang Guisen, Shi Xiaoqin, Xiao Shusheng, Xu Suyun. A new infectious source of epidemic hemorrhagic fever -- the discovery of social rats. *Fujian Medical Jourl* 1985.
426. Lan Mingyang ZH, Meng Yangchun. Investigation on virus carrying wild rats in two epidemic areas of epidemic hemorrhagic fever. *Jiangsu Medicine* 1983; (08): 18-9.
427. Chen Huaxin TL, Bai Xiaomeng, Chen Lufei, He Shaowen, Wan Fanghua, Long Qingzhong, Liu Hong, Hu Meijiao, Liu Guangzhong. Report on surveillance data in surveillance spots on epidemic hemorrhagic f ever in some areas. *Chinese Jourl of Epidemiology* 1999; (06): 368-72.
428. Wang Yuanyuan CZ, Qi Xinxuan, Li Ke, Ma Yingji, Zhang Bao, Cao Jie. Relationship between incidence rate and incidence of Apodemus Apodemus infection in epidemic hemorrhagic fever. *J Harbin Med Univ* 1985.
429. Ye Kelong DL, Tian Meiying, Zhou Liandi, Li Yongliang, Xu Zhiyi. Comparative alysis of host infection characteristics of epidemic hemorrhagic fever animals. *Chinese Jourl of Public Health* 1993; **12**(6): 3.
430. Du Wei LP, Yu Qingyun. Study on infectious source of epidemic hemorrhagic fever. *Jourl of Taishan Medical College* 1996; **17**(3): 3.
431. Lin Fuxi YW, Cao Xiliang. Prelimiry study on the distribution law of epidemic hemorrhagic fever in different terrain. *Chinese Jourl of Epidemiology* 1989; **10**(1): 3.
432. Yang Zhanqing MX, Yu Xiaomin, Liu Yunxi, Gao Xueyun, Lu Ziyu, Zhang Fubo, Wang Jinfeng, Liu Jingshan. Study on the transmission mode of epidemic hemorrhagic fever virus among host animals and its epidemiological significance. *Chinese Jourl of Epidemiology* 2020.
433. Li Hongyuan ZK, Xing Chengliang, Tong Kuiming, Liu Dianli, Yuan Youhe, Liu Ruizhang. Localization of epidemic hemorrhagic fever virus antigen in hamsters. *J Harbin Med Univ* 1984; (3).
434. Jia Chengjian GZ. Epidemic characteristics of epidemic hemorrhagic fever in Lingbao City from 1996 to 2005. *Occupation and health* 2007; **23**(9): 731-2.
435. Yang Yongbiao LY, Zhang Yonghua, Ma Xueyun, Zhuang Qianshan. Epidemiological investigation of epidemic hemorrhagic fever in Linyi City. *PLA Jourl of Preventive Medicine* 1995; **13**(1): 3.
436. Li Xingzong WS. Alysis of hemorrhagic fever surveillance results of rel syndrome in Lanshan District, Linyi City. *Chinese Jourl of Vector Biology and Control* 2005; **16**(1): 78-.
437. Yang Yongbiao WX. A Study on the Prevention of Hemorrhagic Fever with Rel Syndrome (HFRS)in Hedong District of Linyi City. *Chinese Jourl of Vector Biology and Control* 2002.
438. Ordiry ZJ, Kang Xiping, Yu Suzhen, Zhuang Xinhua, Shao Changgui, Li Shiwei, Du Lixiao. Monitoring study of hemorrhagic fever with rel syndrome in Linyi City in 1991_1997. *Jourl of Linyi Medical College* 1998.
439. Yang Yongbiao SX, Ma Xueyun, Ai Maobo. Dymic alysis of epidemic hemorrhagic fever in Linyi City from 1973 to 1992. *Jourl of Linyi Medical College* 1994.
440. Wu Qiao YN, Ma Lijun. Prelimiry examition of mice carrying epidemic hemorrhagic fever virus antigen in Linyi area. *Chinese Jourl of Epidemiology* 2020.
441. Shengjie C. Surveillance and alysis of hemorrhagic fever with rel syndrome in Lintan county from 1994 to 1998. *Disease surveillance* 1999.
442. Jiang Mingjun WYa, Wu Lifeng. Surveillance and alysis of hemorrhagic fever with rel syndrome in Linshu County. *Medical animal control* 1999.
443. Wang Fuyou DY. Epidemiological investigation and alysis of epidemic hemorrhagic fever in Linshu county from 1991 to 2000. *Occupation and health* 2004.
444. Diansong P. Epidemiological investigation of epidemic hemorrhagic fever in Linshu county from 1990 to 1999. *Medical animal control* 1991.

445. Wang Fuyou DY. Alysis of the epidemiological dymics of rel syndrome hemorrhagic fever in Linshu County in 1973_1997. *Disease surveillance* 1999.
446. Jianren X. Investigation on epidemic focus of epidemic hemorrhagic fever in genglou Township, Linhai City. *Jourl of Strait Preventive Medicine* 630.
447. Jianren X. Investigation report on EHF focus in Datian Town, Linhai City in 1997. *Medical animal control* 1998.
448. Qin Caiming LY, Yao Wenqing, Sun Yingwei, Han Yanghuan, Zhang Jibo. Comparison of pathogenic and molecular biological detection of hemorrhagic fever with rel syndrome in rats in Liaoning Province. *Chinese Jourl of Zoonoses* 2010; **26**(06): 528-31.
449. Liu Dexing HY, Wang Zhiqing, Hu Lingmei. Investigation on the first outbreak of epidemic hemorrhagic fever in western Liaoning. *PLA Jourl of Preventive Medicine* 1990.
450. Zhang Jie WZ, Li Xin, Liu Xuesheng, Liu Yun, Sun Yingwei, Yao Wenqing. An alysis of the surveillance results of hemorrhagic fever with rel syndrome in Liaoning province, Chi, 2012-2016. *Chinese Jourl of Vector Biology and Control* 2019.
451. Dong Xue ZY, Li Xin, Zhao Changzhi, Wang Bing. Genotyping of hantavirus isolates from Liaoning Province. *Chin Exp Clin Virol* 2005.
452. She Yushao WL. Surveillance of epidemic hemorrhagic fever in Liwan District from 1983 to 1990. *Hygiene and epidemic prevention in Guangdong* 1992.
453. Ran Longshuang LP, Luo Zuquan. Investigation on epidemic hemorrhagic tropical toxicity of rodents in Duting Town, Lichuan County. *Jourl of enshi medical college* 1986.
454. Huang Jinbo LY, Liu Fuming, Pan Xiaomeng. Monitoring results of hemorrhagic fever with rel syndrome in Lishui City. *Zhejiang Preventive Medicine* 2015; **27**(2): 3.
455. Wang Peiwei ZL. Investigation on tural foci of epidemic hemorrhagic fever in Yueqing City. *Zhejiang Preventive Medicine* 1998; **10**(3): 2.
456. Yang Youcai WY, Xu Xuewei, Ren Cizao, Huang Deming, Liu Hong. Investigation on epidemic focus of epidemic hemorrhagic fever in Langya Mountain Area. *Anhui Jourl of Preventive Medicine* 1997.
457. Zhang Xiancai ZX, Tang Gangquan, Tang Qianlong, Liu Mingqiang, Wang Zhaoxiao. Nine year surveillance of epidemic hemorrhagic fever in Kaiyang County. *Jourl of Preventive Medicine Information* 1993; (S1): 1.
458. Liu Dengquan LZ, Deng Xuejin, Liu Qiqiong, Liu Xuecheng, Zhang Jiake. Investigation on host animals of hemorrhagic fever with rel syndrome in Kaijiang County. *Modern Preventive Medicine* 2000; **27**(4): 3.
459. Liu Dengquan LZ, Deng Xuejin, Liu Qiqiong, Liu Xuecheng, Zhang Jiake. Surveillance report of hemorrhagic fever with rel syndrome in Kaijiang County in 1998. *Public Health in China* 1999; **15**(7): 653-.
460. Gao Yi ZG. Surveillance of hemorrhagic fever with rel syndrome in Kaihua County from 1984 to 2001. *Zhejiang Preventive Medicine* 2004.
461. Huang Baotong ZZ, Liu Xuezhen, Zhao Shili, Hu Guangying, Wang Luozhong, Zhu Kongli, Cao Dianliang. Epidemiological investigation of epidemic hemorrhagic fever in Jun County. *Chinese Jourl of Epidemiology* 1994; **02**: 102-.
462. He Pishan MY, Li Xuegang, Zhang Juntang. Alysis of Surveillance Data of Hemorrhagic Fever with Rel Syndrome in Jun County from 1975 to 1998. *Jourl of Linyi Medical College* 1999; (02): 22-4.
463. Wenbin J. Comprehensive alysis of monitoring, prevention and treatment of hemorrhagic fever with rel syndrome in Jiujiang City. *Jiujiang Medicine* 2000; **15**(3): 2.
464. Wenbin J. Alysis of epidemic status of epidemic hemorrhagic fever in Jiujiang City from 1961 to 1984. *Public Health in China* 1987; (5): 1.
465. Peng Shenghong WX. Investigation on the distribution and epidemic factors of epidemic hemorrhagic fever in Jingzhou area. *Chinese Jourl of Epidemiology* 2020.
466. Wang Yinlei YY. Alysis of surveillance results of hemorrhagic fever with rel syndrome in Jinjiang City from 2011 to 2012. *Jourl of Strait Preventive Medicine* 2013; (6): 2.
467. Wang Wenjing ZT, Jiang Chengwen. Investigation report on the outbreak of house mouse epidemic hemorrhagic fever in Jinzhou coastal area. *Chinese Jourl of Epidemiology* 2020.
468. Wei Longjiang XK, Zhou Baoyu. Alysis of epidemic characteristics of epidemic hemorrhagic fever in Jinzhou area from 1993 to 1998. *Medical animal control* 2000; **16**(3): 144-6.
469. Zhu Kuangji WQ. Alysis of epidemic situation of epidemic hemorrhagic fever in Jinhua County from 1966 to 1997. *Zhejiang Preventive Medicine* 1999.
470. Chen Zhiqing PZ. Alysis of hemorrhagic fever surveillance results in 2011-2012 in Jinhua City with inter-rat rel syndrome. *Chinese Jourl of Preventive Medicine* 2014; (8): 3.
471. Yang Nianzhong JQ. Study on epidemic focus of epidemic hemorrhagic fever in Jiaojiang City. *Zhejiang Preventive Medicine and Disease Surveillance* 1991; (6): 4.

472. Nianzhong Y. Study on hemorrhagic fever in Jiaojiang area from 1980 to 2000. *Chinese Jourl of Vector Biology and Control* 2002; **13**(6): 2.
473. Liu Shiwen GT, Xu Gang, Shi Yong, Li Jianxiong, Zhang Yanni, Zhou Jun, Liu Xiaoqing, Xiao Fang, Xiong Ying. Whole genome sequence alysis of Hantaan virus strain ayw89-15 isolated from Jiangxi Province. *Chinese Jourl of Zoonoses* 2017; **33**(12): 5.
474. Wei L. Epidemic hemorrhagic fever virus isolated from rodents and social rodents in Jiangxi Province. *Medical Research Communication* 1985.
475. Yu Ping CH, Xiong Ying, Xie Yun, Yuan Hui. Surveillance on hemorrhagic fever with rel syndrome in Jiangxi province from 2008 to 2012. *Modern Preventive Medicine* 2014; **41**(13): 4.
476. Zu Rongqiang WY, Zhu Fengcai, Liu Guangzhong. Study on surveillance and epidemic prediction index of hemorrhagic fever with rel syndrome in Jiangsu Province. *Chinese Jourl of Epidemiology* 1999; **20**(4): 220-3.
477. Lan Mingyang MY, Zhou Hongfu, Han Zhixin, Tao Buzhi. Host detection and virus isolation of epidemic hemorrhagic fever in Jiangsu Province. *Acta Acad Med Suzhou* 1985.
478. Shi Ping JZ, Liu Guangzhong. Epidemiological surveillance of hemorrhagic fever with rel syndrome in Jiangsu Province from 2001 to 2009. *Chinese Jourl of Vector Biology and Control* 1995; **6**(2): 3.
479. Wu Yangsheng ZF, Jin Zhikuan, Zu Rongqiang, Liu Guangzhong. Epidemiological surveillance of hemorrhagic fever with rel syndrome in Jiangsu Province from 2001 to 2008. *Chinese Jourl of Vector Biology and Control* 1997; **8**(3): 2.
480. Wu Bin WS, Meng Fanyue, Li Liang, Qi Xian, Zu Rongqiang, Tang Fenyang. Epidemiological surveillance of hemorrhagic fever with rel syndrome in Jiangsu Province from 2001 to 2007. *Jiangsu Preventive Medicine* 2010; **21**(4): 3.
481. Meng Fanyue WY, Li Liang, Zu Rongqiang, Zhu Fengcai. Epidemiological surveillance on hemorrhagic fever with rel syndrome in Jiangsu province from2001to 2006. *Jiangsu Preventive Medicine* 2008.
482. Zu Rongqiang WY, Zhu Fengcai, Liu Guangzhong. Surveillance of hemorrhagic fever with rel syndrome in Jiangsu Province from 1996 to 2000. *Chinese Jourl of Epidemiology* 2003; **24**(2): 5.
483. Li October GY, Mo Youmei, Yang Mingrui, Chen Xiangyi, Wang Guiqing, Yan Hong. Studies on Epidemic Hemorrhagic Fever among Rats in Jianghan Plian, Hubei Province, Chi. *Chinese Jourl of Preventive Medicine* 1997; **31**(3): 3.
484. Zhang Yourong HH. EHF virus antigen was detected from rosette grey muskrat for the first time in Jiande county. *Zhejiang Disease Surveillance* 1989; (S3): 3-.
485. Li Hongyuan ZK, Xing Chengliang, Tong Kuiming, Liu Dianli, Yuan Youhe, Liu Ruizhang. Investigation on Infectious Source of Epidemic Hemorrhagic Fever in Rat Area. *J Harbin Med Univ* 2000; **16**(3): 4.
486. Zhao Fuhe LJ. An Alysis of the Results on the HFRSV tural Infection in Rodents for f ourteen years in Jining. *Chinese Jourl of Vector Biology and Control* 2002; **13**(4): 288-90.
487. Xu Dejiang GS, Ren Jinjing. Alysis of rel syndrome hemorrhagic fever surveillance in Jin railroad area in 1984_2001. *Preventive medicine literature information* 2002; **8**(5): 1.
488. Cai Zhenxi WM. Epidemiological investigation of EHF in Ji'an City. *Chinese Jourl of Frontier Health and Quarantine* 1992; (6): 2.
489. Yongfa X. Surveillance of host animals and alysis of human epidemic situation of hemorrhagic fever with rel syndrome in Ji'an City from 2003 to 2007. *Primary Health Care in China* 2008; (12): 47-8.
490. Liu Jiangqiu FY, Cai Zenglin, Xie Zhigang, Zhao Zhanlin, Lu Zhixin. Investigation on antigen and antibody of hemorrhagic fever with rel syndrome carried by wild rats in Changbai Mountain Area of Jilin Province. *Shenyang Military Medicine* 2001; (1): 2.
491. Mu Cuiying Z, Ma Changhong, Luo Gongjun, Shao Lijun. Investigation on rodents and pathogen detection report at Hunchun railway port. *Chinese Jourl of Frontier Health and Quarantine* 2015; (S1): 1.
492. Zhang Yun ZX, Zhang Binggen, Shen Jianzhong, Tang Jiaqi, Bao Mingrong, Wu Guanghua. Investigation on Grey musk shrew as host animal of epidemic hemorrhagic fever. *Public Health in China* 1987.
493. Senior high school promotion WZ, Fang Hong, Jin Suzheng. Investigation on epidemic hemorrhagic fever in Huangyan Area. *Shanghai Jourl of Preventive Medicine* 1993; (3).
494. Zheng Guiming LX, Yao Hansong, Wei Duanxian. Investigation on infectious sources of epidemic hemorrhagic fever in Huaiyang County. *Hen Jourl of Preventive Medicine* 1986; (2).
495. Yi Guangsheng CY, Chen Zhenya, Yi Yan, Li Min, Wei Minmin. Investigation on the composition of rodent species and the status of epidemic hemorrhagic tropical toxin in Huain Coal Mine. *Anhui Jourl*

of Preventive Medicine 1997.
496. Guan Jinyu ZW, Zheng Xiaonong. Investigation on tural foci of hemorrhagic fever with rel syndrome in Huating County. *Medical animal control* 2008; **24**(002): 125-6.
497. Dang Qinghui GJ, Zhu Wait, Ma Jiming, Yan Jun, Li Guotai. Epidemiological characteristics of hemorrhagic fever with rel syndrome in Huating country. *Chinese Jourl of tural Medicine* 2010; (6): 464-6.
498. Dai Defang ZH, Liu Yunzhi, Liu Fuqiang, Li Fangcai, Gao Lidong, Wu Zigui, Li Wei. Monitoring study on hemorrhagic fever with r el syndrome (HFRS) in Hun Province. *Disease surveillance* 2007; **22**(002): 78-82.
499. Fu Jiarong LX, Wang Chuyin, Hu Jianzhong, Yuan Xiwen, Long Qingzhong, Li Wei, Hu Yuanhao. Surveillance of hemorrhagic fever with rel syndrome in Ningxiang County, Hun Province from 1991 to 2002. *Chinese Jourl of Epidemiology* 2003; **24**(8): 751-.
500. He Qing HJ, Liu Fuqiang, He Qinglong. An investigation on an outbreak of hemorrhagic fever with rel syndrome in Dao county, Hun province. *Chinese Jourl of tural Medicine* 2010; (1): 30-2.
501. Hu Manxia ZS, Wang Junyong, Yang Jun, Hu Deqiang, Mou Baiyu. Investigation Report on IgG Antibody of Rodent Epidemic Hemorrhagic Fever in the Area Near Suifenhe Highway Port. *Chinese Jourl of Frontier Health and Quarantine* 2001.
502. Cong Meili GW, Wang Jianbo, Wang Wen, Zhou Runhong, Li Minghui, Zhang Junong, Zhang Yongzhen. Genetic characteristies of hantaviruses crried by Microtus maximowixzii in Yakesbi of Inner Mongolia China. *Chinese Jourl of Epidemiology* 2012; **33**(8): 4.
503. Holmes EC, Zhang YZ. The evolution and emergence of hantaviruses. *Curr Opin Virol* 2015; **10**: 27-33.
504. Yanagihara R, Gu SH, Arai S, Kang HJ, Song JW. Hantaviruses: rediscovery and new beginnings. *Virus Res* 2014; **187**: 6-14.
505. Zhang Y, Yuan J, Yang X, et al. A novel hantavirus detected in Yunnan red-backed vole (Eothenomys miletus) in China. *J Gen Virol* 2011; **92**(Pt 6): 1454-7.
506. Wang Jinglin ZH, Zhou Jihua, Yang Weihong, Zhang Yuzhen, Mi Zhuqing, Rode Rong, Yang Jiao, Yang Ligui, Zhao Wusheng, Zhang Pinyuan, Li Huachang, Li Wei, Lu Tao, Zhang Xiliang. Tula like hantavirus was detected in Chinese rats for the first time. *Chinese Jourl of Zoonoses* 2010; **26**(5): 5.
507. Cong ML, Guo WP, Wang JB, et al. [Genetic characteristics of hantaviruses carried by Microtus maximowixzii in Yakeshi of Inner Mongolia, China]. *Zhonghua Liu Xing Bing Xue Za Zhi* 2012; **33**(8): 832-5.
508. Pengfei Y, Yang Z, Huaxin C, Yongzhen Z. Advances in Molecular Epidemiology of Pumalaviruses. *Chinese Journal of Epidemiology* 2008.
509. Wu Yanping HB, Hu Guangwei, Yao Laishun, Gou Weimin, Feng Guodong, Jin Longzhe, Cui Qingjin, Ma Li, Zhang Qiang. Alysis of epidemic trend of hemorrhagic fever with rel syndrome in Jilin Province. *Chinese Jourl of Endemic Disease Control* 2004; **19**(3): 2.
510. Liu Gang LC, Hu Guangwei, Li Yue, Yao Laishun, Chen Yuqing, Huang Biao, Ren Ming, Chen Yunzhi, Guan Shixin, Yu Chuanyou, Baozhong, Zhong Xiangdong, Sun Yuexin, Li Wenwen, Li Dexin. Pumala hantavirus found in China. *Chin Exp Clin Virol* 2003; **17**(1): 3.
511. Wu Donglin WH, Shen Bo, Hu Guangwei, Li Dexin. RT-PCR detection and sequence alysis of pumala virus in Jilin Province. *Chi Health Engineering* 2008.
512. Geng Yingzhi YW, Liu Yun, Sun Yingwei, Wang Bo, Han Yanghuan, Li Xin, Chen Jingyi, Zhao Zhuo. Detection and gene characteristics of pumala virus in Liaoning Province. *Chinese Jourl of Pathogenic Biology* 2012; **007**(006): 426-8.
513. Tang Lihua ZQ, Xiu Meihong, Hu Guangwei, Shen Bo, Yang Xianda, Liang Mifang, Li Dexin. Discovery of a new subtype of pumala virus in China. *Jourl of Virology* 2007; **23**(4): 6.
514. Liu G. Molecular biology of pumara virus and its diagnosis. 2004.
515. Xian C. Isolation and evolution of hantavirus in jilin province: Sichuan University; 2005.
516. Lihua T, Quanfu Z, red X, et al. Discovery of a new subtype of pumara virus in china. *Journal of Virology* 2007; **23**(4): 6.
517. Iashina L, Slonova R, Oleĭnik O, et al. A new genetic variant of the PUUV virus from the Maritime Territory and its natural carrier red-grey vole Clethrionomys rufocanus. 2004; **49**(6): 34-7.
518. Shu-Yuan X, Spik KW, Li D, Schmaljohn CSJVr. Nucleotide and deduced amino acid sequences of the M and S genome segments of two Puumala virus isolates from Russia. 1993; **30**(1): 97-103.
519. Plyusnin A, Vapalahti O, Lehväslaiho H, et al. Genetic variation of wild Puumala viruses within the serotype, local rodent populations and individual animal. 1995; **38**(1): 25-41.
520. Juto P, Elgh F, Ahlm C, et al. The first human isolate of Puumala virus in Scandinavia as cultured from phytohemagglutinin stimulated leucocytes. 1997; **53**(2): 150-6.
521. Abu Daud NH, Kariwa H, Tkachenko E, et al. Genetic and antigenic analyses of a Puumala virus

isolate as a potential vaccine strain. 2008; **56**(3): 151-65.
522. Escutenaire S, Chalon P, Heyman P, et al. Genetic characterization of Puumala hantavirus strains from Belgium: evidence for a distinct phylogenetic lineage. 2001; **74**(1-2): 1-15.
523. Asikainen K, Hänninen T, Henttonen H, et al. Molecular evolution of Puumala hantavirus in Fennoscandia: phylogenetic analysis of strains from two recolonization routes, Karelia and Denmark. 2000; **81**(12): 2833-41.
524. Lundkvist Å, Wiger D, Hörling J, et al. Isolation and characterization of Puumala hantavirus from Norway: evidence for a distinct phylogenetic sublineage. 1998; **79**(11): 2603-14.
525. Lundkvist A, Cheng Y, Sjölander KB, Niklasson B, Vaheri A, Plyusnin AJJov. Cell culture adaptation of Puumala hantavirus changes the infectivity for its natural reservoir, Clethrionomys glareolus, and leads to accumulation of mutants with altered genomic RNA S segment. 1997; **71**(12): 9515-23.
526. Dekonenko A, Yakimenko V, Ivanov A, et al. Genetic similarity of Puumala viruses found in Finland and western Siberia and of the mitochondrial DNA of their rodent hosts suggests a common evolutionary origin. 2003; **3**(4): 245-57.
527. Johansson P, Olsson M, Lindgren L, et al. Complete gene sequence of a human Puumala hantavirus isolate, Puumala Umeå/hu: sequence comparison and characterisation of encoded gene products. 2004; **105**(2): 147-55.
528. Razzauti M, Plyusnina A, Sironen T, Henttonen H, Plyusnin AJJoGV. Analysis of Puumala hantavirus in a bank vole population in northern Finland: evidence for co-circulation of two genetic lineages and frequent reassortment between strains. 2009; **90**(8): 1923-31.
529. Plyusnin A, Vapalahti O, Ulfves K, et al. Sequences of wild Puumala virus genes show a correlation of genetic variation with geographic origin of the strains. 1994; **75**(2): 405-9.
530. Yashina LN, Abramov SA, Dupal TA, et al. Hokkaido genotype of Puumala virus in the grey red-backed vole (Myodes rufocanus) and northern red-backed vole (Myodes rutilus) in Siberia. *Infect Genet Evol* 2015; **33**: 304-13.
531. Nemirov K, Lundkvist A, Vaheri A, Plyusnin A. Adaptation of Puumala hantavirus to cell culture is associated with point mutations in the coding region of the L segment and in the noncoding regions of the S segment. *J Virol* 2003; **77**(16): 8793-800.
532. Kramski M, Achazi K, Klempa B, Krüger DH. Nephropathia epidemica with a 6-week incubation period after occupational exposure to Puumala hantavirus. *J Clin Virol* 2009; **44**(1): 99-101.
533. Bahr U, Zeier M, Muranyi W. Characterization of a new Puumala virus genotype associated with hemorrhagic fever with renal syndrome. *Virus Genes* 2006; **33**(2): 229-34.
534. Plyusnin A, Hörling J, Kanerva M, et al. Puumala hantavirus genome in patients with nephropathia epidemica: correlation of PCR positivity with HLA haplotype and link to viral sequences in local rodents. *J Clin Microbiol* 1997; **35**(5): 1090-6.
535. Bowen MD, Kariwa H, Rollin PE, Peters CJ, Nichol ST. Genetic characterization of a human isolate of Puumala hantavirus from France. *Virus Res* 1995; **38**(2-3): 279-89.
536. Klingström J, Heyman P, Escutenaire S, et al. Rodent host specificity of European hantaviruses: evidence of Puumala virus interspecific spillover. *J Med Virol* 2002; **68**(4): 581-8.
537. Avsic-Zupanc T, Petrovec M, Duh D, Plyusnina A, Lundkvist A, Plyusnin A. Puumala hantavirus in Slovenia: analyses of S and M segment sequences recovered from patients and rodents. *Virus Res* 2007; **123**(2): 204-10.
538. Razzauti M, Plyusnina A, Henttonen H, Plyusnin A. Microevolution of Puumala hantavirus during a complete population cycle of its host, the bank vole (Myodes glareolus). *PLoS One* 2013; **8**(5): e64447.
539. Plyusnin A, Mustonen J, Asikainen K, et al. Analysis of puumala hantavirus genome in patients with nephropathia epidemica and rodent carriers from the sites of infection. *J Med Virol* 1999; **59**(3): 397-405.
540. Nemirov K, Leirs H, Lundkvist A, Olsson GE. Puumala hantavirus and Myodes glareolus in northern Europe: no evidence of co-divergence between genetic lineages of virus and host. *J Gen Virol* 2010; **91**(Pt 5): 1262-74.
541. Razzauti M, Plyusnina A, Niemimaa J, Henttonen H, Plyusnin A. Co-circulation of two Puumala hantavirus lineages in Latvia: a Russian lineage described previously and a novel Latvian lineage. *J Med Virol* 2012; **84**(2): 314-8.
542. Tian H, Stenseth NC. The ecological dynamics of hantavirus diseases: From environmental variability to disease prevention largely based on data from China. *PLoS Negl Trop Dis* 2019; **13**(2): e0006901.
543. Tang LH, Zhang QF, Xiu MH, et al. [Identification of a new Puumala hantavirus subtype in rodents from China]. *Bing Du Xue Bao* 2007; **23**(4): 320-5.
544. Plyusnina A, Laakkonen J, Niemimaa J, et al. Genetic analysis of hantaviruses carried by Myodes

and Microtus rodents in Buryatia. *Virol J* 2008; **5**: 4.
545. Liu G, Li C, Hu GW, et al. [Identification of Puumala like viruses in China]. *Zhonghua Shi Yan He Lin Chuang Bing Du Xue Za Zhi* 2003; **17**(1): 55-7.
546. Khan A, Khan AS. Hantaviruses: a tale of two hemispheres. *Panminerva Med* 2003; **45**(1): 43-51.
547. Guo WP, Lin XD, Wang W, et al. A new subtype of Thottapalayam virus carried by the Asian house shrew (Suncus murinus) in China. *Infect Genet Evol* 2011; **11**(8): 1862-7.
548. Chen Liwei YQ, Qiu Deyi, Yang Hanhan, Liu Dexing, He Qiuxing. Investigation on the pathogens carried by rodents at Shenwan port in Zhongshan city. *Chinese Jourl of Frontier Health and Quarantine* 2019; (1): 4.
549. Yang Jun JW, Jiao Dan, Xu Ning, Liang Huijie, Cheng Cheng, Wang Xiaojie, Fu Weiming, Wang Yanmei, Geng Cong. Investigation on rodent pathogens in volcanic lava area of Xunke County, Heilongjiang Province. *Chinese Jourl of Vector Biology and Control* 2015; **26**(003): 238-41.
550. Guo Gang XJ, Huang Lin, Sheng Jinliang, Abdul Zaire. Nucleic acid detection and genotyping of rat hantavirus in xinjiang. *Chinese Jourl of Vector Biology and Control* 2013; (2): 3.
551. Liu Jianjun YF, He Jianfan, Zhang Xiaolan, Liang Zhuon, Zhang Shunxiang, Zhang Hailong, Yang Hong. Study on the molecular characteristic of tural infection of rodents with Hantaviruses in Shenzhen city. *Chinese Jourl of Preventive Medicine* 2008; **42**(5): 5.
552. Zhang Yongzhen XQ, Li Minghui, Zou Yang, Lv Wei, Dai Defang, Chen Huaxin. An epidemiologic investigation of hantaviruses carried by rodent hosts in Hun province. *Chinese Jourl of Epidemiology* 2007; **28**(1): 5.
553. Li Minghui CX, Yang Guoqing, Shen Tiefeng, Liu Bao, Guo Wenping, Zhang Yongzhen. Alysis of hantavirus carried by Rattus norvegicus in residential areas of Huludao City. *Chinese Jourl of Vector Biology and Control* 2011; **22**(3): 4.
554. Yangnu L. Alysis of monitoring results of hemorrhagic fever with rel syndrome in Cixi City from 1996 to 2011. *Chinese Jourl of Vector Biology and Control* 2012.
555. Wang Yinlei YY. Alysis of monitoring results of hemorrhagic fever with rel syndrome in Jinjiang City from 2011 to 2012. *Jourl of Strait Preventive Medicine* 2013; (6): 2.
556. He Xu QC, Wang Chuan. Alysis of rat monitoring results at Lianyungang port from 2014 to 2015. *Port Health Control* 2016; **21**(5): 4.
557. Cao Yingchun GR, Wang Lixia, Li Mi. Detection and alysis of hantavirus antigen in Taon City, Jilin Province in 2013. *Chinese Jourl of Endemic Disease Control* 2015; (4): 1.
558. Wang B, Cai CL, Li B, et al. Detection and characterization of three zoonotic viruses in wild rodents and shrews from Shenzhen city, China. *Virol Sin* 2017; **32**(4): 290-7.
559. He W, Fu J, Wen Y, Cheng M, Mo Y, Chen Q. Detection and Genetic Characterization of Seoul Virus in Liver Tissue Samples From Rattus norvegicus and Rattus tanezumi in Urban Areas of Southern China. *Front Vet Sci* 2021; **8**: 748232.
560. Liu Shiwen XG, Gong Tian, Shi Yong, Li Jianxiong, Liu Xiaoqing, Xiao Fang, Zhang Yanni, Zhou Jun, Xiong Ying. Detection and genotyping of murine hantavirus in nchang. *Modern Preventive Medicine* 2016; **43**(6): 5.
561. Ting G. Epidemic Status of Hemorrhagic Fever with Renal Syndrome in Yunnan Province from 2014 to 2015 and Investigation on Host Animals in Some Areas. *Dali University* 2016.
562. Hu Tingsong HQ, Li Shaoxiong, Huang Yong, Hu Haimei, Gao Lifen, Luo Qiongmei, He Yongzhi, Zhou Yongcun, Zhang Hailin, Zhang Fuqiang. Epidemiological characteristics and host animals of hemorrhagic fever with rel syndrome in Chuxiong City, Yunn Province from 2015 to 2018. *Chinese Jourl of Vector Biology and Control* 2020; **31**(2): 7.
563. Zou Yang ZH, Zhang Yunzhi, Mi Zhuqing, Yang Weihong, Yuan Qinghong, Zhang Yuzhen, Wang Jinglin, Xue Yanping, Chen Huaxin, Zhang Yongzhen. Epidemiological study on hantavirus in Rattus norvegicus and Rattus flavipectus in Yunn Province. *Chinese Jourl of Vector Biology and Control* 2006; **17**(5): 5.
564. Du Yanhua LY, Ma Hong, Wang Haifeng, Xu Bianli, Huang Xueyong. Etiological alysis of hantavirus in rats in Hen Province from 2014 to 2016. *Tianjin Pharmaceutical* 2017; **045**(006): 648-51.
565. Shaoxia S. Gene Sequence Analysis and Molecular Epidemiology of Hemorrhagic Fever with Renal Syndrome Virus: Shandong University; 2005.
566. Liu Dongying LJ, Li Jinlin, Chen Wen, Luo Fan, Li Qing, Yang Zhanqiu. Genetic alysis on S segment of hantaviruses in rodent hosts from Wuhan area, Hubei province. *Chinese Jourl of Epidemiology* 2012; **33**(8): 4.
567. Wang QW, Tao L, Lu SY, et al. Genetic and hosts characterization of hantaviruses in port areas in Hainan Province, P. R. China. *PLoS One* 2022; **17**(3): e0264859.
568. Yan Qingli XY, Li Bingbing, Pang Yixiu, He Fang, Liu Chuncheng, Zhao Huairong, Yang Pengfei, Yao Haibo, He njiang. Genetic characteristics of rodents infected with positive Seoul virus in Huai'an

City, Jiangsu Province. *Chinese Jourl of Frontier Health and Quarantine* 2019; (4): 6.
569. Su Q, Chen Y, Li M, et al. Genetic Characterization and Molecular Evolution of Urban Seoul Virus in Southern China. *Viruses* 2019; **11**(12).
570. Fan Shengtao GX, Li Yuanguo, Ying Ying, Guo Jiao, Zhang Zhaowei, Liu Hong, Wu Jing, Yang Songtao, Zhao Yongkun, Qin Chuan, Gao Yuwei, Xia Xianzhu. Genetic evolution of hantavirus carried by host animals in Jilin Province. *Chinese Jourl of Biological Products* 2014; **27**(4): 6.
571. Tao Z, Wang Z, Song S, Wen H, Ren G, Wang G. Genetic properties of medium (M) and small (S) genomic RNA segments of Seoul hantavirus isolated from Rattus norvegicus and antigenicity analysis of recombinant nucleocapsid protein. *Virus Genes* 2007; **34**(1): 23-30.
572. Sun Li ZY, Li Linhong, Zhang Yanping, Zhang Aimei, Hao Zongyu, Sun Jianwei, Chen Huaxin. Genetics subtypes and distribution of Seoul virus in Hen. *Chinese Jourl of Epidemiology* 2005; **26**(8): 5.
573. Sun Bingxin SY, Shen Bo, Wu Jing. Genotype alysis of hantavirus in rats in Changchun. *Chinese Jourl of Vector Biology and Control* 2015; **26**(6): 5.
574. Zhao Chen ZY, Li Yan, Yuan Zhonghai, Sun Kexin, Zhang Lei, Luo Enjie. Genotype alysis of HFRS pathogens in Jilin Province. *J Microbiol* 2011; **31**(5): 4.
575. Bao Huimin WY, Yuan Lihong, Chen Shouyi, Yang Zhicong, Lu Jiahai. Genotypes of hantavirus in small mammals in Guangzhou. *Jourl of Tropical Medicine* 2018; **18**(5): 4.
576. Geng Yingzhi TJ, Liu Yun, Wang Bo, Sun Yingwei, Li Xin, Yao Wenqing. Genotypic alysis of Seoul hantavirus in Liaoning Province. *Public Health in China* 2012; **28**(012): 1594-6.
577. Wang Aon DY, Li Boqi, Li Yongyong, Liu Zhu. Genotyping and sequence alysis of viruses carried by wild animals in Raohe area. *Chinese Jourl of Zoonoses* 2020; **36**(3): 5.
578. Zhang Fengxian ZY, Chen Huaxin, Zhang Yumin, Bo Fubao, Wang Dawei, Yang Yueqing, Guo Qiang, Chu Xiuzhen, Liu Xiaoping, Ren Xianyun. Genotyping of hantavirus in rats in Hohhot. *Chinese Jourl of Vector Biology and Control* 2007; **18**(1): 45-8.
579. Yan Yanzhen YL, Hu Guangwei, Du Zhansen, Li Minghui, Zhang Yongzhen. Genotyping of Seoul hantavirus in Jilin Province. *Chinese Jourl of Vector Biology and Control* 2006; **17**(4): 3.
580. Jing W. Geographic epidemiology and pathogenic gene sequence analysis of hemorrhagic fever with renal syndrome in Changchun: Jilin University.
581. Zuo SQ, Fang LQ, Zhan L, et al. Geo-spatial hotspots of hemorrhagic fever with renal syndrome and genetic characterization of Seoul variants in Beijing, China. *PLoS Negl Trop Dis* 2011; **5**(1): e945.
582. Yin Xiaoping SF, Zhao Shanshan, Tian Yanhe, Bartel, Cheng Tianli, Zhang Jiangguo, Gao Yufeng. Hantavirus detected from small mammals in the Chi-Kazakhstan border. *Chinese Jourl of Vector Biology and Control* 2018; **29**(1): 4.
583. Zhang YZ, Zhang FX, Wang JB, et al. Hantaviruses in rodents and humans, Inner Mongolia Autonomous Region, China. *Emerg Infect Dis* 2009; **15**(6): 885-91.
584. Zhang YZ, Lin XD, Shi NF, et al. Hantaviruses in small mammals and humans in the coastal region of Zhejiang Province, China. *J Med Virol* 2010; **82**(6): 987-95.
585. Tan Yi GF, Wei Zengliang, Yang Jinye. Host animals and virus infection of hemorrhagic fever with rel syndrome in Guangxi. *Chinese Jourl of Vector Biology and Control* 2010; (006): 000.
586. Cheng Cheng JW, Fu Weiming, Cao Suya, Xu Ning, Wang Yanmei, Geng Cong, Wang Yulong. Investigation on hantavirus infection in rodents in Heilongjiang Port Area. *Chinese Jourl of Zoonoses* 2015; **31**(7): 6.
587. Zhao Shuang CZ, Zhan Zhihui, Ye Tingting, Wang Xiuyan. Investigation on Hantaviruses carried by rodents in nsha ports. *Chinese Jourl of Vector Biology and Control* 2016; **27**(6): 3.
588. Jiang Jiafu WX, Wang Riming, Zuo Shuqing, Xu Weicai, Guo Tianyu, Chen Liquan, Cao Wuchun. Investigation on hantaviruses infection in rodents from free markets in Beijing areas. *Chinese Jourl of Epidemiology* 2006; **27**(2): 5.
589. Wang Yan WY, Han Xu. Investigation on rodent composition and hantavirus in hemorrhagic fever with rel syndrome epidemic area in Hebei Province. *Chinese Jourl of Vector Biology and Control* 2017; **28**(6): 4.
590. Qu Yonggang YG, Zou Yang, Yan Genqiang, Chen Huaxin, Zhang Yongzhen. Isolation and characterization of Hantavirus carried by rodents in Huludao, Liaoning province. *Chinese Jourl of Epidemiology* 2006; **27**(6): 5.
591. Wei Yamei HZ, Zhang Yanbo, Han Xu, Cai Yan, Xu Yonggang, Qi Shunxiang, Li Qi. Isolation, recovery and typing of hantavirus in Hebei Province. *Chinese Jourl of Vector Biology and Control* 2016; **027**(005): 447-9,58.
592. Liu S, Wei Y, Han X, et al. Long-term retrospective observation reveals stabilities and variations of hantavirus infection in Hebei, China. *BMC Infect Dis* 2019; **19**(1): 765.
593. Hu T, Fan Q, Hu X, et al. Molecular and serological evidence for Seoul virus in rats (Rattus norvegicus) in Zhangmu, Tibet, China. *Arch Virol* 2015; **160**(5): 1353-7.

594. Wang Zhiqiang WY, Fu Jihua, Zhao Ling, Sun Chengyang, Zhang Xueqi, Zhang Yanxue, Fan Shuzhen, Wang nda. Molecular biology of hantavirus Shandong isolate. *Chin Exp Clin Virol* 2003; **17**(2): 3.
595. Zhang Xu MM, Zhang Zhen, Tian Jing, Sun Peilong, Zhou Dayu, Bai Mei, Zhang Haochun, Zhong Yi. Molecular epidemiological characteristics of rodent hantavirus in Jinzhou City. *Public Health in China* 2015; **31**(1): 3.
596. Yang Pengfei MC, Gao , Zhang Yongzhen, Zhu Jianbo. Molecular epidemiological investigation of hantavirus in Bayannur area. *Jourl of Southwest Normal University* 2008; **33**(3): 4.
597. Duan Zhengxiu TY, Xiong Haiping, Chen Huaxin, Zhang Yongzhen, Zheng Jinhai. Molecular epidemiological investigation of hantavirus in Ouhai area of Wenzhou City. *Chinese Tropical Medicine* 2009; **9**(4): 3.
598. Na G. Molecular epidemiological investigation of hantavirus in Xinjiang and some areas of Inner Mongolia: Shihezi University; 2008.
599. Yao Kun JJ, Zhang Wenyi, Zhao Qiumen, Jiang Xining, Zuo Shuqing, Wu Xiaoming, Zhan Lin, Chu Chenyi, Zhang Panhe, Yang Hong, Cao Wuchun. Molecular epidemiological investigation of hantavirus infection in host animals in some areas of Northeast China. *Chinese Jourl of Pathogenic Biology* 2007; **2**(1): 4.
600. Ma Hongbo SH, Du Jian, Tan Hua, Lin Jican, Yao Ruodong, Ke Mingjian, Feng Zili. Molecular epidemiological study on hemorrhagic fever with rat kidney syndrome virus at Zhuhai Port. *Chinese Jourl of Frontier Health and Quarantine* 2009; **32**(4): 4.
601. Yu W. Molecular epidemiology of hantavirus in hemorrhagic fever with renal syndrome focus: Xinjiang Agricultural University; 2003.
602. Wang Yuping XQ, Cai Hengzhong, Chen Jun, Zhang Jianming, Zheng Yanping, Gao Bo. Molecular epidemiology of hantavirus in rodents at Fujian port. *Chinese Jourl of Vector Biology and Control* 2012; **23**(4): 3.
603. Fan Feineng YP, Shi nfeng, Gao , Chen Guohua, Li Minghui, Zou Yang, Deng Xiaozhao, Zhang Yongzhen. Molecular epidemiology of hantavirus in rodents in Cixi City, Zhejiang Province. *Chinese Jourl of Epidemiology* 2008; **29**(4): 4.
604. Xiong Haiping LM, Zhu Ying, Duan Zhengxiu, Tian Junhua, Chen Huaxin, Zhang Yongzhen. Molecular epidemiology of hantavirus in rodents in Wuhan. *Chinese Tropical Medicine* 2010; **10**(6): 3.
605. Zhang Xiaomei SS, Zhai Wenji, Wang Mei, Li Xiaojuan, Wang Zhiqiang, Li Dexin, Zhang Quanfu. Molecular epidemiology of hantavirus in Shandong Province. *Chinese Jourl of Vector Biology and Control* 2009; **20**(5): 4.
606. Wang Zhiyu SS, Wang Zhiqiang, Bi Zhenqiang. Molecular epidemiology of hemorrhagic fever with rel syndrome virus isolated in Shandong. In: Epidemiology CJo, editor. East China Epidemiology Academic Exchange; 2004; 2004.
607. Zhou Jihua ZH, Wang Jinglin, Yang Weihong, Zhang Yuzhen. Tural infection and genotyping of hantavirus in rodents in four counties of Yunn Province. *Chinese Jourl of Preventive Medicine* 2009.
608. Li Yun SL, Liu Jiann, Yan Jihuan, Nie Weizhong, Liu Shuping. Sequence alysis of hantavirus S gene carried by Rattus norvegicus at Qinhuangdao Port. *Chinese Jourl of Vector Biology and Control* 2017; **28**(5): 3.
609. Guan Fuyin TY, Mo Jianjun, Xie Zhenguo, Chen Minmei. Study on hantavirus genotype in northern Guangxi. *Applied Preventive Medicine* 2016; **22**(5): 4.
610. Li Jinmei ZH, Yang Weihong, Zhou Jihua, Wang Jinglin, Zhang Yuzhen, Deng Shuzhen. Study on host animals and genotyping of hantavirus in Yunn Province in 2007. *Endemic Diseases Bulletin* 2009; (6): 1-7.
611. Zhou Jihua ZH, Wang Jinglin, Yang Weihong, Mi Zhuqing, Zhang Yunzhi, Zhang Yuzhen, Song Xianyi, Hu Qiuling, Dong Yingkuan, Pu Wenhua, Hu Haimei, Gao Lifen, Yuan Qinghong, Ya Hongxiang, Feng Yun. Study on host animals and molecular epidemiology of hantavirus in Chuxiong Prefecture, Yunn Province. *Chinese Jourl of Epidemiology* 2009.
612. Fan Feineng XJ, Fan Chunping. Surveillance and alysis of hemorrhagic fever with rel syndrome in Cixi City from 1996 to 2005. *Chinese Jourl of Vector Biology and Control* 2008; **19**(3): 254-5.
613. Gao Yufeng SG, Tan Ze, Jiang Lu, Cheng Xiaolan, Song Fenglin. Surveillance and detection of rodents and hantavirus in Liaoning port from 2008 to 2015. *Chinese Jourl of Frontier Health and Quarantine* 2017; **40**(2): 4.
614. Wang Zhiqiang YX, Wang Yulu, Wang Xianjun, Kang Dianmin, Wang Mei, Li Zhongzheng. Surveillance of host virus of hemorrhagic fever with rel syndrome in Shandong Province from 1998 to 2002. *Preventive medicine literature information* 2003; (06): 59-60.
615. Yang Fan G, Liu Jianjun, Yang Hong, Zhang Xiaolan, He Jianfan, Liang Zhuon, Zhang Shunxiang, Yao Pingping, Weng Jingqing, He Yaqing. Surveillance on tural infection of rodents with hantavirus in

Shenzhen city and identification of a hantavirus strain SZ2083. *Chinese Jourl of Epidemiology* 2006; **27**(11): 981-4.
616. Lin Xiandan YP, Liao Xiaowei, Li Meifu, Gao , Chen Yi, Zeng Shidian, Wen Huaijia, Chen Lingping, Li Minghui, Zhang Yongzhen. The molecular epidemiologic investigation of hantavirus carried by rodent hosts in Wenzhou, Zhejiang province. *Chinese Jourl of Epidemiology* 2008; **29**(9): 4.
617. Liu DY, Liu J, Liu BY, et al. Phylogenetic analysis based on mitochondrial DNA sequences of wild rats, and the relationship with Seoul virus infection in Hubei, China. *Virol Sin* 2017; **32**(3): 235-44.
618. Sun X, Shi Y, Huang P, et al. Rats (Rattus norvegicus and Rattus losea) harboring Seoul hantavirus in Qingyuan, southern China: a survey during 2011-2013. *Southeast Asian J Trop Med Public Health* 2014; **45**(5): 1058-64.
619. Zhang YZ, Dong X, Li X, et al. Seoul virus and hantavirus disease, Shenyang, People's Republic of China. *Emerg Infect Dis* 2009; **15**(2): 200-6.
620. Guo G, Sheng J, Wu X, et al. Seoul virus in the Brown Rat (Rattus norvegicus) from Ürümqi, Xinjiang, Northwest of China. *J Wildl Dis* 2016; **52**(3): 705-8.
621. Zuo SQ, Zhang PH, Jiang JF, et al. Seoul virus in patients and rodents from Beijing, China. *Am J Trop Med Hyg* 2008; **78**(5): 833-7.
622. Zhou JH, Zhang HL, Wang JL, et al. [Survey on host animal and molecular epidemiology of hantavirus in Chuxiong prefecture, Yunnan province]. *Zhonghua Liu Xing Bing Xue Za Zhi* 2009; **30**(3): 239-42.
623. Zhao T, Dang YQ, Wang AN, et al. Viral Metagenomics Analysis of Rodents From Two Border Provinces Located in Northeast and Southwest China. *Front Microbiol* 2021; **12**: 701089.
624. Zhang X, Zhou S, Wang H, Hu J, Guan Z, Liu H. [Study on the genetic difference of SEO type Hantaviruses]. *Zhonghua Liu Xing Bing Xue Za Zhi* 2000; **21**(5): 349-51.
625. Zhang FX, Zou Y, Chen HX, et al. [Study on the epidemiological characteristics of hemorrhagic fever with renal syndrome in Inner Mongolia]. *Zhonghua Liu Xing Bing Xue Za Zhi* 2007; **28**(11): 1101-4.
626. Yuan ZG, Luo SJ, Xu HJ, et al. Generation of E3-deleted canine adenovirus type 2 expressing the Gc glycoprotein of Seoul virus by gene insertion or deletion of related terminal region sequences. *J Gen Virol* 2010; **91**(Pt 7): 1764-71.
627. Yuan ZG, Li XM, Mahmmod YS, Wang XH, Xu HJ, Zhang XX. A single immunization with a recombinant canine adenovirus type 2 expressing the seoul virus Gn glycoprotein confers protective immunity against seoul virus in mice. *Vaccine* 2009; **27**(38): 5247-51.
628. Yuan Z, Zhang X, Zhang S, et al. Development of recombinant canine adenovirus type-2 expressing the Gn glycoprotein of Seoul virus. *Biologicals* 2008; **36**(3): 162-7.
629. Yao LS, Qin CF, Pu Y, et al. Complete genome sequence of Seoul virus isolated from Rattus norvegicus in the Democratic People's Republic of Korea. *J Virol* 2012; **86**(24): 13853.
630. Yang F, Guli B, Liu JJ, et al. [Surveillance on natural infection of rodents with hantavirus in Shenzhen city and identification of a hantavirus strain SZ2083]. *Zhonghua Liu Xing Bing Xue Za Zhi* 2006; **27**(11): 981-4.
631. Xiao H, Tong X, Gao L, et al. Spatial heterogeneity of hemorrhagic fever with renal syndrome is driven by environmental factors and rodent community composition. *PLoS Negl Trop Dis* 2018; **12**(10): e0006881.
632. Wang Y, Cao S, Cheng C, Ju W, Hua Y. Genetic characterization of a hantavirus isolated from Heilongjiang province, China. *Can J Vet Res* 2019; **83**(1): 75-7.
633. Wang N, Yin JX, Zhang Y, et al. Genetic Evolution Analysis and Host Characteristics of Hantavirus in Yunnan Province, China. *Int J Environ Res Public Health* 2022; **19**(20).
634. Tao Z, Wang Z. Sequence analysis of the M genome segment of two Seoul virus isolates from Shandong Province, China. *Acta Virol* 2007; **51**(1): 67-8.
635. Sun L, Zhang YZ, Li LH, et al. [Genetics subtypes and distribution of Seoul virus in Henan]. *Zhonghua Liu Xing Bing Xue Za Zhi* 2005; **26**(8): 578-82.
636. Plyusnina A, Ibrahim IN, Plyusnin A. A newly recognized hantavirus in the Asian house rat (Rattus tanezumi) in Indonesia. *J Gen Virol* 2009; **90**(Pt 1): 205-9.
637. Luo Y, Li Y, Huang YL, et al. [Genetic characteristics of hantavirus detected in rodents in Shenzhen]. *Zhonghua Liu Xing Bing Xue Za Zhi* 2022; **43**(11): 1804-10.
638. Lin XD, Yang PF, Liao XW, et al. [The molecular epidemiologic investigation of hantavirus carried by rodent hosts in Wenzhou, Zhejiang province]. *Zhonghua Liu Xing Bing Xue Za Zhi* 2008; **29**(9): 891-4.
639. Lin XD, Guo WP, Wang W, et al. Migration of Norway rats resulted in the worldwide distribution of Seoul hantavirus today. *J Virol* 2012; **86**(2): 972-81.
640. Li Y, Cazelles B, Yang G, et al. Intrinsic and extrinsic drivers of transmission dynamics of

hemorrhagic fever with renal syndrome caused by Seoul hantavirus. *PLoS Negl Trop Dis* 2019; **13**(9): e0007757.
641. Li G, Pan L, Mou D, et al. Characterization of truncated hantavirus nucleocapsid proteins and their application for serotyping. *J Med Virol* 2006; **78**(7): 926-32.
642. Lee C, Jeong J, Lee T, et al. Virus-mimetic polymer nanoparticles displaying hemagglutinin as an adjuvant-free influenza vaccine. *Biomaterials* 2018; **183**: 234-42.
643. Kaya S, Yılmaz G, Erensoy S, Yağçı Çağlayık D, Uyar Y, Köksal I. [Hantavirus infection: two case reports from a province in the Eastern Black Sea Region, Turkey]. *Mikrobiyol Bul* 2010; **44**(3): 479-87.
644. Kariwa H, Isegawa Y, Arikawa J, et al. Comparison of nucleotide sequences of M genome segments among Seoul virus strains isolated from eastern Asia. *Virus Res* 1994; **33**(1): 27-38.
645. Jiang JF, Wu XM, Wang RM, et al. [Investigation on hantaviruses infection in rodents from free markets in Beijing areas]. *Zhonghua Liu Xing Bing Xue Za Zhi* 2006; **27**(2): 145-9.
646. Jiang F, Wang L, Wang S, et al. Meteorological factors affect the epidemiology of hemorrhagic fever with renal syndrome via altering the breeding and hantavirus-carrying states of rodents and mites: a 9 years' longitudinal study. *Emerg Microbes Infect* 2017; **6**(11): e104.
647. Guo WP, Lin XD, Wang W, Li MH, Zhu HB, Zhang YZ. [Genetic characterization of two Seoul virus strains isolated from patients and rats in Hebei province]. *Zhonghua Liu Xing Bing Xue Za Zhi* 2010; **31**(8): 916-9.
648. Fan FN, Yang PF, Shi NF, et al. [Study on the molecular epidemiology of hantavirus carried by rodent hosts in Cixi, Zhejiang province]. *Zhonghua Liu Xing Bing Xue Za Zhi* 2008; **29**(4): 365-8.
649. Duan ZX, Li MH, Yu J, Wang W, Guo WP, Zhang YZ. [Comparison on the difference of virulence between Hantaan virus and Seoul virus isolated both from Rattus norvegicus]. *Zhonghua Liu Xing Bing Xue Za Zhi* 2009; **30**(10): 1047-50.
650. Clement J, LeDuc JW, Lloyd G, et al. Wild Rats, Laboratory Rats, Pet Rats: Global Seoul Hantavirus Disease Revisited. *Viruses* 2019; **11**(7).
651. Chan YC, Wong TW, Yap EH. Haemorrhagic fever with renal syndrome: clinical, virological and epidemiological perspectives. *Ann Acad Med Singap* 1987; **16**(4): 696-701.
652. Yongtao S. Molecular Biological Studies on Genotyping, Mutation and Detection of Hantavirus [Ph D]: The Fourth Military Medical University; 1996.
653. Shuqing Z. Molecular epidemiology of hemorrhagic fever with renal syndrome in beijing [Ph.D]: PLA Academy of Military Medical Sciences; 2007.
654. Huang P. Rats(Rattus norvegicus and losea) harboring Seoul hantavirus in southern China. 2015 Hotspot Seminar on Prevention and Treatment of New Infectious Diseases; 2015; Guangzhou, Guangdong, China; 2015. p. 6.
655. Winterroot, Jie L, Hengyi X, Shumei T, Qun H. Establishment of Double Fluorescence RT-RAA Method for Detection of Hantavirus in Hemorrhagic Fever with Renal Syndrome. *Chinese Journal of Frontier Health and Quarantine* 2022; **45**(04): 253-6+63.
656. Fengxian Z, Yongzhen Z, Huaxin C, et al. Genotyping of hantavirus in rodents in hohhot. *Chinese Journal of Vector Biology and Control* 2007; (01): 45-8.
657. Zhen Z, Lingling M, Yingwei S, et al. Carrying rate and gene sequence analysis of Hantavirus in rodents in Jinzhou City. *Chinese sanitary insecticide* 2018; **24**(06): 572-4.
658. Qingli Y, Xingyuezhen, Bingbing L, et al. Study on the Genetic Characteristics of Rodents Infected with Seoul Virus in Huai'an City, Jiangsu Province. *Chinese Journal of Frontier Health and Quarantine* 2019; **42**(04): 254-8+83.
659. Yanzhen Y, Laishun Y, Guangwei H, Zhansen D, Minghui L, Yongzhen Z. Genotyping of seoul type hantavirus in jilin province. *Chinese Journal of Vector Biology and Control* 2006; (04): 324-6.
660. Hongxiang Y, Yunzhi Z. Diagnosis and analysis of mixed infection of tsutsugamushi disease and hemorrhagic fever with renal syndrome in Yunnan Province. *Chinese Journal of Zoonoses* 2016; **32**(03): 266-70.
661. Ping W, Weijia J, Jingzhu Z, et al. Etiological detection of serum samples and rat lung samples from patients in high incidence areas of hemorrhagic fever with renal syndrome in Guizhou Province in 2013 and 2014. *Chinese sanitary insecticide* 2022; **28**(05): 443-6.
662. Jingqing W, Ronghui X, Yiyu L, et al. Characterization of M Gene Sequence of Hantavirus Microtus fortis Strain ZT10. *J Virologica Sinica* 2006; (03): 235-7.
663. Bingxin S, Yu S, Bo S, Jing W. Genotype Analysis of Hantavirus Carried by Rodents in Changchun City. *Chinese Journal of Vector Biology and Control* 2015; **26**(06): 600-4.
664. Yancheng L, Shuhong C, Lufei C, Kaili W, Jing Z, Jihong L. Isolation and Gene Identification of a Seoul Type Hantavirus. *Public Health Management in China* 2009; **25**(05): 487-8.
665. Li S, Yongzhen Z, Linhong L, et al. Study on genotype and distribution of type ii hantavirus in henan province. *Chinese Journal of Epidemiology* 2005; (08): 578-82.

666. Bo S, Donglin W, Dongfeng P, et al. Isolation and identification of three Seoul type viruses of hemorrhagic fever with renal syndrome in Hunchun City, Jilin Province. *Chinese Journal of Public Health Engineering* 2008; (03): 167-9.
667. Shiwen L, Yanni Z, Fang X, et al. Isolation, Identification and Whole Genome Sequence Analysis of Two Seoul Viruses in Gao'an City, Jiangxi Province. *Chinese Journal of Zoonoses* 2022; **38**(02): 102-7.
668. Shiwen L, Gang X, Tian G, et al. Detection and genotyping of rat hantavirus in Nanchang. *Modern Preventive Medicine* 2016; **43**(06): 1085-8+96.
669. Shiwen L, Ying X, Courageously, Jianxiong L, Qian W, Tian G. Establishment of a dual real-time fluorescent quantitative RT-PCR method for detection of Hantan virus and Seoul virus. *Chinese Journal of Zoonoses* 2021; **37**(06): 478-83.
670. Minghui L, Xiaoping C, Guoqing Y, et al. Analysis of Hantavirus Carried by Rattus norvegicus in Huludao Residential Area. *Chinese Journal of Vector Biology and Control* 2011; **22**(03): 239-42.
671. Wei H, Zhanqiu Y, Jijiang Y, et al. Genotypes of Hantavirus in Different Areas of Hubei Province *Public Health in China* 2002; (06): 31-3.
672. Yingzhi G, Tianjiang, Yun L, et al. Analysis of Genotypes of Hantavirus of Seoul Type in Liaoning Province. *Chinese Journal of Public Health* 2012; **28**(12): 1594-6.
673. Shengtao F, Xiaolong G, Yuanguo L, et al. Genetic evolution of hantavirus in jilin province. *Chinese Journal of Biological Products* 2014; **27**(04): 467-71+75.
674. Xiaoqing D, Limin S, Yaqian W, Min H, Ning DS, Shougang Z. Serological detection and genotyping analysis of suspected cases of hemorrhagic fever with renal syndrome in Nanjing metropolitan area. *Chinese Journal of Zoonoses* 2022; **38**(04): 322-6.
675. Ning T, Wang L, Liu S, et al. Monitoring Neutralization Property Change of Evolving Hantaan and Seoul Viruses with a Novel Pseudovirus-Based Assay %J Virologica Sinica. 2021; **36**(01): 104-12.
676. Liu D-Y, Liu J, Liu B-Y, et al. Phylogenetic analysis based on mitochondrial DNA sequences of wild rats, and the relationship with Seoul virus infection in Hubei, China %J Virologica Sinica. 2017; **32**(03): 235-44.
677. Na G. Molecular epidemiology of hantavirus in xinjiang and inner mongolia: Shihezi University; 2008.
678. Weisheng M. Study on host changes and epidemiological characteristics of hemorrhagic fever with renal syndrome in shandong province from 2003 to 2005: Shandong University; 2006.
679. Xu H. Study on the characteristics of hantavirus and the genetic characteristics of hantavirus population in hebei province: Hebei Medical University; 2009.
680. Xiaoxia H. Study on Pathogen Genotypes and Distribution Characteristics of Hemorrhagic Fever with Renal Syndrome in Epidemic Focus of Hebei Province: Hebei Medical University; 2006.
681. Essbauer S, Schmidt J, Conraths F, et al. A new Puumala hantavirus subtype in rodents associated with an outbreak of Nephropathia epidemica in South-East Germany in 2004. 2006; **134**(6): 1333-44.
682. Wang Y-p, Zhang X-l, Zhang J-m, et al. Complete genome sequence of a novel mutation of Seoul virus isolated from Suncus murinus in the Fujian Province of China. 2015; **3**(2): e00075-15.
683. Tao Z, Wang Z, Song S, Wen H, Ren G, Wang GJVG. Genetic properties of medium (M) and small (S) genomic RNA segments of Seoul hantavirus isolated from Rattus norvegicus and antigenicity analysis of recombinant nucleocapsid protein. 2007; **34**(1): 23-30.
684. Kariwa H, Isegawa Y, Arikawa J, et al. Comparison of nucleotide sequences of M genome segments among Seoul virus strains isolated from eastern Asia. 1994; **33**(1): 27-38.
685. Macé G, Feyeux C, Mollard N, et al. Severe Seoul hantavirus infection in a pregnant woman, France, October 2012. *Euro Surveill* 2013; **18**(17): 20464.
686. Jameson LJ, Logue CH, Atkinson B, et al. The continued emergence of hantaviruses: isolation of a Seoul virus implicated in human disease, United Kingdom, October 2012. *Euro Surveill* 2013; **18**(1): 4-7.
687. Plyusnina A, Ferenczi E, Rácz GR, et al. Co-circulation of three pathogenic hantaviruses: Puumala, Dobrava, and Saaremaa in Hungary. *J Med Virol* 2009; **81**(12): 2045-52.
688. Liu Fuqiang CL, Gao Lidong, Zeng Ge, Cai Liang. Alysis on Surveillance Results of Hemorrhagic Fever with Rel Syndrome in Hun Province in 2008. *Practical Preventive Medicine* 2009; **16**(4): 4.
689. Du Jie WJ, Zhu Liye, Jiang Tao, Ding Zhentao, Liu Qiquan. Alysis on HFRS surveillance of Fuyang in 2008. *Anhui Medicine* 2010; **31**(4): 3.
690. Liu Yi LZ, Wang Limao, Duan Yongjun, Yue Qi, Wang Hong, Danbazeli, Qi Teng, Huang Jianzhong, Li Fan, Wu Chaoxue, He Qianjun, Ze Rensangzhu. Alysis of experimental detection results of Dege pestis tural focus in 2008. *Jourl of Preventive Medicine Information* 2010; (1): 1-3.
691. Liu Yong LJ, Zhai Wenji, Zhang Xiaomei, Wang Zhiqiang, Li Shixue. Epidemic situation and Prevention Countermeasures of hemorrhagic fever with rel syndrome in Shandong Province from 2008

to 2011. *Preventive Medicine Forum* 2012; **18**(2): 3.
692. Lu Aitao GW, Song Zhuangzhi, Han Song, Huo Lixia, Liu Baolong. Inter-rat surveillance alysis of rel syndrome hemorrhagic fever in Inner Mongolia in 2008_2011. *Chinese Jourl of Preventive Medicine* 2012; **13**(9): 2.
693. Wang Chengxiang YY, Liang Kun, Ma Huan, Ji Hongmei. Alysis of pestis Surveillance Results in Urumqi County, Xinjiang in 2013. *Disease prevention and control notification* 2014; (2): 2.
694. Yijie X. Epidemic characteristics of epidemic hemorrhagic fever in Baishan City from 2008 to 2017. *Jourl of Strait Preventive Medicine* 2019; (1): 2.
695. Yuhong Y. Surveillance and alysis of hemorrhagic fever with rel syndrome in Bayannur City from 2000 to 2005. *Preventive Medicine Forum* 2006; (6): 2.
696. Zhang Lingzhi YL, Li Mengmeng, Li Baorong. Surveillance and Epidemic Alysis of Hemorrhagic Fever with Rel Syndrome in Chifeng City from 2005 to 2009. *Medical animal control* 2010; **26**(12): 2.
697. Zhang Lingzhi YL, Chen Dongli. Surveillance and epidemic alysis of hemorrhagic fever with rel syndrome in Chifeng City from 1998 to 2005. *Medical animal control* 2006; **22**(1): 2.
698. Tang Tianyong TY, Tang Jing, Huang Hua, Shen Huakun, Yuan Dongya. Monitoring of urban, farmland and farmhouse rats and investigation of hemorrhagic fever with rel syndrome(HFＲS) virus. *Medical animal control* 2017; **33**(6): 3.
699. Deng Liangli TW, Xiao Lin, He Jianhan, Liao Jun, Jiang Zheng, Liu Zhu, Ma Lin, Shi Wei. Investigation on rat population density and hantavirus carrying rate of Rattus norvegicus in Chengdu City. *Modern Preventive Medicine* 2011.
700. Hao Chaoxin FJ, Sun Yingjie, Cheng Jigeng, Wang Jianzhong. Discussion on the relationship between seasol changes of Rattus norvegicus and the incidence of Rattus norvegicus hemorrhagic fever. *Public Health in China* 1993.
701. Liu Changqing HZ, Qi Shunxiang. Investigation on epidemic situation of hemorrhagic fever with rel syndrome among rats in 6 cities of Hebei Province. *Chinese Jourl of Vector Biology and Control* 2000.
702. Han Xu HZ, Wei Yamei, Zhang Yanbo, Xu Yonggang, Qi Shunxiang, Li Qi. Epidemiology of hemorrhagic fever with rel syndrome in Hebei province in 2012. *Chinese Tropical Medicine* 2013.
703. Han Zhanying WY, Han Xu, Zhang Yanbo, Xu Yonggang, Qi Shunxiang, Li Qi. Epidemiological alysis of hemorrhagic fever with rel syndrome in Hebei province, Chi in 2011. *Chinese Jourl of Vector Biology and Control* 2013.
704. Zhang Zuoru NJ, Meng Zongda. Investigation and epidemic situation alysis of epidemic hemorrhagic fever in Hebei Province in 1984. *Chinese Jourl of Rodent Control* 1986.
705. Zhou Yong YW, Huang Aiwu, Liu Jiaqin. Background investigation report on rodents and mites at Hefei Airport Port. *Chinese Jourl of Frontier Health and Quarantine* 2008; **31**(3): 195-7.
706. Sun Binglin HZ, Kong Fanping, Xu Qinfang, Lu Liming, Zhu Shiyan, Sheng Guo'an, Li Li. It is the first time that social mice carry epidemic hemorrhagic fever virus antigen in Lin'an County, Hangzhou. *Chinese Jourl of Rodent Control* 1985; (1).
707. Yan Fuzhi WZ, Liao Zishu, Lv Taifu, Tang Yuping. Investigation on Infectious Sources of Epidemic Hemorrhagic Fever in Guizhou province. *Chinese Jourl of Epidemiology* 2020.
708. Wang Zhaoxiao LT, Fu Deqing, Lu Daqi, Wang Jianmin, Yan Fuzhi, Liao Zishu, Yang Xiuzhen, Fan Jingwei, Fan Haiqun, Jiang Guowei, Liu Yuefang, Hong Zhimin, Yang Maowen. Epidemiological study on epidemic hemorrhagic fever in Guizhou Province. *Guizhou Medicine* 1989.
709. Fu Deqing TH, Han Yong, Zhang Caihong. Investigation on host animals of HFRS in Guiyang Longdongbao Airport. *Guizhou Medicine* 2000; **24**(9): 2.
710. Zhimin P. Surveillance of epidemic hemorrhagic fever in Huangpu District of Guangzhou in 1992. *Hygiene and epidemic prevention in Guangdong* 1993; (4): 23-6.
711. Pu Shaoming QJ, Li Xinrui. A Monitor and alyse on epidemic of hemorrhagic fever with rel syndrome from Fangcun district in Guangzhou *Medical animal control* 2004; **20**(4): 237-.
712. Liang Caiyun XY, Qiu Jichun, Di Biao, Yao Jianming. Isolation of hemorrhagic fever virus with pulmory rel syndrome from Rattus norvegicus in Baiyun Mountain, Guangzhou. *Chinese Jourl of Vector Biology and Control* 2000; **11**(1): 2.
713. Hao Ruifeng LF, Guo Rongtong, Zhao Ronghui, Yan Yuchen. Epidemic hemorrhagic fever virus isolated from Rattus norvegicus for the first time in Guangdong Province. *Guangzhou Pharmaceutical* 1986; (05): 13-6.
714. Chaohui Z. Investigation on epidemic hemorrhagic fever virus in rodent belt in Fuping County. *Chinese Jourl of Rodent Control* 1985.
715. Deng Huimin WQ, Guan Chen Ping. Epidemic Alysis of Epidemic Hemorrhagic Fever in Fuzhou from 1984 to 2004. *Jourl of Strait Preventive Medicine* 2006; **12**(1): 2.
716. Li Xianfeng HC, Hong Chaochang, Pan Liang, Yuan Gaolin, Lin Kailing, Weng Wenzhong, Xiao Shusheng, Huang Zhengjing, Li Yanting. Epidemiological investigation of hemorrhagic fexueqing in rel

syndrome in mountainous areas of Northern Fujian. *Chinese Jourl of Epidemiology* 2020.
717. Wang Ruikang WY. Epidemiological investigation and alysis of epidemic hemorrhagic fever in Langxi County from 1965 to 1995. *Grass roots medicine in Chi* 1997.
718. Li Bingxin MS, An Jinlong, Jin Jun, Jin Donghui, Shao Lijun, Cao Guoxiang. Investigation on tural infection of rodent hantavirus in Ji'an port area. *Port Health Control* 2012; (3): 3.
719. Yan Yanzhen ZY, Yao Laishun, Hu Guangwei, Li Minghui, Du Zhansen, Chen Huaxin. Typing of hantavirus in Rattus norvegicus in Jilin Province. *Proceedings of the tiol Symposium on Zoonosis* 2005.
720. Zhang YZ, Zou Y, Yao LS, et al. Isolation and characterization of hantavirus carried by Apodemus peninsulae in Jilin, China. *J Gen Virol* 2007; **88**(Pt 4): 1295-301.
721. Zhang YZ, Xiao QY, Li MH, et al. [An epidemiologle investigation of hantaviruses carried by rodent hosts in Hunan province]. *Zhonghua Liu Xing Bing Xue Za Zhi* 2007; **28**(1): 65-9.
722. Zhang YZ, Xiao DL, Wang Y, et al. [The epidemic characteristics and preventive measures of hemorrhagic fever with syndromes in China]. *Zhonghua Liu Xing Bing Xue Za Zhi* 2004; **25**(6): 466-9.
723. Zhang YZ. Discovery of hantaviruses in bats and insectivores and the evolution of the genus Hantavirus. *Virus Res* 2014; **187**: 15-21.
724. Zhang Y, Lu SQ, Zhang YZ, Zhao BX, Xu JG. [The study for the immuno-protection of E. coli O157:H7 outer membrane protein]. *Zhonghua Yi Xue Za Zhi* 2004; **84**(1): 58-62.
725. Xiong CL, Hao ZY, Lu SQ, et al. [Analyses on the isolation and characterization of canine rabies virus isolates in Henan]. *Zhonghua Liu Xing Bing Xue Za Zhi* 2007; **28**(3): 261-7.
726. Wang MR, Wang W, Lin XD, Mei SH, Guo WP, Zhang YZ. [Investigation on the natural infectious status of hantaviruses among small mammals in Longquan city, Zhejiang province]. *Zhonghua Liu Xing Bing Xue Za Zhi* 2011; **32**(6): 598-601.
727. Dong X, Zhang YZ, Li X, Zhao CZ, Wang B. [Genotype of isolated strain of hantavirus in Liaoning area]. *Zhonghua Shi Yan He Lin Chuang Bing Du Xue Za Zhi* 2005; **19**(1): 39-42.
728. Dong GM, Xu GL, Xiao QY, et al. [An epidemiological study of rabies virus in domestic dogs, cats and wildlife and the immunogenicity study for rabies vaccines derived from different cell cultured virus strains]. *Bing Du Xue Bao* 2007; **23**(6): 417-23.
729. Chen XP, Cong ML, Li MH, et al. Infection and pathogenesis of Huaiyangshan virus (a novel tick-borne bunyavirus) in laboratory rodents. *J Gen Virol* 2012; **93**(Pt 6): 1288-93.
730. Zuo SQ, Wu XM, Sun PY, et al. [Study on the molecular epidemiology of hantaviruse carried by hosts in northern suburb of Beijing]. *Zhonghua Liu Xing Bing Xue Za Zhi* 2004; **25**(5): 421-4.
731. Zheng Z, Wang P, Wang Z, et al. The characteristics of current natural foci of hemorrhagic fever with renal syndrome in Shandong Province, China, 2012-2015. *PLoS Negl Trop Dis* 2019; **13**(5): e0007148.
732. Jiang JF, Zuo SQ, Zhang WY, et al. Prevalence and genetic diversities of hantaviruses in rodents in Beijing, China. *Am J Trop Med Hyg* 2008; **78**(1): 98-105.
733. Jiang JF, Wu XM, Zuo SQ, et al. [Study on the association between hantavirus infection and Rattus norvegicus]. *Zhonghua Liu Xing Bing Xue Za Zhi* 2006; **27**(3): 196-9.
734. Zhou J, Zhang X, Chen M, et al. Epidemiological study on hemorrhagic fever with renal syndrome in flood areas. *Zhong Nan Da Xue Xue Bao Yi Xue Ban* 2011; **36**(3): 223-8.
735. Zhao R, Zhu BL, Guan DW, et al. Diagnostic aspects for epidemic hemorrhagic fever in legal medical autopsy: report of 2 cases and review. *Leg Med (Tokyo)* 2009; **11 Suppl 1**: S541-3.
736. Zhao Q, Yang X, Liu H, et al. Effects of climate factors on hemorrhagic fever with renal syndrome in Changchun, 2013 to 2017. *Medicine (Baltimore)* 2019; **98**(9): e14640.
737. Zhang YZ, Holmes EC. What is the time-scale of hantavirus evolution? *Infect Genet Evol* 2014; **25**: 144-5.
738. Zhang WY, Wang LY, Liu YX, et al. Spatiotemporal transmission dynamics of hemorrhagic fever with renal syndrome in China, 2005-2012. *PLoS Negl Trop Dis* 2014; **8**(11): e3344.
739. Zhang WY, Fang LQ, Jiang JF, et al. Predicting the risk of hantavirus infection in Beijing, People's Republic of China. *Am J Trop Med Hyg* 2009; **80**(4): 678-83.
740. Zeng Y, Feng Y, Zhao Y, et al. An HFman Probe-Based Multiplex Reverse Transcription Loop-Mediated Isothermal Amplification Assay for Simultaneous Detection of Hantaan and Seoul Viruses. *Diagnostics (Basel)* 2022; **12**(8).
741. Yu XJ, Tesh RB. The role of mites in the transmission and maintenance of Hantaan virus (Hantavirus: Bunyaviridae). *J Infect Dis* 2014; **210**(11): 1693-9.
742. Yu PB, Tian HY, Ma CF, et al. Hantavirus infection in rodents and haemorrhagic fever with renal syndrome in Shaanxi province, China, 1984-2012. *Epidemiol Infect* 2015; **143**(2): 405-11.
743. Yu PB, Li S, Wei J, et al. [Comparison of direct immune-fluorescent assay and real-time quantitative PCR in detecting the Hantavirus]. *Zhonghua Yu Fang Yi Xue Za Zhi* 2013; **47**(4): 367-70.
744. Yu J, Deng XZ, Yang ZQ, et al. [Study on the transmission of Hantaan virus and Orientia

tsutsugamushi by naturally dual infected Leptotrombidium scutellare through stinging]. *Zhonghua Yu Fang Yi Xue Za Zhi* 2010; **44**(4): 324-8.
745. Ye W, Xu Y, Wang Y, et al. Hantaan virus can infect human keratinocytes and activate an interferon response through the nuclear translocation of IRF-3. *Infect Genet Evol* 2015; **29**: 146-55.
746. Yao PP, Zhu HP, Xu F, et al. [Study on the difference of genes and the type identification of hantavirus from Lishui, Zhejiang province]. *Zhonghua Liu Xing Bing Xue Za Zhi* 2009; **30**(2): 175-8.
747. Yao PP, Zhu HP, Deng XZ, et al. [Molecular evolution analysis of hantaviruses in Zhejiang Province]. *Bing Du Xue Bao* 2010; **26**(6): 465-70.
748. Yang X, Wang C, Wu L, Jiang X, Zhang S, Jing F. Hemorrhagic fever with renal syndrome with secondary hemophagocytic lymphohistiocytosis in West China: a case report. *BMC Infect Dis* 2019; **19**(1): 492.
749. Yang J, Sun JF, Wang TT, et al. Targeted inhibition of hantavirus replication and intracranial pathogenesis by a chimeric protein-delivered siRNA. *Antiviral Res* 2017; **147**: 107-15.
750. Xu R, Yang XY, Yang DF, Zou CY, Gong PL, Zeng FD. Phase I evaluation of the safety and pharmacokinetics of a single-dose intravenous injection of a murine monoclonal antibody against Hantaan virus in healthy volunteers. *Antimicrob Agents Chemother* 2009; **53**(12): 5055-9.
751. Xiao H, Tong X, Huang R, et al. Landscape and rodent community composition are associated with risk of hemorrhagic fever with renal syndrome in two cities in China, 2006-2013. *BMC Infect Dis* 2018; **18**(1): 37.
752. Xiao H, Huang R, Gao LD, et al. Effects of Humidity Variation on the Hantavirus Infection and Hemorrhagic Fever with Renal Syndrome Occurrence in Subtropical China. *Am J Trop Med Hyg* 2016; **94**(2): 420-7.
753. Wu Z, Lu L, Du J, et al. Comparative analysis of rodent and small mammal viromes to better understand the wildlife origin of emerging infectious diseases. *Microbiome* 2018; **6**(1): 178.
754. Wu Y, Wang J, Liu Q, Li T, Luo M, Gong Z. Practice of integrated vector surveillance of arthropod vectors, pathogens and reservoir hosts to monitor the occurrence of tropical vector-borne diseases in 2020 in Zhejiang Province, China. *Front Vet Sci* 2022; **9**: 1003550.
755. Wu W, Guo J, Guan P, Sun Y, Zhou B. Clusters of spatial, temporal, and space-time distribution of hemorrhagic fever with renal syndrome in Liaoning Province, Northeastern China. *BMC Infect Dis* 2011; **11**: 229.
756. Wei X, Meng B, Peng H, et al. Hemorrhagic fever with renal syndrome caused by destruction of residential area of rodent in a construction site: epidemiological investigation. *BMC Infect Dis* 2022; **22**(1): 761.
757. Wei X, Li X, Song S, et al. Trends and focuses of hantavirus researches: a global bibliometric analysis and visualization from 1980 to 2020. *Arch Public Health* 2022; **80**(1): 218.
758. Watson DC, Sargianou M, Papa A, Chra P, Starakis I, Panos G. Epidemiology of Hantavirus infections in humans: a comprehensive, global overview. *Crit Rev Microbiol* 2014; **40**(3): 261-72.
759. Wang X, Shen W, Qin Y, et al. A case-control study on the risk factors for hemorrhagic fever with renal syndrome. *BMC Infect Dis* 2020; **20**(1): 103.
760. Wang QW, Huang P, Yue M, et al. [Genetic characteristics of hantavirus from rodents in port area of Ningde , Fujian province in the summer of 2020]. *Zhonghua Liu Xing Bing Xue Za Zhi* 2021; **42**(7): 1266-73.
761. Wang Q, Zhou H, Han YH, et al. [Epidemiology and surveillance programs on hemorrhagic fever with renal syndrome in Mainland China, 2005 - 2008]. *Zhonghua Liu Xing Bing Xue Za Zhi* 2010; **31**(6): 675-80.
762. Wang Q, Yue M, Yao P, et al. Epidemic Trend and Molecular Evolution of HV Family in the Main Hantavirus Epidemic Areas From 2004 to 2016, in P.R. China. *Front Cell Infect Microbiol* 2020; **10**: 584814.
763. Tian H, Yu P, Cazelles B, et al. Interannual cycles of Hantaan virus outbreaks at the human-animal interface in Central China are controlled by temperature and rainfall. *Proc Natl Acad Sci U S A* 2017; **114**(30): 8041-6.
764. Tian H, Yu P, Bjørnstad ON, et al. Anthropogenically driven environmental changes shift the ecological dynamics of hemorrhagic fever with renal syndrome. *PLoS Pathog* 2017; **13**(1): e1006198.
765. Tang Z, Xu XJ, He XJ, et al. Analysis of hemorrhagic fever with renal syndrome and its pathogenic gene sequence based on geographic information system. *J Biol Regul Homeost Agents* 2017; **31**(2): 431-8.
766. Tan Y, Bi FY, Wei ZL. [Identification and analysis of an SEO strain of hemorrhagic fever with renal syndrome hantavirus from Guangxi]. *Zhonghua Shi Yan He Lin Chuang Bing Du Xue Za Zhi* 2008; **22**(6): 443-5.
767. Sun L, Shao Q, Wang ZQ, et al. Spatial structure of rodent populations and infection patterns of

hantavirus in seven villages of Shandong Province from February 2006 to January 2007. *Chin Med J (Engl)* 2011; **124**(11): 1639-46.
768. Singh H, Kaur H, Medhi B. Novel therapeutic approaches toward Hantaan virus and its clinical features' similarity with COVID-19. *Indian J Pharmacol* 2020; **52**(5): 347-55.
769. Shen L, Sun M, Wei X, et al. Spatiotemporal association of rapid urbanization and water-body distribution on hemorrhagic fever with renal syndrome: A case study in the city of Xi'an, China. *PLoS Negl Trop Dis* 2022; **16**(1): e0010094.
770. She K, Li C, Qi C, et al. Epidemiological Characteristics and Regional Risk Prediction of Hemorrhagic Fever with Renal Syndrome in Shandong Province, China. *Int J Environ Res Public Health* 2021; **18**(16).
771. Shang C, Zhang QF, Yin QL, Li DX, Li JD. [Influence factors related epidemics on hantavirus disease]. *Zhonghua Liu Xing Bing Xue Za Zhi* 2020; **41**(6): 968-74.
772. Qi R, Sun XF, Qin XR, et al. Suggestive Serological Evidence of Infection with Shrew-Borne Imjin Virus (Hantaviridae) in Humans. *Viruses* 2019; **11**(12).
773. Onyuok SO, Hu B, Li B, et al. Molecular Detection and Genetic Characterization of Novel RNA Viruses in Wild and Synanthropic Rodents and Shrews in Kenya. *Front Microbiol* 2019; **10**: 2696.
774. Ma HW, Ye W, Chen HS, et al. In-Cell Western Assays to Evaluate Hantaan Virus Replication as a Novel Approach to Screen Antiviral Molecules and Detect Neutralizing Antibody Titers. *Front Cell Infect Microbiol* 2017; **7**: 269.
775. Ma C, Wang Z, Li S, et al. Analysis of an outbreak of hemorrhagic fever with renal syndrome in college students in Xi'an, China. *Viruses* 2014; **6**(2): 507-15.
776. Luo Y, Lv H, Yan H, et al. Meteorological change and hemorrhagic fever with renal syndrome epidemic in China, 2004-2018. *Sci Rep* 2022; **12**(1): 20037.
777. Lü X, Zhang F, Li Y, Xue X, Yin W, Xu Z. Antigenic characterization of expressed complete and different truncated recombinant nucleocapsid proteins of hantaan virus by monoclonal antibodies. *Hybridoma (Larchmt)* 2011; **30**(5): 445-50.
778. Lü X, Yin W, Yang Q, et al. Identification of oligopeptides mimicking the receptor-binding domain of Hantaan virus envelope glycoprotein from a phage-displayed peptide library. *Can J Microbiol* 2009; **55**(6): 664-71.
779. Liu Z, Wang F, Yuan L, et al. Development of a SYBR-Green I quantitative PCR assay for the detection and genotyping of different hantaviruses. *Int J Mol Med* 2016; **38**(3): 951-60.
780. Liu YY, Chen LJ, Zhong Y, et al. Specific interference shRNA-expressing plasmids inhibit Hantaan virus infection in vitro and in vivo. *Acta Pharmacol Sin* 2016; **37**(4): 497-504.
781. Liu XY, Xue KN, Rong R, Zhao CH. Fault Tree Analysis: Investigation of Epidemic Hemorrhagic Fever Infection Acquired in Animal Laboratories in China. *Biomed Environ Sci* 2016; **29**(9): 690-5.
782. Liu X, Zhang T, Xie C, Xie Y. Changes of HFRS Incidence Caused by Vaccine Intervention in Yichun City, China, 2005-2013. *Med Sci Monit* 2016; **22**: 295-301.
783. Liu X, Jiang B, Gu W, Liu Q. Temporal trend and climate factors of hemorrhagic fever with renal syndrome epidemic in Shenyang City, China. *BMC Infect Dis* 2011; **11**: 331.
784. Liu R, Ma R, Liu Z, et al. HTNV infection of CD8(+) T cells is associated with disease progression in HFRS patients. *Commun Biol* 2021; **4**(1): 652.
785. Liu R, Ma H, Shu J, et al. Vaccines and Therapeutics Against Hantaviruses. *Front Microbiol* 2019; **10**: 2989.
786. Liu JJ, Yang F, He JF, et al. [Study on the molecular characteristic of natural infection of rodents with Hantaviruses in Shenzhen city]. *Zhonghua Yu Fang Yi Xue Za Zhi* 2008; **42**(5): 324-8.
787. Liu E, Lv S, Yi P, et al. Central nervous system infection with Seoul Orthohantavirus in a child after hematopoietic stem cell transplantation: a case report. *Virol J* 2022; **19**(1): 75.
788. Li XG, Zhai WJ, Li XJ. [Research on hantanvirus infection of rodents in Junan county, Shandong province]. *Zhonghua Liu Xing Bing Xue Za Zhi* 2009; **30**(7): 760.
789. Li Q, Cai Y, Wei Y, et al. Genovariation Study of Hantavirus in Main Endemic Areas of Hemorrhagic Fever with Renal Syndrome in Hebei Province, China. *PLoS One* 2016; **11**(7): e0159731.
790. Lee SH, Kim WK, No JS, et al. Dynamic Circulation and Genetic Exchange of a Shrew-borne Hantavirus, Imjin virus, in the Republic of Korea. *Sci Rep* 2017; **7**: 44369.
791. Klein TA, Kim HC, Chong ST, et al. Hantaan virus surveillance in small mammals at firing points 10 and 60, Yeoncheon, Gyeonggi Province, Republic of Korea. *Vector Borne Zoonotic Dis* 2012; **12**(8): 674-82.
792. Klein TA, Kim HC, Chong ST, et al. Hantaan virus surveillance targeting small mammals at nightmare range, a high elevation military training area, Gyeonggi Province, Republic of Korea. *PLoS One* 2015; **10**(4): e0118483.
793. Kikuchi F, Aoki K, Ohdachi SD, et al. Genetic Diversity and Phylogeography of Thottapalayam

thottimvirus (Hantaviridae) in Asian House Shrew (Suncus murinus) in Eurasia. *Front Cell Infect Microbiol* 2020; **10**: 438.
794. Kariwa H. [Rodent associated hantaviruses and hantavirus infections]. *Uirusu* 2017; **67**(1): 25-32.
795. Kang HJ, Gu SH, Cook JA, Yanagihara R. Dahonggou Creek virus, a divergent lineage of hantavirus harbored by the long-tailed mole (Scaptonyx fusicaudus). *Trop Med Health* 2016; **44**: 16.
796. Jiang DB, Sun YJ, Cheng LF, et al. Construction and evaluation of DNA vaccine encoding Hantavirus glycoprotein N-terminal fused with lysosome-associated membrane protein. *Vaccine* 2015; **33**(29): 3367-76.
797. Jalal S, Kim CM, Kim DM, et al. Geographical clustering of Hantavirus isolates from Apodemus agrarius identified in the Republic of Korea indicate the emergence of a new Hantavirus genotype. *J Clin Virol* 2022; **146**: 105030.
798. Huang Y, Zhao L, Zhang Z, et al. Detection of a Novel Rickettsia From Leptotrombidium scutellare Mites (Acari: Trombiculidae) From Shandong of China. *J Med Entomol* 2017; **54**(3): 544-9.
799. Huang XX, Li Q, Han ZY, Zhang YB, Wei YM, Xu YG. [Genotyping and sequence analysis on G2 segment of hantavirus carried by rat in Hebei province]. *Zhonghua Shi Yan He Lin Chuang Bing Du Xue Za Zhi* 2009; **23**(3): 165-7.
800. Huang LY, Zhou H, Yin WW, et al. [The current epidemic situation and surveillance regarding hemorrhagic fever with renal syndrome in China, 2010]. *Zhonghua Liu Xing Bing Xue Za Zhi* 2012; **33**(7): 685-91.
801. Hu XQ, Li SG, Liu H, Wang J, Hua RM. Diversity and distribution of host animal species of hantavirus and risk to human health in Jiuhua mountain area, China. *Biomed Environ Sci* 2014; **27**(11): 849-57.
802. Hu G, Bai WT, Wu XA, Wang HT, Xu ZK, Zhang FL. [Construction and immunogenic study of recombinant adenovirus containing chimeric gene G2S0.7 and CTL epitopes of Hantaan virus]. *Xi Bao Yu Fen Zi Mian Yi Xue Za Zhi* 2010; **26**(11): 1070-4.
803. He J, Wang Y, Mu D, et al. The Impacts of Climatic Factors and Vegetation on Hemorrhagic Fever with Renal Syndrome Transmission in China: A Study of 109 Counties. *Int J Environ Res Public Health* 2019; **16**(18).
804. He J, Christakos G, Wu J, et al. Spatiotemporal variation of the association between climate dynamics and HFRS outbreaks in Eastern China during 2005-2016 and its geographic determinants. *PLoS Negl Trop Dis* 2018; **12**(6): e0006554.
805. Guo Y, Wang W, Sun Y, et al. Crystal Structure of the Core Region of Hantavirus Nucleocapsid Protein Reveals the Mechanism for Ribonucleoprotein Complex Formation. *J Virol* 2016; **90**(2): 1048-61.
806. Guo G, Guo B, Wu X, et al. Molecular evidence of Tula virus in Microtus obscurus in the region of Yili, Xinjiang, China. *BMC Infect Dis* 2019; **19**(1): 527.
807. Gong ZY, Hou J, Liu QY, et al. [Study on comprehensive monitoring of mouse and effect of hemorrhagic fever with renal syndrome vaccine in high prevalence areas of natural focus infectious disease of Zhejiang province in 1994--2010]. *Zhonghua Yu Fang Yi Xue Za Zhi* 2012; **46**(10): 908-11.
808. Goeijenbier M, Verner-Carlsson J, van Gorp EC, et al. Seoul hantavirus in brown rats in the Netherlands: implications for physicians--Epidemiology, clinical aspects, treatment and diagnostics. *Neth J Med* 2015; **73**(4): 155-60.
809. Fang LQ, Goeijenbier M, Zuo SQ, et al. The association between hantavirus infection and selenium deficiency in mainland China. *Viruses* 2015; **7**(1): 333-51.
810. Dong Y, Ma T, Zhang X, et al. Incorporation of CD40 ligand or granulocyte-macrophage colony stimulating factor into Hantaan virus (HTNV) virus-like particles significantly enhances the long-term immunity potency against HTNV infection. *J Med Microbiol* 2019; **68**(3): 480-92.
811. Deng XZ, Zhang Y, Kong J, et al. [Molecular epidemiological study on the host and role of the Hantavirus and Orientia tsutsugamushi in the same epidemic area]. *Zhonghua Yu Fang Yi Xue Za Zhi* 2008; **42**(8): 574-7.
812. Chen JT, Qin J, Li K, et al. Identification and characterization of a novel subtype of Tula virus in Microtus arvalis obscurus voles sampled from Xinjiang, China. *Infect Genet Evol* 2019; **75**: 104012.
813. Chang ZR, Lu L, Mao DQ, et al. Dynamics of Rodent and Rodent-borne Disease during Construction of the Three Gorges Reservoir from 1997 to 2012. *Biomed Environ Sci* 2016; **29**(3): 197-204.
814. Cao SC, Li JD, Lu P, et al. [Hantavirus mucosal vaccine through different mucosal with heat-labile enterotoxin B subunit as adjuvants]. *Zhonghua Shi Yan He Lin Chuang Bing Du Xue Za Zhi* 2008; **22**(3): 174-6.
815. Cao S, Zhang Y, Liu F, et al. Secretory expression and purification of recombinant Escherichia coli heat-labile enterotoxin B subunit and its applications on intranasal vaccination of hantavirus. *Mol*

Biotechnol 2009; **41**(2): 91-8.
816. Plyusnin A, Cheng Y, Lehväslaiho H, Vaheri AJVG. Unique and conserved features of Tula hantavirus M gene encoding envelope glycoproteins G1 and G2. 1996; **12**(3): 257-63.
817. Scharninghausen JJ, Pfeffer M, Meyer H, et al. Genetic evidence for Tula virus in Microtus arvalis and Microtus agrestis populations in Croatia. 2002; **2**(1): 19-27.
818. Plyusnin A, Cheng Y, Vapalahti O, et al. Genetic variation in Tula hantaviruses: sequence analysis of the S and M segments of strains from Central Europe. 1995; **39**(2-3): 237-50.
819. Reusken C, de Vries A, Adema J, et al. First genetic detection of Tula hantavirus in wild rodents in the Netherlands. 2008; **57**(6): 500-3.
820. Bowen MD, Gelbmann W, Ksiazek TG, Nichol ST, Nowotny NJJoMV. Puumala virus and two genetic variants of Tula virus are present in Austrian rodents. 1997; **53**(2): 174-81.
821. Tkachenko EA, Witkowski PT, Radosa L, et al. Adler hantavirus, a new genetic variant of Tula virus identified in Major's pine voles (Microtus majori) sampled in southern European Russia. *Infect Genet Evol* 2015; **29**: 156-63.
822. Plyusnin A, Vapalahti O, Lankinen H, et al. Tula virus: a newly detected hantavirus carried by European common voles. *J Virol* 1994; **68**(12): 7833-9.
823. Vapalahti O, Lundkvist A, Kukkonen SK, et al. Isolation and characterization of Tula virus, a distinct serotype in the genus Hantavirus, family Bunyaviridae. *J Gen Virol* 1996; **77 (Pt 12)**: 3063-7.
824. Two New Hantaviruses Found in China. *Chinese Journal of Difficult and Complicated Cases* 2008; **7**(4): 1.
825. Lin XD, Zhou RH, Fan FN, et al. Biodiversity and evolution of Imjin virus and Thottapalayam virus in Crocidurinae shrews in Zhejiang Province, China. *Virus Res* 2014; **189**: 114-20.
826. Qi R, Sun XF, Qin XR, Wang LJ, Yu XJJV. Suggestive Serological Evidence of Infection with Shrew-Borne Imjin Virus (Hantaviridae) in Humans. 2019; **11**(12): 1128.
827. Kim, Young-Sik, Kurata, et al. Lethal disease in infant and juvenile Syrian hamsters experimentally infected with Imjin virus, a newfound crocidurine shrew-borne hantavirus. 2015.
828. Gu SH, Kang HJ, Baek LJ, et al. Genetic diversity of Imjin virus in the Ussuri white-toothed shrew (Crocidura lasiura) in the Republic of Korea, 2004-2010. *Virol J* 2011; **8**: 56.
829. Song JW, Kang HJ, Gu SH, et al. Characterization of Imjin virus, a newly isolated hantavirus from the Ussuri white-toothed shrew (Crocidura lasiura). *J Virol* 2009; **83**(12): 6184-91.
830. Song JW, Baek LJ, Schmaljohn CS, Yanagihara RJEID. Thottapalayam Virus, a Prototype Shrewborne Hantavirus. 2007; **13**(7): 980-5.
831. Okumura M, Yoshimatsu K, Kumperasart S, Nakamura I, Arikawa JJC, Immunology V. Development of Serological Assays for Thottapalayam Virus, an Insectivore-Borne Hantavirus▽. 2007; **14**(2): 173-81.
832. Guo WP, Lin XD, Wen W, et al. A new subtype of Thottapalayam virus carried by the Asian house shrew (Suncus murinus) in China. 2011; **11**(8): 1862-7.
833. Yong-Kyu C, Gerald J, Alan S, et al. Cross-Neutralization of Hantaviruses with Immune Sera from Experimentally Infected Animals and from Hemorrhagic Fever with Renal Syndrome and Hantavirus Pulmonary Syndrome Patients. (6): 1581.
834. Wang Zhanju WX, Li Xiaopeng, Zhao Xiaomei, Cheng Yaozu, Wei or, Pu Xingchang, Shi Xueming. Discovery of Human Hepatitis B Virus Carried by Citellus dauricus and Serial Verification Study. *Medical animal control* 1997; **13**(3): 3.
835. Song Weihan CY, Yin Hongzhen, Jiang Yunpeng, Zhu Dezhi, Yuan Baoshan, Liu Yue, Song Weiguo. A Prelimiry Study on Hepatitis B like Hepatitis in Citellus dauricus. *Jourl of Bethune Medical University* 1991.
836. Zhanju W, Xiaobo W, Xiaopeng L, et al. Discovery of Human Hepatitis B Virus Carried by Citellus dauricus and Serial Verification Study. *Chinese Journal of Pest Control* 1997; **13**(3): 3.
837. Yu JM, Li LL, Zhang CY, et al. A novel hepatovirus identified in wild woodchuck Marmota himalayana. *Sci Rep* 2016; **6**: 22361.
838. Li LL, Liu MM, Shen S, et al. Detection and characterization of a novel hepacivirus in long-tailed ground squirrels (Spermophilus undulatus) in China. *Arch Virol* 2019; **164**(9): 2401-10.
839. Nie FY, Tian JH, Lin XD, et al. Discovery of a highly divergent hepadnavirus in shrews from China. *Virology* 2019; **531**: 162-70.
840. Yao Z. Investigation on Hepatitis E Infection of Rat like Animals in Three Counties (Cities) of pestis Foci of Rattus domesticus in Yunnan. 1991.
841. He W, Wen Y, Xiong Y, Zhang M, Cheng M, Chen Q. The prevalence and genomic characteristics of hepatitis E virus in murine rodents and house shrews from several regions in China. *BMC Vet Res* 2018; **14**(1): 414.
842. Wang B, Li W, Zhou JH, et al. Chevrier's Field Mouse (Apodemus chevrieri) and Père David's Vole

(Eothenomys melanogaster) in China Carry Orthohepeviruses that form Two Putative Novel Genotypes Within the Species Orthohepevirus C. *Virol Sin* 2018; **33**(1): 44-58.
843. Bai H, Li W, Guan D, et al. Characterization of a Novel Rat Hepatitis E Virus Isolated from an Asian Musk Shrew (Suncus murinus). *Viruses* 2020; **12**(7).
844. Cheng X. Epidemic Survey of Anti HEV Antibody in Rodents in Southern Xinjiang. *Chinese Community Physician (Medical Major)* 2012.
845. Li W, Guan D, Su J, et al. High prevalence of rat hepatitis E virus in wild rats in China. *Vet Microbiol* 2013; **165**(3-4): 275-80.
846. Khuroo MS, Khuroo MS, Khuroo NS. Hepatitis E: Discovery, global impact, control and cure. *World J Gastroenterol* 2016; **22**(31): 7030-45.
847. Zheng XY, Qiu M, Ke XM, et al. Molecular Detection and Phylogenetic Characteristics of Herpesviruses in Rectal Swab Samples from Rodents and Shrews in Southern China. *Vector Borne Zoonotic Dis* 2016; **16**(7): 476-84.
848. Yao Z. Investigation on the infection of hepatitis E in rodents from three counties of plague natural foci in Yunnan Province. *Dali University* 2021.
849. Morikawa S, Qing T, Xinqin Z, Saijo M, Kurane I. Genetic diversity of the M RNA segment among Crimean-Congo hemorrhagic fever virus isolates in China. *Virology* 2002; **296**(1): 159-64.
850. Tang Q, Zhao XQ, Wang HY, et al. [Molecular epidemiology of Xinjiang hemorrhagic fever viruses]. *Zhonghua Shi Yan He Lin Chuang Bing Du Xue Za Zhi* 2005; **19**(4): 312-8.
851. Bente DA, Forrester NL, Watts DM, McAuley AJ, Whitehouse CA, Bray M. Crimean-Congo hemorrhagic fever: history, epidemiology, pathogenesis, clinical syndrome and genetic diversity. *Antiviral Res* 2013; **100**(1): 159-89.
852. Morikawa S, Saijo M, Kurane I. Recent progress in molecular biology of Crimean-Congo hemorrhagic fever. *Comp Immunol Microbiol Infect Dis* 2007; **30**(5-6): 375-89.
853. Liu P, Qiu Y, Xing C, et al. Detection and genome characterization of two novel papillomaviruses and a novel polyomavirus in tree shrew (Tupaia belangeri chinensis) in China. *Virol J* 2019; **16**(1): 35.
854. Wu Z, Yang L, Yang F, et al. Novel Henipa-like virus, Mojiang Paramyxovirus, in rats, China, 2012. *Emerg Infect Dis* 2014; **20**(6): 1064-6.
855. Chen JJ, Zhang XA, Fan H, et al. Distribution and characteristics of Beilong virus among wild rodents and shrews in China. *Infect Genet Evol* 2020; **85**: 104454.
856. Woo PCY, Wong AYP, Wong BHL, et al. Comparative genome and evolutionary analysis of naturally occurring Beilong virus in brown and black rats. *Infect Genet Evol* 2016; **45**: 311-9.
857. Woo PC, Lau SK, Wong BH, Wu Y, Lam CS, Yuen KY. Novel variant of Beilong Paramyxovirus in rats, China. *Emerg Infect Dis* 2012; **18**(6): 1022-4.
858. Li Z, Yu M, Zhang H, et al. Beilong virus, a novel paramyxovirus with the largest genome of non-segmented negative-stranded RNA viruses. *Virology* 2006; **346**(1): 219-28.
859. Woo PC, Lau SK, Wong BH, Wong AY, Poon RW, Yuen KY. Complete genome sequence of a novel paramyxovirus, Tailam virus, discovered in Sikkim rats. *J Virol* 2011; **85**(24): 13473-4.
860. Zhang C, Song F, Xiu L, et al. Identification and characterization of a novel rodent bocavirus from different rodent species in China. *Emerg Microbes Infect* 2018; **7**(1): 48.
861. Xiong YQ, You FF, Chen XJ, Chen YX, Wen YQ, Chen Q. Detection and phylogenetic analysis of porcine bocaviruses carried by murine rodents and house shrews in China. *Transbound Emerg Dis* 2019; **66**(1): 259-67.
862. Li Yongyong LH, Xing Tianyao, Lv Chengliang, Zhang Xiwei, Yu Wei. Alysis of rodent surveillance at Raohe port in Heilongjiang province. *Chinese Jourl of Frontier Health and Quarantine* 2020; **43**(4): 4.
863. Jiang Lu WG, Tan Yang, Cheng Xiaolan, Gao Yufeng, Yao Lisi. Detection of Boca like virus from rodents and its phylogenetic alysis. *Chinese Jourl of Frontier Health and Quarantine* 2017; **40**(4): 5.
864. He W, Gao Y, Wen Y, et al. Ungulate bocaparvovirus 4 and rodent bocavirus are different genotypes of the same species of virus. *Virol Sin* 2022; **37**(2): 215-22.
865. Lau SK, Yeung HC, Li KS, et al. Identification and genomic characterization of a novel rat bocavirus from brown rats in China. *Infect Genet Evol* 2017; **47**: 68-76.
866. Wang J, Li N, Li Z, et al. Identification of a novel bocaparvovirus in a wild squirrel in Kunming, Yunnan Province, China. *Arch Virol* 2020; **165**(6): 1469-74.
867. Xiong YQ, Zhou JH, Zhang MY, You FF, Li DL, Chen Q. Presence of rat bocavirus in oropharyngeal and fecal samples from murine rodents in China. *Arch Virol* 2018; **163**(11): 3099-103.
868. Ao Y, Li X, Li L, et al. Two novel bocaparvovirus species identified in wild Himalayan marmots. *Sci China Life Sci* 2017; **60**(12): 1348-56.
869. Sun Minghao LZ, Li Bingxin, Yang Huaining, Ma Shutao, He Chen. Investigation on rodents and their pathogens at Ji'an highway bridge port in 2016. *Chinese Jourl of Frontier Health and Quarantine*

2018; **41**(1): 3.
870. Liang Huijie WD, Wang Yumei, Zhang Jun, Shang Tianshu. Alysis on special monitoring of rodents at Heilongjiang border ports from 2019 to 2020. *Chi Port Science and Technology* 2021; **003**(010): 23-9.
871. Huaibo W, Qi S, Xifeng Y, et al. Investigation on rat borne diseases in the border area of Erenhot between China and Mongolia. 2018.
872. Tang, HB, Chen, et al. Characterization of Akabane virus from domestic bamboo rat, Southern China. 2017; **2017,207**(-): 280-5.
873. Liu JW, Wen HL, Fang LZ, et al. Prevalence of SFTSV among Asian house shrews and rodents, China, January-August 2013. *Emerg Infect Dis* 2014; **20**(12): 2126-8.
874. Huang XY, Du YH, Wang HF, et al. Prevalence of severe fever with thrombocytopenia syndrome virus in animals in Henan Province, China. *Infect Dis Poverty* 2019; **8**(1): 56.
875. Sun Y, Liu MM, Luo LM, et al. Seroprevalence of Severe Fever with Thrombocytopenia Syndrome Virus in Hedgehog from China. *Vector Borne Zoonotic Dis* 2017; **17**(5): 347-50.
876. Chen C, Li P, Li KF, et al. Animals as amplification hosts in the spread of severe fever with thrombocytopenia syndrome virus: A systematic review and meta-analysis. *Int J Infect Dis* 2019; **79**: 77-84.
877. Gu XL, Su WQ, Zhou CM, et al. SFTSV infection in rodents and their ectoparasitic chiggers. *PLoS Negl Trop Dis* 2022; **16**(8): e0010698.
878. Wang Qingkui GH, Hu Jianli, Zhang Zhenyu, Wang Yanping, Jiao Yongjun, Li Zhifeng, Hu Shuming, Lu Dajiang, Wang Xianghua, Liu Hanze. Surveillance of vector and host animals of fever with thrombocytopenia syndrome in Donghai County, Jiangsu Province from 2010 to 2011. *Chinese Jourl of Vector Biology and Control* 2013; (04): 313-6.
879. Wang J, Fan N, Fu S, et al. Isolation and Characterization of Wuxiang Virus from Sandflies Collected in Yangquan County, Shanxi Province, China. *Vector Borne Zoonotic Dis* 2021; **21**(6): 446-57.
880. Shen S, Duan X, Wang B, et al. A novel tick-borne phlebovirus, closely related to severe fever with thrombocytopenia syndrome virus and Heartland virus, is a potential pathogen. *Emerg Microbes Infect* 2018; **7**(1): 95.
881. Ni H, Yang F, Li Y, et al. Apodemus agrarius is a potential natural host of severe fever with thrombocytopenia syndrome (SFTS)-causing novel bunyavirus. *J Clin Virol* 2015; **71**: 82-8.
882. Li Z, Hu J, Bao C, et al. Seroprevalence of antibodies against SFTS virus infection in farmers and animals, Jiangsu, China. *J Clin Virol* 2014; **60**(3): 185-9.
883. Wang Y, Zhao J, Zheng M, et al. A novel cardiovirus in wild rats. *Virol J* 2018; **15**(1): 58.
884. Mahmood A, Shama S, Ni H, et al. Viral Metagenomics Revealed a Novel Cardiovirus in Feces of Wild Rats. *Intervirology* 2019; **62**(1): 45-50.
885. Ao Y, Xu J, Duan Z. A novel cardiovirus species identified in feces of wild Himalayan marmots. *Infect Genet Evol* 2022; **103**: 105347.
886. Zhang M, Li Q, Wu F, et al. Epidemiology, Genetic Characterization, and Evolution of Hunnivirus Carried by Rattus norvegicus and Rattus tanezumi: The First Epidemiological Evidence from Southern China. *Pathogens* 2021; **10**(6).
887. Lu G, Huang M, Chen X, et al. Identification and genome characterization of a novel feline picornavirus proposed in the Hunnivirus genus. *Infect Genet Evol* 2019; **71**: 47-50.
888. Zhang M, You F, Wu F, He H, Li Q, Chen Q. Epidemiology and genetic characteristics of murine kobuvirus from faecal samples of Rattus losea, Rattus tanezumi and Rattus norvegicus in southern China. *J Gen Virol* 2021; **102**(9).
889. You FF, Zhang MY, He H, He WQ, Li YZ, Chen Q. Kobuviruses carried by Rattus norvegicus in Guangdong, China. *BMC Microbiol* 2020; **20**(1): 94.
890. Gao Y, He W, Fu J, Li Y, He H, Chen Q. Epidemiological Evidence for Fecal-Oral Transmission of Murine Kobuvirus. *Front Public Health* 2022; **10**: 865605.
891. Du J, Lu L, Liu F, et al. Distribution and characteristics of rodent picornaviruses in China. *Sci Rep* 2016; **6**: 34381.
892. Phan TG, Kapusinszky B, Wang C, Rose RK, Lipton HL, Delwart EL. The fecal viral flora of wild rodents. *PLoS Pathog* 2011; **7**(9): e1002218.
893. Luo XL, Lu S, Jin D, Yang J, Wu SS, Xu J. Marmota himalayana in the Qinghai-Tibetan plateau as a special host for bi-segmented and unsegmented picobirnaviruses. *Emerg Microbes Infect* 2018; **7**(1): 20.
894. Wang L, Tang Q, Liang G. Rabies and rabies virus in wildlife in mainland China, 1990-2013. *Int J Infect Dis* 2014; **25**: 122-9.
895. Yu Jinning LH, Tang Qing, Tao Xiaoyan, Wu Hui, Mo Zhaojun, Zhang Hong, Wang Dingming, Weng Jingqing, Shen Ruihua, Zhu Fengcai, Wang Xianjun, Liu Hong, Shen Xinxin, Wang Shumei. Study

on the status of infection and distribution of rabies virus in China. *Chinese Jourl of Epidemiology* 2010; (5): 4.
896. Tang HB, Pan Y, Wei XK, et al. Re-emergence of rabies in the Guangxi province of Southern China. *PLoS Negl Trop Dis* 2014; **8**(10): e3114.
897. Li Y, Rainey JJ, Yang H, et al. Assessing clinicians' Post-Exposure Prophylaxis recommendations for rabies virus exposures in Hunan Province, China. *PLoS Negl Trop Dis* 2021; **15**(7): e0009564.
898. Chao J, Peng Q, Zhao J, et al. Different rabies outbreaks on two beef cattle farms in the same province of China: Diagnosis, virus characterization and epidemiological analysis. *Transbound Emerg Dis* 2021; **68**(3): 1216-28.
899. Wang Xinxing LJ, Wang Wenguang, Sun Xiaomei, He Chunyan, Dai Jiejie. Prelimiry investigation of viruses to the wild tree shrews (Tupaia belangeri Chinese). *Zoological research* 2011; (01): 66-9.
900. Meng X, Wang Y, Lu S, et al. Actinomyces gaoshouyii sp. nov., isolated from plateau pika (Ochotona curzoniae). *Int J Syst Evol Microbiol* 2017; **67**(9): 3363-8.
901. Tian Z, Zhang D, Lu S, et al. Georgenia wutianyii sp. nov. and Georgenia yuyongxinii sp. nov. isolated from plateau pika (Ochotona curzoniae) on the Qinghai-Tibet plateau of China. *Int J Syst Evol Microbiol* 2020; **70**(4): 2318-24.
902. Tian Z, Lu S, Jin D, et al. Mumia zhuanghuii sp. nov., isolated from the intestinal contents of plateau pika (Ochotona curzoniae) on the Qinghai-Tibet Plateau. *Int J Syst Evol Microbiol* 2020; **70**(4): 2233-8.
903. Tian Z, Yang J, Lai XH, et al. Microbacterium caowuchunii sp. nov. and Microbacterium lushaniae sp. nov., isolated from plateau pika (Ochotona curzoniae) on the Qinghai-Tibet Plateau of PR China. *Int J Syst Evol Microbiol* 2021; **71**(2).
904. Li J, Lei W, Yang J, et al. Aeromicrobium chenweiae sp. nov. and Aeromicrobium yanjiei sp. nov., isolated from Tibetan antelope (Pantholops hodgsonii) and plateau pika (Ochotona curzoniae), respectively. *Int J Syst Evol Microbiol* 2020; **70**(8): 4683-90.
905. Li J, Lu S, Yang J, et al. Actinomyces lilanjuaniae sp. nov., isolated from the faeces of Tibetan antelope (Pantholops hodgsonii) on the Qinghai-Tibet Plateau. *Int J Syst Evol Microbiol* 2019; **69**(11): 3485-91.
906. Yang C, Bai Y, Dong K, et al. Actinomyces marmotae sp. nov. and Actinomyces procaprae sp. nov. isolated from wild animals and reclassification of Actinomyces liubingyangii and Actinomyces tangfeifanii as Boudabousia liubingyangii comb. nov. and Boudabousia tangfeifanii comb. nov., respectively. *Int J Syst Evol Microbiol* 2019; **71**(3).
907. Zhou J, Zhang S, Zhang G, et al. Characterization of isolates of members of the genus Actinomyces from Marmota himalayana: description of Actinomyces faecalis sp. nov., Actinomyces respiraculi sp. nov., and Actinomyces trachealis sp. nov. *Int J Syst Evol Microbiol* 2021; **71**(7).
908. Dong K, Lu S, Yang J, et al. Nocardioides jishulii sp. nov.,isolated from faeces of Tibetan gazelle (Procapra picticaudata). *Int J Syst Evol Microbiol* 2020; **70**(6): 3665-72.
909. Zhang G, Yang J, Lai XH, et al. Corynebacterium zhongnanshanii sp. nov. isolated from trachea of Marmota himalayana, Corynebacterium lujinxingii sp. nov. and Corynebacterium wankanglinii sp. nov. from human faeces. *Int J Syst Evol Microbiol* 2021; **71**(11).
910. Dong K, Yang J, Lu S, et al. Microbacterium wangchenii sp. nov., isolated from faeces of Tibetan gazelles (Procapra picticaudata) on the Qinghai-Tibet Plateau. *Int J Syst Evol Microbiol* 2020; **70**(2): 1307-14.
911. Meng X, Lai XH, Lu S, et al. Actinomyces tangfeifanii sp. nov., isolated from the vulture Aegypius monachus. *Int J Syst Evol Microbiol* 2018; **68**(12): 3701-6.
912. Lu M, Li F, Liao Y, et al. Epidemiology and Diversity of Rickettsiales Bacteria in Humans and Animals in Jiangsu and Jiangxi provinces, China. *Sci Rep* 2019; **9**(1): 13176.
913. Masuzawa T, Uchishima Y, Fukui T, et al. Detection of Anaplasma phagocytophilum and Anaplasma bovis in small wild mammals from Taichung and Kinmen Island, Taiwan. *Jpn J Infect Dis* 2014; **67**(2): 111-4.
914. Hu Manxia ZJ, Fan Donghui, Fu Weiming, Sun Xiufeng, Yang Liwei. 绥芬河、东宁口岸地区鼠类感染新发蜱媒病原的检测. *Chinese Jourl of Vector Biology and Control* 2009; **20**(6): 4.
915. Lu Qunying SJ, Gu Shiping, Zheng Baifu, Zheng Shougui, Ge Junhua, Chai Chengliang, Jiang Liping, Ling Feng, Zhao Jiangling. Aplasma species among rodent animals,cattle and goats in Zhejiang. *Chinese Jourl of Zoonoses* 2018; **34**(4): 7.
916. Liping J, Shougui Z, Qunying L, Xiaodong Y, Profit, Suyun C. Ehrlichia and Intangible DNA fragments were detected and sequenced in rats in Zhejiang Province. *Chinese Journal of Health Laboratory Technology* 2007; **17**(11): 3.
917. Duan R, Lv D, Fan R, et al. Anaplasma phagocytophilum in Marmota himalayana. *BMC Genomics* 2022; **23**(1): 335.
918. Yan Y, Lu C, Gong P, et al. Molecular detection and phylogeny of Anaplasma spp. closely related

to Anaplasma phagocytophilum in small ruminants from China. *Ticks Tick Borne Dis* 2022; **13**(5): 101992.
919. Zheng W, Liu Y, Tao H, et al. First Molecular Evidence of Anaplasma phagocytophilum in Rodent Populations of Nanchang, China. *Jpn J Infect Dis* 2018; **71**(2): 129-33.
920. Zhang L, Liu H, Xu B, et al. Rural residents in China are at increased risk of exposure to tick-borne pathogens Anaplasma phagocytophilum and Ehrlichia chaffeensis. *Biomed Res Int* 2014; **2014**: 313867.
921. Dong T, Qu Z, Zhang L. Detection of A. phagocytophilum and E. chaffeensis in patient and mouse blood and ticks by a duplex real-time PCR assay. *PLoS One* 2013; **8**(9): e74796.
922. Zhao XG, Li H, Sun Y, et al. Dual infection with Anaplasma phagocytophilum and Babesia microti in a Rattus norvegicus, China. *Ticks Tick Borne Dis* 2013; **4**(5): 399-402.
923. Yang J, Liu Z, Guan G, et al. Prevalence of Anaplasma phagocytophilum in ruminants, rodents and ticks in Gansu, north-western China. *J Med Microbiol* 2013; **62**(Pt 2): 254-8.
924. Zhan L, Cao WC, Jiang JF, et al. Anaplasma phagocytophilum in livestock and small rodents. *Vet Microbiol* 2010; **144**(3-4): 405-8.
925. Zhan L, Cao WC, Jiang JF, et al. Anaplasma phagocytophilum from Rodents and Sheep, China. *Emerg Infect Dis* 2010; **16**(5): 764-8.
926. Bian CL, Gong ZD, Zhang LY, et al. [Identification of Anaplasma phagocytophilum in small mammals from Hengduan Mountains of Southwest China]. *Zhonghua Liu Xing Bing Xue Za Zhi* 2009; **30**(12): 1277-80.
927. Zhan L, Cao WC, Chu CY, et al. Tick-borne agents in rodents, China, 2004-2006. *Emerg Infect Dis* 2009; **15**(12): 1904-8.
928. Zhan L, Cao WC, de Vlas S, et al. A newly discovered Anaplasma phagocytophilum variant in rodents from southeastern China. *Vector Borne Zoonotic Dis* 2008; **8**(3): 369-80.
929. Zhan L, He J, Saren GW, et al. [Investigation on Anaplasma phagocytophilum infection in rodents from forest areas in northeastern China]. *Zhonghua Liu Xing Bing Xue Za Zhi* 2007; **28**(2): 157-9.
930. Cao WC, Zhan L, He J, et al. Natural Anaplasma phagocytophilum infection of ticks and rodents from a forest area of Jilin Province, China. *Am J Trop Med Hyg* 2006; **75**(4): 664-8.
931. Hongze Z. Investigation on the infection of small mammals with phagocytosis of phagocytes in natural foci of plague in Yunnan: Dali University; 2019.
932. Zhang L, Cui F, Wang L, et al. Investigation of anaplasmosis in Yiyuan County, Shandong Province, China. 2011; **4**(7): 568-72.
933. Lu Zhixin HL, Cai Zenglin, Lu Hui, Jin Xiantao, Zhao Zhanlin, Zhao Yugui. Investigation on the host of tsutsugamushi disease in tural foci in northeast chi. *Chin J Zoonoses* 1997; **008**(003): 222-3.
934. Zhan Lin HJ, Sa Rengaowa, Wu Xiaoming, Wang Jianbo, Zhao Qiumin, Zhang Panhe, Huang Hain, Jiang Guigui, Jiang Jiafu, Zhang Jingbo, Chu Chenyi, Gao Yan, Yang Hong, Cao Wuchun. Investigation on Aplasma phagocytophilum infection in rodents from forest areas in northeastern Chi. *Chinese Jourl of Epidemiology* 2007; **28**(2): 3.
935. Wang Zhuo WJ, Yu Miao, Feng Li, Wang Xiuhong, Xu Zhiwei, Tian Pu, Wu Yimin. Investigation of new Ehrlichia infection and sequence alysis of GroEL gene in rodents in northeast border area. *Chinese Jourl of Zoonoses* 2019; **35**(4): 330-3.
936. Xiao Fangzhen DY, Lin Daihua, Han Tengwei, Xu Guoying, Liu Jing, Liu Weijun. Study of Candidatus Neoehrlichia mikurensis in rodent in Fujiang Province and an investigation of the characteristics of its 16rR and gROEL genes. *Chinese Jourl of Pathogenic Biology* 2020; **15**(4): 5.
937. Yang Yijun WQ, Wang Zhuo, Jin Meisong, Zhang Qingbo, Xu Zhiwei, Wu Yimin. Investigation on tick-borne rickettsia in rodents collected in eastern mountains of Liaoning Province and Jilin Province in China. *Chinese sanitary insecticide* 2019; **25**(5): 4.
938. Xiao Fangzhen CM, Han Tengwei, Zhou Shuyu, Deng Yanqin. Study on mixed infection of rodents carrying tick borne pathogens in Sanming City. *Chinese Jourl of Pathogenic Biology* 2019; **14**(7): 4.
939. Li Lingli ZS, Du Chunhong, Peng Honghong, Zhao Qiumin, Dai Ke, Zhang Jiusong. Investigation on the carrying status of several bacterial pathogens of small mammals in Gongshan area of Yunn Province. *Jourl of Parasites and Medical Insects*.
940. Qunying L, Suyun C, Weilong P, Jiangling Z, Liping J, Junhua G. Analysis of Ehrlichia mikuri and its 16S rRNA gene in animals in Zhejiang Province. *Chinese Journal of Epidemiology* 2012; **33**(9): 2.
941. Li H, Jiang J, Tang F, et al. Wide distribution and genetic diversity of "Candidatus Neoehrlichia mikurensis" in rodents from China. *Appl Environ Microbiol* 2013; **79**(3): 1024-7.
942. Wang Z, Wang JW, Miao YU, et al. Investigation on Candidatus Neoehrlichia mikurensis in rodents collected in forest area in the northeastern region of China. 2019.
943. Maza L, Pezzlo MT, Bittencourt CE, Peterson EM. Rickettsia, Orientia, Ehrlichia, Anaplasma, and Coxiella: Color Atlas of Medical Bacteriology; 2020.
944. Pan Hua LS, Ma Yuhai, Tong Shide, Sun Yang. Ehrlichia like microorganisms were detected in rats

in Guangzhou suburbs. *Chin J Zoonoses* 2003; **19**(5): 3.
945. Jiang Liping ZS, Wang Zhigang, Ye Xiaodong, Meng Zhen, Cheng Suyun, Zheng Haiou, Xie Shuyun, Lu Qunying, Wang Zhen, Ling Feng, Mo Shihua. Ehrlichia and Intangible DNA Fragments Detected from Rodents in Jindong District. *Zhejiang Preventive Medicine* 2008; **20**(1): 4.
946. Zhao Qiumin WX, Zhang Panhe, Li Jianmin, Yang Hong, Wei Maoti, Zhang Xitan, Cao Wuchun. Study on compound infection of three tick borne diseases in ticks and mice. *Chinese Jourl of Epidemiology* 2005; **26**(1): 5.
947. Gao Yumin ZX, Cao Wuchun, Dai Qinghua, Zhang Panhe, Chen Zhenguang, Dai Xiaohong, Fang Liqun, Yang Hong. Detection of Ehrlichia chaffiensis in ticks and rodents by semi nested PCR. *Chin J Zoonoses* 2000; **16**(03): 25-8.
948. Zhang Yuzhen WY, Yang Weihong, Zhu Xianghui, Pi Zilin, Feng Yun, He Lifang, Zhang Hailin, Zhang Lijuan. Serological and molecular epidemiological investigation of Rickettsia in rodents in Qujing City, Yunn Province. *Chinese Jourl of Zoonoses* 2015; **31**(1): 6.
949. Chai Chengliang SJ, Lu Qunying, Ling Feng, Jiang Liping, Ge Junhua, Gu Shiping, Ye Xiaodong, Liu Helan, Chen Enfu. A Cross-sectiol study on tick -borne Rickettsiae infections among murine -like animals. *Zhejiang Preventive Medicine* 2014; **26**(7): 5.
950. Wang Zhuo WQ, Yu Miao, Zhang Qingbo, Guo Zunyuan, Feng Li, Wang Xiuhong, Wu Yimin. Serological investigation of several important tick borne pathogens in rodents in forest areas of Chi DPRK border areas. *J Microbiol* 2017.
951. Feng Li WZ, Yang Yijun, Wang Liqiang, Wang Feng, Yu Miao, Wu Yimin. Detection of Ehrlichia chaffeensis nucleic acid in rodents in northeast forest region. *Chinese Jourl of Zoonoses* 2016; **32**(4): 4.
952. Li Chan XF, Yang Zhangnv, Zhang Yun, Zhu Hanping. Investigation on the tural Infection of 5 Pathogens in Rodent like Animals in Longyou County, Zhejiang Province. *Disease surveillance* 2021; **36**(9): 5.
953. Huanglin. Investigation and Preliminary Analysis on Pathogens of Anamorphosis and Ehrlichia in Sheep and Rodents in Some Areas of Xinjiang. Shihezi University; 2013; 2013.
954. Zhuo W, Qiong W, Shenjiang Y, et al. Investigation of Ehrlichia chaffiensis infection in ticks and rodents in Changbai Mountain area and 16S rRNA sequence analysis. *Journal of Pathogen Biology* 2017; **12**(10): 5.
955. Xianghua Y, Xiaohe Z, Qingxiang N, Chaorong N, Yi L. Analysis on the results of comprehensive surveillance of rat borne diseases in the coastal areas of southern zhejiang province. *Chinese Journal of Public Health Management* 2016; (2): 3.
956. Minghui W, Riqing L, Huiping C, Peiru L, Mingde G, Can W. Investigation on infection of Leptospira chaffiensis by rodent parasitic ticks in Jinmen area in 2009. *Epidemic report* 2010; **26**(9): 134-40.
957. Hua P, Shizhong L, Yuhai M, Shide T, Yang S. Ehrlichia like microorganisms were detected in rats in Guangzhou suburbs. *Chinese Journal of Zoonoses* 2003; **19**(5): 3.
958. Fangzhen X, Yanqin D, Daihua L, et al. Investigation of New Ehrlichia Carried by Rodents in Fujian Province and Characteristics of 16S rRNA and groEL Genes. *Journal of Pathogen Biology* 2020; **15**(4): 5.
959. Chan L, Fang X, Zhangnv Y, Yun Z, Hanping Z. Investigation on the Natural Infection of 5 Pathogens in Rodent like Animals in Longyou County, Zhejiang Province. *Disease Surveillance* 2021; **36**(9): 5.
960. Qingliang Z, Huanglin, Xia Y, et al. Investigation and 16s rRNA Sequence Analysis of Intangible Rodents and Ehrlichia in Some Areas of Xinjiang. *Chinese Journal of Zoonoses* 2013; (08): 15-9.
961. Shanshan T, Yujian Z, Xinghua C, et al. Survey on Pathogens Carried by Rodents at Ulashtai Port on the Sino Mongolian Border in 2017. *Chinese Journal of Frontier Health and Quarantine* 2018; **41**(3): 3.
962. Lingli L. Investigation on the Molecular Biological Characteristics of Mosquito borne Virus Newly Found in Yunnan Province and the Carrying Situation of Rodent Pathogens: Anhui Medical University.
963. Li DM, Hou Y, Song XP, et al. High prevalence and genetic heterogeneity of rodent-borne Bartonella species on Heixiazi Island, China. *Appl Environ Microbiol* 2015; **81**(23): 7981-92.
964. Zhang L, Peng Q, Gu XL, et al. Host specificity and genetic diversity of Bartonella in rodents and shrews from Eastern China. *Transbound Emerg Dis* 2022.
965. Liu H, Han T, Liu W, Xu G, Zheng K, Xiao F. Epidemiological characteristics and genetic diversity of Bartonella species in rodents from southeastern China. *Zoonoses Public Health* 2022; **69**(3): 224-34.
966. Chunhong D, Jiaxiang Y, wintersweet C, et al. Investigation on Bartonella infection of indoor rat like animals in western Yunnan. *Disease Surveillance* 2016; (3): 5.
967. Xueyun L. Investigation and Whole Genome Sequencing Analysis of Bartonella Carried by Rodent like Animals in Guangdong Province: Southern Medical University; 2019.

968. Profit, Liping J, Zhongliang L, Kaiman Y, Zhen M, Lingling M. Phylogenetic analysis of the rpoB gene of Bartonella rat in Zhejiang Province. *Chinese Journal of Vector Biology and Control* 2011; **22**(2): 3.
969. Jieqiong M, wintersweet C, Zhongke C, Qiyong L. Prevalence of rat borne Bartonella. *Disease Surveillance* 2018; **33**(1): 8.
970. Jieqiong M. Genetic Diversity and Phylogenetic Evolution of Rattus Bartonella in China: Shandong University.
971. wintersweet C, Xiuping S, Guichang L, et al. Analysis of genetic diversity and distribution characteristics of Bartonella muris from Heixiazi Island, China. The 5th International Forum on Sustainable Control of Vector Organisms; 2014; 2014.
972. Falian Y, crowing Wc, Hui Y. Characteristics of Bartonella infection in rodents in Yunnan. *Medical animal control* 2007; **023**(004): 256-7.
973. Qifei H, Pinghang L, Jianming Z, et al. Investigation on Bartonella infection of rat like animals at Fuzhou Port. *Chinese Journal of Frontier Health and Quarantine* 2014; (6): 5.
974. Meng Y, Peng W, Hui Y, et al. Distribution and Genome Sequence of Bartonella in Rodent like Animals in Some Areas of Jiangxi Province. *Chinese Journal of Zoonoses* 2021; **37**(3): 4.
975. Ye Xi YM, Li Guowei. Investigation of Bartonella infection in rodents in Fujian Province. *Chinese Journal of Zoonoses* 2006; **022**(008): 779-81.
976. Dongmei L. Analysis of genetic diversity and distribution characteristics of Bartonella muris from Heixiazi Island, China. *The 5th International Forum on Sustainable Control of Vector Organisms* 2014.
977. Gui-Ming FU, Sun JM, Yang ZN, et al. Investigation of Bartonella infection in rats by molecular technology. 2009.
978. Castle KT, Kosoy M, Lerdthusnee K, et al. Prevalence and diversity of Bartonella in rodents of northern Thailand: a comparison with Bartonella in rodents from southern China. *Am J Trop Med Hyg* 2004; **70**(4): 429-33.
979. Li DM, Liu QY, Yu DZ, Zhang JZ, Gong ZD, Song XP. Phylogenetic analysis of Bartonella detected in rodent fleas in Yunnan, China. *J Wildl Dis* 2007; **43**(4): 609-17.
980. Ling F, Ding F, Gong ZY, et al. Investigation of Bartonella infection in rodents in Zhejiang province,China. 2014; **16**(6): 1751–9.
981. Sun JM, Liang LU, Liu QY, et al. Molecular epidemiological investigation of Bartonella infection in ticks in Zhejiang province. 2010.
982. Bai HM, Yang FL, Yang HJCJoE. Study on Bartonella species in rodents in western Yunnan, China. 2005; **26**(11): 868.
983. Ru AHCBLWNMLZFPSXLRLMX. Bartonella Species Investigated among Rodents from Shaanxi Province of China. *Biomedical and Environmental Sciences* 2020; (3): 5.
984. Shuangyan Z. Investigation and Isolation and Identification of Bartonella Infection in Rodent Animals in Heilongjiang Forest Region of China: Central South University; 2012.
985. Falian Y. Investigation of Bartonella in Yunnan Province. *Endemic Diseases Bulletin* 2005; **20**(001): 73-4.
986. Xiaomei C, Ying W, Jiemin W, Yanfei G, Huaiyuan W, Xiaolong Z. Investigation and genetic characteristics analysis of Bartonella carried by rats at Suifenhe Port in 2018. *Chinese sanitary insecticide* 2019; **25**(5): 4.
987. Fu L, peak S, Yanning L, et al. Investigation of Bartonella infection in rat like animals in Beijing. *Chinese Journal of Zoonoses* 2021; **37**(6): 5.
988. Du Chunhong YJ, Li Dongmei, Wang Xiufang, Cheng Xiaoou, Yang Guangcan, Liu Zhengxiang. Investigation of Bartonella infection in small rodents in households in western Yunnan. *Disease surveillance* 2016; (3): 5.
989. Yexi, Guowei L, Meilin Y, Wei L, Liqiong S. Investigation on the infection and gene type of Bartonella murine in coastal areas of Fujian Province. *Chinese Journal of Epidemiology* 2009; (10): 4.
990. Ju Wendong YF, Jiao Dan, Wang Yanmei, Cheng Cheng, Xu Ning, Han Xiyu, Geng Cong, Zhao Deshun, Sun Yi. Investigation of Bartonella infection in rodents at Mudanjiang airport in Heilongjiang province, China. *Chinese Jourl of Vector Biology and Control* 2017; **28**(3): 4.
991. Chen Baobao AC, Lei Yi, Fan Suoping, Li Dongmei, Sun Yangxin. Investigation of the Bartonella infection in wild rodent populations in Dingbian plague areas of Shaanxi. *Chinese Jourl of Health Inspection* 2017; **27**(15): 4.
992. Li Dongmei YD, Liu Qiyong, Gong Zhengda. Study on the prevalence of Bartonella species in rodent hosts from different environmental areas in Yunan. *Chinese Jourl of Epidemiology* 2004; **25**(11): 4.
993. Yang Weihong SX, Liang Wei, Feng Yun, Zhang Yuzhen, Zhang Yunzhi, Zhang Hailin, Li Dongmei. Investigation on rodents carrying Bartonella in residential areas of Dehong Prefecture, Yunnan Province.

Disease surveillance 2018; **33**(1): 6.
994. Wang Ying WJ, Wan Daozheng, Ci Ying, Cao Xiaomei, Wang Jing, Zhang Xiaolong. Investigation on Bartonella infection of small mammals at Alashankou port on the border between China and Kazakhstan. *Jourl of Parasites and Medical Insects* 2020; **27**(1): 5.
995. Ying B, Kosoy MY, Maupin GO, Tsuchiya KR, Gage KL. Genetic and ecologic characteristics of Bartonella communities in rodents in southern China. *Am J Trop Med Hyg* 2002; **66**(5): 622-7.
996. Ying B, YKosoy M. It is confirmed for the first time that Bartonella is endemic in Yunnan rat population in China. *Chinese Journal of Zoonoses* 2002; **18**(3): 5.
997. Wei L, Yexi, Meilin Y, Guowei L, Liqiong S. Distribution of Bartonella among rodents and regions in Xiamen. *Chinese Journal of Pathogenic Biology* 2009; **4**(8): 3.
998. Weihong Y, Xiuping S, Wei L, et al. Investigation on rodents carrying Bartonella in residential areas of Dehong Prefecture, Yunnan Province. *Disease Surveillance* 2018; **33**(1): 6.
999. Yang Weihong SX, Liang Wei, Feng Yun, Zhang Yuzhen, Zhang Yunzhi, Zhang Hailin, Li Dongmei. Investigation of tural infection status of Bartonella in house rodents in Dehong prefecture of Yunan province. *Disease surveillance* 2018; **33**(1): 6.
1000. Cao Xiaomei WY, Wang Jiemin, Gao Yanfei, Wang Huaiyuan, Zhang Xiaolong. Surveillance and genetic alysis of Bartonella in rodents at Suifenhe Port in 2018. *Chinese sanitary insecticide* 2019; **25**(5): 4.
1001. Li Fu DX, Lv Yanning, Sun Yulan, Wang Xiaomei, Li Renqing, Chen Lijuan, Wang Quanyi. Investigation of Bartonella infection in murine-like animals in Beijing, China. *Chinese Jourl of Zoonoses* 2021; **37**(6): 5.
1002. Liu Aihua CL, Zhang Caijun, Chen Fang, Shi Ming, Chen Liang, Shen Peiqing, Liu Ruwen, Jiao Jianlin, Li Malin, Bao Fukai. Detection of Borrelia burgdorferi in wild tree shrews by touchdown PCR. *Chinese Tropical Medicine* 2009; **9**(4): 3.
1003. Zhang Guobao YL, Zhang Xiaoyu, Hu Xiaoxiong. Alysis of the results of serological surveillance on 3 kinds of rat- borne diseases. *Chinese Jourl of Health Inspection* 2015; (21): 3.
1004. Dong Shanshan LY, Duan Cunjuan, Guo Ying, Shi Liyuan, Zhong Youhong, Li Dongmei, Wang Peng. Investigation of the status of Bartonella infection among rodents in Jianchuan county of Yunn, 2017. *Disease surveillance* 2019; **34**(11): 4.
1005. Ye Xi YM, Li Guowei. The investigation on the infection of Bartonellaspecies in rodent hosts in Fujian. *Chinese Jourl of Zoonoses* 2006; **022**(008): 779-81.
1006. Wang Ying WJ, Wan Daozheng, Ci Ying, Cao Xiaomei, Wang Jing, Zhang Xiaolong. Investigation on Bartonella Infection of Small Mammals at Alashankou Port on China Kazakhstan Border. *Jourl of Parasites and Medical Insects* 2020; **27**(1): 5.
1007. Bai Ying MK, GO Maupin, KL Gage, Dong Xingqi, Ma Yongkang. Discovery of Bartonella species in rodents in Yunan. *Chin J Zoonoses* 2002; **18**(3): 5.
1008. Wang Zhuo WQ, Jin Meisong, Xing Yuepeng, Yang Yijun, Wu Yimin. Investigation and Genotype Alysis of Bartonella Rodent Infection in Changbai Mountains, China. *Chinese Jourl of Vector Biology and Control* 2021; (005): 032.
1009. Yang Meng WP, Yuan Hui, Liu Xiaoqing, Zhou Haijian, Xu Xiaoqian, Xiong Changhui, Huang Xingkui, Xie Yun, Pan Huanhong. Study of Bartonella prevalence and genome sequences in rodents in some areas of Jiangxi Province. *Chinese Jourl of Zoonoses* 2021; **37**(3): 212-5.
1010. Qin XR, Liu JW, Yu H, Yu XJ. Bartonella Species Detected in Rodents from Eastern China. *Vector Borne Zoonotic Dis* 2019; **19**(11): 810-4.
1011. Yu J, Xie B, Bi GY, et al. Prevalence and diversity of small rodent-associated Bartonella species in Shangdang Basin, China. *PLoS Negl Trop Dis* 2022; **16**(6): e0010446.
1012. Li Dongmei XA, Song Xiuping, Li Qingduo, Kang Yang, Liu Qiyong. Bartonella distribution in turally infected rodent tissues. *Chinese Jourl of Vector Biology and Control* 2021; **32**(2): 7.
1013. Song Xiuping LD, Jia Lijun, Lu Liang, Wang Jun, Liu Yunyan, Jiang Yayun, Liu Qiyong. Investigation of Bartonella infection in small mammals in Inner Mongolia, China. *Chinese Jourl of Vector Biology and Control* 2015; **26**(3): 5.
1014. Rao Huaxiang YJ, Li Shoujiang, Song Xiuping, Li Dongmei. Gene polymorphisms of Bartonella species in small mammals in Maixiu tiol Forest Park in the Qinghai-Tibet Plateau, China. *Chinese Jourl of Vector Biology and Control* 2021; **32**(4): 6.
1015. Sun Jimin SX, Fu Guiming, Lu Liang, Liu Qiyong. Phylogenetic alysis of Bartonellaspp . from rodents of Zhejiang Province. *Chinese Jourl of Zoonoses* 2010; **026**(006): 532-4,45.
1016. Rao H, Li S, Lu L, et al. Genetic diversity of Bartonella species in small mammals in the Qaidam Basin, western China. *Sci Rep* 2021; **11**(1): 1735.
1017. Wang Jing SQ, Yang Yu, Liu Lijuan, Li Ming, Fu Yingqun, Zhao Gang, Ci Ying, Guo Tianyu, Zhang Xiaolong. Investigation of Bartonella in the Rat Population of Heixiazi Island on the Sino Russian

Border. *Chinese Jourl of Frontier Health and Quarantine* 2021; (044-006).
1018. Xiao Fangzhen LD, Zhou Shuxuan, Xu Guoying, Deng Yanqin. Investigation and sequence alysis on Bartonella infection in rodents in Fujian Province, China. *Chinese Jourl of Zoonoses* 2017; **33**(7): 6.
1019. Dan Y. Infection of Bartonella in Small Mammals in the Field of pestis Natural Focus in Yunnan Province and Analysis of Its Influencing Factors: Dali University; 2019.
1020. Jing W, Qi S, Yu Y, et al. Investigation of Bartonella in the Rat Population of Heixiazi Island on the Sino Russian Border. *Chinese Journal of Frontier Health and Quarantine* 2021; (044-006).
1021. Yu J, Li Q, Lu L, et al. Detection and genetic diversity of Bartonella species in small mammals from the central region of the Qinghai-Tibetan Plateau, China. *Sci Rep* 2022; **12**(1): 6996.
1022. Rao HX, Yu J, Guo P, et al. Bartonella Species Detected in the Plateau Pikas (Ochotona curzoiae) from Qinghai Plateau in China. *Biomed Environ Sci* 2015; **28**(9): 674-8.
1023. Yu J, Zhang XY, Chen YX, Cheng HB, Li DM, Rao HX. Molecular detection and genetic characterization of small rodents associated Bartonella species in Zhongtiao Mountain, China. *PLoS One* 2022; **17**(2): e0264591.
1024. Liu Q, Sun J, Lu L, et al. Detection of bartonella species in small mammals from Zhejiang Province, China. *J Wildl Dis* 2010; **46**(1): 179-85.
1025. Hao L, Yuan D, Guo L, et al. Molecular detection of Bartonella in ixodid ticks collected from yaks and plateau pikas (Ochotona curzoniae) in Shiqu County, China. *BMC Vet Res* 2020; **16**(1): 235.
1026. Ye X, Li GW, Yao ML, Luo W, Su LQ. [Study on the prevalence and genotypes of Bartonella species in rodent hosts from Fujian coastal regions]. *Zhonghua Liu Xing Bing Xue Za Zhi* 2009; **30**(10): 989-92.
1027. Lin JW, Hsu YM, Chomel BB, et al. Identification of novel Bartonella spp. in bats and evidence of Asian gray shrew as a new potential reservoir of Bartonella. *Vet Microbiol* 2012; **156**(1-2): 119-26.
1028. Jieqiong M. The genetic diversity and phylogenetic analysis of rodent-borne Bartonella species in China: Shandong University.
1029. Li Bo LS, Luo Tao, Abu Likmu Abuduresiti, Hao Qin, Wang Xinhui, Zhang Lin, Guliyai Baokaixi, Li Wei. Infection status of pathogens in Citellus undulatus in Guertu, Xinjiang. *Disease surveillance* 2020; **35**(7): 4.
1030. Ling Feng DF, Gong Zhenyu, Chen Enfu, Hou Juan, Miao Ziping, Shi Xuguang, Qin Shuwen, Li Dongmei, Liu Qiyong. Investigation of Bartonella infection in rodents in Zhejiang province, China. *Chinese Jourl of Vector Biology and Control* 2014; **25**(1): 24-7.
1031. Li Xueyun CJ, Li Congrong, Ke Bixia, Lin Wenqing, Peng Zhiqiang, Ke Changwen. Genotype characteristics of Bartonella and the infection in rodents in Guangdong Province, China. *Chinese Jourl of Zoonoses* 2018; **34**(5): 6.
1032. Song XP, Zhang HB, Liu QY, et al. Seroprevalence of Bartonella henselae and Identification of Risk Factors in China. *Biomed Environ Sci* 2020; **33**(1): 72-5.
1033. Liu Q, Eremeeva ME, Li D. Bartonella and Bartonella infections in China: from the clinic to the laboratory. *Comp Immunol Microbiol Infect Dis* 2012; **35**(2): 93-102.
1034. Huang K, Kelly PJ, Zhang J, et al. Molecular Detection of Bartonella spp. in China and St. Kitts. *Can J Infect Dis Med Microbiol* 2019; **2019**: 3209013.
1035. Zhang Y, Zhang Z, Lou Y, Yu Y. Prevalence of hemoplasmas and Bartonella species in client-owned cats in Beijing and Shanghai, China. *J Vet Med Sci* 2021; **83**(5): 793-7.
1036. Liu M, Biville F. Managing iron supply during the infection cycle of a flea borne pathogen, Bartonella henselae. *Front Cell Infect Microbiol* 2013; **3**: 60.
1037. Liu YY, Zhao LS, Song XP, et al. Development of fluorogenic probe-based and high-resolution melting-based polymerase chain reaction assays for the detection and differentiation of Bartonella quintana and Bartonella henselae. *J Microbiol Methods* 2017; **138**: 30-6.
1038. Sun YQ, Wang T, Zhang YY, et al. Human infections with neglected vector-borne pathogens in China: A systematic review. *Lancet Reg Health West Pac* 2022; **22**: 100427.
1039. Zhang JB, Wen BH, Chen ML, Li LL, Qiu L, Niu DS. [Development of a quantitative real-time polymerase chain reaction for detecting Bartonella henselae]. *Zhonghua Liu Xing Bing Xue Za Zhi* 2007; **28**(3): 277-81.
1040. Yang XR, Liu QY, Cui BY, Wang LX, Peng ZH, Ren DS. [Using direct enzyme linked immunosorbent assay for the detection of IgG antibody on Bartonella henselae among healthy people in Changping, Beijing]. *Zhonghua Liu Xing Bing Xue Za Zhi* 2007; **28**(7): 688-91.
1041. Zhang Y, Zhang ZL, Yin JY, et al. [Sero-epidemiological investigation on Rickettsia typhi, Bartonella henselae and Orientia tsutsugamushi in farmers from rural areas of Tianjin, 2007 - 2009]. *Zhonghua Liu Xing Bing Xue Za Zhi* 2011; **32**(3): 256-9.

1042. Zhang L, Cui F, Wang L, et al. Investigation of anaplasmosis in Yiyuan County, Shandong Province, China. *Asian Pac J Trop Med* 2011; **4**(7): 568-72.
1043. Rao Huaxiang YJ, Li Shoujiang, Song Xiuping, Li Dongmei. Gene polymorphisms of Bartonella species in small mammals in Maixiu tiol Forest Park in the Qinghai-Tibet Plateau, China. *Chinese Jourl of Vector Biology and Control* 2021.
1044. Hu H, Liu Z, Fu R, Liu Y, Ma H, Zheng W. Detection and phylogenetic analysis of tick-borne bacterial and protozoan pathogens in a forest province of eastern China. *Acta Trop* 2022; **235**: 106634.
1045. Shanshan D, Yanping L, Cunjuan D, et al. Survey of Bartonella infection in rodents in Jianchuan County, Yunnan Province in 2017. *Disease Surveillance* 2019; **34**(11): 4.
1046. Lin Guohua YC. Investigation of Bartonella infection in rat like animals in Longhai City. *Chinese Jourl of Zoonoses* 2010.
1047. Huang Qifei LP, Zhang Jianming, Wang Yuping, Huang Yeneng, Li Xiaoning, Zhou Tianxi, Wei Ling, Wu Qing. Investigation of Bartonella infection in rodents in Fuzhou harbor. *Chinese Jourl of Frontier Health and Quarantine* 2014; (6): 5.
1048. Yexi. Investigation on the infection and gene type of Bartonella murine in coastal areas of Fujian Province. *Chinese Journal of Epidemiology* 2009; (010): 000.
1049. Xiuping S, Xiuping S, DongMei L, et al. Investigation of Bartonella infection in rodents in Inner Mongolia. *The 5th International Forum on Sustainable Control of Vector Organisms* 2014.
1050. Malania L, Bai Y, Osikowicz LM, et al. Prevalence and Diversity of Bartonella Species in Rodents from Georgia (Caucasus). 2016: 466-71.
1051. Hsieh JW, Tung KC, Chen WC, et al. Epidemiology of Bartonella Infection in Rodents and Shrews in Taiwan. 2010.
1052. Tsai YL, Chuang ST, Chang CC, Kass PH, Chomel BB. Bartonella species in small mammals and their ectoparasites in Taiwan. *Am J Trop Med Hyg* 2010; **83**(4): 917-23.
1053. Hsieh JW, Tung KC, Chen WC, et al. Epidemiology of Bartonella infection in rodents and shrews in Taiwan. *Zoonoses Public Health* 2010; **57**(6): 439-46.
1054. Jiyipong T, Jittapalapong S, Morand S, Raoult D, Rolain JM. Prevalence and genetic diversity of Bartonella spp. in small mammals from Southeastern Asia. *Appl Environ Microbiol* 2012; **78**(23): 8463-6.
1055. Luo Wei YX, Yao Meilin, Li Guowei, Su Liqiong. Study on the distribution of Bartonella species in rodent hosts from different areas in Xiamen. *Chinese Jourl of Pathogenic Biology* 2009; **4**(8): 3.
1056. Ye Xi LG, Yao Meilin, Luo Wei, Su Liqiong. Study on the prevalence and genotypes of Bartonella species in rodent hosts from Fujian coastal reg"on. *Chinese Jourl of Epidemiology* 2009; (010): 000.
1057. Song Xiuping LQ, Lu Liang, Zhao Wei, Li Guichang, Li Dongmei, Sun Jimin, Huang Ruting. Isolation and sequence alysis of Bartonella in small mammals in Hain province. *Chinese Jourl of Vector Biology and Control* 2010; (2): 3.
1058. He Xiutian XY, Yuan Dongbo, Yang Aiguo, Fan Xiaohu, Tan Xiong, Zhong Yeqing, Hao Lili. PCR Detection and Phylogenetic Alysis of Bartonella spp.and Aplasma spp.in Ixodid Ticks Collected from Yak and Plateau Pika in Songpan County of Sichuan Province. *Jourl of Animal Husbandry and Veteriry Medicine* 2020; **51**(6): 9.
1059. Yang W, Song X, Wei L, et al. Investigation of natural infection status of Bartonella in house rodents in Dehong prefecture of Yunnan province. 2018.
1060. Lin Guohua YC. Investigation of Bartonella infection in rat like animals in Longhai City. *Chinese Jourl of Zoonoses* 2010; **26**(010): 989-.
1061. Du Chunhong YJ, Zuo Shuqing, Li Dongmei, Wang Xiufang, Cheng Xiaoou, Liu Zhengxiang, Yang Guangcan. Detection of pathogens carried by small mammals in residential areas of western yunnan. *Chinese Jourl of Zoonoses* 2016; **32**(7): 9.
1062. Yang Weihong SX, Liang Wei, Feng Yun, Zhang Yuzhen, Zhang Yunzhi, Zhang Hailin, Li Dongmei. Investigation of tural infection status of Bartonella in house rodents in Dehong prefecture of Yunnan province. *Disease surveillance* 2018; **33**(1): 6.
1063. Lin JW, Hsu YM, Chomel BB, et al. Identification of novel Bartonella spp. in bats and evidence of Asian gray shrew as a new potential reservoir of Bartonella. 2012; **156**(1-2): 119-26.
1064. Song XP, Liu QY, Liang LU, et al. Isolation and sequence analysis of Bartonella in small mammals in Hainan province. 2010; **21**(2): 131-3.
1065. Zhitao L, Dexing L, Jian C, et al. Surveillance and Analysis of Small Mammals and Their Surface Parasites and Pathogens Carried in Different Port Areas of Zhongshan Port from 2020 to 2022. 2022.
1066. Tsai YL. Bartonella and Babesia species in mammals and their ectoparasites in Taiwan:

University of California, Davis.; 2011.
1067. Weihong Y, Xiuping S, Wei L, et al. Investigation on rodents carrying Bartonella in residential areas of Dehong Prefecture, Yunnan Province. *Disease Surveillance* 2018; **33**(1): 6.
1068. Yao XY, Liu H, Sun J, et al. Epidemiology and Genetic Diversity of Bartonella in Rodents in Urban Areas of Guangzhou, Southern China. *Front Microbiol* 2022; **13**: 942587.
1069. Jian R, Ren Q, Xue J, et al. Genetic diversity of Bartonella infection in residential and field rodents in Hebei, China. *Front Microbiol* 2022; **13**: 1039665.
1070. Lin JW, Chen CY, Chen WC, Chomel BB, Chang CCJJoMM. Isolation of Bartonella species from rodents in Taiwan including a strain closely related to 'Bartonella rochalimae' from Rattus norvegicus. 2008; **57**(12): 1496-501.
1071. Chun-Hong DU, Yin JX, Dong-Mei LI, et al. Investigation of Bartonella infection in small rodents in households in western Yunnan. 2016.
1072. Liu Q, Eremeeva ME, Li DJCi, microbiology, diseases i. Bartonella and Bartonella infections in China: from the clinic to the laboratory. 2012; **35**(2): 93-102.
1073. Tsai Y-L, Chomel BB, Chang C-C, et al. Bartonella and Babesia infections in cattle and their ticks in Taiwan. 2011; **34**(2): 179-87.
1074. Qifei H. Investigation on Bartonella infection of rat like animals at Fuzhou Port: Fujian Medical University.
1075. Rao Huaxiang YJ, Li Shoujiang, Song Xiuping, Li Dongmei. Gene polymorphisms of Bartonella species in small mammals in Maixiu tiol Forest Park in the Qinghai-Tibet Plateau, China. *Chinese Jourl of Vector Biology and Control* 2020.
1076. Li D-M, Hou Y, Song X-P, et al. High prevalence and genetic heterogeneity of rodent-borne Bartonella species on Heixiazi Island, China. 2015; **81**(23): 7981-92.
1077. Bai Heming YF, Yang Hui, Zhang Qing. Study on Bartonella species in rodents in western Yunnan, China. *Chinese Jourl of Epidemiology* 2005; **26**(11): 868-70.
1078. Zhuo W, Qiong W, Jinmeisong, Yuepeng X, Yijun Y, Yimin W. Investigation and Genotype Analysis of Bartonella Rodent Infection in Changbai Mountains, China. *Chinese Journal of Vector Biology and Control* 2021; (005): 032.
1079. Meng Y, Huanhong P, Peng W, et al. Distribution and Genome Sequence of Bartonella in Rodent like Animals in Some Areas of Jiangxi Province. *Chinese Journal of Zoonoses* 2021; **37**(3): 212-5.
1080. Weihong Y, Xiuping S, Wei L, et al. Investigation of natural infection status of Bartonella in house rodents in Dehong prefecture of Yunnan province. 2018; **33**(1): 20-5.
1081. Li D, Yang W, Li Q, et al. High prevalence and genetic variation of Bartonella species inhabiting the bats in southwestern Yunnan. 2021; **29**(9): 1245.
1082. Hong AC, Bao CB, Wen L, et al. Bartonella species investigated among rodents from Shaanxi Province of China. 2020; **33**(3): 201-5.
1083. Ye Xi YM, Li Guowei, Luo Wei, Su Liqiong. Study onthe prevalence andgenotypes of Bartonella species in Suncusmurinus fromFujiancoastal region. *Chinese Jourl of Zoonoses* 2010; **26**(06): 601-3.
1084. Zhao Zhiliang GW, Wang Jiaole, Wang Yuan, Liu Fang, Gao Xiaoqiang, Wang Li. The first detection of rat-borne Bartonella in Lanzhou Zhongchuan Intertiol Airport. *Chinese Jourl of Frontier Health and Quarantine* 2019; (3): 3.
1085. Yang Falian BH, Yang Hui, Yu Binbin. Isolation, identification and sequence analysis of Bartonella from Lancang, Yunnan. *Modern Preventive Medicine* 2007; **34**(19): 3.
1086. Li DM, Liu QY, Yu DZ, Zhang JZ, Gong ZD, Song XPJJoWD. Phylogenetic analysis of Bartonella detected in rodent fleas in Yunnan, China. 2007; **43**(4): 609-17.
1087. Liu H, Han T, Liu W, et al. Epidemiological characteristics and genetic diversity of Bartonella species in rodents from southeastern China. 2022; **69**(3): 224-34.
1088. Ying B, Kosoy MY, Maupin GO, Tsuchiya KR, Gage KLJTAjotm, hygiene. Genetic and ecologic characteristics of Bartonella communities in rodents in southern China. 2002; **66**(5): 622-7.
1089. Su Q, Chen Y, Wang B, et al. Epidemiology and genetic diversity of zoonotic pathogens in urban rats (Rattus spp.) from a subtropical city, Guangzhou, southern China. 2020; **67**(5): 534-45.
1090. Lin J-W, Chen C-Y, Chen W-C, Chomel BB, Chang C-CJJomm. Isolation of Bartonella species from rodents in Taiwan including a strain closely related to 'Bartonella rochalimae'from Rattus norvegicus. 2008; **57**(12): 1496-501.
1091. Liu Q, Sun J, Lu L, et al. Detection of Bartonella species in small mammals from Zhejiang Province, China. 2010; **46**(1): 179-85.
1092. Rao H, Li S, Lu L, et al. Genetic diversity of Bartonella species in small mammals in the Qaidam Basin, western China. 2021; **11**(1): 1-10.

1093. Hao L, Yuan D, Guo L, et al. Molecular detection of Bartonella in ixodid ticks collected from yaks and plateau pikas (Ochotona curzoniae) in Shiqu County, China. 2020; **16**(1): 1-9.
1094. Han HJ, Li ZM, Li X, et al. Bats and their ectoparasites (Nycteribiidae and Spinturnicidae) carry diverse novel Bartonella genotypes, China. 2022; **69**(4): e845-e58.
1095. Song X, Li D, Jia L, et al. Investigation of Bartonella infection in small mammals in Inner Mongolia, China. 2015; **26**(3): 233-7.
1096. Yu J, Zhang X-Y, Chen Y-X, Cheng H-B, Li D-M, Rao H-XJPo. Molecular detection and genetic characterization of small rodents associated Bartonella species in Zhongtiao Mountain, China. 2022; **17**(2): e0264591.
1097. Yu J, Xie B, Bi G-Y, et al. Prevalence and diversity of small rodent-associated Bartonella species in Shangdang Basin, China. 2022; **16**(6): e0010446.
1098. Jian R, Ren Q, Xue J, et al. Genetic diversity of Bartonella infection in residential and field rodents in Hebei, China. 2022; **13**.
1099. Zhang L, Peng Q, Gu XL, et al. Host specificity and genetic diversity of Bartonella in rodents and shrews from Eastern China. 2022.
1100. Shi Jinfeng GC, Zhang Kun, Ju Wendong, Wang Lixiang, Wang Hongxia, Li Zhenjiang, Huang Xiaoming, Cheng Cheng. Molecular biological evidence of bartonella washoensis carried by chipmunks first found at tongjiang port, heilongjiang province. *Chinese Jourl of Vector Biology and Control* 2017; **28**(5): 3.
1101. von Loewenich FD, Seckert C, Dauber E, et al. Prosthetic valve endocarditis with Bartonella washoensis in a human European patient and its detection in red squirrels (Sciurus vulgaris). 2019; **58**(1): e01404-19.
1102. Ulan Tuya GW, Yin Xuhong, Cao Minzhi, Guo Shengchun, Wang Qiang, Ariben Jiri Gala, Liu Meiqin. Survey on Borrelia bacteria in rodents from Bayannur, Inner Mongolia Autonomous Region, China. *Chinese Jourl of Vector Biology and Control* 2018; **29**(3): 3.
1103. Zhang Fang WW, Li Lu. Borrelia Burgdorferii Infection and Genotypes in Ticks and Rodents Collected in Qinghai Province. *PLA Jourl of Preventive Medicine* 2016; **34**(6): 4.
1104. Zhang F, Gong Z, Zhang J, Liu Z. Prevalence of Borrelia burgdorferi sensu lato in rodents from Gansu, northwestern China. *BMC Microbiol* 2010; **10**: 157.
1105. Zhang Lin SY, Geng Zhen, Hou Xuexia, Hao Qin. Investigation on Borrelia burgdorferi in Rodents from Huzhu, Zeku and Qilian Counties, Qinghai Province. *Chinese Jourl of Vector Biology and Control* 2015; **26**(2): 3.
1106. Guo Qiang TF, Kabnur Mamuti, Gao Yanfei, Zhao Danyun, Chen Weijun, Qi Jun. Investigation on rats and pathogens at Huoerguosi port regions in Xinjiang, 2016. *Chinese Jourl of Frontier Health and Quarantine* 2017; **40**(4): 3.
1107. Wang Shuangqing ZB, Zhang Jianmin, Cao Guoping, Zhong Jianyue, Yu Zhangyou. Investigation on Borrelia burgdorferi in rats captured at Kaihua county in Quzhou of Zhejiang province. *Chinese Jourl of Vector Biology and Control* 2018; **29**(6): 4.
1108. Decai Z, Hongyuan, Zhongqiu Z, et al. Borrelia burgdorferi Isolated from Ticks and Mice in Liaoning Province. *Chinese Journal of Vector Biology and Control* 1992; **3**(1): 19-.
1109. Hall H, Zhaoqing J, Cheng H, Zhanwei G, Zhiting W, Jian H. Study on Borrelia burgdorferi carried by Rattus rufus in Tiereketilin pastoral area, Habahe, Xinjiang. *Chinese Journal of Zoonoses* 2000; **16**(3): 1.
1110. Xia Z, Yongxue L, Lanli, Qihui W. A preliminary investigation on the main host animals and transmission vectors of borrelia burgdorferi in 5 cities of guangxi. *Journal of Pathogen Biology* 2019; **14**(11): 4.
1111. Zhiling Z, Shiping G, Yi H, Liping J, Qunying L. DNA fragment of Borrelia burgdorferi was detected in mice in Anji County. *Chinese Journal of Health Laboratory Technology* 2010; (11): 2.
1112. Liping J, Qunying L, Zhongliang L, Lei Z, Fuxing W, Zheng Z. OspA gene of Borrelia burgdorferi was detected in rodents in Zhejiang Province. *Chinese Journal of Vector Biology and Control* 2012; **23**(2): 3.
1113. Cunjuan D, Ying G, Xuexia H, et al. Investigation on Borrelia burgdorferi Infection in Rodents and Vector Ticks in Jinghong City, Yunnan Province. *Disease Surveillance* 2019; **34**(3): 5.
1114. Hall H, Zhaoqing J, Zhiting W, Cheng H, Changlin L. Preliminary Study on Borrelia burgdorferi Carried in Mole Microtus. *Medical animal control* 1999; **15**(10): 525-6.
1115. Liping J. Application of 5S ~ 23S rRNA and D shaving gene of Borrelia burgdorferi in mouse detection in Zhejiang Province. *Chinese Journal of Vector Biology and Control* 2014; (25): 253.
1116. Chenyi C, Jing H, Jianbo W, et al. Infection of Lyme disease spirochetes in ticks and rats and their genotyping in Daxing'an Mountains. *Chinese Journal of Epidemiology* 2006.
1117. Xiao-Ping Y, Li-Juan L, Yan-He T, Zhen L, Ba-Te. Sequence Analysis of Positive Genes of

Rodent and Tick Pathogens at Alashankou Port, China Kazakhstan Border. China Association of Inspection and Quarantine; China Academy of Inspection and Quarantine Sciences; 2015; 2015.
1118. road As, Liya L, Xiaojing M, et al. Investigation on Borrelia burgdorferi carried by rodents in some areas of northern Xinjiang. *China Animal Quarantine* 2022; (007): 039.
1119. Weijun L, Fangzhen X, Sixian L, Tengwei H, Shuda Z, Yanqin D. Investigation on Borrelia burgdorferi Infection of Rodents in 5 Counties (Cities) of Fujian Province.
1120. Xiaoping Y. Investigation on Borrelia burgdorferi Carried by Rodents in Alashankou Port Area, Xinjiang. *Chinese Journal of Frontier Health and Quarantine* 2016; **39**(4): 3.
1121. Mantang H, Zhaoqing J. Preliminary Study on Borrelia burgdorferi Carried in Mole Microtus. *Chinese Journal of Pest Control* 1999; **15**(10): 2.
1122. Zhansen D, Chunsheng W, Xiujun Y, et al. Detection of Borrelia burgdorferi infection in wild rats and domestic animals in Jilin Province. *Chinese Journal of Zoonoses* 2003; **19**(4): 3.
1123. Chu CY, Jiang BG, Liu W, et al. Presence of pathogenic Borrelia burgdorferi sensu lato in ticks and rodents in Zhejiang, south-east China. *J Med Microbiol* 2008; **57**(Pt 8): 980-5.
1124. Hou X, Xu J, Hao Q, Xu G, Geng Z, Zhang L. Prevalence of Borrelia burgdorferi sensu lato in rodents from Jiangxi, southeastern China region. *Int J Clin Exp Med* 2014; **7**(12): 5563-7.
1125. Chu CY, Liu W, Jiang BG, et al. Novel genospecies of Borrelia burgdorferi sensu lato from rodents and ticks in southwestern China. *J Clin Microbiol* 2008; **46**(9): 3130-3.
1126. Wu Q, Liu Z, Wang J, et al. Pathogenic analysis of Borrelia garinii strain SZ isolated from Northeastern China. *Parasit Vectors* 2013; **6**: 177.
1127. Takada N, Masuzawa T, Ishiguro F, et al. Lyme disease Borrelia spp. in ticks and rodents from northwestern China. *Appl Environ Microbiol* 2001; **67**(11): 5161-5.
1128. Chu CY, He J, Wang JB, et al. [Investigation on Borrelia burgdorferi sensu lato in ticks and rodents collected in Da Xing-An Mountains Forest areas of China]. *Zhonghua Liu Xing Bing Xue Za Zhi* 2006; **27**(8): 681-4.
1129. Shih CM, Chao LL. Lyme disease in Taiwan: primary isolation of Borrelia burgdorferi-like spirochetes from rodents in the Taiwan area. *Am J Trop Med Hyg* 1998; **59**(5): 687-92.
1130. Wan K, Zhang Z, Wang H, Hou X. [Preliminary investigation on reservoir hosts of Borrelia burgdorferi in China]. *Wei Sheng Yan Jiu* 1999; **28**(1): 7-9.
1131. Shih CM, Chang HM, Chen SL, Chao LL. Genospecies identification and characterization of Lyme disease spirochetes of genospecies Borrelia burgdorferi sensu lato isolated from rodents in Taiwan. *J Clin Microbiol* 1998; **36**(11): 3127-32.
1132. Li YX, Wang S, Zeng X. [Isolation and Identification of Borrelia burgdorferi sensu lato in Leye county, Guangxi province]. *Zhonghua Liu Xing Bing Xue Za Zhi* 2008; **29**(12): 1269-70.
1133. Zuo SY, Tang K, Li Y, et al. [DNA detection and sequence analysis of Borrelia burgdorferi sensu lato in rodents from Helongjiang and forest region]. *Zhonghua Liu Xing Bing Xue Za Zhi* 2012; **33**(6): 643-4.
1134. Shih CM, Chao LL. Genetic analysis of the outer surface protein C gene of Lyme disease spirochaetes (Borrelia burgdorferi sensu lato) isolated from rodents in Taiwan. *J Med Microbiol* 2002; **51**(4): 318-25.
1135. Chao LL, Liu LL, Shih CM. Prevalence and molecular identification of Borrelia spirochetes in Ixodes granulatus ticks collected from Rattus losea on Kinmen Island of Taiwan. *Parasit Vectors* 2012; **5**: 167.
1136. Zhang ZF, Wan KL, Zhang JS. [Studies on epidemiology and etiology of Lyme disease in China]. *Zhonghua Liu Xing Bing Xue Za Zhi* 1997; **18**(1): 8-11.
1137. Wang L, Wan K, Liu S, et al. [The first discovery of endemic Lyme disease in Shandong province]. *Zhonghua Liu Xing Bing Xue Za Zhi* 2000; **21**(4): 292-4.
1138. Wang Y, Li S, Wang Z, Zhang L, Cai Y, Liu Q. Prevalence and Identification of Borrelia burgdorferi Sensu Lato Genospecies in Ticks from Northeastern China. *Vector Borne Zoonotic Dis* 2019; **19**(5): 309-15.
1139. Hao Q, Hou X, Geng Z, Wan K. Distribution of Borrelia burgdorferi sensu lato in China. *J Clin Microbiol* 2011; **49**(2): 647-50.
1140. Zhai B, Niu Q, Yang J, et al. Identification and molecular survey of Borrelia burgdorferi sensu lato in sika deer (Cervus nippon) from Jilin Province, north-eastern China. *Acta Trop* 2017; **166**: 54-7.
1141. He Z, Jiang B, Huang L, et al. High Diversity and Prevalence of Borrelia burgdorferi sensu lato in Wildlife Hosts, Domestic Animals, and Ticks in Yunnan Province, Southwestern China. *Front Microbiol* 2022; **13**: 876079.
1142. Chu CY, Jiang BG, He J, et al. Genetic diversity of Borrelia burgdorferi sensu lato isolates from Northeastern China. *Vector Borne Zoonotic Dis* 2010.
1143. Chu CY, Jiang BG, He J, et al. Genetic diversity of Borrelia burgdorferi sensu lato isolates

from Northeastern China. *Vector Borne Zoonotic Dis* 2011; **11**(7): 877-82.
1144. Dong Y, Zhou G, Cao W, et al. Global seroprevalence and sociodemographic characteristics of Borrelia burgdorferi sensu lato in human populations: a systematic review and meta-analysis. *BMJ Glob Health* 2022; **7**(6).
1145. Zhan L, Chu CY, Zuo SQ, et al. Anaplasma phagocytophilum and Borrelia burgdorferi in rabbits from southeastern China. *Vet Parasitol* 2009; **162**(3-4): 354-6.
1146. Stark JH, Li X, Zhang JC, et al. Systematic Review and Meta-analysis of Lyme Disease Data and Seropositivity for Borrelia burgdorferi, China, 2005–2020. *Emerg Infect Dis* 2022; **28**(12): 2389-97.
1147. Hao Q, Geng Z, Hou XX, et al. Seroepidemiological investigation of lyme disease and human granulocytic anaplasmosis among people living in forest areas of eight provinces in China. *Biomed Environ Sci* 2013; **26**(3): 185-9.
1148. Shih CM, Chao LL. An OspA-based genospecies identification of Lyme disease spirochetes (Borrelia burgdorferi) isolated in Taiwan. *Am J Trop Med Hyg* 2002; **66**(5): 611-5.
1149. Lumeng M. Establishment of diagnostic methods and epidemiological investigation of lyme disease and brucellosis: Shihezi University; 2016.
1150. Zhihai H. Molecular epidemiology of Borrelia burgdorferi infection in host animals and vector ticks in western Yunnan: Dali University; 2019.
1151. Bao Qinghan JL, Xu Zhichun, Yu Manchu, Hu Yan, Yu Hongnu. Detection of Borrelia burgdorferi in rat liver and spleen samples by nested PCR. *Zhejiang Preventive Medicine* 2014.
1152. Wan Kanglin ZZ, Jia Wenchun, Zhang Shuqin, Zhang Xiaojie, Song Hongzhang, Mu Jingwen, Zhang Jinsheng. Lyme disease spirochetes were isolated from brown back Ping and Chipmunks in Xiaoxing'anling, Heilongjiang Province. *Chinese Jourl of Vector Biology and Control* 1997; **8**(3): 3.
1153. Pan Liang YE, Zhang Zhefu, Wan Kanglin, Zhang Jinsheng, Zheng Li, Liu Jinyong, Chen Foyun, Chen Zhangming, You Tusheng. Four strains of Lyme disease pathogens were isolated from Ixodes granulosus and social rats. *Chinese Jourl of Vector Biology and Control* 1992.
1154. Fu Weiming WZ, Yang Jun, Ju Wendong. Investigation on the main animal hosts and vectors of Lyme disease spirochete in the border area between Chi and Russia in Heilongjiang Province. *Port Health Control* 2009; (1).
1155. Wu Xiaoming WZ, Wang Jianhua, Li Daojun, Du Yong. Investigation on Rodents in Lyme Disease tural Focus in Weihe Area, Heilongjiang Province. *Chinese Jourl of Vector Biology and Control* 1991; **2**(6): 3.
1156. Yu Deshan GZ, Jiang Jianxiang, Hao Qin, Chen Jianhua, Wang Peng. Investigation on Lyme Disease tural Foci in Diebu County, Gansu Province. *Chinese Jourl of Vector Biology and Control* 2009; **20**(1): 2.
1157. Pan Liang YE, Zhang Zhefu, Wan Kanglin, Zhang Jinsheng, Zheng Li, Liu Jinyong, Chen Foyun, You Tusheng, Chen Zhangming. Investigation on Lyme disease of ticks and mice in Fujian Province and isolation of 4 strains of Lyme disease spirochetes for the first time. *Chinese Jourl of Epidemiology* 1992.
1158. Pan Liang YE, Zhang Zhefu, Wan Kanglin, Zhang Jinsheng, Zheng Li, Liu Jinyong, Chen Foyun, Chen Zhangming, You Tusheng. Investigation of lyme disease in fujian province. *Chinese Jourl of Public Health* 1992; **11**(5): 3.
1159. Huang Yaoping ZH, Cai Songgen, Shi Lishuang, Pan Liang, Huang Xiuhao, Yu Enshu. Discovery and investigation of lyme disease in southern fujian. *Chin J Zoonoses* 1993; **9**(001): 46-7.
1160. Wan Kanglin ZZ, Li Haohao, Hao Jiguang, Xia Xianzhong, Hu Xingwen, Zhang Jinsheng, Hou Xuexia. Leptospira Lyme disease was isolated from Sichuan white bellied mice. *Chinese Jourl of Vector Biology and Control* 1991; **2**(6): 2.
1161. Wan Kanglin GY, Xu Shie, Zhang Zhefu, Pan Linxiang, Xie Linchong, Luo Jingping. Borreliaburgdorferi Isolated from Rattus edwardsi and Rattus norvegicus in Fengxi Protective tural Regionof East Guangdong Province. *Chinese Jourl of Vector Biology and Control* 1999.
1162. Shi Shuzhen LZ, Sun Yi, Gong Zhanwei, Zhang Jijun. Investigationonsmall animals infectionof Borrelia burgdorferi in Diebuforest. *Chinese sanitary insecticide* 2004; **10**(4): 220-1.
1163. Ye Haibo WD, Xu Jia, Yun Xiaoyun, Zhang Xiaolong, Qin Fengkui, Chen Jianchao, Fang Yong, Yao Lisi. Borrelia burgdorferi isolated from rat in Yachang forest farm of Guangxi Province. *Chinese Jourl of Vector Biology and Control* 2017; **28**(4): 4.
1164. Zeng Xia LY, Lan Li, Wang Qihui. Prelimiry investigation of rats and other hosts and the vectors of Borrelia burgdorferi in 5 cities in Guangxi Province. *Chinese Jourl of Pathogenic Biology* 2019; **14**(11): 4.
1165. Chen Wushen WK, Hao Ruifeng, He Jianfeng, He Li, Luo Huiming, Zeng Hanwu, Zhang Jinsheng, Zhang Zhefu. Borrelia burgdorferi was Isolated in Guangdong. *Chinese Jourl of Vector Biology and Control* 1998; (04): 44-5.

1166. Chen Wushen HR, Luo Huiming, Zheng Kui, Chen Tong, He Jiaqiang, Li Weisheng. Discovery of Lyme Disease Foci in Xingang Forest Farm, Zhaoqing, Guangdong. *Chin J Zoonoses* 2000; **16**(2): 2.
1167. Xie Linchong WK, Guo Yan, Xu Shie, Zhang Zhefu, Pan Linxiang, Chen Shenben. Investigation on Lyme Disease tural Foci in Meizhou City, Guangdong Province. *Chinese Jourl of Pathogenic Biology* 2009; **4**(8): 4.
1168. Chen Wushen LH, He Jianfeng, Zheng Kui, Li Wei, Chen Ran. An Survey on Host and Vector of Lyme Disease in Guangdong Province. *Chinese Jourl of Vector Biology and Control* 2000; **11**(3): 4.
1169. Liu Weijun XF, Lin Sixian, Han Tengwei, Zhou Shuyu, Deng Yanqin. An investigation of rodents infected with Borrelia burgdorferi in five counties (cities) of Fujian province, Chi. *Chinese Jourl of Vector Biology and Control* 2019.
1170. Wang Huayong GZ, Li Liqin, Hou Xuexia, Yang Yusong, Hao Qin, Man Yongzhen, Wang Quanyi. Investigation on reservoir hosts and vectors infected by Borrelia burgdorferi in Miyun area. *Chinese Jourl of Vector Biology and Control* 2009.
1171. Zhang Decai ZZ, Sun Guangjiu, Zhang Zhefu, Zhang Jinsheng, Dong Shude, Cai Donghao, Zhuang Linchun, Wu Lipao, Liu Zhichen, Pan Tiehou, Wang Dongwu, Wang Jianwu, Wang Zechun. Investigation on Lyme disease in mountainous areas of Eastern Liaoning. *Chinese Jourl of Epidemiology* 1996.
1172. Zhang Decai HY, Zhang Zhongqiu, Sun Guangjiu, Dong Shude, Zhang Zhefu, Zhang Jinsheng, Hou Xuexia. Borrelia burgdorferi was isolated from ticks and rats in Liaoning Province. *Chinese Jourl of Vector Biology and Control* 1992.
1173. Liu Zengchang SY, Shi Shuzhen, Gong Zhanwei, Zhang Jijun, Xu Rongman, Lu Baolin. Investigation on tural foci of Lyme disease in Lazikou forest area. *Chin J Zoonoses* 2004; **20**(5): 3.
1174. Zhao Minghui XT, Zhang Qiang, Hu Ting, Wang Jianjun, Liu Kaiming, Liao Yun. Detection and Gene Sequence Alysis of Leptospira Infection in Rodents at Jiangxi Port and tiol Inspection and Supervision Area - Zhao Minghui. *Chinese Jourl of Zoonoses* 2016; **32**(7): 5.
1175. Xiao Guoling ZL, Xu Hongtao, Xu Jiankun, Yan Xuling, Liu Guangzhong, Zhang Zhefu, Zhang Jinsheng, Hou Xuexia, Zhang Baotang, Hu Jiahui. Leptospira Lyme disease was isolated from Rattus norvegicus for the first time in Jiangsu Province. *Jiangsu Medicine* 1993; **19**(12): 1.
1176. Geng Zhen HX, Guo Jianhua, Huang Xin, Wang Chunsheng, Wang Bo, Yang Xiujun, Hao Qin. Epidemiological investigation on Lyme disease in Changbai and Tonghua county, Jilin province. *Chinese Jourl of Vector Biology and Control* 2010; (6): 4.
1177. Huang Hain DZ, He Jing, Wu Xiaoming, Jiang Guigui, Gao Yan, Zhao Qiumin, Wang Yufu, Cao Wuchun. Investigation on Borrelia burgdorf eri infection in ticks and animal from a forest area of Jilin province. *Chinese Jourl of Zoonoses* 2006; **22**(8): 4.
1178. Liu Fuqiang HQ, Gao Lidong, Geng Zhen, Zhan Zhifei, Hou Xuexia, Zhang Hong, Wang Maowu, Li Junhua, Guo Shouheng, Dai Defang, Zeng Ge, Wan Kanglin. A prelimiry study on the prevalence of lyme disease in two mountainous villages in hun province. *Disease surveillance* 2008; **23**(6): 4.
1179. Yawei W. Investigation on the New Anamorphosis Carried by Domestic Animals and Vector Ticks in Northern China: PLA Academy of Military Medical Sciences.
1180. Chu Chenyi HJ, Zhao Qiumin, Zhang Panhe, Wu Xiaoming, Jiang Guigui, Gao Yan, Li Hongmei, Huang Hain, Zhan Lin, Cao Wuchun. Molecular epidemiological studies on Borrelia burgdorf eri in rodents collected in the forest area of several provinces and autonomous regions of China. *Chinese Jourl of Zoonoses* 2006; **22**(9): 4.
1181. Zhang L, Zhu X, Hou X, et al. Prevalence and prediction of Lyme disease in Hainan province. *PLoS Negl Trop Dis* 2021; **15**(3): e0009158.
1182. Fu Guiming SJ, Yang Zhangnv, Yang Tianci, Si Guojing, Pang Weilong, Gong Zhenyu, Liu Qiyong. Detection of Bartonella murine by molecular biological techniques. *Chinese Jourl of Vector Biology and Control* 2009; **20**(5): 3.
1183. Tan Xueyun L, Shi Guosheng, Jiang Bin. Monitoring and alysis of rodent situation in Fengdu County from 1997 to 1999. *Practical Preventive Medicine* 2000; **7**(4): 1.
1184. Jiang Liping LQ, Li Zhongliang, Zhang Lei, Wang Fuxing, Zhang Zheng. OspA gene of Borrelia burgdorferi was detected in mice in Zhejiang Province. *Chinese Jourl of Vector Biology and Control* 2012; **23**(2): 3.
1185. Jiang Liping ZS, Mo Shihua, Ye Xiaodong, Zhan Li, Wang Zhigang. Detection and sequence alysis of Borrelia burgdorferi sensu lato D in rat-shape animals from Zhejiang province. *Chinese Jourl of Vector Biology and Control* 2008; **19**(5): 461-3.
1186. Meng Zhen JL, Li Zhongliang, Ying Kaiman, Ling Feng, Lu Qunying. Lyme disease spirochete infection was detected in rats in central Zhejiang Province. *Chinese Jourl of Health Inspection*

2010; (10): 3.
1187. Xia Z, Shusheng W, Tao Z, Shengli B, Yongdong Z. Genetic Characterization of Four Strains Borrelia Burgdorferi Isolated in China. *Microbiology and immunology* 2004; **002**(001): 6-9.
1188. Fang L-Q, Liu K, Li X-L, et al. Emerging tick-borne infections in mainland China: an increasing public health threat. 2015; **15**(12): 1467-79.
1189. Masuzawa TJJJID. Terrestrial distribution of the Lyme borreliosis agent Borrelia burgdorferi sensu lato in East Asia. 2004; **57**(6): 229-35.
1190. Chu C-Y, Liu W, Jiang B-G, et al. Novel genospecies of Borrelia burgdorferi sensu lato from rodents and ticks in southwestern China. 2008; **46**(9): 3130-3.
1191. Zhou J, Xu M, Guo W, et al. Corynebacterium lizhenjunii sp. nov., isolated from the respiratory tract of Marmota himalayana, and Corynebacterium qintianiae sp. nov., isolated from the lung tissue of Pseudois nayaur. *Int J Syst Evol Microbiol* 2021; **71**(5).
1192. Meng Zhen JL, Li Zhongliang, Ying Kaiman, Ling Feng, Lu Qunying. Lyme disease spirochete infection was detected in rats in central Zhejiang Province. *Chinese Jourl of Health Inspection* 2019; **35**(11): 6.
1193. Lu Zhiyu YX, Yu Yonghui, Ding Sheng, Xia Guanghui, Zhou Dongsheng, Xiong Xiaolu, Xu Jianmin, Jiao Jun. Molecular epidemiological investigation of Rickettsiae in rodents in Jiangxi Province. *Chinese Jourl of Zoonoses* 2021; **37**(7): 8.
1194. Control CJoVBa. Investigation on tural infection of rodent-borne pathogens in rodent populations in Urad port area, Inner Mongolia, Chi in 2015. 2018; **29**(3): 2.
1195. Liu S, Jin D, Lan R, et al. Escherichia marmotae sp. nov., isolated from faeces of Marmota himalayana. *Int J Syst Evol Microbiol* 2015; **65**(7): 2130-4.
1196. Study on the Population Composition and Pathogens Carried by Rat like Animals at Daxie Port. *Travel Medical Science* 2006; **12**(3): 4.
1197. Yongkang T. In 1989, pestis F-1 specific antibody was detected from host animals in historical pestis areas in Zhejiang Province. *Zhejiang Preventive Medicine and Disease Surveillance* 1989.
1198. Goto K, Jiang W, Zheng Q, et al. Epidemiology of Helicobacter infection in wild rodents in the Xinjiang-Uygur autonomous region of China. *Curr Microbiol* 2004; **49**(3): 221-3.
1199. Hu S, Jin D, Lu S, et al. Helicobacter himalayensis sp. nov. isolated from gastric mucosa of Marmota himalayana. *Int J Syst Evol Microbiol* 2015; **65**(Pt 6): 1719-25.
1200. Chen Rongfu LJ, Zhu Weiguo, Wang Bingn, Xu Jingfu. Investigation on historical foci of leptospirosis in Lishui City. *Zhejiang Preventive Medicine* 2002; **14**(10): 1.
1201. Kuanshen Z. Investigation and alysis of Leptospira in rodents in Xianju County from 1987 to 2000. *Chinese Jourl of Vector Biology and Control* 2003; **14**(3): 180-.
1202. Yang Wenying CM, Yang Xiangdong, Pang Jingwen, Du Chunhong. Leptospira serovar and geographic distribution of rodents in Yunnan. *Chinese Jourl of Vector Biology and Control* 1994; **5**(6): 6.
1203. Lisi Y. Preliminary study on rodents, body surface parasites and their pathogens in the port area adjacent to Changbai Mountain of China and Korea: PLA Academy of Military Medical Sciences; 2012.
1204. Shanshan T. Monitoring and Control of Rat Body and Its Surface Parasites at Wulashtai Port: Xinjiang medical university.
1205. Jigui Z. Report on the first isolation of Leptospira javanica from a grey musk shrew. *Chinese Jourl of Epidemiology* 1983.
1206. Zhang Yunshu QW. Detection of serum type 1 (Lp-1) antibody of Legionella pneumophila from wild rats. *Chin J Zoonoses* 1988; **4**(02): 45-.
1207. Liu Fuqiang GL, Wu Zigui, Guo Shouheng, Zhang Hong, Dai Defang, Chen Lizhang. Study on Leptospira Infectionof Rats in Hunan Province in 2006 *Practical Preventive Medicine* 2008; **15**(1): 3.
1208. Fu Guiming GZ, Zhao Zhiya. Surveillance of Leptospirosis in Zhejiang Province in 1997. In: Control CJoVBa, editor.; 1998; 1998.
1209. Hua Gaorong LY, Yin Zhen, Liu Jie. Surveillance of Leptospirosis in Huaiyuan County, Anhui Province in 2005. *Anhui Jourl of Preventive Medicine* 2008; **14**(1): 2.
1210. Han HJ, Wen HL, Liu JW, et al. Pathogenic Leptospira Species in Insectivorous Bats, China, 2015. *Emerg Infect Dis* 2018; **24**(6): 1123-6.
1211. Su Q, Chen Y, Wang B, et al. Epidemiology and genetic diversity of zoonotic pathogens in urban rats (Rattus spp.) from a subtropical city, Guangzhou, southern China. *Zoonoses Public Health* 2020; **67**(5): 534-45.
1212. Yang CW, Pan MJ, Wu MS, et al. Leptospirosis: an ignored cause of acute renal failure in Taiwan. *Am J Kidney Dis* 1997; **30**(6): 840-5.
1213. Liu Fuqiang GL, Chen Lizhang, Zhang Hong, Dai Defang, Wu Zigui, Zeng Ge. Alysis of

leptospira host animal infection in hunan province in 2007. *Contemporary Medicine (Academic Edition)* 2008; (05): 153-5.
1214. Wang Zhendong WS, Liu Lijuan, Yang Yu, Li Ming, Guo Tianyu, Fu Yingqun, Hou Yong, Sun Xiaohong, Xu Baoliang, Wang Jing. The infection status of Leptospira in rodents on the Heixiazi island of Heilongjiang province, China, in 2011. *Chinese Jourl of Preventive Medicine* 2013; **47**(6): 4.
1215. Zhang Cuicai ZT, Xu Jianmin, Jiang Xiugao, Qiu Haiyan. Epidemiological investigation and identification of isolated strains of leptospirosis rodents in Jiangxi Province from 2016 to 2018. *Chinese Jourl of Zoonoses* 2019; **35**(12): 5.
1216. Gu Lili ZY, Hu Yanmei, Wang Jun. Pathogenic Leptospira was isolated from the kidney of grey musk shrew for the first time in Anhui Province. *Chinese Jourl of Epidemiology* 2007; **28**(9): 929-30.
1217. Qin Jincai WY, Pu Cong, Gao Jiyuan. Leptospira lai first found in Beijing. *Progress in Microbiology and Immunology* 1996; **24**(3): 2.
1218. Xu Guoying LD, Xiao Fangzhen, Han Tengwei, Liu Weijun, Zhou Shuxuan, Liu Jing, Deng Yanqin. Survey on infectious status of leptospira in rats of Fujian Province. *Chinese Jourl of Disease Control* 2018; **22**(6): 4.
1219. Liu Ying LS, Ma Qing, Wang Dingming, Tang Guangpeng, Yao Guanghai, Zhou Jingzhu, Chen Zhenghong. Study on MLVA Typing of Leptospira Strains Isolated from Apodemus agrarius in Guizhou Province. *Chinese Jourl of Pathogenic Biology* 2017; **12**(1): 5.
1220. Li Shijun WD, Zhang Cuicai, Li Xiuwen, Tian Kecheng, Liu Ying, Tang Guangpeng, Jiang Xiugao. Sequence Alysis and Gene Species Identification of 16S rR Gene of Apodemus agrarius Leptospira Isolated in Guizhou Province in 2011. *Chinese Jourl of Zoonoses* 2012.
1221. Xiuzhen W. Prelimiry report on surveillance of leptospirosis in Tianzhu County, Guizhou Province. *Jourl of Preventive Medicine Information* 1993; (S1): 1.
1222. Wang Yanxia YA, Kang Kai, Sun Jianwei. Correlation between leptospirosis and host animals in Hen Province. *Chinese Jourl of Zoonoses* 2013; **29**(10): 3.
1223. Chen Zhongmiao ZK. Investigation on seasol fluctuation trend of leptospira carried by Apodemus agrarius. *Disease surveillance* 1999; (004): 014.
1224. Xiao Pu CX, Xiao Wei, Zhu Hongwei, Liu Rongqiang, Wang Zhengliang. Report on Surveillance Results of Leptospirosis in Shuangfeng County from 2005 to 2007. *Practical Preventive Medicine* 2008; **15**(6): 3.
1225. Zeng Yixue GZ, Liu Lunguang, Huang Ziying, Zhu Xiaoping, Zeng Linzi, Liu Xuecheng. Alysis of surveillance results of leptospirosis in sichuan province from 2005 to 2008. *Modern Preventive Medicine* 2009; **36**(24): 3.
1226. Guo Zongqi OB, Huang Ziying, Huang Kailong, Wei Min, Lin Hong, Yang Dejun, Liu Lisheng, Liu Lunguang, Yan Zhixiong, Zhang Lin, Du Juan, Xu Zhuqing. Investigation on the infectious source of leptospirosis in Jiangyou City, Sichuan Province. *Modern Preventive Medicine* 2003; **30**(1): 18-9.
1227. Chen Zhongmiao ZK. Investigation and alysis of Leptospira infection in rodents in Xianju County. *Chinese Jourl of Health Inspection* 1999; (05): 46.
1228. Huang Ziying GZ, Wu Guohui. Leptospira batavi subgroup was found in Apodemus agrarius in Chongqing. *Jourl of Preventive Medicine Information* 1999; (04): 210.
1229. Xiao X, Zhou SH, Jiang N, et al. First record of Leptospira and Blastocystis infections in captive flying squirrels (Trogopterus xanthipes) from Enshi County, China. *Acta Trop* 2019; **197**: 105065.
1230. Sun Xiaohong ZL, Zhang Jianchun, Wei Lian, Zhang Xiaolong, Gao Xu, Zhou Lin, Zhang Xun, Wang Jing. Investigation on the Pathogen of Rat borne Diseases in Chi Vietm Hekou Laojie Border Area. *Chinese Jourl of Frontier Health and Quarantine* 2010; **33**(2): 4.
1231. Zhang C, Xu J, Zhang T, et al. Genetic characteristics of pathogenic Leptospira in wild small animals and livestock in Jiangxi Province, China, 2002-2015. *PLoS Negl Trop Dis* 2019; **13**(6): e0007513.
1232. Yalin W, Lingbing Z, Hongliang Y, et al. High prevalence of pathogenic Leptospira in wild and domesticated animals in an endemic area of China. *Asian Pac J Trop Med* 2011; **4**(11): 841-5.
1233. Wang ZD, Wang SS, Liu LJ, et al. [The infection status of Leptospira in rodents on the Heixiazi island of Heilongjiang province, China,in 2011]. *Zhonghua Yu Fang Yi Xue Za Zhi* 2013; **47**(6): 510-3.
1234. Gong Zhenyu JL, Mei Lingling, Chen Enfu, Yang Jiezhe, Lin Junfen, Sun Songwen, Fu Guiming. Surveillance of Leptospirosis in Zhejiang Province from 1997 to 2000. *Chinese Jourl of Preventive Medicine* 2002; **3**(1): 3.
1235. Guo Zongqi HZ, Liu Lunguang, Liu Lisheng, Ouyang Bing, Wei Min, Lin Hong, Zhu Xiaoping, Zhang Lin, Yuan Heng, Du Juan, Xu Zhuqing. Surveillance of Leptospirosis Infectious Sources in Sichuan Province from 1993 to 2001. *Jourl of Preventive Medicine Information* 2002; **18**(5): 3.
1236. Lei Jinbao ZA, Chen Zhongbing, Jiang Liping, Wang Lihong, Lan Feng. Surveillance of

Leptospira in Longyou County in 2006. *Jourl of Preventive Medicine Information* 2007; **18**(006): 503-4.
1237. Wang Qianxin FM. Alysis of Leptospirosis Epidemic Situation and Surveillance in Changshan County from 1991 to 2006. *Chinese Jourl of Vector Biology and Control* 2008; **19**(4): 3.
1238. Li Qing LH, Fan Zhenguang, Gu Lili. Dymic surveillance of leptospirosis in anhui province from 2005 to 2009. *Chinese Jourl of Disease Control* 2011; **15**(8): 3.
1239. Ying Kaiman ZM. Surveillance of Leptospirosis Host Animals in Pan'an County, Zhejiang Province, 2007-2009. *Disease surveillance* 2011.
1240. Li Qing ZZ. Dymic surveillance and alysis of leptospirosis epidemic situation in Anhui Province from 2015 to 2019. *Chinese sanitary insecticide* 2021; **27**(1): 3.
1241. Ma XJ, Gong XQ, Xiao X, et al. Detection of Leptospira interrogans in Hedgehogs from Central China. *Vector Borne Zoonotic Dis* 2020; **20**(6): 427-31.
1242. Hu W, Lin X, Yan J. Leptospira and leptospirosis in China. *Curr Opin Infect Dis* 2014; **27**(5): 432-6.
1243. Song N, Zhang W, Ding Y, et al. Preliminary Characterization of Dog Derived Pathogenic Strains of Leptospira interrogans Serovar Australis in Nanchang of Jiangxi Province, China. *Front Vet Sci* 2020; **7**: 607115.
1244. He P, Sheng YY, Shi YZ, et al. Genetic diversity among major endemic strains of Leptospira interrogans in China. *BMC Genomics* 2007; **8**: 204.
1245. Li Z, Zhang Y, Du Z, Xin X, Ye Q, Xu Y. Comparative proteomic analysis of Leptospira interrogans serogroup Icterohaemorrhagiae human vaccine strain and epidemic isolate from China. *Arch Microbiol* 2022; **204**(8): 460.
1246. Li SJ, Zhang CC, Li XW, et al. Molecular typing of Leptospira interrogans strains isolated from Rattus tanezumi in Guizhou Province, Southwest of China. *Biomed Environ Sci* 2012; **25**(5): 542-8.
1247. Shao JW, Wei YH, Yao XY, et al. Pathogenic Leptospira Species Are Widely Disseminated among Wild Rodents in Urban Areas of Guangzhou, Southern China. *Microorganisms* 2022; **10**(5).
1248. Shi M, Liang Z, Du X, Jiang X, Nie Y. [A molecular epidemiological investigation on Leptospira interrogans serovar hebdomadis and australia in China]. *Zhonghua Liu Xing Bing Xue Za Zhi* 2000; **21**(2): 91-3.
1249. Xu G, Qiu H, Liu W, et al. Serological and molecular characteristics of pathogenic Leptospira in rodent populations in Fujian Province, China, 2018-2020. *BMC Microbiol* 2022; **22**(1): 151.
1250. Zhou J, Huang X, He H, et al. [Epidemiological study on leptospirosa infection of host animals and healthy population in flood areas]. *Zhong Nan Da Xue Xue Bao Yi Xue Ban* 2009; **34**(2): 99-103.
1251. Xu JM, Jiang XG, Li XW, Zhang Y, Wang J, Xiong CH. [Molecular typing on Leptospira interrogans isolates from Jiangxi province, by pulsed-field gel electrophoresis]. *Zhonghua Liu Xing Bing Xue Za Zhi* 2010; **31**(4): 434-7.
1252. Ping, He, Yue-Ying, et al. Genetic diversity among major endemic strains of Leptospira interrogans in China. 2007; **8**(1): 204-.
1253. Zhang Tiansheng YZ, Luo Gang. Surveillance of Leptospirosis in Jiangyou City from 2002 to 2006. *Jourl of Preventive Medicine Information* 2007; (06): 50-2.
1254. Wang Zhendong WS, Liu Lijuan, Yang Yu, Li Ming, Guo Tianyu, Fu Yingqun, Hou Yong, Sun Xiaohong, Xu Baoliang, Wang Jing. The infection status of Leptospira in rodents on the Heixiazi island of Heilongjiang province, China,in 2011. *Chinese Jourl of Preventive Medicine* 2013; **47**(6): 4.
1255. Qi Teng ML, Wang Limao, Duan Yongjun, Luo Zhidanba, Luo Longze, Liu Jian, Yang Jun, Duan Fenggang, Chen Jiankang, Wang Peng, Nima, Zhaxi Duoji. Alysis of investigation results of pestis tural focus in Batang County, Sichuan Province in 2011. *Parasitic Diseases and infectious diseases* 2012; **10**(2): 4.
1256. Arts PM. Epidemiological survey of leptospirosis in Changde area, Hun Province. *Chinese Jourl of Epidemiology* 1983.
1257. Zheng Jigui CZ, Xiong Jianming, Feng Changyu. Detection of Leptospira from Himalayan musk deer Qu body. *Chinese Jourl of Epidemiology* 1989.
1258. Liu Ying CZ, Li Shijun, Yao Guanghai, Huang He, Ma Qing, Zhou Jingzhu, Tang Guangpeng, Wang Dingming. Alysis on epidemic characteristics of leptospirosis in Guizhou Province from 2010 to 2014. *Chinese Jourl of Zoonoses* 2016; **32**(11): 5.
1259. Liu Rongqiang WZ, Liu Guoxian, Zhu Feiyue, Zhu Jiangyan. Investigation and alysis of Leptospira infection in host animals in Shuangfeng County from 2009 to 2013. *Practical Preventive Medicine* 2014.
1260. Deng Yuanling SX, Zhu Yongjian, Wang Pingyuan, Zhou Zhishan. Surveillance of Leptospirosis in Qingyuan City, Guangdong Province in 2007. *Occupation and health* 2008; **24**(21): 3.
1261. Jiang N, Dai B, Yan Z, et al. [Research on the recombinant plasmid pDJH2 of L. interrogans

serovar lai: sequencing and alignment with other known bacterial Omp sequence]. *Hua Xi Yi Ke Da Xue Xue Bao* 1996; **27**(4): 341-7, 53.
1262. Xiong Zihui L. Epidemiological surveillance report of leptospirosis in Liuyang County from 1964 to 1983. *Public Health in China* 1993; **9**(8): 2.
1263. Qiwen J. Investigation on Leptospira carried by rodents in Liuyang County. *Chinese Jourl of Rodent Control* 1988.
1264. Li Feng CS. Investigation on rodent population distribution and parasites in Funiu Mountains of nyang. *Chinese sanitary insecticide* 2015; (5): 3.
1265. Fan Rusheng ZY, Tan Jianming, Pan Qinmeng, Huang Qin, Li Xiaosong, Mo Guanying. Baseline Survey on Taxonomy and Population Fluctuation of Murine Animals and Their Ectoparasites;Forecast Research on the Related Diseases of tural Focus in Maoming Por. *Chinese Jourl of Vector Biology and Control* 2005.
1266. Wang Y, Lu L, Lan R, et al. Isolation and characterization of Listeria species from rodents in natural environments in China. *Emerg Microbes Infect* 2017; **6**(6): e44.
1267. Zhang M, Zhao ZT, Wang XJ, et al. Genetic variants of Orientia tsutsugamushi in domestic rodents, northern China. *Emerg Infect Dis* 2013; **19**(7): 1135-7.
1268. Zheng L, Bi Z, Kou Z, Yang H, Zhang L, Zhao Z. Genotype diversity and distribution of Orientia tsutsugamushi in scrub typhus patients and rodents in Shandong, northern China. *Infect Genet Evol* 2015; **36**: 126-30.
1269. Cao Min WZ, Lu Nianhong, Huang Huadong, Guo Hengbin, Wang Changjun, Zhang Xing, Tang Jiaqi. Investigation on rodents infected with Orientia tsutsugamushi in Sanjie, Anhui Province. *Chinese Jourl of Zoonoses* 2011; **27**(8): 4.
1270. Wang Shiquan LL, Guo Kangyun, Yu Enshu. Investigation on the epidemic focus of tsutsugamushi disease in winter. *Fujian Medical Jourl* 1983; (6).
1271. Li Chunming CS, Guo Yixiu, Kong Lingyi, Shi Jian, Jin Xiusheng, Zhou Liqiang, Ji Xueyong. Confirmation of tsutsugamushi epidemic focus in taihang mountain area, hebei province. *Jourl of Disease Control* 2002.
1272. Pan Zhiming ZD, Chen Xiaoshuang, Luo Lei, Guo Rongtong, Gao Yufan, Tang Ximei, Liu Xiaoning. Investigation on Distribution of Rodents, Fleas and Zoonoses in Guangzhou City. Jourl of Tropical Medicine; 2005; 2005.
1273. Zhou Peisheng KC, Chen Long, Li Guoxiang, Huang Jialiang. Investigation on tural foci of tsutsugamushi disease in southwest Guangxi. *Chinese Jourl of Vector Biology and Control* 1991; **2**(3): 4.
1274. Wang Junli LP, Duan Aixiang, Xu Yunzhen, Yao Yunchao, Meng Fanbin, Liu Yuhua, Wang Yuqin. Investigation Report on Tsutsugamushi Disease in Mengyin County. *Jourl of Linyi Medical College* 1996.
1275. Lu Zhixin HL, Cai Zenglin, Jin Xiantao, Zhao Zhanlin, Chen Tingcai, Li Zhongyi, Tao Zengguang. Hunchun: a newly discovered tural focus of tsutsugamushi disease. *Chinese Jourl of Epidemiology* 1994; **15**(1): 3.
1276. Li Guangmi TQ, Deng Desheng, Luo Zhili. Discovery of tural infection of rickettsia tsutsugamushi in south chi swamp vole. *Chin J Zoonoses* 1985; **1**(01): 56-.
1277. Wu Guanghua GH, Yu Mingming. Investigation on tural Foci of Three Kinds of Tsutsugamushi Disease in East Chi. *Chinese Jourl of Epidemiology* 2000; **21**(1): 3.
1278. Zhou Peilong LL, Zhu Lide, Mai Zhenquan, Cai Hengzheng. Investigation on rat mites and scrub typhus in southern mountainous areas of Hun Province. *Postgraduate Medical Information* 1983; (1).
1279. Lu Zhiyu YX, Yu Yonghui, Ding Sheng, Xia Guanghui, Zhou Dongsheng, Xiong Xiaolu, Xu Jianmin, Jiao Jun. Molecular epidemiological investigation of Rickettsiae in rodents in Jiangxi Province. *Chinese Jourl of Zoonoses* 2021.
1280. Zhang Qian LY, Wu Xiaoming, Zhao Qiumin, Zhang Panhe, Yang Hong, Cao Wuchun. Investigation on tural infection of mice with Orientia tsutsugamushi in some areas of Inner Mongolia and Xinjiang, China. *Chinese Jourl of Epidemiology* 2006; **27**(6): 475-8.
1281. Yuan TT, Ma L, Jiang BG, et al. First Confirmed Infection of Candidatus Rickettsia Tarasevichiae in Rodents Collected from Northeastern China. *Vector Borne Zoonotic Dis* 2020; **20**(2): 88-92.
1282. Liu W, Li H, Lu QB, et al. Candidatus Rickettsia tarasevichiae Infection in Eastern Central China: A Case Series. *Ann Intern Med* 2016; **164**(10): 641-8.
1283. Jia N, Zheng YC, Jiang JF, Ma L, Cao WC. Human infection with Candidatus Rickettsia tarasevichiae. *N Engl J Med* 2013; **369**(12): 1178-80.
1284. Shao JW, Yao XY, Song XD, et al. Molecular detection and genetic diversity of Rickettsia spp. in pet dogs and their infesting ticks in Harbin, northeastern China. *BMC Vet Res* 2021; **17**(1): 113.

1285. Hanwha. Preliminary Study on Pathogens of Tick borne Infectious Diseases in Some Natural Scenic Spots in China: PLA Academy of Military Medical Sciences; 2015.
1286. Cheng C, Wendong J, Dan J, et al. Investigation on new tick borne diseases at Heilongjiang Port. *Chinese Journal of Frontier Health and Quarantine* 2015; (3): 6.
1287. Yadi F. Surveillance of chirickettsia infection in new tarasevi based on sentinel hospitals: Academy of Military Medical Sciences.
1288. Chen Jinjin TF, Wang Xiaoqin, Zhang Zaizhong, Chu Chenyi, Wang Ning, Zhang Panhe, Fang Liqun, Zhou Haisheng. Investigation of rodent borne pathogens in scenic areas of Beijing. *Jourl of the Academy of Military Medical Sciences* 2020; (2): 9.
1289. Fu Xiuping ZJ, Shen Xiaojing, Luan Mingchun, Li Menglei, Zhang Lijuan. Investigation on Rickettsia infection in rats in Xinyang area of Hen Province. *Chinese Jourl of Epidemiology* 2007.
1290. Chang Binggong ZJ, Tian Xiaodong, Cai Jun, Lu Zhenzhi. Epidemiological Study on Spotted Fever Sera of Three Farms in Qiongzhong District, Hain Province. *Chinese Jourl of Vector Biology and Control* 1999.
1291. He Jianfeng ZK, Li Wei, Luo Huiming, Li Linghui, Bi Dezeng, Zhang Yuanfu, Chang Binggong. Investigation on the tural foci of spotted fever group rickettsia in guangdong province. *Chinese Jourl of Epidemiology* 2003: 广东省医药卫生科研项目.
1292. Zhou L, Fan M, Chen J, Cai H, Zhou F, Zhu H. [Analysis of fatty acid composition of spotted fever group rickettsiae isolated in China by gas chromatography]. *Wei Sheng Wu Xue Bao* 1993; **33**(4): 290-6.
1293. Zhang H, Yang H, Zhang PHJCJoVB, Control. Spotted Fever Group Rickettsia DNA was Detected in Wild Rodent and Tick in Dali, Yunnan Province, China. 2004.
1294. Zhang YZ, Wang Y, Yang WH, et al. Seroprevalence and molecular biological investigation of commonly known Rickettsiaspecies in rodents in Qujing Prefecture,Yunnan Province,China. 2015.
1295. Wang Z, Miao YU, Yang SJ, et al. Epidemiological survey of spotted fever group rickettsia in rodents in northeastern China. 2015.
1296. Wang Z, Wang R, Zhou DZ, et al. Investigation on tick-borne Rickettsia and its coinfections in rodents collected in forest area in the Northeastern China. 2015.
1297. Guo Wenxiu GY, Tian Feng. Investigations on pathogens of medical vectors in Manzhouli port areas，2016. *Chinese Jourl of Frontier Health and Quarantine* 2017; **40**(6): 3.
1298. Chang Binggong ZJ, Tian Xiaodong, Cai Jun, Lu Zhenzhi. Epidemiological Study on Spotted Fever Sera of Three Farms in Qiongzhong District, Hain Province. In: Control CJoVBa, editor. 全国四体; 1998; 1998.
1299. Shi Jian ZL, Chen Suliang, Li Chunming, Kong Lingyi, Zu Wengang, Liu Wenyan, Jin Xiusheng, Dong Hui, Guo Yixiu, Li Guiyin, Zhang Yali. Investigation on the Vector and Host of Tsutsugamushi Disease in an Epidemic Focus in Taihang Mountain, Hebei Province. *Chinese Jourl of Vector Biology and Control* 2000; **11**(6): 462-4.
1300. Chen Zhenguang CM, Zhong Jianping, Bi Dezeng, Zhang Panhe. Detection of Spotted Fever Group Rickettsia in Rodents and Ticks in Ninghua Forest Area of Fujian Province by PCR/RFLP. *Travel Medical Science* 2002; **008**(002): 30-2.
1301. Yuan Qinghong YX. Detection and alysis of rat spotted fever Rickettsia gene in Luxi County, Yunn Province. *Chinese Tropical Medicine* 2020; (2): 4.
1302. Xu N, Gai W, Zhang Y, et al. Confirmation of Rickettsia conorii Subspecies indica Infection by Next-Generation Sequencing, Shandong, China. *Emerg Infect Dis* 2021; **27**(10): 2691-4.
1303. Song S, Chen C, Yang M, et al. Diversity of Rickettsia species in border regions of northwestern China. *Parasit Vectors* 2018; **11**(1): 634.
1304. Gu XL, Wang R, Zhou CM, et al. Natural Mediterranean Spotted Fever Foci, Qingdao, China. *Emerg Infect Dis* 2022; **28**(12): 2524-7.
1305. Zhang YY, Sun YQ, Chen JJ, et al. Mapping the global distribution of spotted fever group rickettsiae: a systematic review with modelling analysis. *Lancet Digit Health* 2022.
1306. Cheng X, Jin Y, Lao S, et al. Multispacer Typing (MST) of Spotted Fever Group Rickettsiae Isolated from Humans and Rats in Chengmai County, Hainan Province, China. *Trop Med Health* 2014; **42**(3): 107-14.
1307. Chen Z, Chen M, Zhong J, Bi D. Using PCR/RFLP to detect spotted fever group rickettsia in ticks and rodents collected in Ninghua, Fujian province. *Zhonghua Yu Fang Yi Xue Za Zhi* 2002; **36**(2): 106-8.
1308. Li J, Kelly P, Guo W, et al. Molecular detection of Rickettsia, Hepatozoon, Ehrlichia and SFTSV in goat ticks. 2020; **20**: 100407.
1309. Teng Z, Zhao N, Ren R, et al. Human Rickettsia felis infections in Mainland China. *Front Cell*

Infect Microbiol 2022; **12**: 997315.
1310. Zhang J, Lu G, Kelly P, et al. First report of Rickettsia felis in China. *BMC Infect Dis* 2014; **14**: 682.
1311. Fang W, Yao L, Cao X, et al. First Molecular Detection of Rickettsia felis-Like Organism in Eulaelaps stabularis from the Changbai Mountain Area of China. *J Parasitol* 2015; **101**(5): 514-9.
1312. Zhang L, Zhang J, Hu M, Feng X. [Severe encephalitis caused by infection of Rickettsia felis: a case report]. *Zhonghua Wei Zhong Bing Ji Jiu Yi Xue* 2021; **33**(4): 491-3.
1313. Hu Jingshe MY, Xie Qiyi, Li Baofeng, Luo Zhaohua. Investigation report on an epidemic of murine typhus in Tongxian County, Beijing. *Jourl of Public Health and Disease Control* 1983.
1314. Yang Changqing HY, Li Shufeng, Zhang Yan, Jing Qingzhong, Jin Muzi. Pathogen investigation of rodents in the western ground crossings of inner Mongolia. *Chinese Jourl of Frontier Health and Quarantine* 2020; **24**(6): 7.
1315. Chai Chengliang SJ, Lu Qunying, Ling Feng, Jiang Liping, Ge Junhua, Gu Shiping, Ye Xiaodong, Liu Helan, Chen Enfu. A Cross-sectiol study on tick -borne Rickettsiae infections among murine − like animals. *Zhejiang Preventive Medicine* 2014; **26**(7): 5.
1316. Province EDoHaEPSoH. Rickettsia mossii, the pathogen of endemic typhus in our province. *Hen Medicine* 1981.
1317. Hu Jingshe MY, Xie Qiyi, Li Baofeng, Luo Zhaohua. Investigation report on an epidemic of murine typhus in Tongxian County, Beijing. *Jourl of Public Health and Disease Control* 1983; (4): 13-7.
1318. Yang X, Chen ML, Wen BH, et al. [Detection of Rickettsia prowazekii by quantitative real-time PCR]. *Zhonghua Liu Xing Bing Xue Za Zhi* 2006; **27**(11): 963-7.
1319. Ya HX, Zhang HL, Zhou JH, et al. [An outbreak of endemic typhus in Baoshan city, Yunnan province]. *Zhonghua Liu Xing Bing Xue Za Zhi* 2011; **32**(1): 47-50.
1320. Lyu Y, Shen Y, Hu CY, et al. The first reported outbreak of an undetermined species of human infection with spotted fever group Rickettsia in Lu'an, China. *Acta Trop* 2021; **223**: 106072.
1321. Feng HM, Chen TS, Lin BH, et al. Serologic survey of spotted fever group rickettsiosis on Hainan Island of China. *Microbiol Immunol* 1991; **35**(9): 687-94.
1322. Ai CX. [Survey of natural infection of ticks with Rickettsia of the spotted fever group in the northwestern section of Xinjiang Province]. *Zhonghua Liu Xing Bing Xue Za Zhi* 1983; **4**(2): 103-4.
1323. He JF, Zheng K, Li W, et al. [Study on spotted fever group Rickettsiae in Guangdong province]. *Zhonghua Liu Xing Bing Xue Za Zhi* 2003; **24**(8): 700-3.
1324. Zhang JZ, Fan MY, Bi DZ. Detection of spotted fever group rickettsiae in ticks and rodents by polymerase chain reaction technique in People's Republic of China. *Acta Virol* 1995; **39**(5-6): 263-7.
1325. Zhang JZ, Fan MY, Bi DZ. [The application of PCR to epidemiological study on spotted fever group rickettsiae]. *Zhonghua Liu Xing Bing Xue Za Zhi* 1995; **16**(1): 25-8.
1326. Zhang JZ, Fan MY, Bi DZ, Cui WF, Han YF. Genotypic identification of three new strains of spotted fever group rickettsiae isolated in China. *Acta Virol* 1996; **40**(4): 215-9.
1327. Jiao Y, Wen B, Chen M, Niu D, Zhang J, Qiu LJAotNYAoS. Analysis of Immunoprotectivity of the Recombinant OmpA of Rickettsia heilongjiangensis. 2005; **1063**(Dec): p.261-5.
1328. Zhang Yun WG, Deng Xiaozhao, Wang Zhongcan, Cao Guangwen. Relationship between geographical landscape and epidemic characteristics of tsutsugamushi disease in southeast coastal areas. *Public Health in China* 2010; (2): 2.
1329. Long Zhimei LJ, Huang Jialiang, Yu Xinglong, Zhao Chunsheng, Jiang Kanghua, Lu Zhenfu, Yan Aiwu. A prelimiry study on the relationship between rat like animals and human diseases in the south chi sea. *Chin J Zoonoses* 1996; **12**(3): 3.
1330. Chen Min FM, Bi Dezeng, Zhang Jianzhi, Chen Xiangrui, Chen Zhenguang, Huang Yaoping. Detection, Isolation and Identification of Spotted Fever Group Rickettsia in Fujian Province. *Chin J Microbiol Immunol* 1997; **017**(006): 433-7.
1331. Zhao S, Yang M, Jiang M, et al. Rickettsia raoultii and Rickettsia sibirica in ticks from the long-tailed ground squirrel near the China-Kazakhstan border. *Exp Appl Acarol* 2019; **77**(3): 425-33.
1332. Sentausa E, El Karkouri K, Robert C, Raoult D, Fournier PE. Sequence and annotation of Rickettsia sibirica sibirica genome. *J Bacteriol* 2012; **194**(9): 2377.
1333. Gui Z, Cai H, Qi DD, et al. Identification and genetic diversity analysis of Rickettsia in Dermacentor nuttalli within inner Mongolia, China. *Parasit Vectors* 2022; **15**(1): 286.
1334. Liu QH, Walker DH, Zhou GF. Serologic survey for antibodies to Rickettsia sibirica in Inner Mongolia, People's Republic of China. *Ann N Y Acad Sci* 1990; **590**: 237-42.
1335. Zhang L, Jin J, Fu X, Raoult D, Fournier PE. Genetic differentiation of Chinese isolates of Rickettsia sibirica by partial ompA gene sequencing and multispacer typing. *J Clin Microbiol* 2006; **44**(7): 2465-7.

1336. Chen M, Fan MY, Bi DZ, Zhang JZ, Huang YP. Detection of Rickettsia sibirica in ticks and small mammals collected in three different regions of China. *Acta Virol* 1998; **42**(1): 61-4.
1337. Zou Y, Wang Q, Fu Z, et al. Detection of spotted fever group Rickettsia in Haemaphysalis longicornis from Hebei Province, China. *J Parasitol* 2011; **97**(5): 960-2.
1338. Teng Z, Shi Y, Peng Y, et al. Severe Case of Rickettsiosis Identified by Metagenomic Sequencing, China. *Emerg Infect Dis* 2021; **27**(5): 1530-2.
1339. Yu X, Jin Y, Fan M, Xu G, Liu Q, Raoult D. Genotypic and antigenic identification of two new strains of spotted fever group rickettsiae isolated from China. *J Clin Microbiol* 1993; **31**(1): 83-8.
1340. Liu QH, Chen GY, Jin Y, et al. Evidence for a high prevalence of spotted fever group rickettsial infections in diverse ecologic zones of Inner Mongolia. *Epidemiol Infect* 1995; **115**(1): 177-83.
1341. Chen M, Fan MY, Bi DZ. [A molecular epidemiologic investigation of north Asia fever in scenic spots of Beijing suburb]. *Zhonghua Liu Xing Bing Xue Za Zhi* 1997; **18**(4): 197-200.
1342. Chen M, Fan M, Bi D, Zhang J. [Cloning and sequence analysis of rOmpA gene fragment of spotted fever group Rickettsiae isolated in China]. *Wei Sheng Wu Xue Bao* 1998; **38**(4): 276-82.
1343. Zhang L, Jin J, Fu X, Raoult D, Fournier P-EJJoCM. Genetic differentiation of Chinese isolates of Rickettsia sibirica by partial ompA gene sequencing and multispacer typing. 2006; **44**(7): 2465-7.
1344. Kun T. Study on the carrying status of three pathogens in ticks and rats in Heilongjiang Forest Region: Central South University; 2012.
1345. Muzi J, Yunhua, Yufei C, Ruixing X, Bin L. Investigation on Rodents and Their Pathogens in Inner Mongolia Port Area from 2014 to 2015. *Chinese Journal of Vector Biology and Control* 2017; **28**(4): 3.
1346. Ya Hongxiang ZH, Zhou Jihua, Duan Hengxian, Zhao Xiaoze, Sun Guijuan, Zheng Weibin, Huang Wenli. Investigation on an outbreak of endemic typhus in Baoshan City, Yunnan Province. *Chinese Jourl of Epidemiology* 2011; **32**(1): 4.
1347. Yuan Qinghong YX. Investigation of Rickettsia typhi in small mammals in Luxi county，Yunn province，China. *Chinese Jourl of Vector Biology and Control* 2020; **31**(3): 6.
1348. Lu Tingting FY, Hou Yong, Yang Yu, Xu Baoliang, Ying Changqing, Cai Jing, Wang Jiancheng, Zhao Tingting. The infection status of Rickettsia typhi in rodents on Heixiazi Island at the Sino-Russian Border *Chinese Jourl of Vector Biology and Control* 2015; **26**(6): 5.
1349. Zhang SF, Du J, Mi XM, et al. Rickettsia typhi infection in severe fever with thrombocytopenia patients, China. *Emerg Microbes Infect* 2019; **8**(1): 579-84.
1350. Kuo CC, Wardrop N, Chang CT, Wang HC, Atkinson PM. Significance of major international seaports in the distribution of murine typhus in Taiwan. *PLoS Negl Trop Dis* 2017; **11**(3): e0005430.
1351. Yang WH, Dong T, Zhang HL, et al. Murine typhus in drug detoxification facility, Yunnan Province, China, 2010. *Emerg Infect Dis* 2012; **18**(8): 1388-90.
1352. Zhang L, Shan A, Mathew B, et al. Rickettsial Seroepidemiology among farm workers, Tianjin, People's Republic of China. *Emerg Infect Dis* 2008; **14**(6): 938-40.
1353. Chai CL, Lu QY, Sun JM, et al. [Sero-epidemiologic investigation on tick-borne diseases of humans and domestic animals in Zhejiang province]. *Zhonghua Liu Xing Bing Xue Za Zhi* 2010; **31**(10): 1144-7.
1354. Wu YM, Zhang ZQ, Wang HJ, Yang Q, Feng L, Wang JW. [Investigation on the epidemiology of Far-East tick-borne spotted fever in the Northeastern area of China]. *Zhonghua Liu Xing Bing Xue Za Zhi* 2008; **29**(12): 1173-5.
1355. Bassett DC, Ho AK, Tam JS, Lam LY, Cheng AF. The laboratory diagnosis of rickettsial diseases in Hong Kong. *J Trop Med Hyg* 1992; **95**(5): 327-30.
1356. Huang Hain DZ, He Jing, Wu Xiaoming, Jiang Guigui, Gao Yan, Zhao Qiumin, Cao Wuchun. Detection of Spotted Fever Group Rickettsia in Rodents in Some Provinces. *Chinese Jourl of Vector Biology and Control* 2006; **17**(2): 83-5.
1357. Zuo Shuangyan TK, Zheng Yuanchun, Huo Qiubao, Song Yudong, Zeng Xiaomin. Ddetection and sequence alysis of spotted fever group Rickettsiae in rodents from Heilongjiang forest region. *Jourl of Central South University (Medical Edition)* 2013; **38**(5): 443-7.
1358. Wang Zhuo YM, Yang Shenjiang, Qiu Guangbin, Zhai Rubo, Guo Zunyuan, Feng Li, Wu Yimin. Investigation of Spotted Fever Rickettsia in Rats in the Border Area between Chi and North Korea. *Chinese Jourl of Preventive Medicine* 2015; (3): 3.
1359. Huang Hain DZ, He Jing, Wu Xiaoming, Jiang Guigui, Gao Yan, Zhao Qiumin, Cao Wuchun. Detection of Spotted Fever Group Rickettsia in Rodents in Some Provinces. *Chinese Jourl of Vector Biology and Control* 2006.
1360. Xiuping F, Jingshan Z, Xiaojing S, Mingchun L, Menglei L, Lijuan Z. Investigation on Rickettsia Infection of Rodents in Xinyang District, Henan Province. *Chinese Journal of Epidemiology*

2007; **28**(6): 3.
1361. Jianping Z, Zhenguang C, Min C, Bidzeng. Detection of Spotted Fever Group Rickettsia in Rat Ticks by PCR/RFLP. *Chinese Journal of Health Laboratory Technology* 2005; **15**(4): 2.
1362. Wendong J, Cheng C, Weiming F, et al. Investigation on Spotted Fever Group Rickettsia at Heilongjiang Port. *Journal of Parasites and Medical Insects* 2015; (1): 7.
1363. Yingzi L. Isolation and identification of spotted fever group rickettsia Hainan strain. *Jiangxi Medical College* 2001.
1364. Xiaomei C, Xiaolong Z, Wei F, et al. Investigation on Pathogens of Rat Parasitic Tick Infection at Erlianhot Port. *Chinese Journal of Vector Biology and Control* 2014.
1365. Huijie L, Dongwei W, Yumei W, Jun Z, Tianshu S. Analysis on special monitoring of rodents at Heilongjiang border ports from 2019 to 2020. *China Inspection and Quarantine* 2021; **003**(010): 23-9.
1366. Zhenguang C, Min C, Jianping Z, Bidzeng. Detection of Spotted Fever Group Rickettsia in Rodents and Ticks in Ninghua Forest Area, Fujian Province. *Chinese Journal of Preventive Medicine* 2002; **036**(002): 106-8.
1367. Zhenguang C, Maoyin Z, Jinrong H, et al. Investigation on Spotted Fever Group Rickettsia Infection of Animals in Ninghua Forest Region, Fujian Province. *Chinese Journal of Vector Biology and Control* 1999; **010**(005): 367-70.
1368. First Isolation of Spotted Fever Group Rickettsia in Guangdong Province. *South China Journal of Preventive Medicine* 2002; (01): 18-20.
1369. Zhenguang C, Min C, Jianping Z, Bidzeng. Detection of Spotted Fever Group Rickettsia in Rodents and Ticks in Ninghua Forest Area, Fujian Province. *Chinese Journal of Preventive Medicine* 2002; **36**(2): 3.
1370. Hongxiang Y, Jinglin W, Youhong Z. Investigation on natural infection of rickettsia by rodents and vector insects in some areas of yunnan province. *Chinese Journal of Disease Control & Prevention* 2019; (2): 5.
1371. Hailin Z, Hong Y, Panhe Z, et al. Spotted fever group rickettsia DNA was detected in rats and ticks in Dali City, Yunnan Province. *Chinese Journal of Vector Biology and Control* 2004; **15**(6): 2.
1372. Hua D, Yanfei G, Xiaolan C, Yufeng G. Nucleic Acid Detection and Sequence Analysis of Spotted Fever Group Rickettsia Carried by Ticks at Manzhouli Port. *Chinese Journal of Frontier Health and Quarantine* 2014; (5): 3.
1373. Luoyuan X. Discovery, Isolation, Identification and Potential Pathogenicity of New Spotted Fever Group Rickettsia in Southern China. 2020.
1374. Jinrong H, Yan G. Serological Investigation of Spotted Fever Group Rickettsia in Dapu County, Guangdong Province. *Chinese Journal of Vector Biology and Control* 1998; **9**(6): 3.
1375. Zhenguang C. Investigation on tick carrying rodents and infected spotted fever and Lyme disease in Ninghua County. *Strait Journal of Preventive Medicine* 1995; **1**(1): 2.
1376. Wang Zhuo WR, Zhou Dezhuang, Yang Yijun, Yu Miao, Wang Feng, Feng Li, Wu Yimin. Detection and compound infection of tick borne Rickettsia in rodents in Northeast Forest Area. *Chinese Jourl of Vector Biology and Control* 2015; **26**(005): 467-70.
1377. Yimin W, Guoping L, Anming W, et al. Investigation on natural foci of tick borne spotted fever in some areas. *Chinese Journal of Public Health* 2003; **19**(9): 2.
1378. Zhenguang C, Rongman X. Investigation on the Vector and Host of Spotted Fever Ticks in Ninghua, Fujian. *Chinese Journal of Public Health* 1999; **15**(8): 2.
1379. Niu, Lina, Hu, et al. Isolation and characterization of Streptococcus respiraculi sp nov from Marmota himalayana (Himalayan marmot) respiratory tract. 2018.
1380. Yu X, Jin Y, Fan M, Xu G, Liu Q, Raoult DJJoCM. Genotypic and antigenic identification of two new strains of spotted fever group rickettsiae isolated from China. 1993; **31**(1): 83-8.
1381. Liu Q-H, Chen G-Y, Jin Y, et al. Evidence for a high prevalence of spotted fever group rickettsial infections in diverse ecologic zones of Inner Mongolia. 1995; **115**(1): 177-83.
1382. Cai Zenglin TZ, Shen Baojun, Quan Lihua, Gu Xiufu, Liu Guoping, Lou Dan, Liu Guodong, Wu Yimin, Ding Guilin. Investigation Report on the tural Focus of Beiya Tick borne Spotted Fever in Luobei County, Heilongjiang Province. *Public Health in China* 1985; **4**(2).
1383. Chen Shuangyan GY, Zhang Haipeng, Tan Hongli, Wang Peng. Investigation of Yersinia enterocolitica in wild plague foci in Jianchuan,Yunnan. *Disease surveillance* 2016; (2): 4.
1384. Yongjin C, Yinbao L, Guizhu Y, Zuxiang X, Jiufei Y. Distribution of Yersinia enterocolitica in rodents. *Chinese Journal of Zoonoses* 1987; (01): 37-8.
1385. Zeng Min YH, Liang Qiuguang, Pan Zhu, Yang Liu. Distribution of Yersinia enterocolitica in resting period of pestis in Guangdong Province. *Chinese Tropical Medicine* 2006; **6**(3): 2.
1386. Zhang Cheng LD, Luo Jun, Li Kaihua, Sha Jundong, Li Haizhi. Investigation and Control of

pestis Animal Disease Epidemic in Zhenkang County. *Chinese Jourl of Endemic Disease Control* 1997; (2): 108-9.
1387. Qin S, Liang J, Tang D, et al. Serological investigation of plague and brucellosis infection in Marmota himalayana plague foci in the Altun Mountains on the Qinghai-Tibet Plateau. *Front Public Health* 2022; **10**: 990218.
1388. Tang D, Duan R, Chen Y, et al. Plague Outbreak of a Marmota himalayana Family Emerging from Hibernation. *Vector Borne Zoonotic Dis* 2022; **22**(8): 410-8.
1389. Mou W, Li B, Wang X, et al. Flea index predicts plague epizootics among great gerbils (Rhombomys opimus) in the Junggar Basin China plague focus. *Parasit Vectors* 2022; **15**(1): 214.
1390. Xi J, Duan R, He Z, et al. First Case Report of Human Plague Caused by Excavation, Skinning, and Eating of a Hibernating Marmot (Marmota himalayana). *Front Public Health* 2022; **10**: 910872.
1391. Chen Y, Song K, Chen X, et al. Attenuation of Yersinia pestis fyuA Mutants Caused by Iron Uptake Inhibition and Decreased Survivability in Macrophages. *Front Cell Infect Microbiol* 2022; **12**: 874773.
1392. Liu BX, Duan R, Wang HH, et al. [Analysis on prevalence and epidemic risk of animal plague in different ecological plague foci in Inner Mongolia Autonomous Region]. *Zhonghua Yu Fang Yi Xue Za Zhi* 2022; **56**(1): 9-14.
1393. Cao S, Liu X, Huang Y, et al. Proteogenomic discovery of sORF-encoded peptides associated with bacterial virulence in Yersinia pestis. *Commun Biol* 2021; **4**(1): 1248.
1394. Mahmoudi A, Kryštufek B, Sludsky A, et al. Plague reservoir species throughout the world. *Integr Zool* 2021; **16**(6): 820-33.
1395. He Z, Wei B, Zhang Y, et al. Distribution and Characteristics of Human Plague Cases and Yersinia pestis Isolates from 4 Marmota Plague Foci, China, 1950-2019. *Emerg Infect Dis* 2021; **27**(10): 2544-53.
1396. Li J, Wang Y, Liu F, et al. Genetic source tracking of human plague cases in Inner Mongolia-Beijing, 2019. *PLoS Negl Trop Dis* 2021; **15**(8): e0009558.
1397. Ji N, Chen X, Liu G, et al. Theileria, Hepatozoon and Taenia infection in great gerbils (Rhombomys opimus) in northwestern China. *Int J Parasitol Parasites Wildl* 2021; **15**: 79-86.
1398. Liang J, Qin S, Duan R, et al. A Lytic Yersina pestis Bacteriophage Obtained From the Bone Marrow of Marmota himalayana in a Plague-Focus Area in China. *Front Cell Infect Microbiol* 2021; **11**: 700322.
1399. Shi L, Qin J, Zheng H, et al. New Genotype of Yersinia pestis Found in Live Rodents in Yunnan Province, China. *Front Microbiol* 2021; **12**: 628335.
1400. Zhou H, Guo S. Two cases of imported pneumonic plague in Beijing, China. *Medicine (Baltimore)* 2020; **99**(44): e22932.
1401. Kong JJ, Wang P, Liang Y, Su LQ, Shi LY. [Epidemiological analysis of Plague in Dehong Dai and Jingpo Autonomous Prefecture of Yunnan province, 1950-2019]. *Zhonghua Liu Xing Bing Xue Za Zhi* 2020; **41**(9): 1504-8.
1402. Park YH, Remmers EF, Lee W, et al. Ancient familial Mediterranean fever mutations in human pyrin and resistance to Yersinia pestis. *Nat Immunol* 2020; **21**(8): 857-67.
1403. Liu B, Bai L, Fu Y, et al. Genetic and molecular features for hepadnavirus and plague infections in the Himalayan marmot. *Genome* 2020; **63**(6): 307-17.
1404. Zhang Y, Ying X, He Y, et al. Invasiveness of the Yersinia pestis ail protein contributes to host dissemination in pneumonic and oral plague. *Microb Pathog* 2020; **141**: 103993.
1405. Chou PC, Lin FP, Hsu HL, Chang CJ, Lu CH, Chen JK. Electrorheological Sensor Encapsulating Microsphere Media for Plague Diagnosis with Rapid Visualization. *ACS Sens* 2020; **5**(3): 665-73.
1406. Cui Y, Schmid BV, Cao H, et al. Evolutionary selection of biofilm-mediated extended phenotypes in Yersinia pestis in response to a fluctuating environment. *Nat Commun* 2020; **11**(1): 281.
1407. Sun Z, Zhang Z, Liu Q, et al. Identifying the spatiotemporal clusters of plague occurrences in China during the Third Pandemic. *Integr Zool* 2020; **15**(1): 69-78.
1408. Wang Y, Zhou L, Fan M, et al. Isolated Cases of Plague - Inner Mongolia-Beijing, 2019. *China CDC Wkly* 2019; **1**(1): 13-6.
1409. Liu S, Feng J, Pu J, et al. Genomic and molecular characterisation of Escherichia marmotae from wild rodents in Qinghai-Tibet plateau as a potential pathogen. *Sci Rep* 2019; **9**(1): 10619.
1410. Xu L, Stige LC, Leirs H, et al. Historical and genomic data reveal the influencing factors on global transmission velocity of plague during the Third Pandemic. *Proc Natl Acad Sci U S A* 2019; **116**(24): 11833-8.
1411. Zhang Q, Xin Y, Zhao H, et al. Human Macrophages Clear the Biovar Microtus Strain of Yersinia pestis More Efficiently Than Murine Macrophages. *Front Cell Infect Microbiol* 2019; **9**: 111.

1412. Dai R, Qi M, Xiong H, et al. Serological Epidemiological Investigation of Tibetan Sheep (Ovis aries) Plague in Qinghai, China. *Vector Borne Zoonotic Dis* 2019; **19**(1): 3-7.
1413. Fang H, Liu L, Zhang Y, et al. BfvR, an AraC-Family Regulator, Controls Biofilm Formation and pH6 Antigen Production in Opposite Ways in Yersinia pestis Biovar Microtus. *Front Cell Infect Microbiol* 2018; **8**: 347.
1414. Tian L. Relationship between environmental factors and the spatial distribution of Spermophilus dauricus during 2000-2015 in China. *Int J Biometeorol* 2018; **62**(10): 1781-9.
1415. Shi L, Yang G, Zhang Z, et al. Reemergence of human plague in Yunnan, China in 2016. *PLoS One* 2018; **13**(6): e0198067.
1416. Zhao SS, Pulati Y, Yin XP, et al. Wildlife Plague Surveillance Near the China-Kazakhstan Border: 2012-2015. *Transbound Emerg Dis* 2017; **64**(6): e48-e51.
1417. Zhang YJ, Wang C, Luo T, Guo R, Meng WW. [Epidemics and risk factors of plague in Junggar Basin, Xinjiang Uygur Autonomous Region, 2007-2016]. *Zhonghua Liu Xing Bing Xue Za Zhi* 2017; **38**(10): 1394-8.
1418. Meng WW, Wang XH, Luo T, et al. [Dynamics of F1 antibody responses to Yersinia pestis infection in Rhombomys opimus]. *Zhonghua Yu Fang Yi Xue Za Zhi* 2017; **51**(4): 353-7.
1419. Wang X, Wei X, Song Z, et al. Mechanism study on a plague outbreak driven by the construction of a large reservoir in southwest china (surveillance from 2000-2015). *PLoS Negl Trop Dis* 2017; **11**(3): e0005425.
1420. Li B, Azhati R, Meng WW, et al. [Experimental observation on the histopathological and ultrastructural pathology of Great Gerbils (Rhombomys opimus) in the Junggar Basin by subcutaneous injecting of Yersinia pestis]. *Zhonghua Yu Fang Yi Xue Za Zhi* 2017; **51**(2): 172-5.
1421. Li CX, Wang P, Shen XN, et al. [Isolation and biological characteristics on Yersinia pestis phage YP060]. *Zhonghua Liu Xing Bing Xue Za Zhi* 2016; **37**(6): 868-71.
1422. Zhang AP, Wei RJ, Xiong HM, Wang ZY. [Advance to the research of the climate factor effect on the distribution of plague]. *Zhonghua Yu Fang Yi Xue Za Zhi* 2016; **50**(5): 459-62.
1423. Shi G, Ju C, Zhang R, et al. [Risk assessments and control strategies of plague in five key surveillance counties, Zhejiang province]. *Zhonghua Yu Fang Yi Xue Za Zhi* 2015; **49**(10): 896-900.
1424. Ge P, Xi J, Ding J, et al. Primary case of human pneumonic plague occurring in a Himalayan marmot natural focus area Gansu Province, China. *Int J Infect Dis* 2015; **33**: 67-70.
1425. Wei B, Xiong H, Yang X, et al. [The epidemiology and etiology research of Tibetan sheep plague in Qinghai plateau]. *Zhonghua Liu Xing Bing Xue Za Zhi* 2015; **36**(3): 271-4.
1426. Liang Y, Xie F, Tang X, et al. Chromosomal rearrangement features of Yersinia pestis strains from natural plague foci in China. *Am J Trop Med Hyg* 2014; **91**(4): 722-8.
1427. Shi L, Ye R, Dong S, et al. [Genotyping and its epidemiological significance on Yunnan Yersinia pestis under Fse I enzyme digestion method]. *Zhonghua Liu Xing Bing Xue Za Zhi* 2014; **35**(2): 182-5.
1428. Zhou X, Zhang B, Cong X, et al. [Application of Best Subsets Regression on the risk classification for Spermophilus Dauricus Focus]. *Zhonghua Liu Xing Bing Xue Za Zhi* 2014; **35**(2): 170-3.
1429. Dai RX, Wei BQ, Li CX, et al. [The pathogenic ecology research on plague in Qinghai plateau]. *Zhonghua Yu Fang Yi Xue Za Zhi* 2013; **47**(12): 1083-8.
1430. Fang XY, Yang RF, Xu L, et al. [Ecological-geographic landscapes of natural plague foci in China VII. Typing of natural plague foci]. *Zhonghua Liu Xing Bing Xue Za Zhi* 2012; **33**(11): 1144-50.
1431. Qi Z, Zhao H, Zhang Q, et al. Acquisition of maternal antibodies both from the placenta and by lactation protects mouse offspring from Yersinia pestis challenge. *Clin Vaccine Immunol* 2012; **19**(11): 1746-50.
1432. Sun S, Yang X, Yuan Y, et al. Draft genome sequence of Yersinia pestis strain 2501, an isolate from the great gerbil plague focus in Xinjiang, China. *J Bacteriol* 2012; **194**(19): 5447-8.
1433. Bevins SN, Baroch JA, Nolte DL, Zhang M, He H. Yersinia pestis: examining wildlife plague surveillance in China and the USA. *Integr Zool* 2012; **7**(1): 99-109.
1434. Zhang Y, Dai X, Wang X, et al. Dynamics of Yersinia pestis and its antibody response in great gerbils (Rhombomys opimus) by subcutaneous infection. *PLoS One* 2012; **7**(10): e46820.
1435. Du CH, Wang P, Zhang JZ, Tang X, Song ZZ. [Preparation and identification of monoclonal antibodies against Pla protein of Yersinia pestis]. *Xi Bao Yu Fen Zi Mian Yi Xue Za Zhi* 2011; **27**(9): 985-8.
1436. Wang LM, Song XY, Zhu XP, et al. [Fleas notified on Microtus fuscus foci in Sichuan province]. *Zhonghua Liu Xing Bing Xue Za Zhi* 2011; **32**(7): 685-8.
1437. Tian G, Qiu Y, Qi Z, et al. Histopathological observation of immunized rhesus macaques with plague vaccines after subcutaneous infection of Yersinia pestis. *PLoS One* 2011; **6**(4): e19260.

1438. Ge PF, Zhang H, Guo ZB, et al. [Study on genotypes and geographical distribution of 58 strains of Yersinia pestis in Gansu]. *Zhonghua Liu Xing Bing Xue Za Zhi* 2010; **31**(7): 840.
1439. Eppinger M, Guo Z, Sebastian Y, et al. Draft genome sequences of Yersinia pestis isolates from natural foci of endemic plague in China. *J Bacteriol* 2009; **191**(24): 7628-9.
1440. Wang LM, Song XY, Zhu XP, et al. [Monitoring the Microtus fuscus plague epidemic in Sichuan province during 2000-2008]. *Zhonghua Liu Xing Bing Xue Za Zhi* 2009; **30**(11): 1175-8.
1441. Wang LM, Luo ZD, Yue Q, et al. [Study on the natural foci of Marmota himalayana plague in Sichuan province]. *Zhonghua Liu Xing Bing Xue Za Zhi* 2009; **30**(6): 601-5.
1442. Liu H, Wang H, Qiu J, et al. Transcriptional profiling of a mice plague model: insights into interaction between Yersinia pestis and its host. *J Basic Microbiol* 2009; **49**(1): 92-9.
1443. Qi ZZ, Li C, Wang L, et al. [Study on the pathogen of plague in Sanjiangyuan area in Qinghai province]. *Zhonghua Liu Xing Bing Xue Za Zhi* 2009; **30**(1): 55-7.
1444. Nikitin A, Maramovich AS, Bazanova LP, et al. [Epizootological characteristics of the natural foci of plague in China: a review of literature]. *Med Parazitol (Mosk)* 2009; (1): 51-8.
1445. Li Y, Gao H, Qin L, et al. Identification and characterization of PhoP regulon members in Yersinia pestis biovar Microtus. *BMC Genomics* 2008; **9**: 143.
1446. Wu AG, Li TY, Feng JM, Dong XQ. [Study on the epidemiological significance related to community-structural difference of the rat plague host and vectors in Western Yunnan, China]. *Zhonghua Liu Xing Bing Xue Za Zhi* 2008; **29**(4): 346-50.
1447. Zhang YJ, Dai X, Abulimiti, et al. [Study on the situation of plague in Junggar Basin of China]. *Zhonghua Liu Xing Bing Xue Za Zhi* 2008; **29**(2): 136-44.
1448. Maramovich AS, Kosilko SA, Innokent'eva TI, et al. [Plague in China. Threat of transmission to regions of Siberia and Far East]. *Zh Mikrobiol Epidemiol Immunobiol* 2008; (1): 95-9.
1449. Zhou D, Han Y, Yang R. Molecular and physiological insights into plague transmission, virulence and etiology. *Microbes Infect* 2006; **8**(1): 273-84.
1450. Li M, Song Y, Li B, et al. Asymptomatic Yersinia pestis infection, China. *Emerg Infect Dis* 2005; **11**(9): 1494-6.
1451. Li W, Hai R, Yu DZ, Zhang ZK, Cai H. [Establishment and application of real-time fluorescence polymerase chain reaction based on the TaqMan probes for detection of Yersinia pestis]. *Zhonghua Liu Xing Bing Xue Za Zhi* 2005; **26**(8): 613-6.
1452. Song Y, Tong Z, Wang J, et al. Complete genome sequence of Yersinia pestis strain 91001, an isolate avirulent to humans. *DNA Res* 2004; **11**(3): 179-97.
1453. Hai R, Yu DZ, Wei JC, et al. [Molecular biological characteristics and genetic significance of Yersinia pestis in China]. *Zhonghua Liu Xing Bing Xue Za Zhi* 2004; **25**(6): 509-13.
1454. Zietz BP, Dunkelberg H. The history of the plague and the research on the causative agent Yersinia pestis. *Int J Hyg Environ Health* 2004; **207**(2): 165-78.
1455. Fukushima H, Hao Q, Wu K, et al. Yersinia enterocolitica O9 as a possible barrier against Y. pestis in natural plague foci in Ningxia, China. *Curr Microbiol* 2001; **42**(1): 1-7.
1456. Dong XQ, Lindler LE, Chu MC. Complete DNA sequence and analysis of an emerging cryptic plasmid isolated from Yersinia pestis. *Plasmid* 2000; **43**(2): 144-8.
1457. Li Cunxiang DR, Zhao Haihong, He Jian, Ren Lingling, Zhang Shanhu. Report on isolation of 10 strains of yersinia pestis carrying wild － type bacteriophage. *Medical animal control* 2013; (9): 2.
1458. Li Yong JZ. Alysis of plague surveillance in Yuanzhou District, Guyuan, Ningxia, 1977-2008. *Endemic Diseases Bulletin* 2009.
1459. Diseases IToADA. Investigation report on mountain plague tural foci of Xinjiang in 1987. *Endemic Diseases Bulletin* 1988; **Investigation Team of Alpine Desert Animal Diseases**(3).
1460. Xu Zhujia ZH. Epidemiological surveillance report of plague in Raoping County in 1990. *Hygiene and epidemic prevention in Guangdong* 1993; (2): 26-8.
1461. Jin Yong YX, He Jian, Dai Ruixia, Zhao Haihong, Xin Youquan, Xiong Haoming, Wu Hailian, Li Jun, Wei Baiqing. Epidemiological Alysis of pestis in Delingha City from 1995 to 2014. *Medical animal control* 2015.
1462. Junsheng A. Surveillance and Alysis of the Epidemic Situation of Citellus Alxa pestis Foci from 1996 to 2015. *Disease prevention and control notification* 2016; (4): 3.
1463. Albumitti WX, Peng Yi, Chen Jianxing, Dai Xiang, Yu Guolin. Surveillance Report on pestis in Guertu Area, Wusu County, Xinjiang in 1996. *Endemic Diseases Bulletin* 1997; (3 期): 67-8.
1464. Yang Zengyuan LJ, Li Honggen, Luo Qisong, Liang Wei. A case report of human glandular pestis. *Chinese Jourl of Vector Biology and Control* 2001; (002): 012.
1465. Wang Chengxin LG. Epidemic situation and prevention of major human rodent diseases in Chi from 2000 to 2005. *Science and Technology Guide* 2007; **25**(1): 6.

1466. Huang Jianzhong DZ. Prelimiry Study on tural Foci of pestis in Shiqu County, Sichuan Province from 2000 to 2013. *Jourl of Preventive Medicine Information* 2015; (1): 8.
1467. Qi B. Epidemiological analysis of pestis surveillance in the focus of microtus in Qinghai from 2001 to 2015: Jilin University.
1468. River PY, Jinjiang Y, Jianyong S, et al. Surveillance and Analysis of pestis in Wenquan County, Xinjiang, 2002-2004. *Bulletin of Disease Control and Prevention* 2006; **21**(6): 27-8.
1469. Wang Li BJ, Yue Yongjie, Ruan Chunlai. Alysis of laboratory surveillance results of pestis in shaanxi province in 2003. *Endemic Diseases Bulletin* 2005.
1470. Aziz Muhammad CH, Abu Likmu, Wang Xinhui, Azati, Muhayati, Bolati, and Juleti. Investigation Report on pestis Foci in Wenquan County, Xinjiang Uygur Autonomous Region in 2004. *Endemic Diseases Bulletin* 2007; **22**(004): 43-5.
1471. Jiang Yan WY, Huo Minping. Epidemic dymic alysis of animal pestis in Xilinhot from 2005 to 2013. *Chinese Jourl of Endemic Disease Control* 2015; (3): 1.
1472. Gao Qin SX. Surveillance and Alysis of pestis among Rats in Yanchi County, Ningxia in 2005. *Endemic Diseases Bulletin* 2007; **22**(2): 35-6.
1473. Sun Shi ALA, Bi Yongjiang. Prevalence of pestis of Citellus undulatus in Xinjiang Uygur Autonomous Region from 2006 to 2010 and its control countermeasures. *Disease prevention and control notification* 2013; (5): 2.
1474. Wang Limao MS, Yang Changhong, Zhu Xiaoping, Li Fan, Wu Chaoxue, Liu Yi, Qi Teng, Yue Qi, Wang Hong, Luo Zhidanba, Duan Yongjun, Ze Rensangzhu, Huang Jianzhong. Epidemiological surveillance of marmot plague in Sichuan Province in 2007-2009. *Jourl of Preventive Medicine Information* 2009; **25**(11): 6.
1475. Qi Teng WL, Ma Li, Luo Longze, Luo Zhidanba, Duan Yongjun, Yue Qi, Wang Hong, Liu Yi, Li Fan, Wu Chaoxue, Danbazeli, He Qianjun, Duan Fenggang, Liu Qisheng, Tang Yong, Ze Rensangzhu, Zhaxi Pengcuo. Alysis of detection results of pestis related samples in Dege County from 2007 to 2010. *Jourl of Preventive Medicine Information* 2011; **27**(11): 4.
1476. Polati SC, Liang Jishan, Guan Rong, Zong Jun. Surveillance and Alysis of pestis in Bortala Mongol Autonomous Prefecture of Xinjiang in 2007. *Endemic Diseases Bulletin* 2010; (4): 2.
1477. Duan Yongjun LW, Wang Limao, Liu Lunguang, Li Fan, He Qianjun, Duan Fenggang, Yang Jun, Dao Ji, Qi Teng. Alysis of the investigation results of human pestis in Litang County in 2012. *Modern Preventive Medicine* 2015; **42**(2): 3.
1478. Li Jing WJ, Miao Zhifeng, Lei Guilian. Yinchuan first isolated a strain of Yersinia pestis from the three toed jumping rat. *Medical animal control* 2005; **21**(011): 792-.
1479. Selihan HJ, Ainiwar, Chen Wenliang, Wang Xinhua, Guo Qingshan. Urumqi first isolated a strain of Yersinia pestis from the foot frog. *Medical animal control* 2010; (11): 1.
1480. Jirui L. The Yersinia pestis animal and rodent species were isolated from the plague foci of Jianchuan, Yunnan. *Endemic Diseases Bulletin* 1999; **02**: 7-.
1481. Gao Zihou DX, Song Zhizhong, Liang Yun, Zhao Wenhong, Yang Zhiming. Study on Host Animals of tural Infection with Plague in Yunn Province. *Endemic Diseases Bulletin* 2008; **23**(3): 15-8.
1482. Bo W. Isolation, Biological Characteristics and Genome Study of Plague Phage from pestis Focal Area of Wild Rat in Heqing.
1483. Yuan Yue ZY, Zhang Haipeng, Su Chao, Yang Lihua, Yang Fengyi, Wu Hesong, Wang Peng. Isolation of Yersinia pestis phage from Rattus norvegicus in Yunn province and its epidemiological significance. *Disease surveillance* 2020; **35**(3): 4.
1484. Zhang Haipeng JX, Gao Jinlian, Yang Fengyi, Wang Peng, Zhong Youhong. Isolation of Yersinia pestis phage from Rattus nitidus in Yunn Province and its epidemiological significance. *Endemic disease control in Chi* 2021; **36**(3): 3.
1485. Guo Mu ZY, Hong Mei, Tian Chuqi, Wu Hesong, Yang Guirong, Liu Zhengxiang, Pu Ennian, Chen Zhijun, Xiao Shuangxing, Wang Peng. Isolation and identification of plague phage from plague foci in Ancient District, Lijiang, Yunnan. *Chinese Tropical Medicine* 2021; **21**(5): 4.
1486. Liang Ying WL, Luo Xiaozhi, Zhao Hongqun, Cai Hong, Li Wei. Genetic characteristics of the first Yersinia pestis strain isolated from Batang county of Sichuan, Chi. *Chinese Jourl of Vector Biology and Control* 2015; **26**(3): 4.
1487. Wang Limao SX, Xue Mei, Li Fuzhong, Jiang Hezhu, Wang Xiao. Detection of Yersinia pestis in Sichuan province for the first time. *Chinese Jourl of Endemic Disease Control* 1999; **14**(5): 2.
1488. Yin Xiaoping YR, Li Dong. Alysis of Plague Surveillance in Mayle Mountain and Its Around Area in Xinjiang. *Endemic Diseases Bulletin* 2010; (1): 37-9.
1489. Fulati Yimingjiang YM, Xiamuxidin Huahong, Liu Mingtao. Monitoring and alysis of pestis prevention and control in Wenquan County, Xinjiang in 2015. *Disease prevention and control notification* 2017; **32**(2): 3.

1490. Li Yongyong LH, Xing Tianyao, Lv Chengliang, Zhang Xiwei, Yu Wei. Alysis of pestis Surveillance Results in Jinghong City, Yunnan Province, 2003-2012. *Chinese Jourl of Frontier Health and Quarantine* 2014; (10): 4.
1491. Prati Yimingjiang SJ, Li Donghui, An Wenyan, Chen Chunxia, Muheyati. Pestis pestis was first isolated from Kuktawu area of Alataw Mountain. *Jourl of Disease Control* 2004.
1492. Yang Jun DY, Luo Zhidanba, Duan Fenggang, Wang Limao, Qi Teng, Tang Yong, Liu Jian, Chen Jiankang, Wang Peng, Nima, Zhaxi Duoji. Investigation of pestis tural Foci in Batang County. *Jourl of Preventive Medicine Information* 2014; **30**(1): 3.
1493. Tang Fang LS, Wang Aiguo, Gao Dongqi, Wu Xiaoming, Zuo Shuqing, Wang Zheng, Cao Wuchun. Epidemiological investigation of hantaviruse carried by hosts in Beijing. *Public Health in China* 2005; **16**(9): 3.
1494. Yugui L. Epidemic Alysis of an Animal Disease Caused by pestis in Chahar Hill. *Chinese Jourl of Vector Biology and Control* 2000; **11**(1): 3.
1495. Zeng Biaocheng YH, Liang Zhongze. Isolation of a strain of pseudotuberculosis from yellow haired mice. *Chin J Zoonoses* 1990; (06): 46-.
1496. Zhang Chunhua LJ, Pu Qingjiang. Current situation and control strategy of plague focus of Citellus dauricus. *Chinese Jourl of Endemic Disease Control* 2004; **19**(6): 345-.
1497. Ji Jiajun SZ. A Study on the Etiology and Serological Minimum Sampling Quantity of Wild Rats in the pestis Foci of Apodemus Qi and Apodemus miltiorrhiza in Zonggu, Western Yunn. *Medical animal control* 2008; **24**(3): 3.
1498. Ying B. A new understanding of the host of pestis in yunn rattus. *Chinese Jourl of Endemic Disease Control* 2000; **15**(004): 28-9.
1499. Ding Zhihui QM, Ren Jianzhong. Surveillance and Alysis of Animal pestis in Erlianhot City from 2005 to 2010. *Disease monitoring and control* 2011; **5**(7): 426-8.
1500. Shen Xiahua ZR, Deng Kaize. Epidemic Situation of pestis in Gansu Province. *Chinese Jourl of Endemic Disease Control* 1996; **11**(5): 3.
1501. Zhang Anning ZJ. A Study on the Infection of pestis pestis in Non host Animals in pestis tural Focus of Marmota himalaya in Zhangye City, Gansu Province. *Health vocatiol education* 2003; **18**(2): 2.
1502. Wu Deqiang WD. Research Status of pestis Epidemic Area in Gansu Province. *Chinese Jourl of Endemic Disease Control* 1999; **14**(3): 3.
1503. Zhang Anning WH, Qin Wanlong. Study on the Epidemic of Animal pestis in Zhangye Area of Gansu Province from 1982 to 2000. *Endemic Diseases Bulletin* 2002; **17**(3): 3.
1504. Zhao Heguang WZ, Yang Guirong, Li Shuangchang, Wu Di, and Bu Liqun. Investigation Report on pestis Epidemic among Rats at the Border of Geng Ma. *Chinese Jourl of Frontier Health and Quarantine* 1998; **21**(1): 2.
1505. Xiong Mengtao ZW, Zhang Cheng, Zhao Heguang, Jiang Zhifeng, Huang Wenyu, Yang Qiongying. Epidemiological Survey of pestis in Mengding Town, Gengma County. *Endemic Diseases Bulletin* 1997; **012**(001): 66-7.
1506. Zhang Guining LX, Wu Dingchang, Shu Guobiao, Yuan Huafang, Gong Fan. Pestis Foci in Qianxin Prefecture, Guizhou Province. *Chinese Jourl of Endemic Disease Control* 2006; **21**(4): 3.
1507. Chen Guichun LT, Wang Zhaoxiao, Yao Guanghai, Liu Zhaobing, Cai Xinghe, Huang Hongwu, Hu Jing, Wang Dingming, Gong Xiaojun, Dong Li, Yu Chun. Investigation of pestis foci along the reservoir area of Tianshengqiao Hydropower Station in Guizhou Province. *Chinese Jourl of Endemic Diseases* 2003; **22**(005): 414-6.
1508. Zhang Xuedong ZC, Shi Xianming, Li Yugui, Chen Yongjiang, Li Zhenhai, Wang Guiqin, Xiang Youqing, Zhang Jian, Wu Xiaoming, Cui Xiuping, Liu Guanchun. Study on the Epidemic Law of Animal pestis in Hebei Province. *Pestis Prevention and Control Institute of Hebei Province* 2008; **23**(001): 51-2.
1509. Li Zhenhai ZX, Li Yugui. Epidemiological Investigation and Alysis of Animal pestis in Kangbao County, Hebei Province. *Chinese Jourl of Endemic Disease Control* 2004; (5): 296-7.
1510. Liu Manfu ZX, Wang Zhiyu, Liu Hezhi, Shi Xianming. Investigation on parasitic fleas of striped hamster in pestis tural foci in hebei province. *Medical animal control* 2003; **19**(11): 2.
1511. Li Yuanyuan WG, Wang Gang, Liu Fulong. Alysis of pestis surveillance results in Hohhot from 2004 to 2014. *Medical animal control* 2016; (5): 3.
1512. Bartel ZY, Han Bing, Zhao Gang. Monitoring and alysis of Mongolian marmot pestis in Hulunbeier Plateau from 2001 to 2010. *Chinese Jourl of Endemic Disease Control* 2014; (1): 3.
1513. Yang Shugong H, Galken, Aishan, Ahti, Ye Shengrong. Monitoring Report on pestis in 1999 on the North Slope of Tiange'er Mountain in Hutubi County. *Endemic Diseases Bulletin* 2000; **015**(004): 59-60.
1514. Xie Fengzhang YZ. Mixed infection of pestis pestis and erysipelas like bacteria in Marmota

marmota. *Chinese Jourl of Endemic Diseases* 1983; **000**(004): 228.
1515. Quanguo Seal CG. Surveillance and Alysis of pestis of Citellus Alxa in Huining County from 1997 to 2008. *Chinese Jourl of Endemic Disease Control* 2009; **24**(5): 1.
1516. Jiang Lin lin LZ, Zhou Fangxiao, Zhang Gui, Zhang Yanbing, Zhang Fang, Qiao Xiao, Yang Demin, Zhang Tiejun, Liao Kai, Liang Baocheng, Fan Zhiming, Zhang Minglin, Mi Jinxue, Li Jianwu, Yi Wei, Li Yonghai, Pan Xin, Tong Chengwu, Li Yueming, Guan Shujun, Zhang Xiaohui, Sun Lei, Zhang Xibi. Study on the Present Situation of pestis tural Foci in Jilin Province. *Scientific and technological achievements* 2007; **22**(3): 161-6.
1517. Li Huayu ZJ, Chen Hongwei, Happy, Qu Xianfeng, Zhou Bo. Tural focus of grassland pestis in northwest Jilin. *Chinese Jourl of Endemic Disease Control* 1996; **11**(6): 4.
1518. Fan Mengguang HX, Wang Lanfang, Zhang Dayu. Alysis on epidemic characteristics of pestis in Citellus dauricus in recent years. *Chinese Jourl of Vector Biology and Control* 2003; **014**(001): 50-2.
1519. Huang Zhiguang FX, Hu Jun, Ye Gang, Lei Gang, Jiang Wei, Xu Bing, Li Wei. Monitoring Report on pestis in Karamay from 2007 to 2009. *Chinese Jourl of Preventive Medicine* 2011.
1520. Guo Xiumei WM, Feng Xianlei, Huang Jianghe, Wang Qiguo. Investigation on rodents and fleas in pestis tural foci in Karamay in autumn. *Medical animal control* 2015; (10): 4.
1521. Tan Hongli GY, Yang Wenyan, Chen Fumei, Zhang Fuxin, Zhang Zhengfei, Wang Peng. An outbreak of pestis among rats was confirmed for the first time in Gucheng District of Lijiang City. *Chinese Jourl of Zoonoses* 2015; **31**(11): 3.
1522. Zhang Fuxin GY, Zhang Hongtao, Zhang Zhengfei, Liang Yun, He Yingtian, He Jiangong, Yang Tielong, Wang Guoliang, Lai Wenfeng, Lu Liang, Chen Zhijun, Lv Wei, Yang Wenyan. Discovery of pestis Foci in Lijiang City and Alysis of Monitoring Results. *Foreign Medicine (Medical Geography)* 2009; **30**(3): 3.
1523. Li Guojun SG, Li Baorong. Alysis of monitoring results of pestis of Citellus dauricus in Inner Mongolia in 2012. *Medical animal control* 2014; (3): 2.
1524. Shi Qiang FR, Li Jingyuan, Li Jianhua, Chen Zhijun, Wang Jianguo, Qin Fengcheng, Shang Zhikuan, Xu Haiquan, Meng Zhaoming, Zhao Fengqi, Si Qin, Niu Xiang, Xu Baoxiang. Tural focus of pestis of Citellus dauricus in northern Arhorqin Banner, Inner Mongolia. *Endemic Diseases Bulletin* 1990; **5**(2): 71-3.
1525. Han Bing ZG, Zhang Zhongbing, Li Jianyun, Zhang Dayu. Alysis on the epidemic situation of Citellus dauricus pestis focus in Inner Mongolia from 2001 to 2013. *Chinese Jourl of Vector Biology and Control* 2016; **27**(2): 4.
1526. Jin Muzi YH, Chen Yufei, Xu Ruixing, Li Bin. Investigation on Rodents and Their Pathogens in Inner Mongolia Port Area from 2014 to 2015. *Chinese Jourl of Vector Biology and Control* 2017; **28**(4): 3.
1527. Fan Mengguang LJ, Wei Ruiping, Zhao Gang, Mi Jingchuan. Rodent survey of Mongolian gerbil epidemic focus from 2000 to 2012. *Chinese Jourl of Endemic Disease Control* 2013; (4): 3.
1528. Li Zhilin LJ, Liu Xianming, Yao Shengfu. Investigation and Epidemiological Alysis on the Parasitic Fleas of Citellus flavipectus in nhua Mountain, Haiyuan County, Ningxia. *Chinese Jourl of Endemic Disease Control* 1998; **13**(5): 306-7.
1529. Wang Zicun LS, Gao Guiying, Wu Xianglin. Discussion on Epidemic Characteristics of pestis in Ningxia Gerbil Focal Area and Its Control Countermeasures. Endemic Diseases Bulletin; 2016; 2016.
1530. Yang Xiaoyan JJ, He Jian, Jin Yong, Dai Ruixia, Xin Youquan, Zhao Haihong, Feng Jianping, Wu Haisheng, Wu Hailian, Wei Baiqing. Epidemiological alysis of pestis in Qilian County from 1958 to 2014. *Medical animal control* 2016; (8): 3.
1531. Li Min WL. Epidemic dymics of pestis in Qinghai Province from 1999 to 2000 and its control countermeasures. *Endemic Diseases Bulletin* 2001; **16**(4): 41-2.
1532. Baiqing W. Study on the Epidemic Law of pestis in Microtus fuscus. *Chin J Zoonoses* 2004; **20**(6): 3.
1533. Li Fan WL, Yuan Ping. Pestis tural Focus of Marmota himalaya in Sichuan Province. *Chinese Jourl of Endemic Disease Control* 2000; **16**(004): 95-7.
1534. Yang Junping HF. Distribution of Rattus flavipectus in Urad Middle Banner and Investigation of Animal Epidemic of pestis. *Science, Technology and Economy in Inner Mongolia* 2000; (5): 1.
1535. Chen Hong XG, Li Guangqing, Liao Xuechun, Wang Dunzhi, Duan Yongjun, Yan Dongli. First detection of pestis FI antibody from marmota himalaya serum in Sichuan. *Chinese Jourl of Endemic Disease Control* 1991.
1536. Xing Xueli SX, Chen Yali, He Weili. Surveillance Report on pestis of Gerbil unguiculatus in Sunid Right Banner from 1990 to 1993. *Chinese Jourl of Endemic Disease Control* 1995.
1537. Fan Mengguang LJ, Wei Ruiping, Mi Jingchuan, Zhang Zhongbing, Zhao Gang. Epidemiological investigation of animal pestis in the focus of pestis of Brandt's vole type in Chi --

discovery of pestis of gerbil type. *Chinese Jourl of Endemic Diseases* 2014.
1538. Yang Junping HF. Distribution of Rattus flavipectus in Urad Middle Banner and Investigation of Animal Epidemic of pestis. *Science, Technology and Economy in Inner Mongolia* 2000.
1539. Ainevar Kulban S, Ma Junjie, Bai Shengxing, Wang Xinhua, Du Man, Chen Wenliang. Investigation on pestis tural foci of desert animals in Urumqi. *Endemic Diseases Bulletin* 2010.
1540. Wang Xinhua TX, Du Man, Ma Yuexin. Investigation and Alysis of pestis Foci in Gobi Desert, Midong District, Urumqi. *Disease prevention and control notification* 2015.
1541. Lang Bingju MW, Bai Xuefeng. Alysis on the current situation and epidemic situation of pestis focus in Xilingol League. *Chinese Jourl of Endemic Disease Control* 2009.
1542. Wei Baiqing YS, Tian Fuzhang, Zhang Fabin, Dan Zhengcai Ren, Pu Bucai Ren. A human gland pestis caused by playing with plateau pika. *Endemic Diseases Bulletin* 1998.
1543. Bai Kezhong WX, Liu Wanlin, Ma Qiancheng, Zhu Mingfu. Dymic monitoring of human and animal pestis epidemic in Changji Prefecture, Xinjiang. *Pharmacoeconomics in Chi* 2013.
1544. Teng Yunfeng ZH, Xie Xingchu. Discovery of tural Foci of pestis in Shawan, Xinjiang. *Endemic Diseases Bulletin* 1992.
1545. Wang Shusheng LY, Huang Bijin, Tang Peichun, Hadley Khan, Yeerken. Distribution of tural foci of pestis in mountainous areas of Shawan County, Xinjiang. *Endemic Diseases Bulletin* 1996.
1546. Wang Shusheng HB, Liu Yongfu, Hadrihan, Wusman River, Yeerken, Guijiushan, Yasheng. There is a tural focus of pestis in Lujiaowan, the piedmont of Tianshan Mountain, Shawan County, Xinjiang. *Endemic Diseases Bulletin* 1989.
1547. Huang Bijin GZ, Liu Yongfu, Hadley Khan, Tang Peichun, Wang Kunyan, Yasen, Gui Jiushan, Wang Yuping. A Brief Report on Human pestis Prevalence and Control in Tianshan Mountain Area of Shawan County, Xinjiang. *Endemic Diseases Bulletin* 2004.
1548. Jiang Wei SJ, Wang Xinhui, Li Donghui, Albumitti, Pulati, Lei Gang, An Wenyan, Buren Mingde, Muheyati, Aizezi, Yu Xin. Pestis tural focus found in Alatao Mountain, Xinjiang Uygur Autonomous Region. *Endemic Diseases Bulletin* 2004.
1549. Jiang Wei SJ, Wang Xinhui, Li Donghui, Albumitti, Pulati, Lei Gang, An Wenyan, Buren Mingde, Muheyati, Aizezi, Yu Xin. Pestis tural focus found in Alatao Mountain, Xinjiang Uygur Autonomous Region. *Endemic Diseases Bulletin* 2011; **27**(1): 1.
1550. Sun Zhou HC, Nimuge, Julaiti. Surveillance Report on pestis in Jinghe County, Xinjiang Uygur Autonomous Region in 1999. *Disease prevention and control notification* 2001; **16**(1): 2.
1551. Yin Xiaoping YZ, Jiao Wa, Dai Xiang, Re , Bu Renmingde, Ye Ruiyu. Great Gerbil pestis Found in Mayle Mountain Valley, Xinjiang Uygur Autonomous Region. *Disease prevention and control notification* 2011; (1): 1.
1552. Wang Shusheng HB, Hadley Khan, Liu Yongfu, Wu Xiaofang, Wang Chaoju, Yang Kai, Elken, Yu Xin, Zhao Fei, Liu Chengquan. Clinical Report of Three pestis Cases in Shawan County, Xinjiang Uygur Autonomous Region. *Endemic Diseases Bulletin* 1990; **5**(4): 3.
1553. Li Bo AR, Wang Xinhui, Re Turdi, Sun Shi, Li Bing, Zhang Xiaobing, Albumiti Maituohuti. Monitoring and Alysis of pestis tural Foci in Wusugurtu, Xinjiang Uygur Autonomous Region. *Disease prevention and control notification* 2014.
1554. Guoqiang Z. Surveillance Report on pestis in Bayingou, Wusu City, Xinjiang Uygur Autonomous Region. *Disease prevention and control notification* 1999; (2).
1555. Albumiti Maituoheti BM, Lei Gang, Xu Bingchen, Chen Jianxing, Chang Zhiqiang. Surveillance Report on pestis in Guertu Mountain Area, Wusu City, Xinjiang Uygur Autonomous Region in 1998. *Disease prevention and control notification* 2000; **15**(1): 58-9.
1556. Zhang Hongyou SG. Overview of pestis of Citellus undulatus in Xinjiang Uygur Autonomous Region. *Disease prevention and control notification* 1991; **006**(001): 114-7.
1557. Ma Junjie WD, Tuerhon Kubahun. Thoughts on pestis Surveillance in Yili Kazak Autonomous Prefecture, Xinjiang. *Disease prevention and control notification* 2013.
1558. Feng Jianping QM, Wei Boqing, Xiong Haoming, Jinxing, Jinyong, Yang Xiaoyan, Jin Juan, Xin Youquan, Tang Xinyuan, Wang Mei, Dai Ruixia. Etiological Alysis and Epidemiological Significance of pestis in Xinghai County. *Medical animal control* 2015; (12): 3.
1559. Zhang Wanrong SZ. Diagnosis and treatment of a case of glandular pestis. *Endemic Diseases Bulletin* 1990.
1560. Jiao Batai WG. An investigation report on an epidemic of pestis among animals. *Chin J Zoonoses* 1999; **15**(04): 0-.
1561. Wei Baiqing LH, Mu You, Wei Zhiliang, Zhou Yuwei, Guo Qing, Zhu Yingde. A human glandular pestis caused by the stripping of Himalayan marmot. *Endemic Diseases Bulletin* 2007; **22**(2): 2.
1562. Wei Baiqing YS, Tian Fuzhang, Zhang Fabin, Dan Zhengcai Ren, Pu Bucai Ren. A human

glandular pestis caused by plateau pika. *Chinese Jourl of Endemic Disease Control* 1997; **12**(5): 2.
1563. Guo Wentao ZC, Jialuo, Du Pengcheng, Wu Haisheng, Lu Zhansheng. Pathogen surveillance of pestis in Yushu area from 2005 to 2009. *Qinghai Jourl of Animal Husbandry and Veteriry Medicine* 2013; **43**(6): 2.
1564. Dong Xingqi WG, Zhang Liyun, Xiang Yongfen, Yao Yunbo, He Yinghui, Su Yong, Li fa, Yang Dingshou. Investigation and Alysis of an Outbreak of pestis in Yuanjiang County. *Endemic Diseases Bulletin* 1993; **008**(002): 68-70.
1565. Tianyuan L. Pestis F-1 antibody detected in Rattus norvegicus in Binchuan County, Yunn Province. *Chinese Jourl of Endemic Disease Control* 1988; (6).
1566. Huang Y. Tural focus of pestis in Yunn. *Endemic Diseases Bulletin* 1987; (2).
1567. Zhang Yunsheng LJ, Yang Jingshu, He Lifang, Shu Ziyun. Epidemiological investigation and control of pestis outbreak among rats in a village of Luoping County, Yunn Province. *Chinese Jourl of Vector Biology and Control* 2003; **14**(3): 222-3.
1568. Su Liqiong LY, Wu Hesong, Yang Zhiming, Hong Mei. Epidemiological characteristics of human pestis in yunn province from 1986 to 2014. *Chinese Jourl of Vector Biology and Control* 2016; **27**(4): 4.
1569. Huang Ningbo WM, Ma Yongkang. Epidemic Characteristics of pestis of Rattus domesticus in Yunn Province (Alysis of bacteriological test results of pestis of Rattus domesticus in 1982-1998). *Endemic Diseases Bulletin* 2000; (001): 015.
1570. Shouqin L. Current situation and countermeasures of pestis control in Jianchuan County, Yunnan Province. *Endemic Diseases Bulletin* 2002; **17**(3): 48-9.
1571. Xiong Mengtao LG, Duan Xingde, Kong Guangcheng, Ding Baochang, Xu Yunqiao, Zhou Baoxiang, Li Yingsheng. Epidemiological survey of pestis in Menghai County, Yunn Province. *Endemic Diseases Bulletin* 1993; **008**(002): 71-3.
1572. Dong Xingqi SZ, Liang Yun, Wang Guoliang, Yu Guolin, Ma Yongkang. Study on the Current Situation of pestis of Wild Rat in Yunn Province and the Adjacent Relationship between Plague Foci. *Jourl of Kunming Medical College* 2009; **30**(8): 21-5.
1573. Li Jizhong FS. Epidemiological investigation on a case of glandular pestis in Yingjiang County, Yunnan Province. *Medical animal control* 2000; **16**(1): 44-5.
1574. Zhao Wenhong GM, Duan Biao, Su Liqiong. Relationship between seasol fluctuation of flea index and pestis in Yingjiang County, Yunn Province. *Medical animal control* 2016; (3): 3.
1575. Gao Zihou DX, Zhao Wenhong, Song Zhizhong, Yang Zhiming. Population distribution and seasol fluctuation alysis of pestis host animals in Yingjiang County, Yunn Province. *Medical animal control* 2008.
1576. Xiong Mengtao YQ, Zhao Wenhong. Epidemiological investigation and control of pestis among rats in Yunxian County. *Jourl of Preventive Medicine Information* 1996; **12**(4): 2.
1577. Zhang Anning QW, Rong Binguo, Shi Zuohong. Epidemiological investigation of pestis among rats and human pestis in Zhangye area. *Chinese Jourl of Endemic Disease Control* 2000; **15**(6): 4.
1578. Zhang Anning QW, Rong Binguo, Shi Zuohong. Epidemiological investigation of pestis among rats and human pestis in Zhangye area. *Chinese Jourl of Endemic Disease Control* 2003; **18**(2): 2.
1579. Gao Shaodong KX. Alysis of tural foci of pestis in Huaiji County, Zhaoqing City. *Chinese Practical Medicine* 2008; **3**(34): 2.
1580. Zhao Zhiya TY, Lu Miaogui, Li Yanjin, He Suhai. Pestis surveillance in Zhejiang Province, 1989-1990. *Zhejiang Preventive Medicine and Disease Surveillance* 1992; **3**(6): 2.
1581. Zhao Zhiya LM, Shi Guoxiang, Li Yanjin. Alysis of the surveillance results of pestis in zhejiang province in 1992. *Zhejiang Preventive Medicine and Disease Surveillance* 1993; (6): 2.
1582. Zhao Zhiya FG, Weng Jingqing, Yao Pingping, Li Minhong, Lu Qunying, Zhu Zhiyong, Zhu Hanping. Surveillance of hemorrhagic fever with rel syndrome in zhejiang province from 1997 to 1999. *Chinese Jourl of Vector Biology and Control* 2000; (4): 290-2.
1583. Zhao Zhiya LM, Shi Guoxiang, Li Yanjin. Results and Alysis of Seroepidemiological Surveillance of pestis in Zhejiang Province. *Chinese Jourl of Endemic Disease Control* 1994; **9**(1): 2.
1584. Chen Jinhua WZ, Zhu Zhihong, Chen Bo, Lou Ting, Shi Guoxiang. Investigation and Alysis of Yersinia in Rodents in Yiwu City, Zhejiang Province. *Chinese Jourl of Vector Biology and Control* 2015; (003): 026.
1585. Guo Rong DX, Cao Hanli, Xia Lian, Abelmiti, Abu Likmu, Wang Xinhui, Azati, Jiang Wei, Li Bing, Zhang Xiaobing, Lei Gang, Wang Qiguo, Luo Tao, Meng Weiwei, Buren Mingde, Je, Chen Yan, Zhang Yujiang. Spatial and Temporal Distribution of Animal pestis Epidemic in tural Focus of pestis in Junggar Basin. *Chinese Jourl of Epidemiology* 2014; **35**(2): 5.

1586. Bo W. Isolation, biological characteristics and genome study of rat bacteriophage from Heqing rodent plague foci. *Kunming Medical University* 2019.
1587. Fulati Yimingjiang XK, Jinhua, Li Donghui. Surveillance and Alysis of pestis in Wenquan County, Xinjiang, 2002-2004. *Chinese Jourl of Endemic Disease Control* 2006; **21**(6): 27-8.
1588. Duan Yongjun WL, Luo Zhidanba, Xu Guangrong, Xie Fei, Li Guangqing, Zong Kanggui, He Qianjun, Qi Teng, Li Fan, Wu Chaoxue, Danbazeli, Ni Ping. Animal pestis surveillance in Shiqu County, Sichuan Province, 2001-2009. *Jourl of Preventive Medicine Information* 2011; **27**(3): 4.
1589. Zhang Dianming HB. Monitoring Report on pestis in Huining County, Gansu Province, 2001-2003. *Endemic Diseases Bulletin* 2009; (4): 1.
1590. Yin Wenjiang ZH, Xie Weigang, Ma Yun, Kang Baocheng. Monitoring Report on tural Focus of pestis of Gerbil in Junggar Basin, Wujiaqu, Xinjiang Uygur Autonomous Region in 2010. *Corps Medicine* 2012; (4): 3.
1591. Yang Liansheng SG. Pestis focus of Citellus dauricus in Chifeng City. *Medical animal control* 2007; **23**(12): 2.
1592. Cao Guofeng SG, Zhu Jianxin. Treatment and alysis of pestis epidemic area among rats in Aohan Banner of Chifeng City in 2002. *Medical animal control* 2007; **23**(12): 930-.
1593. Chen Yongjin LY, Yin Guizhu, Xu Zuxiang, Yu Jiufei. Isolation of Yersinia enterocolitica from mice. *Chin J Zoonoses* 1987; (01): 37-8.
1594. Yang Zongke YW, Ma Chun. Alysis on Epidemic Factors of pestis in Haiyuan County. *Disease surveillance* 2008.
1595. Liu Jinhua YX, Long Zhimei, Yang Farong, Chen Weijun, Tian Xiaodong, Lu Zhenzhi. Investigation on Spotted Fever Foci in Hain Island. *PLA Jourl of Preventive Medicine* 1996; **14**(5): 3.
1596. Song Fenglin WG, Xue Fang, Li Jianxun, Liu Hongwen, Fan Dehai, Li Changsheng, Jiang Yilin, Gao Yufeng, Qin Feng, Du Haifang, Wang Xiaozhong, Li Ning, Wang Jianxue, Wang Yimin, Wang Weijun, You Zhengai, Sun Yuling, Cui Yi, Tan Xueen, Dong Ping, Li Jingge. Study on Pathogens Carried by Vectors at Frontier Port. *Chinese Jourl of Frontier Health and Quarantine* 2009; **32**(5): 7.
1597. Yang Huayuan FZ, Lu Ruiming, Zhang Tao, Liu Mingquan, Zhang Shiyan, Mai Hai, Liang Qiuguang, Liang Lian. Study on the ture of pestis focus in guangdong province. *Jourl of Tropical Medicine* 2006.
1598. Yuan Gaolin ZB, Chen Jianhua, Huang Lixiong, Xie Wenqiu. Investigation report on four host animals and vector insects of mouse borne diseases in Gutian Western Village. *Fujian Medical Jourl* 1986.
1599. Cao Yifan YR. Investigation on Parasitic Worms of Plateau Pika. *China Veteriry Science and Technology* 1998; **28**(5).
1600. Zhao Chengxiang B, Shi Gao, Fan Mengguang. Surveillance and Alysis of pestis in Mongolian Gerbils from 2001 to 2010. *Medical animal control* 2013; (3): 3.
1601. Ma Yongkang GY, Duan Xingde, Gao Zihou, Li Junyong. Investigation and Alysis of pestis Outbreak in Mojiang County. *Chinese Jourl of Endemic Disease Control* 1997; **12**(6): 2.
1602. Yang Zhiming HJ, Tao Yongguo. Discovery and Alysis of Antibody to pestis F-1 in Maguan County. *Chinese Jourl of Endemic Disease Control* 1998.
1603. Chen Weijian CR, Chen Yongjin, Lian Qinbao, Mei Jianhua. Alysis of current pestis epidemic situation in Lishui area. *Public Health Magement in Chi* 2001; **17**(2): 135-6.
1604. Jiufei Y. Pestis FI antibody detected from Apodemus agrarius in Lishui area. *Zhejiang Disease Surveillance* 1989; (S3): 2-3.
1605. Huang Zhiguang HJ, Feng Xianlei, Ye Gang, Li Wei. Investigation on the damage assessment of pestis in Karamay. *Chinese Jourl of Preventive Medicine* 2011.
1606. Yu Dejiang LQ, Deqing Duoji Ciren. The epidemic area of pestis of Marmota himalaya in Anduo County, qu Prefecture. *Tibetan Medical Jourl* 1990; (001): 011.
1607. Zhixian Y. Monitoring and alysis of pestis in pestis tural focus of Mongolian marmot in Manzhouli from 2001 to 2013. *Chinese Jourl of Vector Biology and Control* 2014.
1608. Fan Mengguang HX, Wang Lanfang, Liu Jun. Alysis on Epidemic Characteristics of pestis in Gerbils unguiculatus in Recent Years. *Neimenggu Preventive Medicine* 2000; **25**(2): 3.
1609. Xu Guangrong YS, Fu Qingpei, Duan Yongjun, Li Fuzhong, Wang Limao 3 Shi Tianji, Huang Guangyue, Li Guangqing, Wu Guokang, Shi Xianling, Liu Yuanxia. First Isolation of pestis pestis from Shiqu County, Sichuan Province. *Endemic Diseases Bulletin* 1991.
1610. Gao Zhengqin YB. Fungal Diversity of Grey Hamster in Xinjiang, China. *Chinese Jourl of Zoonoses* 2014; **30**(7): 709-17.
1611. Li X, Yang Y, Zhang X, et al. Isolation of Penicillium marneffei From Soil and Wild Rodents in Guangdong, SE China. 2011; **172**(6): 447-51.
1612. Huang X, He G, Lu S, Liang Y, Xi LJMB. Role of R hizomys pruinosus as a natural animal

host of P enicillium marneffei in Guangdong, China. 2015; **8**(4): 659-64.
1613. Li Jushang DZ, Pan Lequan, Yao Cuili. A Mycological Study Report on Penicillium in the tural Belt of the Silver Star Bamboo Rat in Guangxi Zhuang Autonomous Region. *Chin J Zoonoses* 1995; **17**(1): 2.
1614. Luo Hong LL, Liu Donghua, Liu Xiaojun, Cao Cunwei, Wu Yi, Wei Gao. Microsatellite Polymorphism of Penicillium marneffei Parasitic in Wild Silver Star Bamboo Rat in Guangxi. *The Chinese Jourl of Dermatovenereology* 2008; **22**(2): 3.
1615. Xiqing L. Study on Penicillium marneffei infection in HIV/AIDS patients and wild bamboo rats in Guangdong. *Sun Yat-sen University* 2009.
1616. Lu S, Li D, Xi L, Calderone R. Interplay of interferon-gamma and macrophage polarization during Talaromyces marneffei infection. *Microb Pathog* 2019; **134**: 103594.
1617. Li Shan DZ, Ma Yun. Study on Penicillium marneffei carried by rodents in Guangxi. *Guangxi Medicine* 1995; **17**(1): 2.
1618. Qi Zhirong YW, Song Xiusheng, Guo Shuifa, Adel, Yang Wenchuan, Lin Yuguang, Hong Lingxian, Guan Jiazhen, Peng Wenfeng. Epidemiological Investigation of Human and Animal Hydatid Disease in Tacheng District, Xinjiang. *Endemic Diseases Bulletin* 1995; **010**(002): 50-4.
1619. Yang Yingzhong YZ, Sun Xiangyong. Serological investigation on echinococcosis of Marmota cinerea in Tianshan, Mas County. *Endemic Diseases Bulletin* 1994.
1620. He Duolong WH. Epidemiological Evaluation of Hydatid Disease in Zeku County, Qinghai Province. *Endemic Diseases Bulletin* 2001; **16**(4): 3.
1621. Chen Gen BG, Han Jian, Jing Tao. Investigation on infection of Capillaria hepatica in plateau pika in Maqu County, Gansu Province. *Chinese Jourl of Pathogenic Biology* 2011; **6**(3): 3.
1622. Xiong Mengtao YG, Wu Xing, Fan Chongzheng, Tao Huihui, Wang Guoliang, Cai Wenfeng. Investigation on small mammals infected with Capillaria hepatica in Yongjian Township, Weishan County. *Endemic Diseases Bulletin* 1999; **14**(003): 58-60.
1623. Huang Di CD, Zhang Yun, Li Xiaomin, Zhan Ximei. Investigation on the infection of Angiostrongylus cantonensis in sils and rodents in Guangdong. *Chinese Jourl of Pathogenic Biology* 2008; **3**(9): 695-6.
1624. Deng ZH, Zhang QM, Huang SY, Jones JL. First provincial survey of Angiostrongylus cantonensis in Guangdong Province, China. *Trop Med Int Health* 2012; **17**(1): 119-22.
1625. Geng Yijie HD, Xie Xu, Deng Pingjian, Gao Shitong, Li Xiaoheng, Wang Jingzhong, Tan Jingguang, Zhang Renli. Investigation on the infection of Angiostrongylus cantonensis in rodents in Shenzhen. *Chinese Jourl of Disease Control* 2011; **38**(3): 3.
1626. Zhao Hongmei SJ, Liu Chunhua, Zhao Shaoyang, Wang Fangyan. Investigation of Schistosoma japonicum infection in wild mice in Sihu area, Hubei Province. *Chinese Jourl of Zoonoses* 2009; **25**(009): 919-20.
1627. Zhu Hongmei HJ, Zhuo Mingying, Xie Kejin, Zhang Zhiping, Yang Mingjian, Wen Hui. Investigation on tural foci of Angiostrongylus cantonensis in northern Fujian. *Chinese Jourl of Zoonoses* 2010; **26**(10): 986-8.
1628. Tang Chongti TL, Qian Yuchun, Cui Guiwen, Kang Yumin, Lu Hongchang, Shu Limin. Investigation on Pathogen Species and Epidemiology of Alveolar Hepatic Hydatid in New Balhu Right Banner, Eastern Inner Mongolia. *Jourl of Xiamen University (tural Science Edition)* 2001; **40**(2): 9.
1629. Hu X, Du J, Tong C, et al. Epidemic status of Angiostrongylus cantonensis in Hainan island, China. *Asian Pac J Trop Med* 2011; **4**(4): 275-7.
1630. Lan Yuqing ZX. Angiostrongylus cantonensis obtained from rat physical examition and its distribution. *Preventive medicine literature information* 2001.
1631. Deng Zhuohui ZQ, Lin Guangli, Huang Shaoyu, Zhang Yi, Lv Shan, Liu Hexiang, Hu Ling, Pei Fuquan, Wang Jinlong, Ruan Caiwen. Investigation on the Epidemic Focus of Angiostrongylus cantonensis in Guangdong Province. *Chinese Jourl of Parasitology and Parasitic Diseases* 2010; (1): 5.
1632. Yang Fazhu ZS, Tu Zhaoping, Ouyang Rong, Xie Hanguo. Comparison of the epidemic situation of angiostrongyliasis cantonensis on both sides of the Taiwan Straits. *Chinese Jourl of tural Medicine* 2008; (2): 2.
1633. Zhang Renli GS, Geng Yijie, Huang Da, Chen Muxin, Liu Jianping, Wu Yuanliang, Zhen Yin, Dai Chuanwen, Zhang Qiwen, Wu Taishun, Ma Zhichao, Chen Wusheng, Li Dalin. Investigation on the Epidemic Focus of Angiostrongylus cantonensis in Shenzhen and Alysis of Its Epidemic Characteristics. *Chinese Jourl of Epidemiology* 2008; **29**(6): 4.
1634. Zilin Z. Investigation on Trichinella hepatica in Wuhan. *Sichuan Animals* 1990; (01): 39.
1635. Ye Yumei WD, Tu Yufa. Investigation of Toxoplasma gondii infection in animals in Yunn Province. *Chinese Jourl of Parasitology and Parasitic Diseases* 2002; (4).
1636. Wan Xinrong JY, Zhao Tianxiao, Guo Pengfei, Shi Yansheng, Bao Xiang, Wang Guanghe. The

relationship between the tural foci of liver trichinellosis and the distribution of rodents in Xilingol area. *Jourl of Zoology* 2007; **42**(1): 14-9.
1637. Zhang Jingfen WS, Zhang Jingbo. Pathological Observation on Infection of Capillaria hepatica in Citellus dauricus. *Chinese Jourl of Endemic Disease Control* 1994; **9**(1): 2.
1638. Wenbing Y. Investigation on animals infected with Trichinella spiralis in Inner Mongolia. *Acta Academiae Medicie Neimongol* 1991; (2): 2.
1639. Hu QA, Zhang Y, Guo YH, et al. Small-scale spatial analysis of intermediate and definitive hosts of Angiostrongylus cantonensis. *Infect Dis Poverty* 2018; **7**(1): 100.
1640. Zhang XX, Cui LY, Cao SZ, et al. [Preliminary survey on the host of Angiostrongylus cantonensis in three plateau lakes of Yunnan Province]. *Zhongguo Ji Sheng Chong Xue Yu Ji Sheng Chong Bing Za Zhi* 2014; **32**(2): 139-42, 47.
1641. Chen CX, He HF, Yin Z, et al. [Investigation on snails Achatina fulica and Pomacea canaliculata infected with Angiostrongylus cantonensis in Panyu region of Guangzhou City]. *Zhongguo Xue Xi Chong Bing Fang Zhi Za Zhi* 2012; **24**(3): 336-8.
1642. Sun R, Li ZY, He HJ, et al. Molecular cloning and characterization of a matrix metalloproteinase, from Caenorhabditis elegans: employed to identify homologous protein from Angiostrongylus cantonensis. *Parasitol Res* 2012; **110**(5): 2001-12.
1643. Deng ZH, Lv S, Lin JY, Lin RX, Pei FQ. An outbreak of angiostrongyliasis in Guanging, People's Republic of China: migrants vulnerable to an emerging disease. *Southeast Asian J Trop Med Public Health* 2011; **42**(5): 1047-53.
1644. Pan B, Wu J, Ruan CW, et al. [Investigation on Angiostrongylus cantonensis infection in rodents in Guangdong Province]. *Zhongguo Ji Sheng Chong Xue Yu Ji Sheng Chong Bing Za Zhi* 2011; **29**(4): 289-92.
1645. Chen D, Zhang Y, Shen H, et al. Epidemiological survey of Angiostrongylus cantonensis in the west-central region of Guangdong Province, China. *Parasitol Res* 2011; **109**(2): 305-14.
1646. Qu ZY, Yang X, Cheng M, et al. Enzootic angiostrongyliasis, Guangdong, China, 2008-2009. *Emerg Infect Dis* 2011; **17**(7): 1335-6.
1647. Wei FR, Liu HX, Lv S, Hu L, Zhang Y. [Multiplex PCR assay for the detection of Angiostrongylus cantonensis larvae in Pomacea canaliculata]. *Zhongguo Ji Sheng Chong Xue Yu Ji Sheng Chong Bing Za Zhi* 2010; **28**(5): 355-8.
1648. Deng ZH, Zhang QM, Lin RX, et al. [Survey on the focus of Angiostrongylus cantonensis in Guangdong Province]. *Zhongguo Ji Sheng Chong Xue Yu Ji Sheng Chong Bing Za Zhi* 2010; **28**(1): 12-6.
1649. Zhou Z, Barennes H, Zhou N, Ding L, Zhu YH, Strobel M. [Two outbreaks of eosinophilic meningitis in Yunann (China) clinical, epidemiological and therapeutical issues]. *Bull Soc Pathol Exot* 2009; **102**(2): 75-80.
1650. Zhang RL, Chen MX, Gao ST, et al. Enzootic angiostrongyliasis in Shenzhen, China. *Emerg Infect Dis* 2008; **14**(12): 1955-6.
1651. Zhang Y, Huang D, Tan QM, Chen DX, Zhan XM. [Epidemiological investigation of Angiostrongylus cantonensis in Jiangmen of Guangdong Province]. *Zhongguo Ji Sheng Chong Xue Yu Ji Sheng Chong Bing Za Zhi* 2008; **26**(5): 370-3.
1652. Lv S, Zhang Y, Steinmann P, Zhou XN. Emerging angiostrongyliasis in Mainland China. *Emerg Infect Dis* 2008; **14**(1): 161-4.
1653. Liu HX, Zhang Y, Lv S, et al. [A comparative study of three methods in detecting Angiostrongylus cantonensis larvae in lung tissue of Pomacea canaliculata]. *Zhongguo Ji Sheng Chong Xue Yu Ji Sheng Chong Bing Za Zhi* 2007; **25**(1): 53-6.
1654. Lin JX, Li YS, Zhu K, et al. [Epidemiological study on group infection of Angiostrongylus cantonensis in Changle City]. *Zhongguo Ji Sheng Chong Xue Yu Ji Sheng Chong Bing Za Zhi* 2003; **21**(2): 110-2.
1655. Liang HK. [The infection of Angiostrongylus cantonensis in definitive and intermediate hosts in Guangzhou 1979-1982]. *Zhonghua Liu Xing Bing Xue Za Zhi* 1984; **5**(4): 245-8.
1656. Ting B, Ho JZ, Chu TC, Luo YS. [A survey of Angiostrongylus cantonensis in Guangzhou]. *Ji Sheng Chong Xue Yu Ji Sheng Chong Bing Za Zhi* 1984; **2**(1): 25-7.
1657. Lisa L, Yousong L, Xiaonong Z, et al. Discovery of six new host species of angiostrongylus cantonensis in fujian province and annual change of infection rate in epidemic focus. *Chinese Journal of Zoonoses* 2006; **22**(6): 5.
1658. Zhuohui D, Qiming Z, Jinyi L, et al. Investigation on the Natural Focus of Angiostrongylus cantonensis in Guangdong Province. *Preventive Medicine in South China* 2008; **34**(4): 4.
1659. Fuhua L, Xiaomei Z, Yanzhong L, Hong T. Investigation on the Natural Focus of Angiostrongylus cantonensis in Yunnan Province. *Chinese Journal of Pathogenic Biology* 2008; **3**(1):

53-6.
1660. powerful A, Xin L, Yong H, et al. Investigation on the Epidemic Focus of Angiostrongylus cantonensis in Shandong Province. *Chinese Journal of Pathogenic Biology* 2007; **2**(2): 2.
1661. Hongman Z, Yuguang T, Xueming L, et al. Investigation on the Epidemic Focus of Angiostrongylus cantonensis in Guangxi. *Tropical Diseases and Parasitology* 2007.
1662. Jian L, Ximin H, Shanqing W, Dong L, symbol. Investigation on natural foci of Angiostrongylus cantonensis in Ding'an County, Hainan Province. *Chinese Tropical Medicine* 2007; **7**(03): 408-9.
1663. Shaorong C, Feng C, Wen F, et al. Investigation on the Natural Epidemic Focus of Angiostrongylus cantonensis in West Lake Area of Eryuan County, Dali Prefecture. *Chinese Journal of Pathogenic Biology* 2011; **6**(5): 3.
1664. Liufang F, Zhenfeng Z, Liqun L, et al. Investigation on the Natural Focus of Angiostrongylus cantonensis in Conghua District, Guangzhou City. *Medical animal control* 2022; (003): 038.
1665. Liyun C. Application of GIS in Epidemiological Study of Angiostrongylus cantonensis in Yilong Lake and Qidang Lake Watershed, Yunnan Province: Dali University; 2013.
1666. Yi Z, Lushan, Kun Y, et al. First Survey on the Distribution of Angiostrongylus cantonensis Natural Foci in China. *Chinese Journal of Parasitology and Parasitic Diseases* 2009; **28**(6): 12-512.
1667. Yunhai G, Yi Z, Lushan, Ling H, Zonglu Z, Xiaonong Z. Angiostrongylus cantonensis surveillance and new discovery of natural foci in Yunnan in 2011. The 9th Member Congress and the 15th Academic Seminar of the Branch of Shellfish Science of the Zoological Society of China and the Chinese Society of Oceanography and Limnology.
1668. Ping X, Deren W. Investigation on natural foci of Angiostrongylus cantonensis in Beihai, Guangxi. *International Journal of Medical Parasitic Diseases* 2013; **040**(002): 67-70.
1669. Investigation of Angiostrongylus cantonensis Epidemic Focus in Shenzhen City and Study on Diagnostic Methods of Angiostrongylus cantonensis. *International Journal of Medical Parasitic Diseases* 2009.
1670. Hongman Z, Yuguang T, Xueming L, et al. Investigation on the Epidemic Focus of Angiostrongylus cantonensis in Guangxi. *Tropical Diseases and Parasitology* 2007; **5**(2): 79-81.
1671. Ximin H, brocade Csh, Jian L, Shanqing W. Investigation on the Epidemic Focus of Angiostrongylus cantonensis in Hainan Province. *Chinese Tropical Medicine* 2007; **7**(11): 2.
1672. Yousong L, Jinxiang L, Xianrang X, Baojian C, Yuzhu C. Investigation on the first human angiostrongyliasis foci in Fujian Province. *Tropical Diseases and Parasitology* 2001; (1): 3.
1673. Pinghua C, Xinguang Z, Zewu Z. Investigation on the Epidemic Focus of Angiostrongylus cantonensis in Dongguan City. *Chinese Tropical Medicine* 2012; **12**(7): 3.
1674. Rongyan Z, Rong O, Chenxin L, Baojian C, Yanrong L, Lisa L. Investigation on the Natural Focus of Angiostrongylus cantonensis in Fujian Province. *Journal of Strait Preventive Medicine* 2017; **23**(5): 3.
1675. Hongchun T, Tianbo H, Qingfu Z, et al. Investigation on the Epidemic Focus of Angiostrongylus cantonensis in Some Areas of Sichuan Province. *Parasitic Diseases and infectious diseases* 2009; **7**(1): 2.
1676. Gou GS, Yefen, Lisa L, Rongyan Z. Investigation on the Epidemic Focus of Angiostrongylus cantonensis in Sanming City. *Chinese Journal of Pathogenic Biology* 2007; **2**(6): 2.
1677. Baoxian H, Xiaoping Z, Haihong S, Anmei S. Investigation on the Natural Focus of Angiostrongylus cantonensis in Nanhui District, Shanghai. *Chinese Tropical Medicine* 2008; **8**(3): 2.
1678. Tianmei L, Wen F, Kerong L, Shaorong C, Yuhua L, Jia L. Surveillance results of Angiostrongylus cantonensis infection in Dali from 2013 to 2014. *Parasitic Diseases and infectious diseases* 2016; **14**(2): 4.
1679. Liang Haokun SH, Xu Bingkun. Investigation on the Infection of Angiostrongylus cantonensis Ending Host, such as Brown House slug, and Its Successive Host, Toad in Guangzhou. *Jourl of Parasitology and Parasitic Diseases* 1983; **1**(4): 53-.
1680. Liang Haokun SH, Xu Bingkun. Investigation on the infection status of end-host, intermediate host and transfer host of Angiostrongylus cantonensis in Guangzhou. *Jourl of Guangzhou Medical College* 1983.
1681. Wu Guanghua JZ, Zhou Jun. Prevention and control of angiostrongyliasis cantonensis and sils. *Intertiol Jourl of Medical Parasitic Diseases* 2009; **36**(6): 5.
1682. Zhu Tiancheng SH, Ye Xiaoguang, Ding Bulan. Investigation on rodents as the fil host of Angiostrongylus cantonensis in Youhao Farm, Xuwen County, Guangdong Province. *Chin J Zoonoses* 1993; **9**(6): 2.
1683. Lin Guohua YC, Cai Maorong, Huang Mingsong, Zhou Yaoxiong, Fang Yanyan, Cheng Youzhu. Investigation on the Infection of Angiostrongylus cantonensis with Rodent Hosts in Zhangzhou

City, Fujian Province. *Chinese Jourl of Parasitology and Parasitic Diseases* 2016; **34**(2): 4.
1684. Li Lisa ZR, Lin Jinxiang, Fang Yanyan. Investigation on rodents infected with Angiostrongylus cantonensis in Fujian Province. *Chinese Jourl of Zoonoses* 2010; **26**(2): 186-8.
1685. Huang Jinyuan ZL, Yang Fazhu, Tu Zhaoping, Zhang Yingzhen, Huang Xiaohong. Investigation on the infection of Angiostrongylus cantonensis in rats and sils in Jiangle County, Fujian Province. *Chinese Jourl of Parasitic Disease Control* 2002; (3).
1686. Yang Fazhu TZ, Zhang Yingzhen, Huang Xiaohong, Zheng Guobin. Investigation on Angiostrongylus cantonensis and Its Intermediate Host in Fujian Province. *Medical animal control* 2001; (2): 3.
1687. Xiao Fangzhen PX, Xu Guoying, Chen Yang, Lin Daihua, Deng Yanqin. Investigation and genetic identification on Babesia infection in rodents in some areas of Fujian Province. *Chinese Jourl of Parasitology and Parasitic Diseases* 2017; **35**(1): 5.
1688. Wei Yicheng JL, Ye Julian, Ying Kaiman, Zheng Bofu. Molecular epidemiological investigation of babesi protozoa in rodents in Dapan mountains, Zhejiang Province. *Chinese Jourl of Preventive Medicine* 2013; (12): 4.
1689. Jiang Liping ZL, Bao Qinghan, Lu Qunying, Cheng Suyun, Xu Baoxiang. Babesia DNA segments detected from rodents in Zhejiang province. *Chinese Jourl of Vector Biology and Control* 2012; (04): 33-5.
1690. Chen XR, Ye LI, Fan JW, et al. Detection of Kobe-type and Otsu-type Babesia microti in wild rodents in China's Yunnan province. *Epidemiol Infect* 2017; **145**(13): 2704-10.
1691. Taohua H. Investigation on Babesia infection of host animals in some areas of Yunnan Province: Dali University; 2016.
1692. Xinrong C. Molecular Epidemiological Investigation of Babesia Microti in Some Small Rodents and Captive Monkey Populations in China: Jilin University.
1693. Zeng Z, Zhou S, Xu G, et al. Prevalence and phylogenetic analysis of Babesia parasites in reservoir host species in Fujian province, Southeast China. *Zoonoses Public Health* 2022; **69**(8): 915-24.
1694. Lin H, Zhao S, Ye Y, Shao L, Jiang N, Yang K. A Fluorescent Recombinase Aided Amplification Assay for Detection of Babesia microti. *Korean J Parasitol* 2022; **60**(3): 201-5.
1695. Li M, Yang X, Masoudi A, et al. The regulatory strategy of proteins in the mouse kidney during Babesia microti infection. *Exp Parasitol* 2022; **235**: 108232.
1696. Piao X, Ma Y, Liu S, Hou N, Chen Q. A Novel Thioredoxin-Like Protein of Babesia microti Involved in Parasite Pathogenicity. *Front Cell Infect Microbiol* 2022; **12**: 826818.
1697. Li Y, Rizk MA, Galon EM, et al. Discovering the Potent Inhibitors Against Babesia bovis in vitro and Babesia microti in vivo by Repurposing the Natural Product Compounds. *Front Vet Sci* 2021; **8**: 762107.
1698. Kumar A, O'Bryan J, Krause PJ. The Global Emergence of Human Babesiosis. *Pathogens* 2021; **10**(11).
1699. Gray JS, Ogden NH. Ticks, Human Babesiosis and Climate Change. *Pathogens* 2021; **10**(11).
1700. An X, Yu L, Wang S, et al. Kinetic Characterization and Inhibitor Screening of Pyruvate Kinase I From Babesia microti. *Front Microbiol* 2021; **12**: 710678.
1701. Wei CY, Wang XM, Wang ZS, et al. High prevalence of Babesia microti in small mammals in Beijing. *Infect Dis Poverty* 2020; **9**(1): 155.
1702. Wang F, Jiang JF, Tian J, Du CH. [Clinical characteristics, diagnosis and treatment of human babesiosis: a review]. *Zhongguo Xue Xi Chong Bing Fang Zhi Za Zhi* 2020; **33**(2): 218-24.
1703. Wang X, Wang J, Liu J, et al. Insights into the phylogenetic relationships and drug targets of Babesia isolates infective to small ruminants from the mitochondrial genomes. *Parasit Vectors* 2020; **13**(1): 378.
1704. Wang H, Wang Y, Huang J, et al. Babesia microti Protein BmSP44 Is a Novel Protective Antigen in a Mouse Model of Babesiosis. *Front Immunol* 2020; **11**: 1437.
1705. Li LH, Wang JZ, Zhu D, et al. Detection of novel piroplasmid species and Babesia microti and Theileria orientalis genotypes in hard ticks from Tengchong County, Southwest China. *Parasitol Res* 2020; **119**(4): 1259-69.
1706. Liu M, Ji S, Rizk MA, et al. Transient Transfection of the Zoonotic Parasite Babesia microti. *Pathogens* 2020; **9**(2).
1707. Lin H, Ji YH, Chen XL, et al. [Sero-prevalence of Babesia infection among voluntary blood donors in Jiangsu Province]. *Zhongguo Xue Xi Chong Bing Fang Zhi Za Zhi* 2019; **31**(5): 516-8.
1708. Chen Z, Li H, Gao X, et al. Human Babesiosis in China: a systematic review. *Parasitol Res* 2019; **118**(4): 1103-12.
1709. Guo J, Miao X, He P, et al. Babesia gibsoni endemic to Wuhan, China: mitochondrial genome sequencing, annotation, and comparison with apicomplexan parasites. *Parasitol Res* 2019; **118**(1): 235-

43.
1710. Xu B, Liu XF, Cai YC, et al. Screening for biomarkers reflecting the progression of Babesia microti infection. *Parasit Vectors* 2018; **11**(1): 379.
1711. Huang S, Zhang L, Yao L, et al. Human babesiosis in Southeast China: A case report. *Int J Infect Dis* 2018; **68**: 36-8.
1712. Man S, Fu Y, Guan Y, et al. Evaluation of a Major Surface Antigen of Babesia microti Merozoites as a Vaccine Candidate against Babesia Infection. *Front Microbiol* 2017; **8**: 2545.
1713. Gao ZH, Huang TH, Jiang BG, et al. Wide Distribution and Genetic Diversity of Babesia microti in Small Mammals from Yunnan Province, Southwestern China. *PLoS Negl Trop Dis* 2017; **11**(10): e0005898.
1714. Liu X, Zheng C, Gao X, Chen J, Zheng K. Complete Molecular and Immunoprotective Characterization of Babesia microti Enolase. *Front Microbiol* 2017; **8**: 622.
1715. Zhang H, Sun Y, Jiang H, Huo X. Prevalence of Severe Febrile and Thrombocytopenic Syndrome Virus, Anaplasma spp. and Babesia microti in Hard Ticks (Acari: Ixodidae) from Jiaodong Peninsula, Shandong Province. *Vector Borne Zoonotic Dis* 2017; **17**(2): 134-40.
1716. Wei F, Song M, Liu H, et al. Molecular Detection and Characterization of Zoonotic and Veterinary Pathogens in Ticks from Northeastern China. *Front Microbiol* 2016; **7**: 1913.
1717. Man SQ, Qiao K, Cui J, Feng M, Fu YF, Cheng XJ. A case of human infection with a novel Babesia species in China. *Infect Dis Poverty* 2016; **5**: 28.
1718. Yuan-Yuan L, Heng P, Huai-Min Z, Jian L, Shao-Li X. [Investigation of two blood parasitic protozoa infection in farmed Macaca fascicularis in Guangxi Zhuang Autonomous Region]. *Zhongguo Xue Xi Chong Bing Fang Zhi Za Zhi* 2016; **28**(2): 141-5.
1719. Zhou X, Xia S, Huang JL, Tambo E, Zhuge HX, Zhou XN. Human babesiosis, an emerging tick-borne disease in the People's Republic of China. *Parasit Vectors* 2014; **7**: 509.
1720. Zhou X, Li SG, Wang JZ, et al. Emergence of human babesiosis along the border of China with Myanmar: detection by PCR and confirmation by sequencing. *Emerg Microbes Infect* 2014; **3**(8): e55.
1721. Zhou X, Li SG, Chen SB, et al. Co-infections with Babesia microti and Plasmodium parasites along the China-Myanmar border. *Infect Dis Poverty* 2013; **2**(1): 24.
1722. Lu Y, Cai YC, Chen SH, et al. [Establishment of the experimental animal model of Babesia microti]. *Zhongguo Ji Sheng Chong Xue Yu Ji Sheng Chong Bing Za Zhi* 2012; **30**(6): 423-7.
1723. Yao LN, Ruan W, Zeng CY, et al. [Pathogen identification and clinical diagnosis for one case infected with Babesia]. *Zhongguo Ji Sheng Chong Xue Yu Ji Sheng Chong Bing Za Zhi* 2012; **30**(2): 118-21.
1724. Sun Y, Liu G, Yang L, Xu R, Cao W. Babesia microti-like rodent parasites isolated from Ixodes persulcatus (Acari: Ixodidae) in Heilongjiang Province, China. *Vet Parasitol* 2008; **156**(3-4): 333-9.
1725. Saito-Ito A, Takada N, Ishiguro F, et al. Detection of Kobe-type Babesia microti associated with Japanese human babesiosis in field rodents in central Taiwan and southeastern mainland China. *Parasitology* 2008; **135**(6): 691-9.
1726. Voorberg-vd Wel A, Kocken CH, Zeeman AM, Thomas AW. Detection of new Babesia microti-like parasites in a rhesus monkey (Macaca mulatta) with a suppressed Plasmodium cynomolgi infection. *Am J Trop Med Hyg* 2008; **78**(4): 643-5.
1727. Zamoto A, Tsuji M, Wei Q, et al. Epizootiologic survey for Babesia microti among small wild mammals in northeastern Eurasia and a geographic diversity in the beta-tubulin gene sequences. *J Vet Med Sci* 2004; **66**(7): 785-92.
1728. Shih CM, Liu LP, Chung WC, Ong SJ, Wang CC. Human babesiosis in Taiwan: asymptomatic infection with a Babesia microti-like organism in a Taiwanese woman. *J Clin Microbiol* 1997; **35**(2): 450-4.
1729. Fu W, Hao H, Hu M, Cui YJCJoFH, Quarantine. Babesia Microti-like Rodent Parasites Isolated from Ixodes Persulcatus (Acari:Ixodidae) at China-Russia Ports of Heilongjiang Province.
1730. Piesman JJIJfP. Intensity and duration of Borrelia burgdorferi and Babesia microti infectivity in rodent hosts. 1988; **18**(5): 687-9.
1731. Chao LL, Yu WC, Shih CMJT, Diseases T-b. First detection and molecular identification of Babesia microti in Rattus losea captured from the offshore Kinmen Island of Taiwan. 2017.
1732. Zhensheng, Wang, Xiaomei, et al. Detection of Babesia parasite infection in rodents in Miyun district of Beijing. The 14th National Conference and the 5th International Parasitology Symposium of Parasitology Professional Committee of Zoological Society of China.
1733. Bown KJ, Lambin X, Telford GR, et al. Relative Importance of Ixodes ricinus and Ixodes trianguliceps as Vectors for Anaplasma phagocytophilum and Babesia microti in Field Vole (Microtus agrestis) Populations. 2008; **74**(23): 7118-25.

1734. Beck R, Vojta L, Curkovi S, Mrljak V, Habrun BJVb, diseases z. Molecular Survey of Babesia microti in Wild Rodents in Central Croatia. 2011; **11**(1): 81-3.
1735. Benach JL, White DJ, Mcgovern JPJAJoTM, Hygiene. Babesiosis in Long Island. Host-parasite relationships of rodent- and human-derived Babesia microti isolates in hamsters. 1978; **27**(6): 1073-8.
1736. Okabayashi T, Hagiya J, Tsuji M, Ishihara C, Satoh H, Morita CJJoVMS. Detection of Babesia microti-like parasite in filter paper-absorbed blood of wild rodents. 2002; **64**(2): 145.
1737. Walter GJAT. [Transmission and course of parasitemia of Babesia microti (Hannover I strain) in the bank vole (Clethrionomys glareolus) and field vole (Microtus agrestis)]. 1984; **41**(3): 259-64.
1738. To?Kacz K, Bednarska M, Alsarraf M, et al. Prevalence, genetic identity and vertical transmission of Babesia microti in three naturally infected species of vole, Microtus spp. (Cricetidae). 2017; **10**(1): 66.
1739. Chao LL, Yu WC, Shih CMJT, Diseases T-b. First detection and molecular identification of Babesia microti in Rattus losea captured from the offshore Kinmen Island of Taiwan. 2016; **8**(2): 313.
1740. Tadin A, Tokarz R, Markoti A, Margaleti J, Lipkin WIJTAjotm, hygiene. Molecular Survey of Zoonotic Agents in Rodents and Other Small Mammals in Croatia. 2015; **94**(2).
1741. Huang Zhengmei BW. Investigation on the infection of parasites in rats in Yuxi area. *Medical animal control* 1995; **011**(002): 151-3.
1742. Zhou Xiaomei ZG, Li Cong, Li Fuhua, Yin Zhiheng, Yang Jialin, Su Ping. Investigation on mice infected with Trichinella hepatica in Kunming. *Chin J Zoonoses* 1998; **14**(5): 2.
1743. Yuan Gaolin LX, Chen Wenjin. Investigation on Parasite Infection in the Liver of Field Mouse in Ningde City. *Chinese Jourl of Vector Biology and Control* 2000; **11**(004): 301-2.
1744. Yang Yuanqing ZC, Wu Jiatong. Rediscovery of Capillaria hepatica in the liver of Gourd rat in Shanghai. *Chinese Jourl of Parasitic Disease Control* 1989; (2): 146-.
1745. Wenzhi W. Investigation and alysis on the infection of Trichinella hepatica and Trichinella spiralis in rats in Anyang City, Hen Province. *Chinese Jourl of Vector Biology and Control* 2010; (002): 021.
1746. Wang Pengpeng HJ. Discovery of Trichinella hepatica in mice. *Zhejiang Animal Husbandry Veteriry* 1987; (2).
1747. Changxiu N. Investigation on the Infection of Trichinella hepatica by Rodents in Some Areas of Hubei Province. *Chin J Zoonoses* 1989; **5**(01): 53-4.
1748. Li Feng CS. Investigation on rodent population distribution and parasites in Funiu Mountains of Nanyang city. *Chinese sanitary insecticide* 2015; (5): 3.
1749. Yongfang D. Capillaria hepatica found in the liver of rats in Shangyu County. *Endemic Diseases Bulletin* 1993; (1).
1750. Hong Lingxian YW, Lin Yuguang, Peng Wenfeng. Investigation on Rodent Protozoa in Xiamen Suburb and Tacheng, Xinjiang. *Wuyi Science* 1994: 5.
1751. Lin Ximeng XB, Zhao Xudong, Li Hui, Huang Qian, Deng Yan, Hao Zongyu, Zhang Aimei. Epidemiological investigation of liver trichinellosis in small animal hosts in Henan Province. *Chinese Jourl of Pathogenic Biology* 2007; **002**(001): 44-6.
1752. Tingzhu W. Investigation on the distribution of rodent population and its parasites in Danjiang reservoir area: Zhengzhou University; 2010.
1753. Xiaomei Z, Guifang Z, Cong L, et al. Investigation on mice infected with Trichinella hepatica in Kunming. *Chinese Journal of Zoonoses* 1998; **14**(5): 2.
1754. Hongbo L, Changwang P, Weiping Y, et al. Epidemiology and Biology of Trichinella hepatica in Wenzhou. *Journal of Wenzhou Medical University* 2000; **30**(001): 13-5.
1755. Lijie S, Zhiyong L, Wei L, et al. Investigation on Infection of Trichinella hepatica in Rodents in Dali Area. *Chinese Journal of Pathogenic Biology* 2003; (5).
1756. Xinrong W, Yu J, Tianbiao Z, et al. The relationship between the natural foci of liver trichinellosis and the distribution of rodents in Xilingol area. *Journal of Zoology* 2007; **42**(1): 14-9.
1757. Huang Zhengmei BW. Investigation on Hepatic Capillaria Infection of Rodents in Yuxi Area, Yunnan Province. *Journal of Practical Parasitic Diseases* 1995.
1758. Xiaomei Z, Guifang Z, Cong L, et al. Investigation on mice infected with Trichinella hepatica in Kunming. *Chin J Zoonoses* 1998; **14**(5): 2.
1759. Mengtao X, Guangrong Y, Xing W, et al. Investigation on small mammals infected with Capillaria hepatica in Yongjian Township, Weishan County. *Endemic Diseases Bulletin* 1999; **14**(003): 58-60.
1760. Fuhua L, Yanzhong L, Hong T, Xiaomei Z. Investigation on Trichinella hepatica of rodents in Yunnan. *Public Health in China* 1992; **8**(1).
1761. Yanmei G, Junjie H, Yanfen Y, Yan Y, Weiwei Z, Benjiang Z. Investigation on the Infection of

Trichinella hepatica by Rodents in Anning City, Yunnan Province and Study on the Host of Experimental Animals. *Chinese Journal of Parasitology and Parasitic Diseases* 2013; **31**(5): 5.
1762. Yanrong L, Zuhua L, Yingjiao L, Aiqin L, column Yh, Jinxiang L. Investigation on Hepatic Capillaria Infection of Rodents in Fujian Province. *Chinese Journal of Parasitic Disease Control* 1993.
1763. Changxiu N. Investigation on the Infection of Trichinella hepatica by Rodents in Some Areas of Hubei Province. *Chinese Journal of Zoonoses* 1989; **5**(01): 53-4.
1764. Zhengmei H. Investigation on Hepatic Capillaria Infection of Rodents in Yuxi Area, Yunnan Province. *Parasitic Diseases and infectious diseases* 1995; (3).
1765. Jiaxu W, Chenghao S, Jianwei H, Wei C, Licong L, Yanfeng Z. Investigation on Capillaria hepatica naturally infected by rodents in Xiamen. *Journal of Strait Preventive Medicine* 2014; (6): 1.
1766. Gen C, Book B, Jian H, Tao J. Investigation on infection of Capillaria hepatica in plateau pika in Maqu County, Gansu Province. *Chinese Journal of Pathogenic Biology* 2011; **6**(3): 3.
1767. Yushun X, Chenghui W, Minsheng H, Ronghuang L. Investigation on Fuqing rodents infected with Trichinella hepatica. *Journal of Strait Preventive Medicine* 1998; **4**(4): 2.
1768. Tingzhu W, Feng L, Shilei C. Investigation on liver capillaries in rats in Danjiang reservoir area. *Medical animal control* 2013; (5): 3.
1769. Benfu L, Xiang X, Fangwei W, et al. Investigation on Infection of Trichinella hepatica in Rodents in Honghe Valley, Yunnan Province. *Chinese Tropical Medicine* 2019; **19**(2): 3.
1770. Wang C, He H, Li M, et al. Parasite species associated with wild plateau pika (Ochotona curzoniae) in southeastern Qinghai Province, China. *J Wildl Dis* 2009; **45**(2): 288-94.
1771. Chai Y, Deng L, Liu H, et al. First detection of Cryptosporidium spp. in red-bellied tree squirrels (Callosciurus erythraeus) in China. *Parasite* 2019; **26**: 28.
1772. Zhao W, Wang J, Ren G, et al. Molecular characterizations of Cryptosporidium spp. and Enterocytozoon bieneusi in brown rats (Rattus norvegicus) from Heilongjiang Province, China. *Parasit Vectors* 2018; **11**(1): 313.
1773. Youshu D. A Report on the Parasitism of Schistosoma mansoni in the Liver of Rattus norvegicus. *Chinese Jourl of Parasitic Disease Control* 2001; **14**(3): 1.
1774. Deng L, Li W, Yu X, et al. First Report of the Human-Pathogenic Enterocytozoon bieneusi from Red-Bellied Tree Squirrels (Callosciurus erythraeus) in Sichuan, China. *PLoS One* 2016; **11**(9): e0163605.
1775. Zhao W, Zhou H, Yang L, et al. Prevalence, genetic diversity and implications for public health of Enterocytozoon bieneusi in various rodents from Hainan Province, China. *Parasit Vectors* 2020; **13**(1): 438.
1776. Shu Qun ZZ, Zhou Qiantao, Zhang Luyu. Interl parasites of Rattus norvegicus and their relationship with human population. *Chin J Zoonoses* 1993; **9**(02): 41-.
1777. Wei Deqiong YY, Tu Yufa. Isolation of Toxoplasma gondii from mice. *Chin J Zoonoses* 1991; (01): 52-3.
1778. Guobi M. Detection of Toxoplasma gondii antibody in Tibetan mastiff and grassland in some areas of Qinghai Province by indirect hemagglutition test. *Advances in Animal Medicine* 2012; **33**(7): 3.
1779. Chaochao L. Investigation on Intestinal Parasitic Infection of Some Rodents and Analysis of Genetic Characteristics of Cryptosporidium: Henan Agricultural University; 2009.
1780. Xiaoxiao Z. Isolation, identification and phylogenetic analysis of Cryptosporidium from camel and bamboo rat: Sichuan Agricultural University; 2014.
1781. Wu Jun YJ, Yin Weixiong. Inspection of parasitic infection in wild white bellied giant rats. *Preventive Medicine in South China* 2008; **24**(1): 2.
1782. Shen Lijie LW, Luo Zhiyong. Investigation on the Infection of Common Parasitic Worms in Rat like Animals in Dali City. *Jourl of Dali University* 2004; (05): 12-3.
1783. Li Wei SL, Luo Zhiyong, Qian Tijun. Investigation on Hymenolepis Infection of Small Mammals in Dali City. *Jourl of Dali University* 2003; **2**(1): 3.
1784. Yang D, Zhao W, Zhang Y, Liu A. Prevalence of Hymenolepis nana and H. diminuta from Brown Rats (Rattus norvegicus) in Heilongjiang Province, China. *Korean J Parasitol* 2017; **55**(3): 351-5.
1785. Wu Jun YJ, Duan Jinhua, Yin Weixiong, Zhang Shiyan, Liang Lian. Investigation on Hymenolepis Taenia Infection in Rattus norvegicus and Rattus flavipectus in Zhanjiang City. *Chinese Jourl of Parasitic Disease Control* 2004; **17**(005): 306-7.
1786. Shao Yiyang SY, Ye Jianping. Investigation on the Infection of Hymenolepis in Rodents in Shaoxing City. *Jourl of Shaoxing University of Arts and Sciences (tural Science)* 2016; **36**(9): 103-6.
1787. Ning Changxiu NT. Investigation on Hymenolepis in Mice in Some Areas of Hubei Province. *Sichuan Animals* 1989; **8**(4): 1.
1788. Wu Jun YJ, Yin Weixiong, Duan Jinhua, Lin Lifeng. Investigation on parasites in rodents in

four cities of the pearl river delta. *Jourl of Tropical Medicine*; (6): 704-6.
1789. Weiping Y, Dexu Z, Xujuling, et al. Investigation and treatment of 11 cases of human infection caused by Hymenolepis diminuta. *Chinese Journal of Zoonoses* 1992; **8**(05): 41-2.
1790. Gonshasa, Qingling M, Jun Q, Xingxing Z, Shuaiqi S. Investigation on parasitic infection in digestive system of experimental mice in Shihezi area. Proceedings of the 13th Academic Seminar of the Veterinary Parasitology Branch of the Chinese Society of Animal Husbandry and Veterinary Medicine; 2015; 2015.
1791. Jun W, Jianrong Y, Jinhua D, Weixiong Y, Shiyan Z, Lian L. Investigation on Hymenolepis Taenia Infection in Rattus norvegicus and Rattus flavipectus in Zhanjiang City. *Chinese Journal of Pathogenic Biology* 2004; **17**(005): 306-7.
1792. Lili Z, Yu C, Xiaodong D, et al. Infection and Epidemiological Analysis of Hymenolepis nana in Dalian. *Chinese Journal of Microecology* 2005; **17**(2): 2.
1793. Sufang L. Detection of Hymenolepis brevis from Golden Hamster. *Laboratory animal and comparative medicine* 1986; (02): 49.
1794. Shen LJ, Wei LI, Qian TJ, Luo ZY. Investigation on Hymenolepis nana Infection in Wild Small Mammals in Xiaguan, Dali, Yunnan. 2002.
1795. Zhou Bo JJ. Domint species of intestil parasites of Rattus rubicus in Northeast China. *Jourl of Northeast Forestry University* 2010; **38**(2): 2.
1796. Wu Jun YJ, Yin Weixiong, Duan Jinhua, Lin Lifeng. Investigation on parasites in rodents in four cities of the pearl river delta. *Jourl of Tropical Medicine* 2004; (6): 704-6.
1797. Qiao Haisheng CQ, Ma Liqing. Serological detection of toxoplasmosis in Marmota himalaya. *Jourl of Livestock Ecology* 2014; **35**(5): 2.
1798. Zhang XX, Huang SY, Zhang YG, Zhang Y, Zhu XQ, Liu Q. First report of genotyping of Toxoplasma gondii in free-living Microtus fortis in northeastern China. *J Parasitol* 2014; **100**(5): 692-4.
1799. Cai Qigang XT, Ma Liqing. Detection of Toxoplasmosis in Marmota himalaya in Qinghai by ELISA. *Chi Animal Quarantine* 2013.
1800. Yang Yuanqing GL, Wu Jiatong, Zhang Chaowei, Chang Zhengshan. The discovery of amoeba cyst like parasites in the spleen of wild rats in Tai'an. *Chinese veteriry parasitic diseases* 1995; (3): 59-60.
1801. Yang Guangrong WX, Xiong Mengtao, Fan Chongzheng, Wu Hesong, Tao held the meeting. Investigation of Schistosoma japonicum infection in rodents in Yunn Province. *Chinese Jourl of Parasitology and Parasitic Diseases* 2000; **18**(4): 4.
1802. Guangrong Y, Mengtao X, Xing W, et al. Analysis of natural infection of schistosoma japonicum in rats. *Chinese Journal of Vector Biology and Control* 2000; **11**(1): 5.
1803. Xiong Mengtao YG, Wu Xing, Fan Chongzheng, Tao Huihui, Wu Hesong, Su Luquan, Duan Caihong. Investigation of Schistosoma japonicum infection in rodents in plateau canyon area. *Endemic Diseases Bulletin* 1999; **14**(4): 3.
1804. Zhou Peisheng KC, Chen Long, Li Guoxiang, Huang Jialiang. Investigation on two species of mice turally infected with Schistosoma japonicum in Dongting Lake area. *Chinese Jourl of Vector Biology and Control* 1996; **7**(2): 1.
1805. Liu J, Zhu C, Shi Y, et al. Surveillance of Schistosoma japonicum infection in domestic ruminants in the Dongting Lake region, Hunan province, China. *PLoS One* 2012; **7**(2): e31876.
1806. Li FY, Hou XY, Tan HZ, et al. Current Status of Schistosomiasis Control and Prospects for Elimination in the Dongting Lake Region of the People's Republic of China. *Front Immunol* 2020; **11**: 574136.
1807. Guoyu X, Jichun T, Guangmei C, et al. Study on the foci of schistosomiasis japonica in njing. *Scientific and technological achievements* 1999; **7**(1): 3.
1808. Wang X, Ruan L, Song Q, et al. First report of Schistosoma sinensium infecting Tupaia belangeri and Tricula sp. LF. *Int J Parasitol Parasites Wildl* 2021; **14**: 84-90.
1809. Guo Zaixian KX, Su Minghua, Zhao Guoqiang. Studies on the tural foci of hydatid disease in wild fau i - confirmation of plateau pika as intermediate host of hydatid disease. *Qinghai Medical Jourl* 1987; (1).
1810. Wang X, Liu J, Zuo Q, et al. Echinococcus multilocularis and Echinococcus shiquicus in a small mammal community on the eastern Tibetan Plateau: host species composition, molecular prevalence, and epidemiological implications. *Parasit Vectors* 2018; **11**(1): 302.
1811. Wu C, Zhang W, Ran B, et al. Genetic variation of mitochondrial genes among Echinococcus multilocularis isolates collected in western China. *Parasit Vectors* 2017; **10**(1): 265.
1812. Zhang X, Jian Y, Li X, Ma L, Karanis G, Karanis P. The first report of Cryptosporidium spp. in Microtus fuscus (Qinghai vole) and Ochotona curzoniae (wild plateau pika) in the Qinghai-Tibetan Plateau area, China. *Parasitol Res* 2018; **117**(5): 1401-7.

1813. Cao YF, Ye RR, Wu JH, Bian JH, Duszynski DW. Eimeria spp. (Apicomplexa: Eimeriidae) from the Plateau pika, Ochotona curzoniae, from Haibei Area, Qinghai Province, China, with the description of two new species. *J Parasitol* 2009; **95**(5): 1192-6.
1814. Fan YL, Lou ZZ, Li L, et al. Genetic diversity in Echinococcus shiquicus from the plateau pika (Ochotona curzoniae) in Darlag County, Qinghai, China. *Infect Genet Evol* 2016; **45**: 408-14.
1815. He W, Ke L, Guo X, et al. A survey on parasites in wild rodents in Xiji County, a northwestern part of China. *Trop Biomed* 2017; **34**(2): 449-52.
1816. Shen Lijie LZ, Li Wei. Seroepidemiological investigation of Trichinella spiralis infection in rodents in Dali Prefecture, Yunnan Province. *Chinese Jourl of Epidemiology* 2004; **25**(3): 1.
1817. Wang ZQ, Cui J, Shen LJJVJ. The epidemiology of animal trichinellosis in China. 2007; **173**(2): 391-8.
1818. Laya ZS, Li Wen. First Discovery of Taenia solium in Qinghai Mice. *Chinese Jourl of Rodent Control* 1988; (1).
1819. Jiahan L. Parasitic relationship between brown back and intestinal parasites in Northeast China. *Northeast Forestry University* 2011.
1820. Yuan D, Qin H, Zhang J, et al. Phylogenetic analysis of HSP70 and cyt b gene sequences for Chinese Leishmania isolates and ultrastructural characteristics of Chinese Leishmania sp. *Parasitol Res* 2017; **116**(2): 693-702.
1821. Guan LR, Yang YQ, Qu JQ, Shen WX. Discovery and study of Leishmania turanica for the first time in China. *Bull World Health Organ* 1995; **73**(5): 667-72.
1822. Niang Jixian YQ, n Xukong, Caidan. Investigation on hydatid disease of wild animals in Huangn area. *Qinghai Jourl of Animal Husbandry and Veteriry Medicine* 1986; (1).
1823. Yang Hongmo LZ. First Discovery of Capillaria hepatica from Mice in Yunn. *Jourl of Parasitology and Parasitic Diseases* 1991; (S1): 1.
1824. Wang Xiangsheng ZW, Xie Chao, Yang Faqing, Liu Xiaofang, Sun Engui, Zhou Guojun, Zhang Xueli, Sun Lilan. Investigation and Morphological Observation of Trypanosoma lugens in Five Provinces (Regions) of Chi. *Chin J Zoonoses* 1998; **14**(2): 36-8.
1825. Kuang Xijin LC, Sun Xiaokang, Lu Wentao, Huang Weifang, Tan Shao'an. Investigation on the Infection of Trypanosoma Lucidum in Rats in Some Areas of Qingyuan City. *Jourl of Tropical Medicine* 2012; **12**(002): 229-30.
1826. Mo Xiuling ZH, Jiang He, Tan Yuguang, Shi Wei, Huang Weiyi. Investigation and Morphological Observation on tural Infection of Trypanosoma lugens in Rattus norvegicus in Some Areas of Guangxi. *Jourl of Tropical Medicine* 2008; **8**(006): 621-2.
1827. Sun Xiaokang JJ, Deng Yuanling, Chen Bin, Zeng Xixi, Tang Jiahui. Investigation on the Infection of Trypanosoma Lucidum in Population and Host Animals in Qingyuan District, Guangdong Province. *Preventive Medicine in South China* 2013; (1): 3.
