# Supplementary material for: Small mammals and associated infections in China: a systematic review and spatial modelling analysis
Source: Lancet Reg Health West Pac. 2024 Dec 18;54:101264. doi: 10.1016/j.lanwpc.2024.101264 (PMC11728903; doi:10.1016/j.lanwpc.2024.101264)

Supplementary appendix 2

Supplement to: Small mammals and associated infections in China: a systematic review and spatial modelling analysis

Table of Contents 2

Appendix Figure S1: The spatial distribution of the small mammal family Cricetidae and Muridae at the county level from 1950 to 2021 in China. 2
Appendix Figure S2: The spatial distribution of the small mammal family Dipodidae, Ochotonidae, Pteromyidae, and Sciuridae at the county level from 1950 to 2021 in China. 3
Appendix Figure S3: The spatial distribution of the small mammal family Soricidae, Spalacidae, Talpidae, and other small mammal families at the county level from 1950 to 2021 in China. 4
Appendix Figure S4: Small mammal species richness at the prefecture and province level in mainland China from 1950 to 2021. 5
Appendix Figure S5: The BRT-predicted county-level average probabilities of presence across 100 bootstrap samples for 12 small mammal species. 6
Appendix Figure S6: The BRT-predicted county-level average probabilities of presence across 100 bootstrap samples for 12 small mammal species. 7
Appendix Figure S7: The BRT-predicted county-level average probabilities of presence across 100 bootstrap samples for 12 small mammal species. 8
Appendix Figure S8: The BRT-predicted county-level average probabilities of presence across 100 bootstrap samples for nine small mammal species. 9
Appendix Figure S9: The mean curves (red) and 95% percentiles (gray) across 100 bootstrap samples for the effects of top six major predictors on the county-specific probability of presence for 16 small mammal species based on the BRT models. 10
Appendix Figure S10: The mean curves (red) and 95% percentiles (gray) across 100 bootstrap samples for the effects of top six major predictors on the county-level probability of presence for 16 small mammal species based on the BRT models. 11
Appendix Figure S11: The mean curves (red) and 95% percentiles (gray) across 100 bootstrap samples for the effects of top six major predictors on the county-level probability of presence for 13 small mammal species based on the BRT models. 12

Appendix Figure S1: The spatial distribution of the small mammal family Cricetidae and Muridae at the county level from 1950 to 2021 in China.

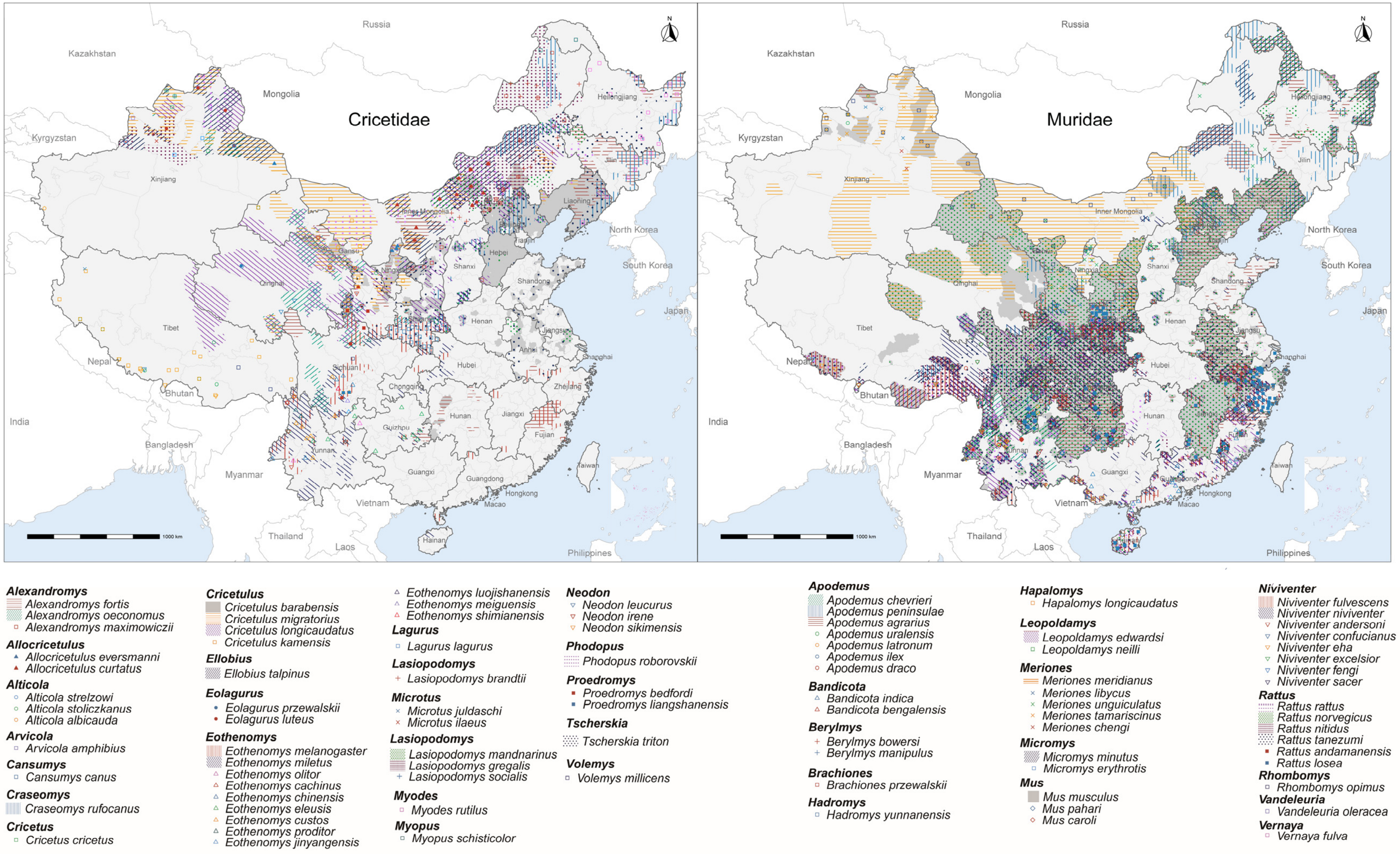

Appendix Figure S2: The spatial distribution of the small mammal family Dipodidae, Ochotonidae, Pteromyidae, and Sciuridae at the county level from 1950 to 2021 in China.

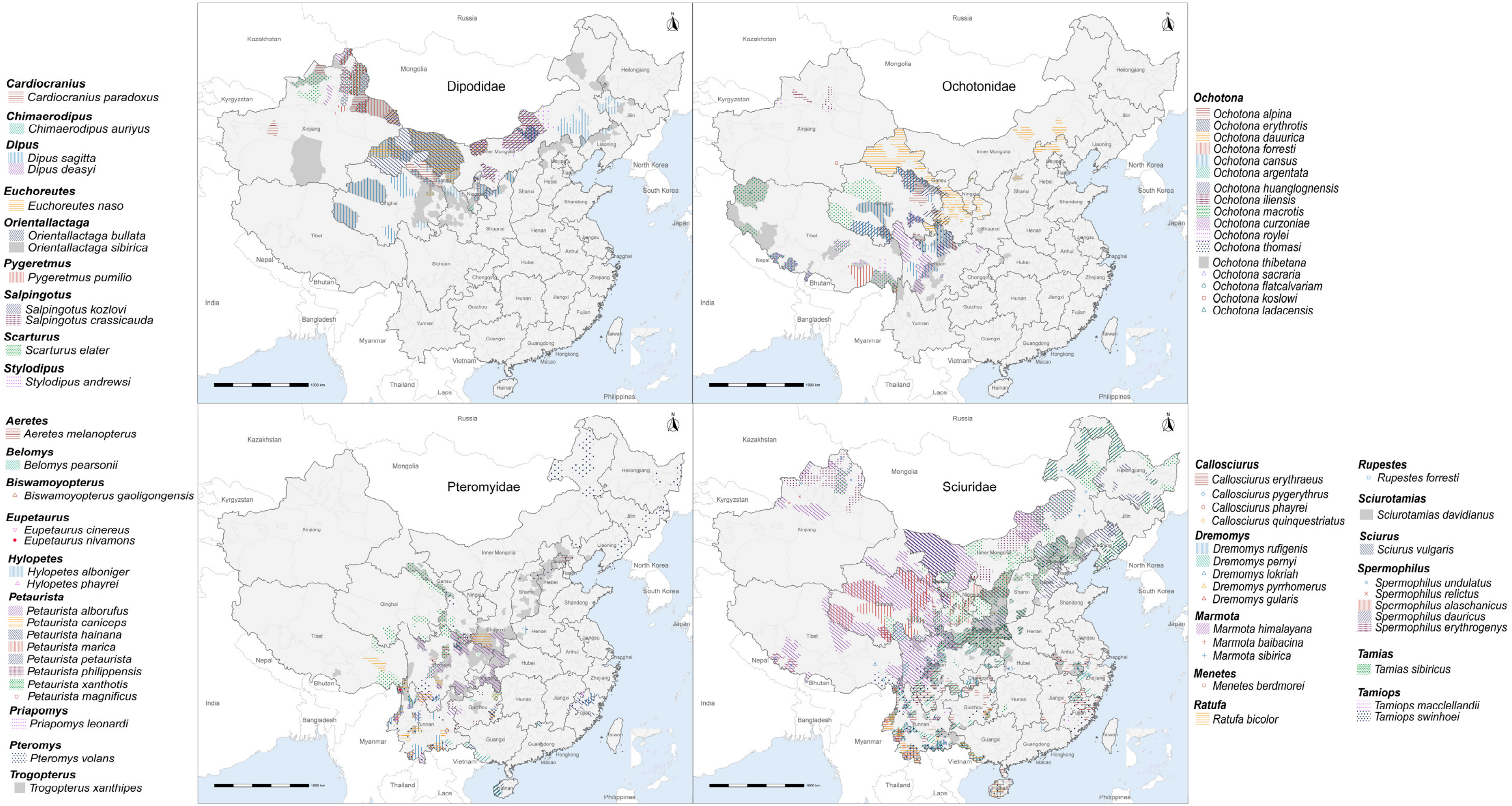

Appendix Figure S3: The spatial distribution of the small mammal family Soricidae, Spalacidae, Talpidae, and other small mammal families at the county level from 1950 to 2021 in China.

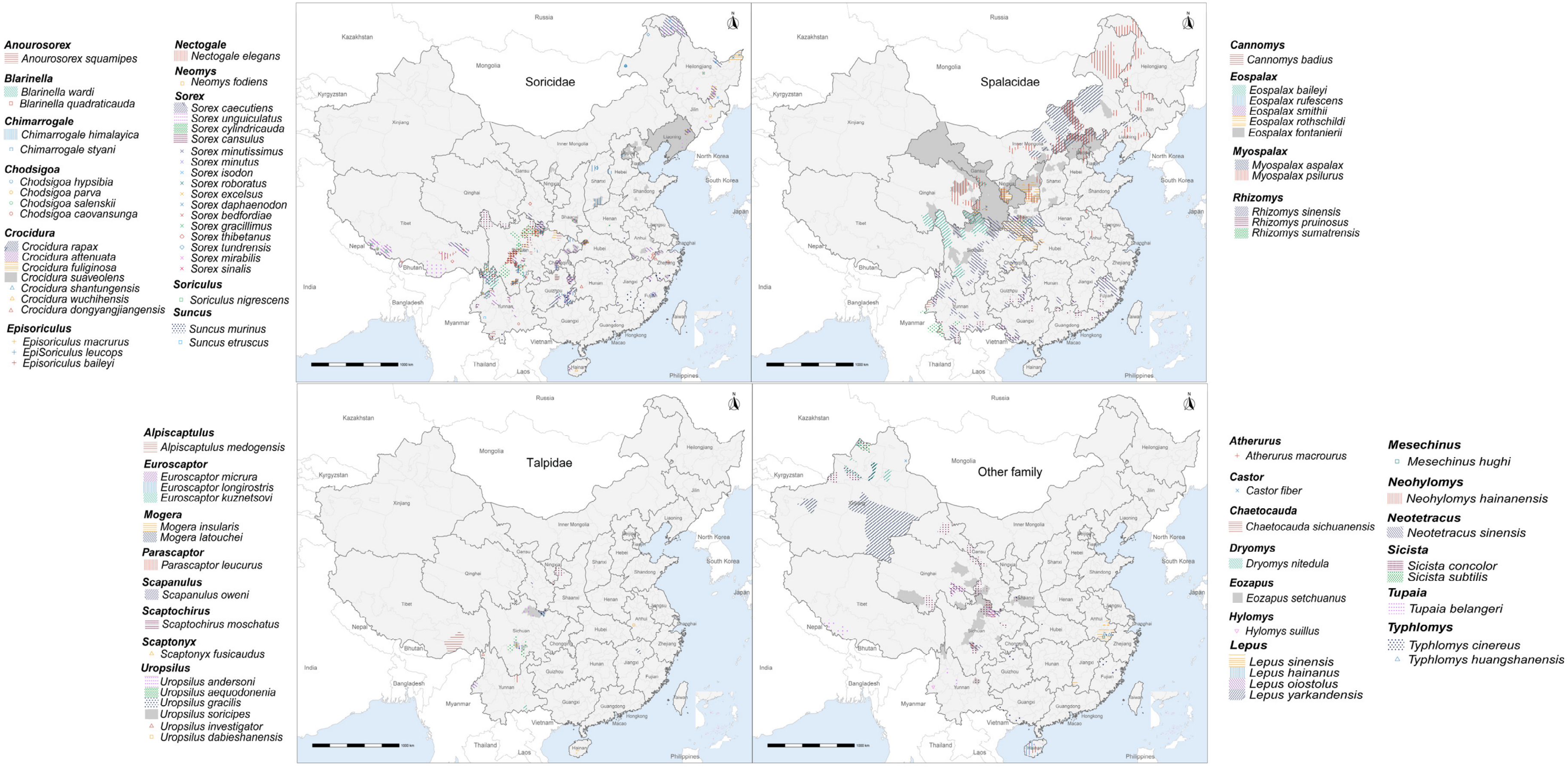

Appendix Figure S4: Small mammal species richness at the prefecture and province level in mainland China from 1950 to 2021. (A) Small mammal species richness (circles) in seven biogeographic zones in mainland of China from 1950 to 2021, I = Northeast district, monsoon climate and mostly covered by montane forest and woodland grassland with some areas of plains; II = North China district, temperature monsoon climate and mostly covered by plain with some areas of mountains and hills; III = Inner Mongolia-Xinjiang district, arid and semi-arid climate and mostly covered by deserts, grasslands and mountains; IV = Qinghai-Tibet district, alpine plateau climate and mostly covered by alpine meadows, alpine grasslands and alpine deserts; V = Southwest district, subtropical monsoon climate and plateau mountain climate, and mostly covered by highland forest steppe, meadow steppe, and forests, shrubs, grasslands and farmland; VI = Central China district, temperate monsoon climate and subtropical monsoon climate, and mostly covered by forests, shrubs, and farmland; VII = South China district, tropical and subtropical climate, and mostly covered by forests, shrubs, grasslands and farmland; (B) Small mammal species richness (pies) at the province level in small mammal families in mainland China from 1950 to 2021.

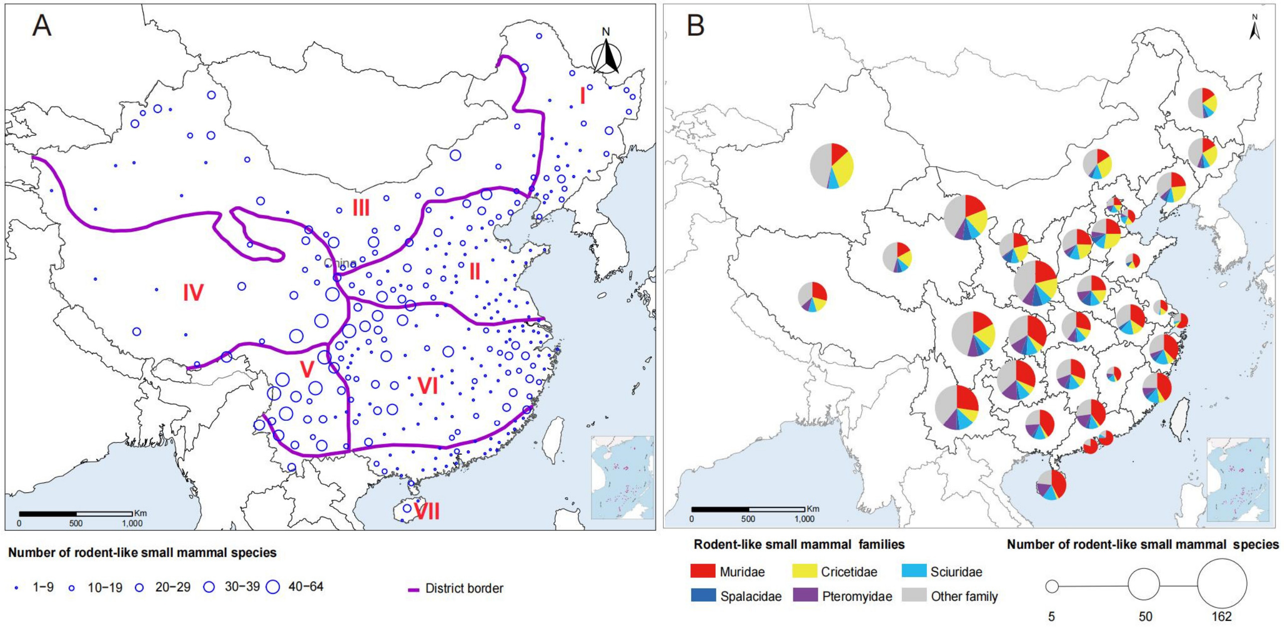

Appendix Figure S5: The BRT-predicted county-level average probabilities of presence across 100 bootstrap samples for 12 small mammal species. (A) *Mus musculus*; (B) *Rattus norvegicus*; (C) *Apodemus agrarius*; (D) *Rattus tanezumi*; (E) *Niviventer niviventer*; (F) *Cricetulus barabensis*; (G) *Rattus nitidus*; (H) *Sciurotamias davidianus*; (I) *Tamias sibiricus*; (J) *Tscherskia triton*; (K) *Spermophilus dauricus*; (L) *Apodemus peninsulae*.

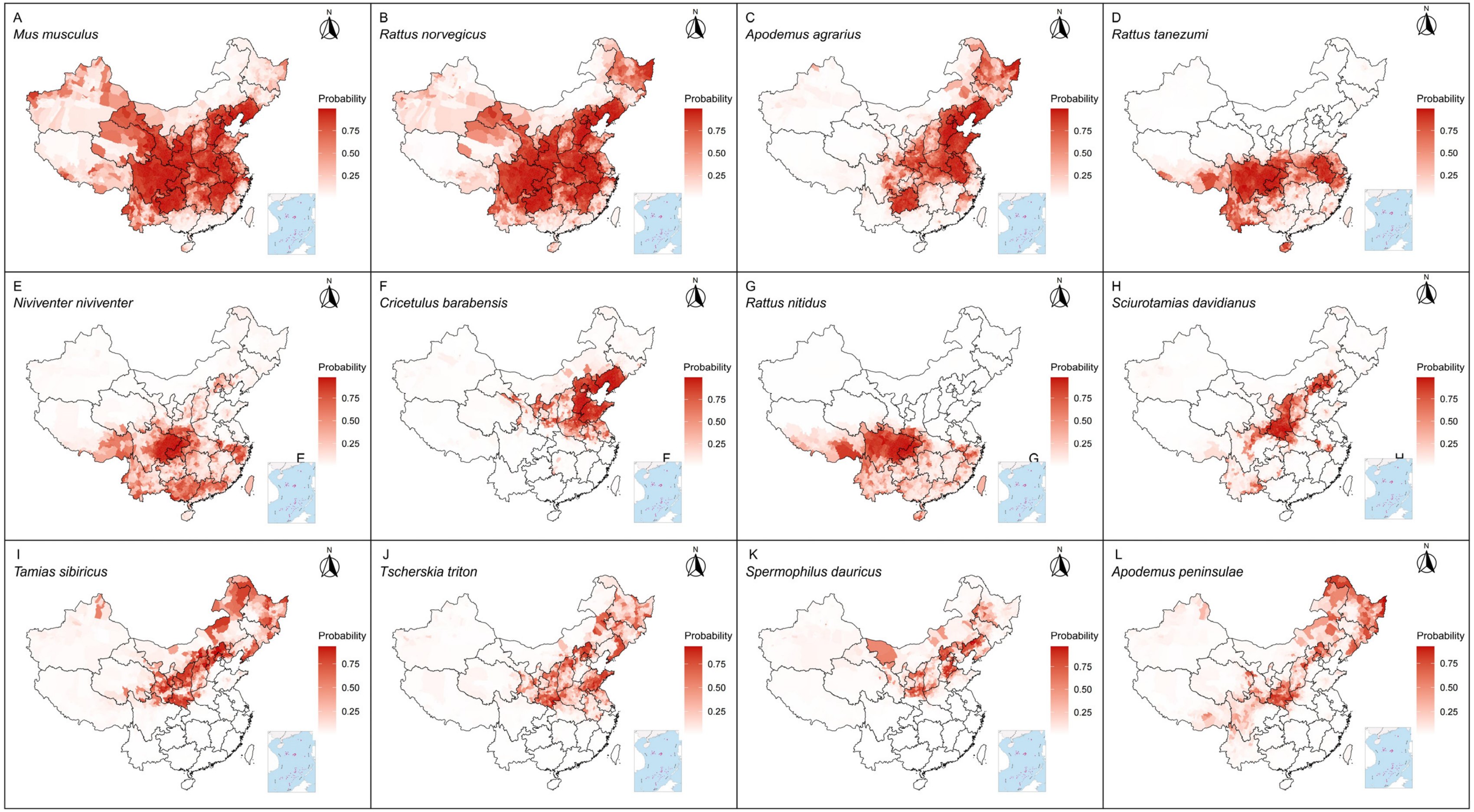

Appendix Figure S6: The BRT-predicted county-level average probabilities of presence across 100 bootstrap samples for 12 small mammal species. (A) *Callosciurus erythraeus*; (B) *Eospalax fontanierii*; (C) *Niviventer fulvescens*; (D) *Leopoldamys edwardsi*; (E) *Tamiops swinhoei*; (F) *Trogopterus xanthipes*; (G) *Micromys minutus*; (H) *Dremomys pernyi*; (I) *Rhizomys sinensis*; (J) *Crocidura suaveolens*; (K) *Rattus rattus*; (L) *Cricetulus longicaudatus*.

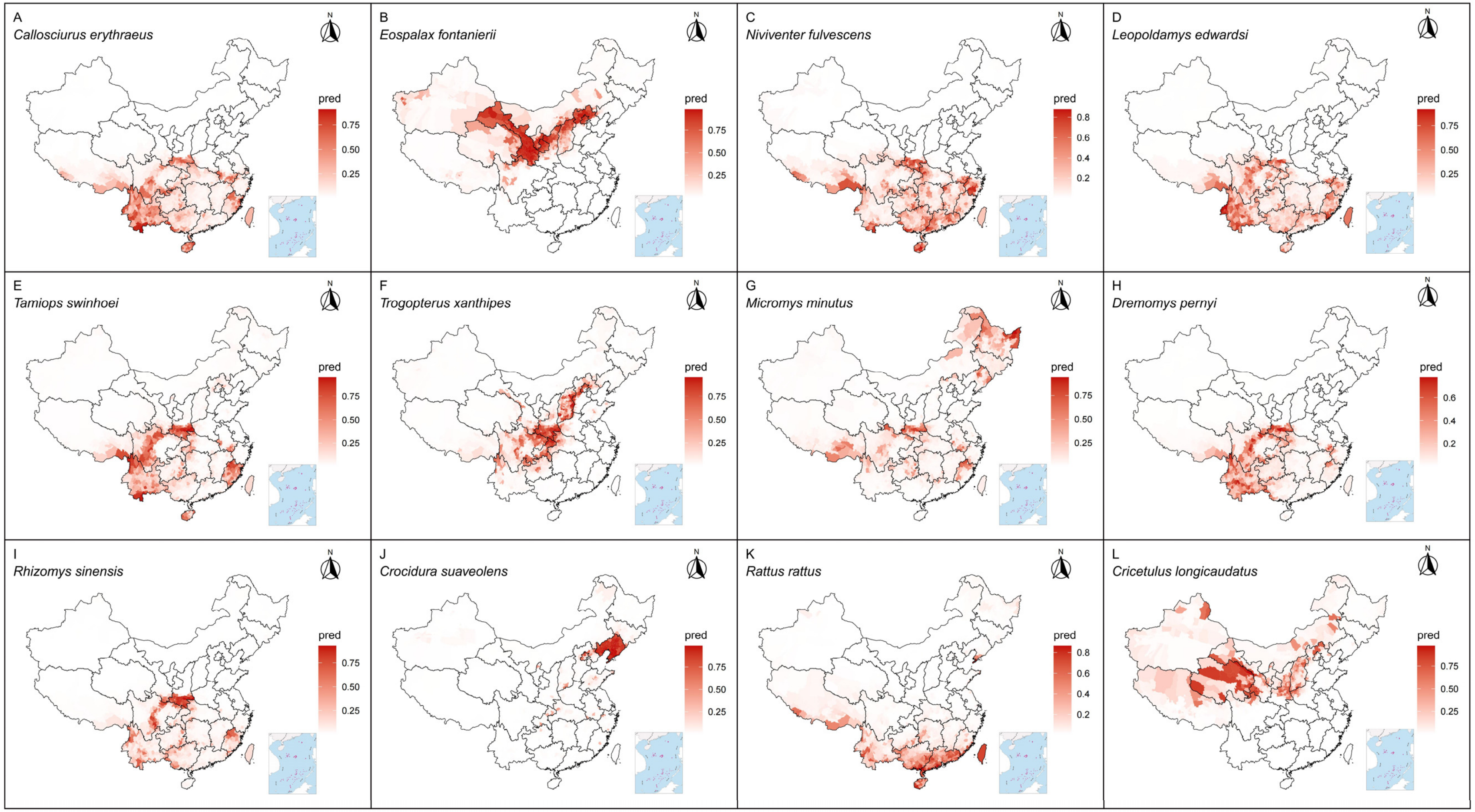

Appendix Figure S7: The BRT-predicted county-level average probabilities of presence across 100 bootstrap samples for 12 small mammal species. (A) *Apodemus chevrieri*; (B) *Meriones meridianus*; (C) *Marmota himalayana*; (D) *Alexandromys fortis*; (E) *Rattus losea*; (F) *Orientallactaga sibirica*; (G) *Eothenomys melanogaster*; (H) *Apodemus draco*; (I) *Pteromys volans*; (J) *Craseomys rufocanus*; (K) *Petaurista alborufus*; (L) *Eothenomys miletus*.

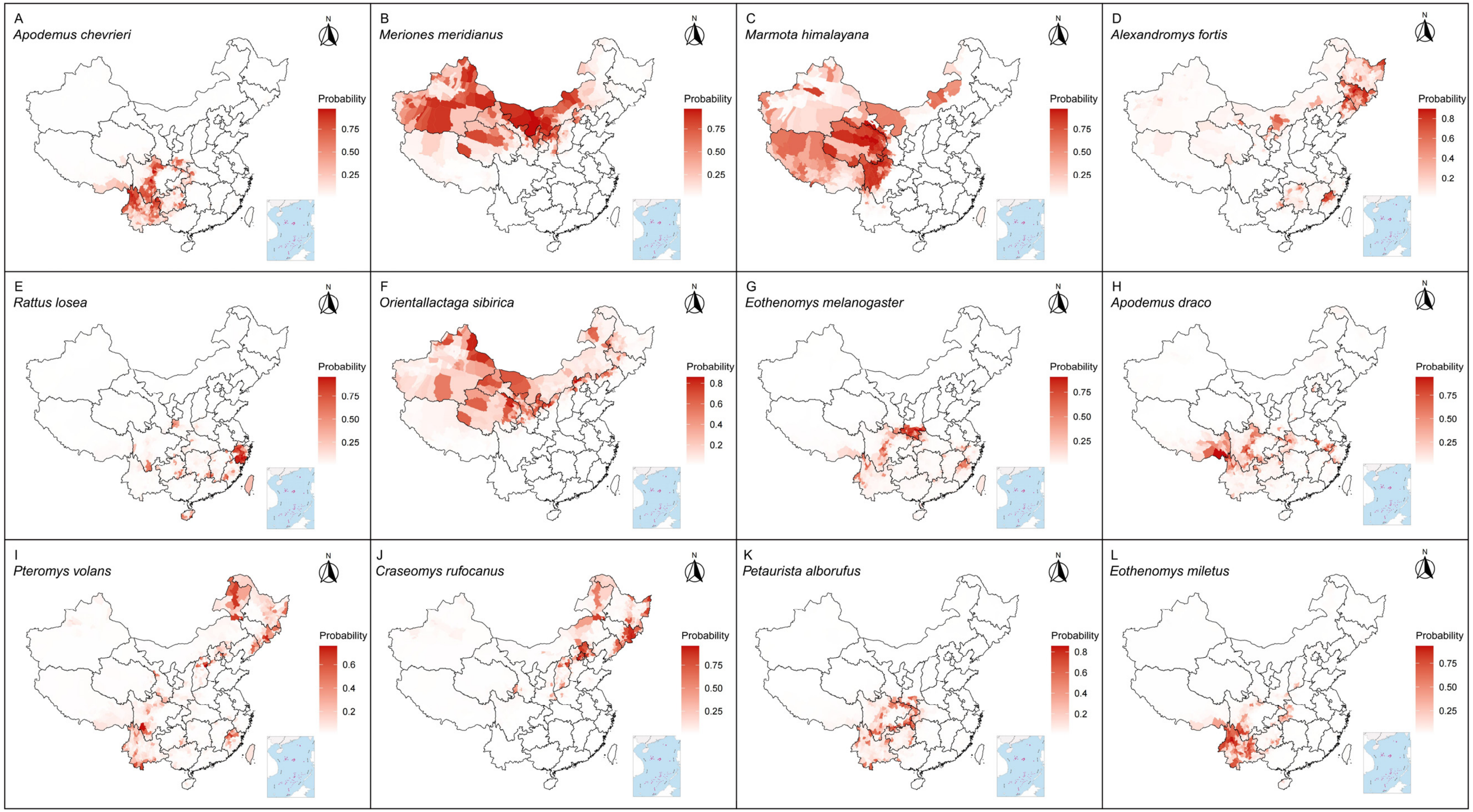

Appendix Figure S8: The BRT-predicted county-level average probabilities of presence across 100 bootstrap samples for nine small mammal species. (A) *Myospalax psilurus*; (B) *Berylmys bowersi*; (C) *Bandicota indica*; (D) *Niviventer confucianus*; (E) *Ochotona thibetana*; (F) *Cricetulus migratorius*; (G) *Anourosorex squamipes*; (H) *Meriones unguiculatus*; (I) *Ochotona dauurica*.

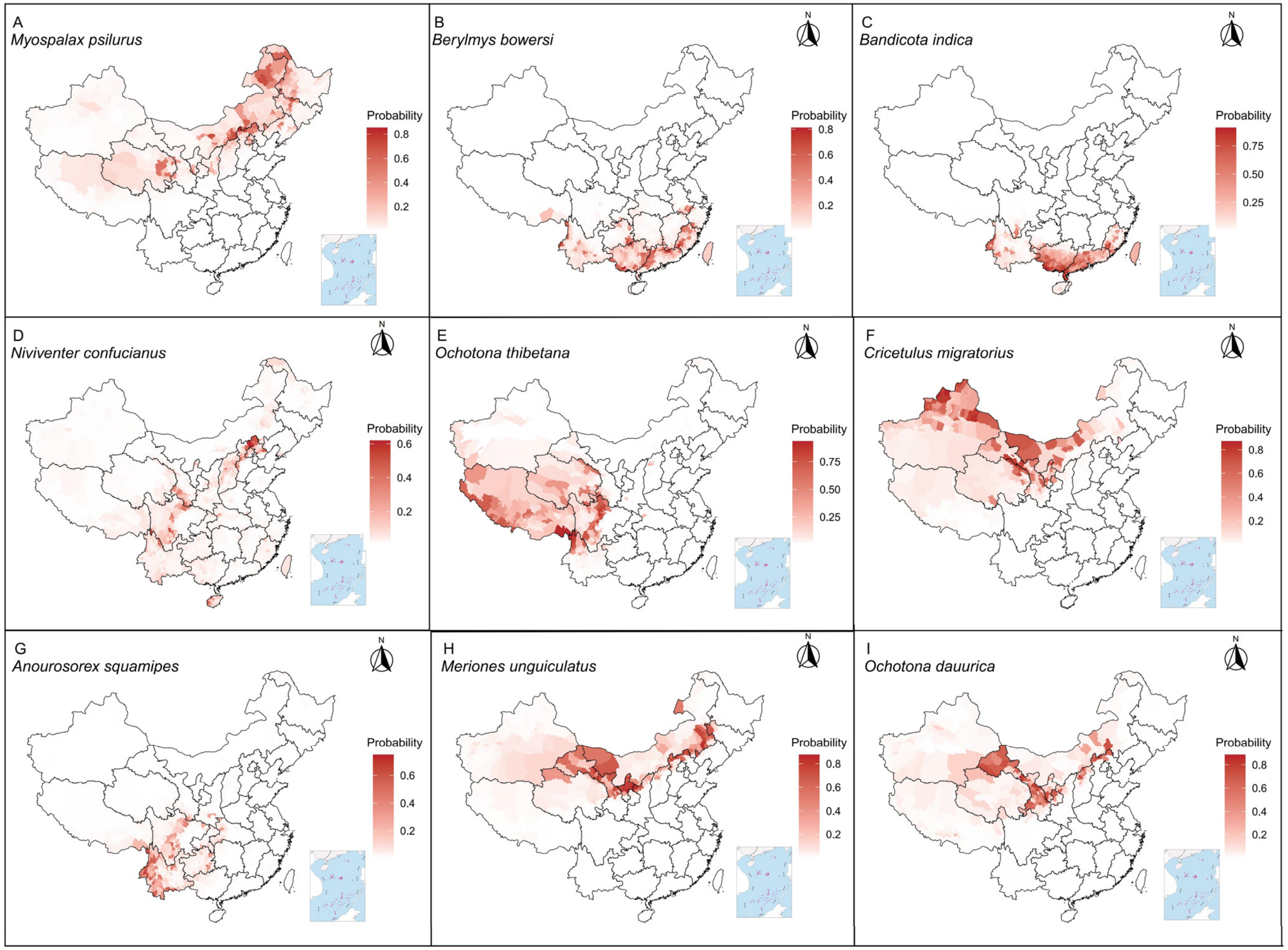

Appendix Figure S9: The mean curves (red) and 95% percentiles (gray) across 100 bootstrap samples for the effects of top six major predictors on the county-specific probability of presence for 16 small mammal species based on the BRT models. (A) *Mus musculus*; (B) *Rattus norvegicus*; (C) *Apodemus agrarius*; (D) *Rattus tanezumi*; (E) *Niviventer niviventer*; (F) *Cricetulus barabensis*; (G) *Rattus nitidus*; (H) *Sciurotamias davidianus*; (I) *Tamias sibiricus*; (J) *Tscherskia triton*; (K) *Spermophilus dauricus*; (L) *Apodemus peninsulae*; (M) *Callosciurus erythraeus*; (N) *Eospalax fontanierii*; (O) *Niviventer fulvescens*; (P) *Leopoldamys edwardsi*.

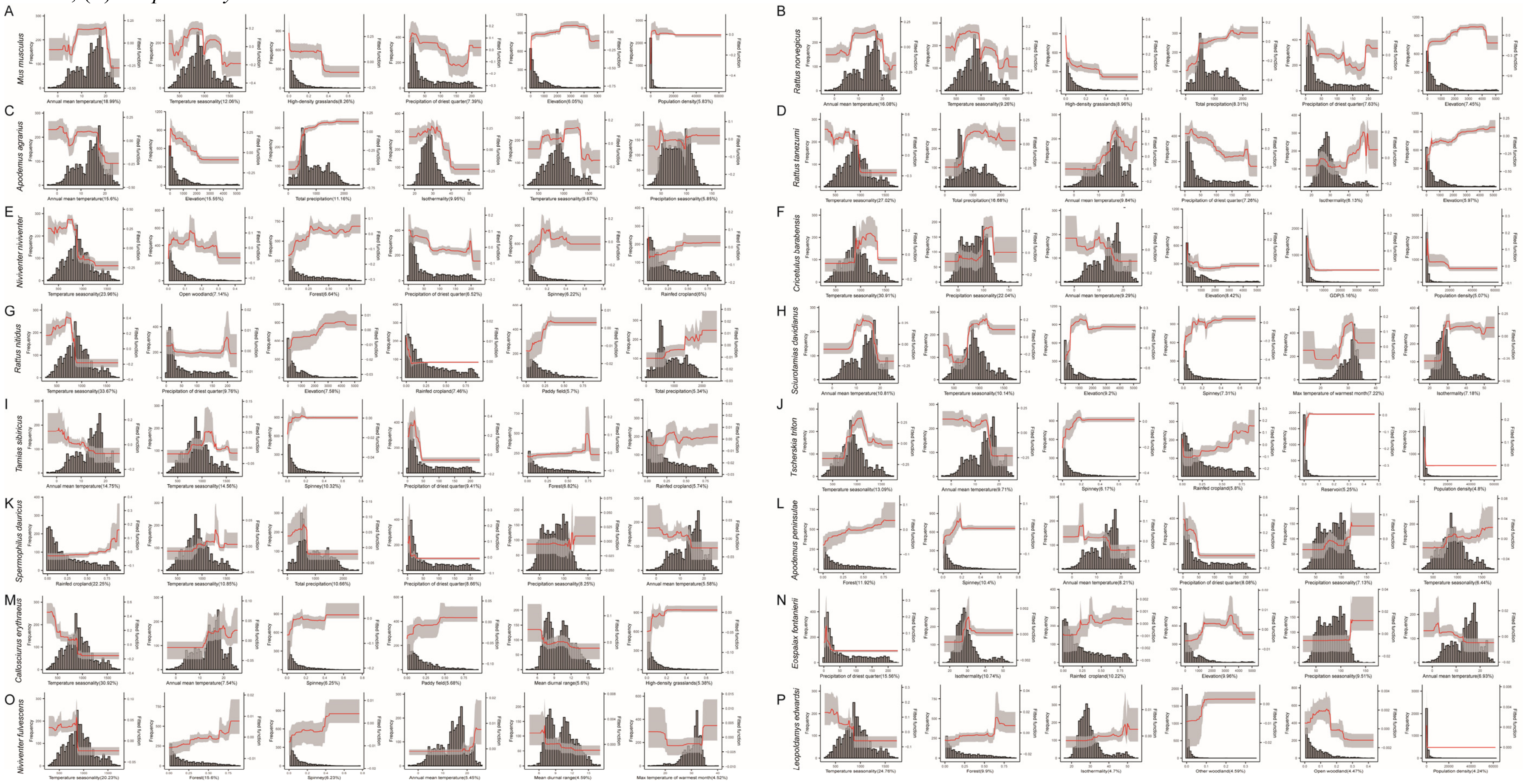

Appendix Figure S10: The mean curves (red) and 95% percentiles (gray) across 100 bootstrap samples for the effects of top six major predictors on the county-level probability of presence for 16 small mammal species based on the BRT models. (A) *Tamiops swinhoei*; (B) *Trogopterus xanthipes*; (C) *Micromys minutus*; (D) *Dremomys pernyi*; (E) *Rhizomys sinensis*; (F) *Crocidura suaveolens*; (G) *Rattus rattus*; (H) *Cricetulus longicaudatus*; (I) *Apodemus chevrieri*; (J) *Meriones meridianus*; (K) *Marmota himalayana*; (L) *Alexandromys fortis*; (M) *Rattus losea*; (N) *Orientallactaga sibirica*; (O) *Eothenomys melanogaster*; (P) *Apodemus draco*.

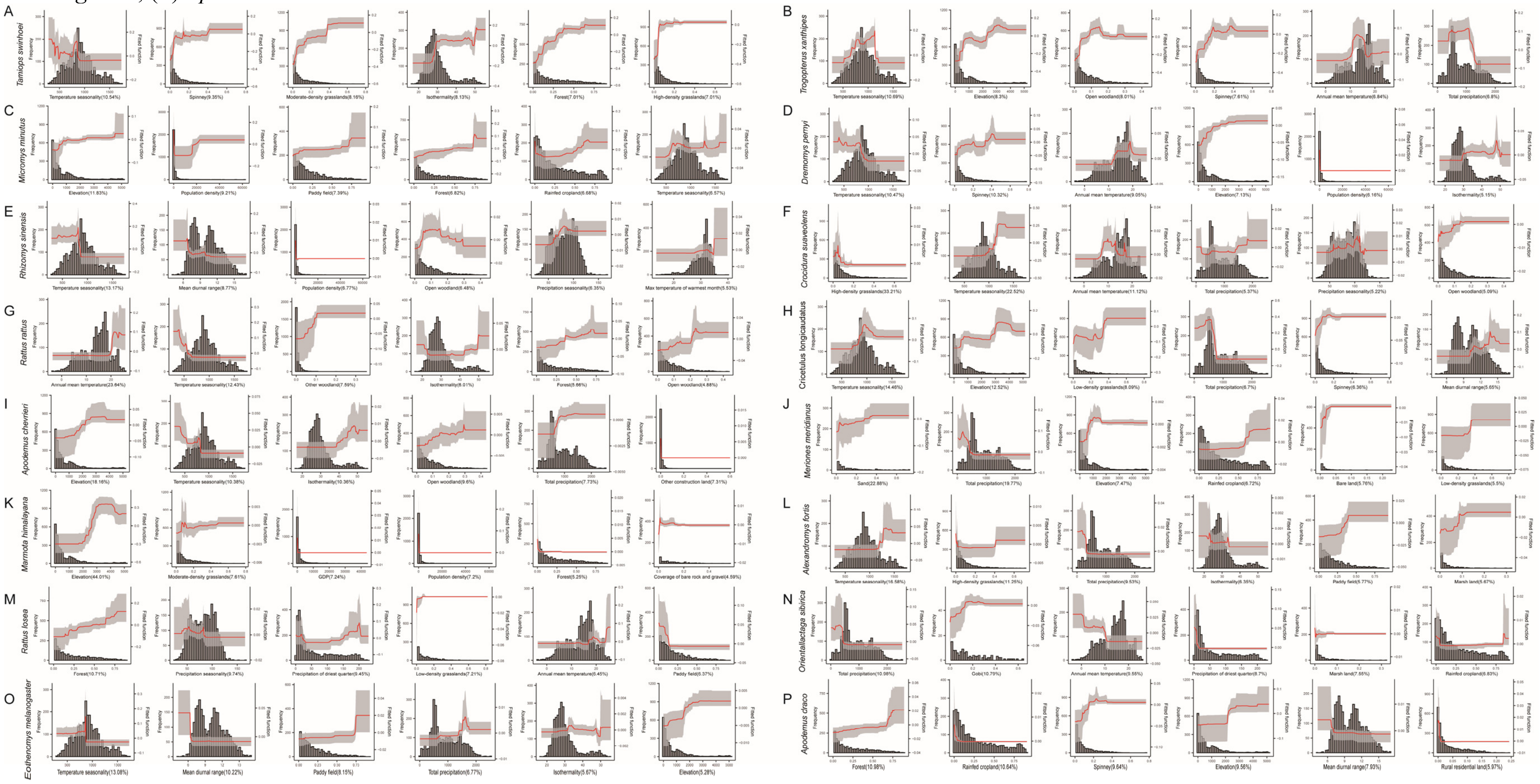

Appendix Figure S11: The mean curves (red) and 95% percentiles (gray) across 100 bootstrap samples for the effects of top six major predictors on the county-level probability of presence for 13 small mammal species based on the BRT models. (A) *Pteromys volans*; (B) *Craseomys rufocanus*; (C) *Petaurista alborufus*; (D) *Eothenomys miletus*; (E) *Myospalax psilurus*; (F) *Berylmys bowersi*; (G) *Bandicota indica*; (H) *Niviventer confucianus*; (I) *Ochotona thibetana*; (J) *Cricetulus migratorius*; (K) *Anourosorex squamipes*; (L) *Meriones unguiculatus*; (M) *Ochotona dauurica*.

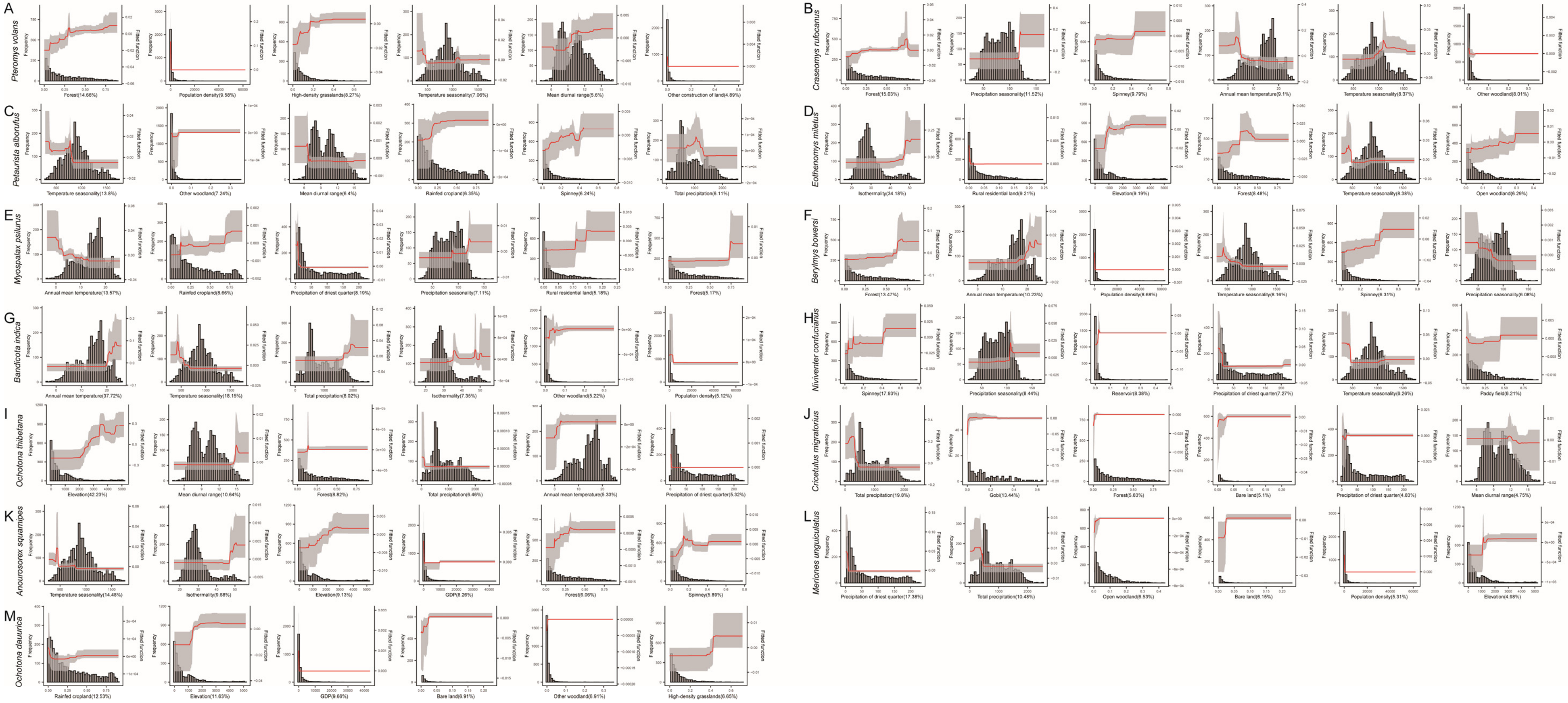

Supplement: Supplementatry Appendix 2 [file mmc7.pdf]
