# Supplementary material for: Small mammals and associated infections in China: a systematic review and spatial modelling analysis
Source: Lancet Reg Health West Pac. 2024 Dec 18;54:101264. doi: 10.1016/j.lanwpc.2024.101264 (PMC11728903; doi:10.1016/j.lanwpc.2024.101264)
Supplement: Supplementatry Appendix 3 [file mmc8.pdf]

Supplementary Appendix 3

Supplement to: Small mammals and associated infections in China: a systematic review and spatial modelling analysis

Table of Contents 3

Appendix Figure S12: The small mammal species and linked small mammal-associated viruses determined by next generation sequencing. 2
Appendix Figure S13: The small mammal species and linked small mammal-associated RNA viruses by next generation sequencing 3
Appendix Figure S14: The small mammal species and linked small mammal-associated DNA viruses by next generation sequencing 4
Appendix Figure S15: The locations of viral microbes detected in small mammal species at county level during 1950–2021 in China 5
Appendix Figure S16: The locations of bacterial microbes detected in small mammal species at county level during 1950–2021 in China 6
Appendix Figure S17: The locations of bacterial and parasitic microbes detected in small mammal species at county level during 1950–2021 in China. 7
Appendix Figure S18: The mean curves (red) and 95% percentiles (gray) across 100 bootstrap samples for the effects of major predictors (RC≥5%) on the predicted county-level presence probability (A) and incidence rate (B) of leptospirosis based on the two-step GBRT models 8
Appendix Figure S19: The mean curves (red) and 95% percentiles (gray) across 100 bootstrap samples for the effects of major predictors (RC≥5%) on the predicted county-level presence probability (A) and incidence rate (B) of Hemorrhagic fever with renal syndrome (HFRS) based on the two-step GBRT models. 9
Appendix Figure S20: Sensitivity analysis on the reported and model-predicted distributions of Hemorrhagic fever with renal syndrome (HFRS) at the county level in China, where serological detection of hantavirus in small mammals is considered evidence of presence 10
Appendix Figure S21: Sensitivity analysis: the mean curves (red) and 95% percentiles (gray) across 100 bootstrap samples for the effects of major predictors (RC≥5%) on the predicted county-level presence probability (A) and incidence rate (B) of Hemorrhagic fever with renal syndrome (HFRS) based on the two-step GBRT models, where serological detection of hantavirus in small mammals is considered evidence of presence in the step-1 GBRT model. 11
Appendix Figure S22: Model-predicted (A and B) and observed geographic distribution of *Mus musculus* based on data from 1950-2021 (A and C) and 1950-2000 (B and D) 12
Appendix Figure S23: Model-predicted (A and B) and observed geographic distribution of *Rattus norvegicus* based on data from 1950-2021 (A and C) and 1950-2000 (B and D). 13
Appendix Figure S24: Observed (A and B) and model-predicted (C and D) geographic distributions and variable importance (E and F) for *Mus musculus* based on “case” counties defined by ≥1 recorded occurrences (A, C, E) and ≥2 recorded occurrences (B, D, F). 14
Appendix Figure S25: Observed (A and B) and model-predicted (C and D) geographic distributions and variable importance (E and F) for *Cricetulus barabensis* based on “case” counties defined by ≥1 recorded occurrences (A, C, E) and ≥2 recorded occurrences (B, D, F). 15

Appendix Figure S12: The small mammal species and linked small mammal-associated viruses determined by next generation sequencing.

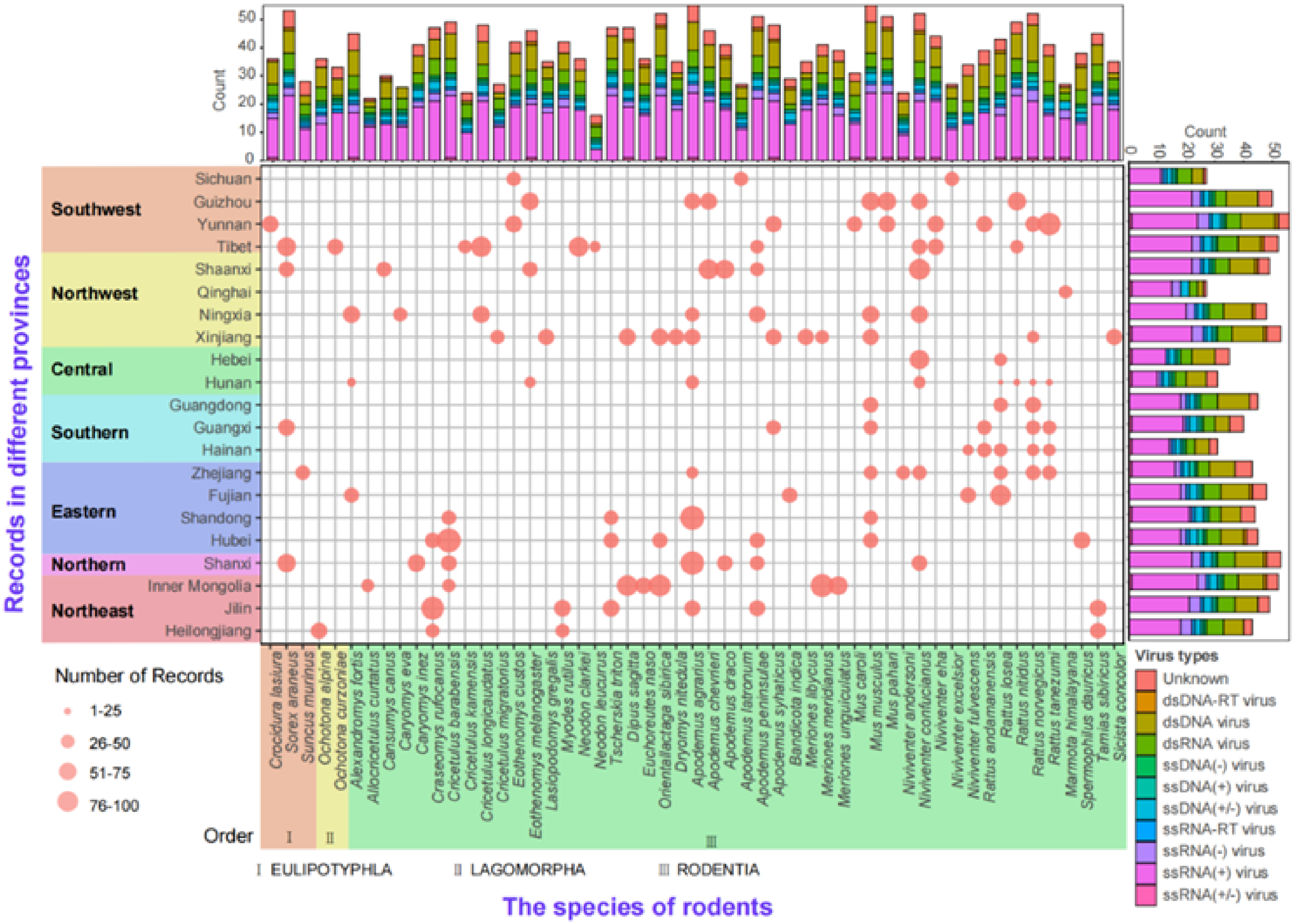

Appendix Figure S13: The small mammal species and linked small mammal-associated RNA viruses by next generation sequencing

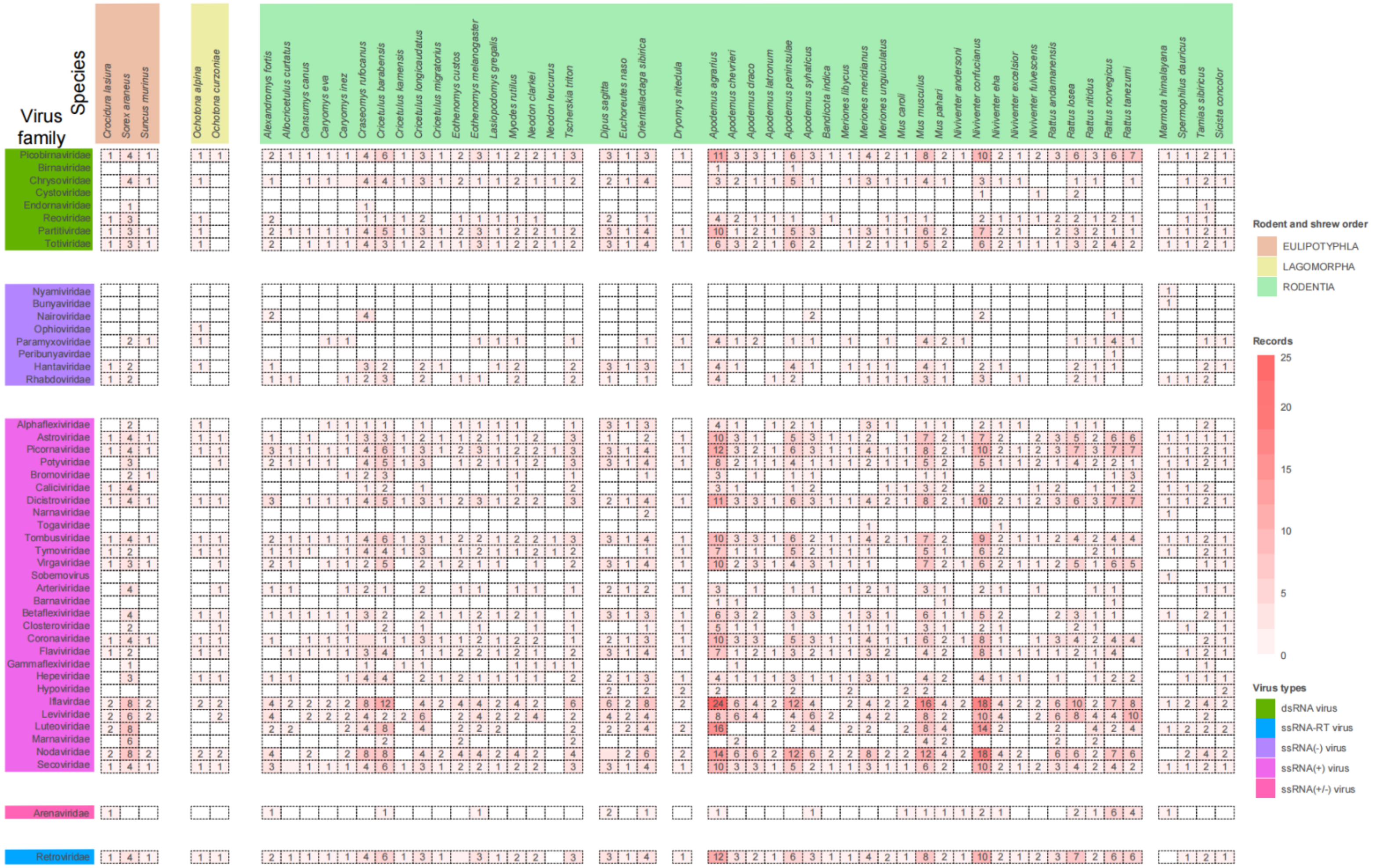

Appendix Figure S14: The small mammal species and linked small mammal-associated DNA viruses by next generation sequencing

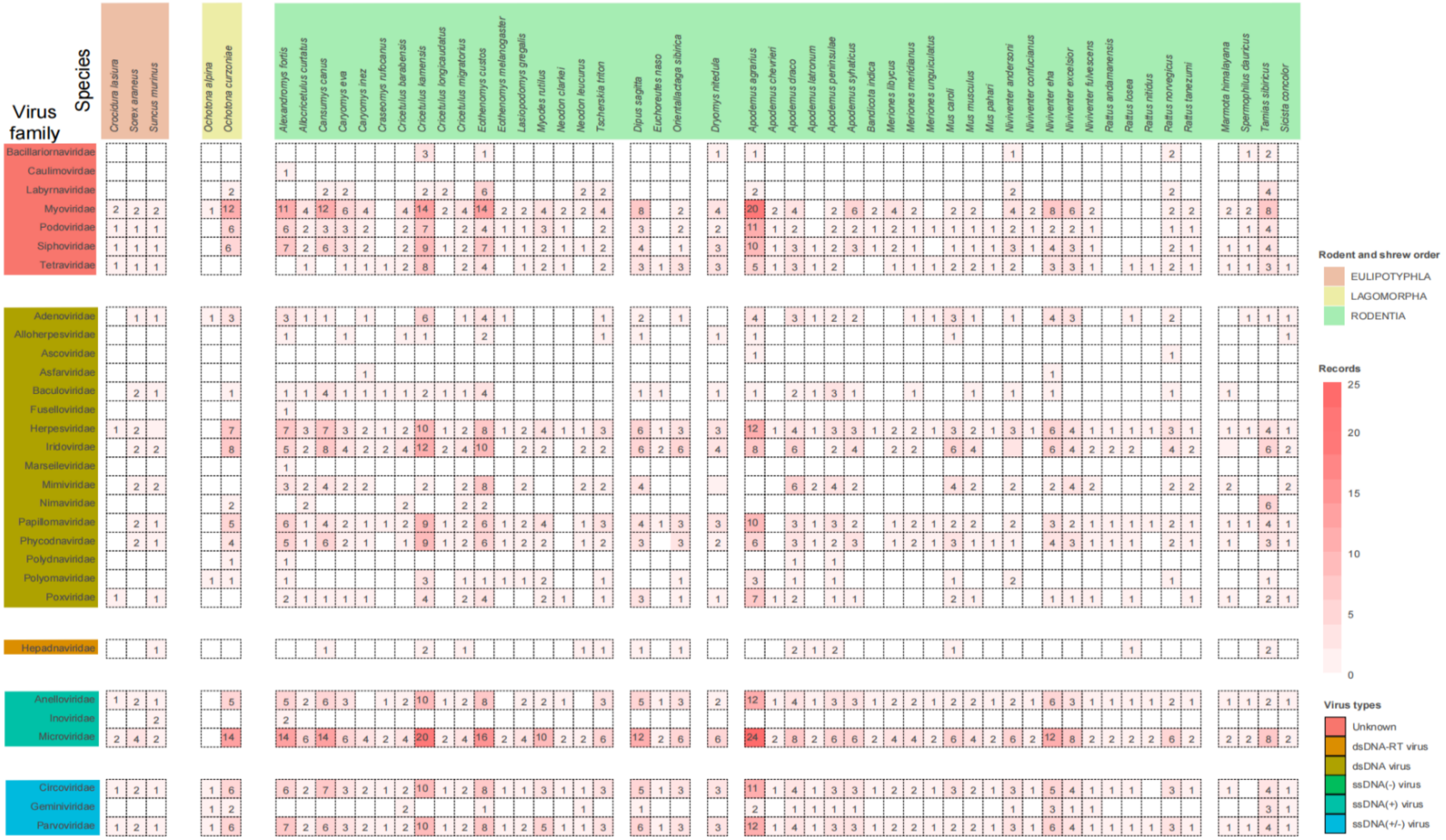

Appendix Figure S15: The locations of viral microbes detected in small mammal species at county level during 1950–2021 in China

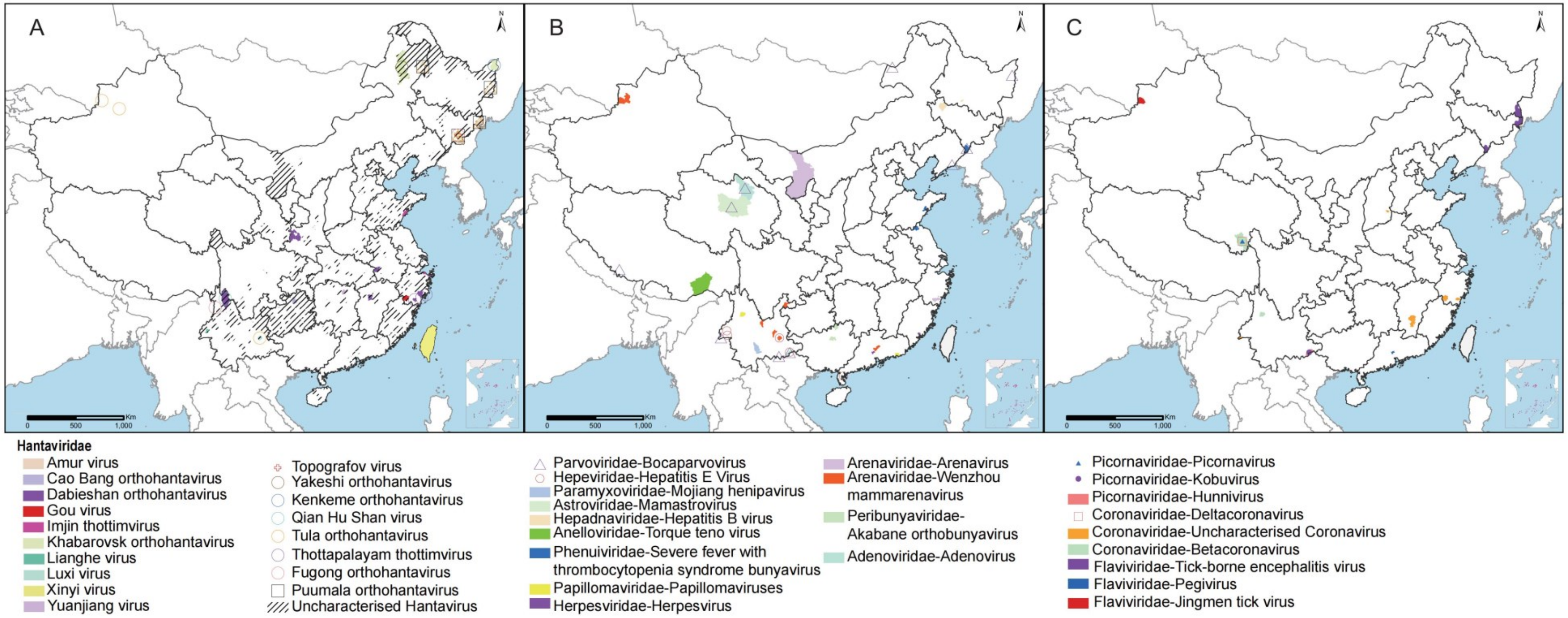

Appendix Figure S16: The locations of bacterial microbes detected in small mammal species at county level during 1950–2021 in China

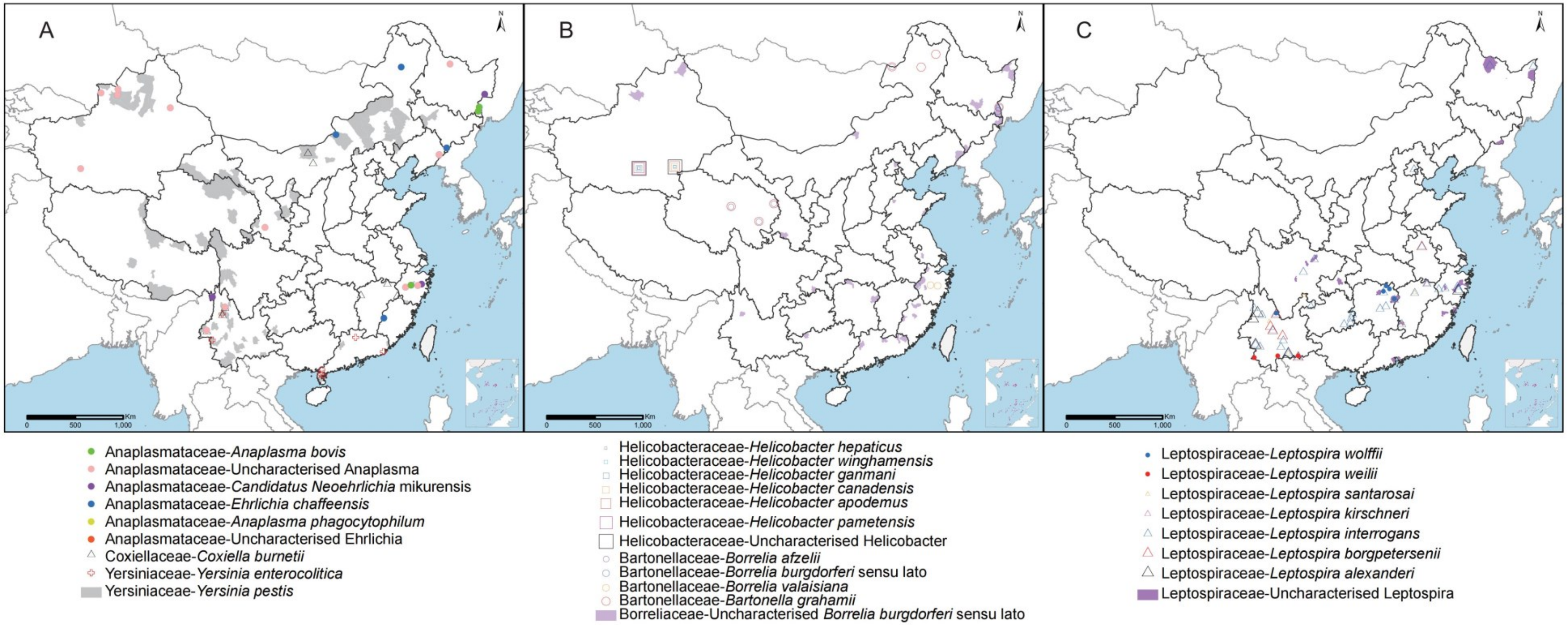

Appendix Figure S17: The locations of bacterial and parasitic microbes detected in small mammal species at county level during 1950–2021 in China.

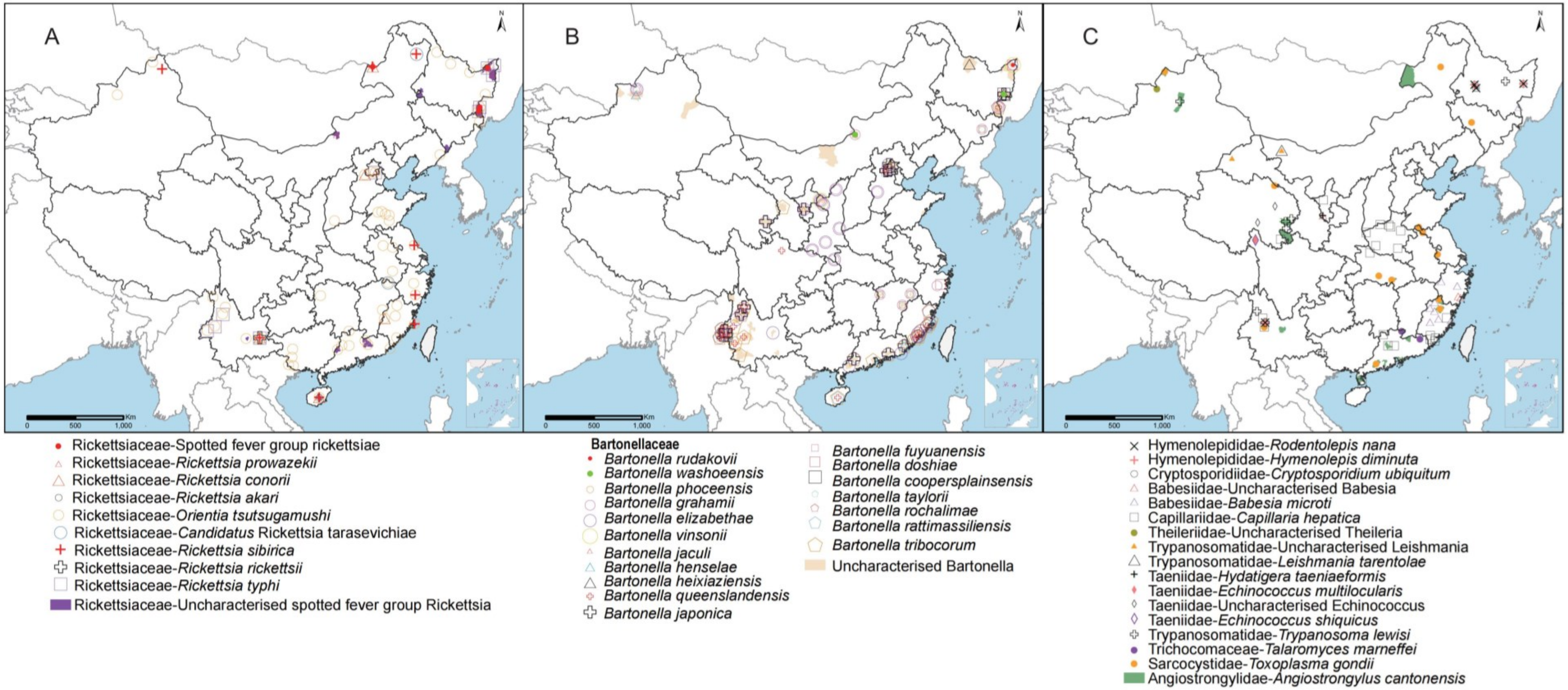

Appendix Figure S18: The mean curves (red) and 95% percentiles (gray) across 100 bootstrap samples for the effects of major predictors (RC≥5%) on the predicted county-level presence probability (A) and incidence rate (B) of leptospirosis based on the two-step GBRT models. The relative contribution of each predictor is shown in the parenthesis in the X-axis label. The presence probabilities are predicted by the step-1 logistic GBRT using all counties, and the incidence rates predicted by the step-2 gamma-distributed GBRT model using counties with predicted presence probability > 0.5.

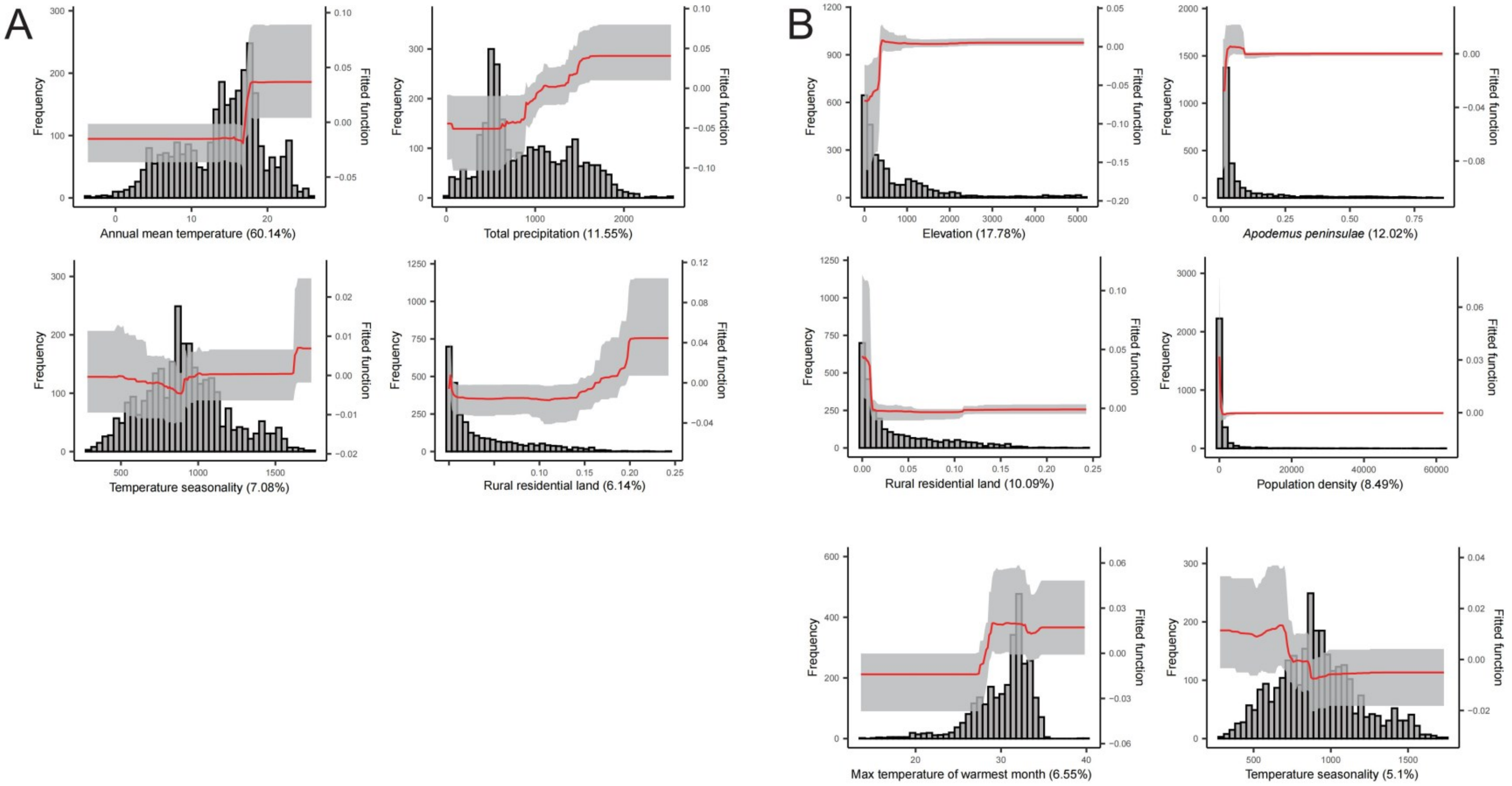

Appendix Figure S19: The mean curves (red) and 95% percentiles (gray) across 100 bootstrap samples for the effects of major predictors (RC≥5%) on the predicted county-level presence probability (A) and incidence rate (B) of Hemorrhagic fever with renal syndrome (HFRS) based on the two-step GBRT models. The relative contribution of each predictor is shown in the parenthesis in the X-axis label. The presence probabilities are predicted by the step-1 logistic GBRT using all counties, and the incidence rates predicted by the step-2 gamma-distributed GBRT model using counties with predicted presence probability > 0.5.

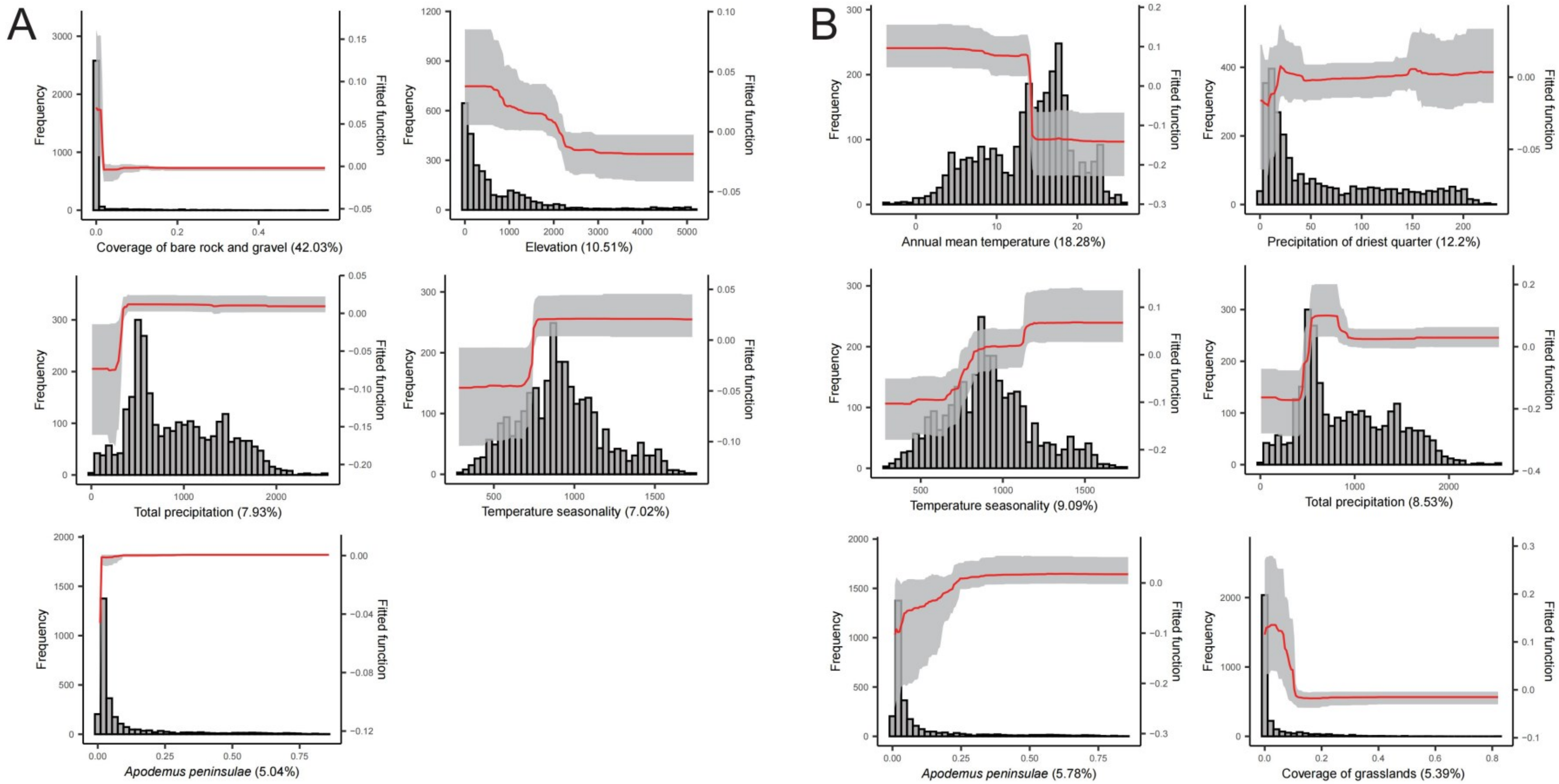

Appendix Figure S20: Sensitivity analysis on the reported and model-predicted distributions of Hemorrhagic fever with renal syndrome (HFRS) at the county level in China, where serological detection of hantavirus in small mammals is considered evidence of presence. (A) Reported annual incidence rate of Hemorrhagic fever with renal syndrome (HFRS) and locations of hantavirus detected from small mammals by molecular detection, isolation, and serological method; (D) spatial distribution of model-predicted incidence rate of HFRS.

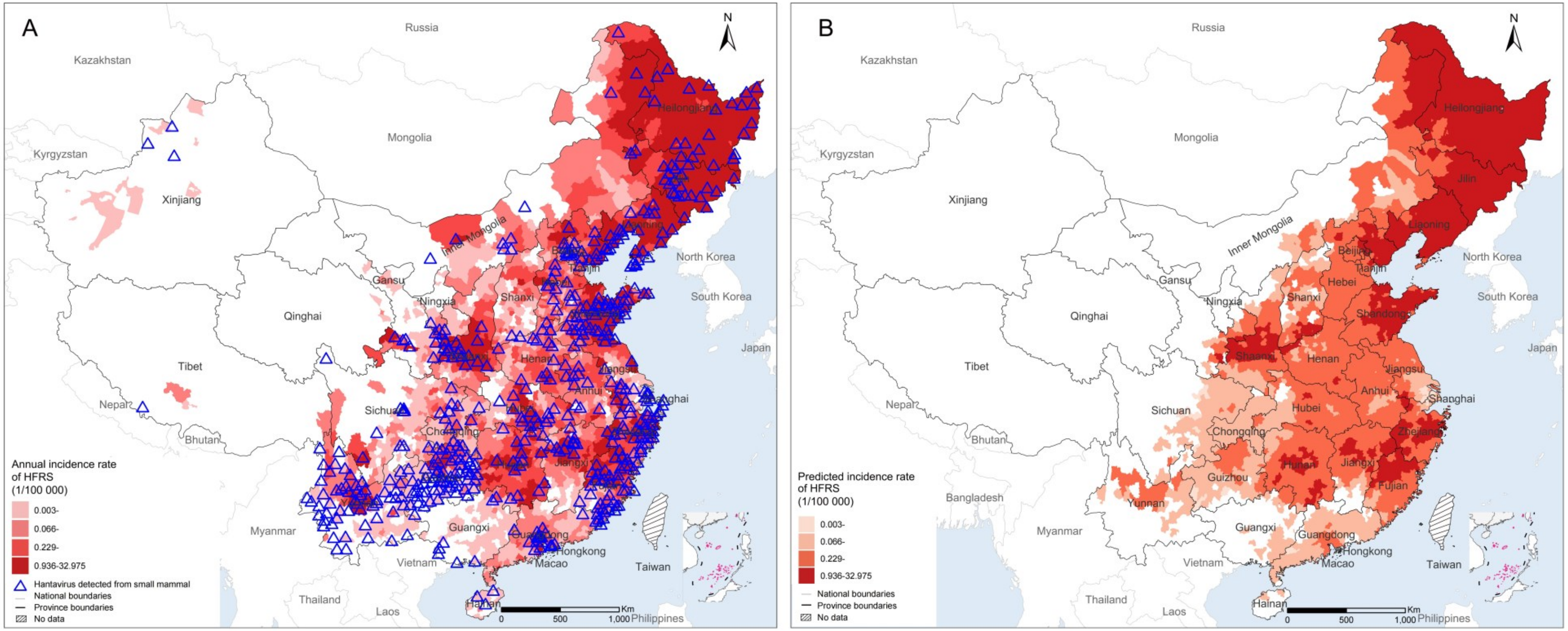

Appendix Figure S21: Sensitivity analysis: the mean curves (red) and 95% percentiles (gray) across 100 bootstrap samples for the effects of major predictors (RC≥5%) on the predicted county-level presence probability (A) and incidence rate (B) of Hemorrhagic fever with renal syndrome (HFRS) based on the two-step GBRT models, where serological detection of hantavirus in small mammals is considered evidence of presence in the step-1 GBRT model. The relative contribution of each predictor is shown in the parenthesis in the X-axis label. The presence probabilities are predicted by the step-1 logistic GBRT using all counties, and the incidence rates predicted by the step-2 gamma-distributed GBRT model using counties with predicted presence probability > 0.5.

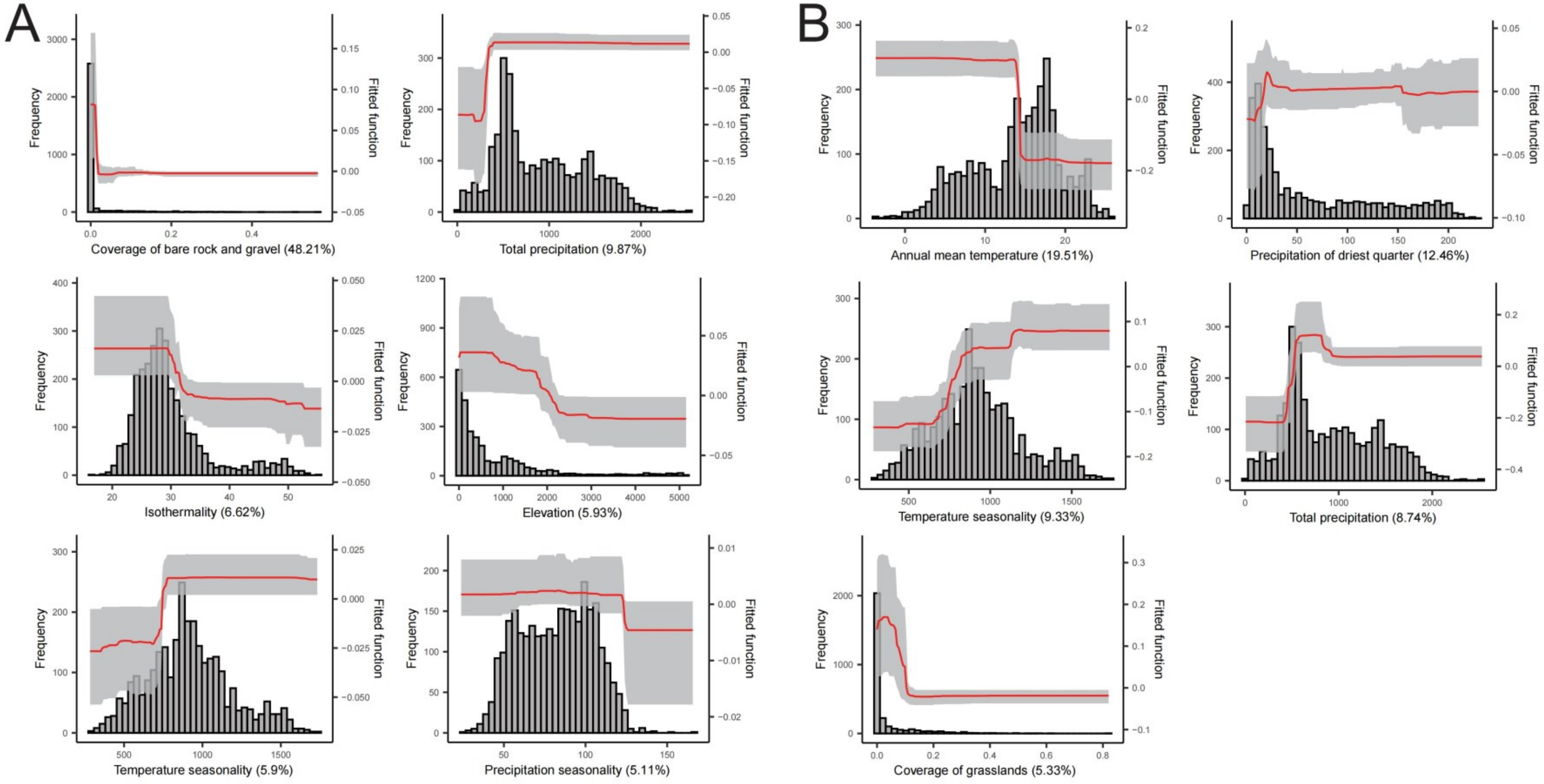

Appendix Figure S22: Model-predicted (A and B) and observed geographic distribution of *Mus musculus* based on data from 1950-2021 (A and C) and 1950-2000 (B and D).

(A) Model-predicted (1950-2021)

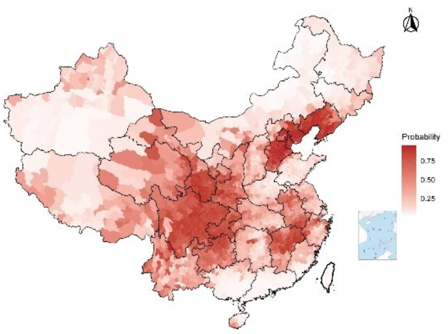

(B) Model-predicted (1950-2000)

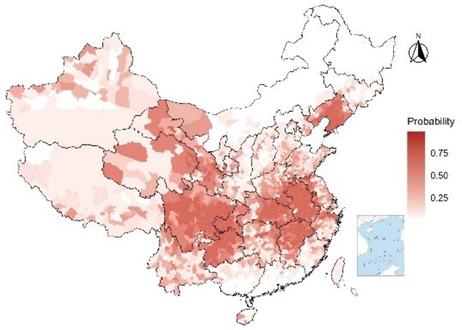

(C) Observed (1950-2021)

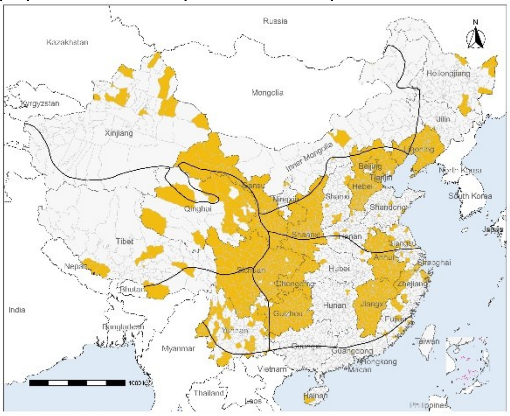

(D) Observed (1950-2000)

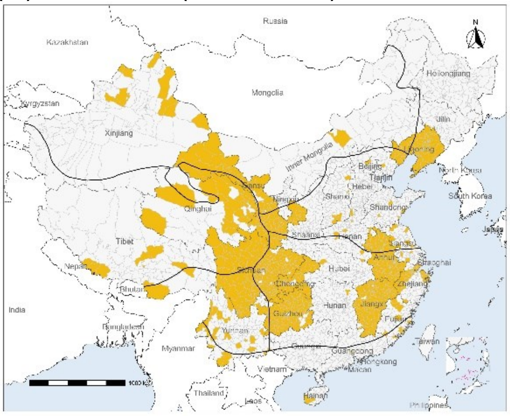

Appendix Figure S23: Model-predicted (A and B) and observed geographic distribution of *Rattus norvegicus* based on data from 1950-2021 (A and C) and 1950-2000 (B and D).

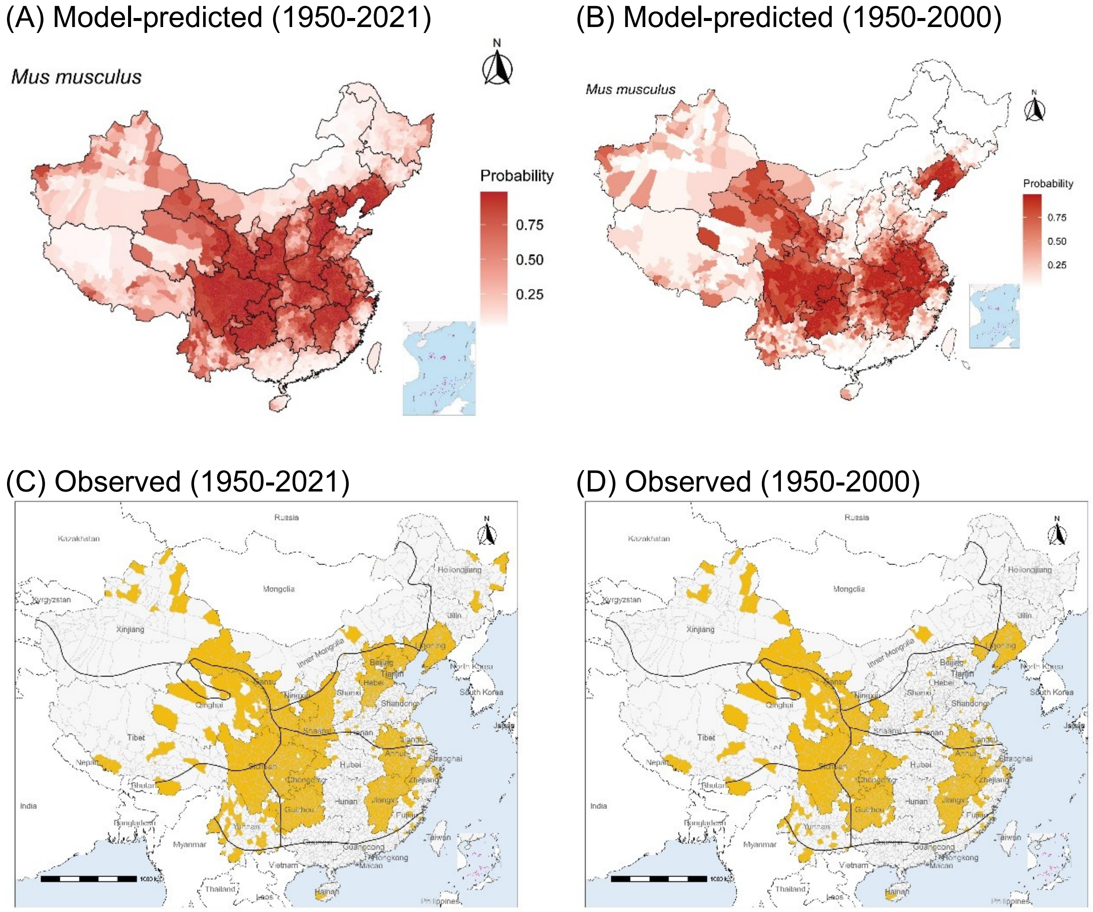

Appendix Figure S24: Observed (A and B) and model-predicted (C and D) geographic distributions and variable importance (E and F) for *Mus musculus* based on “case” counties defined by ≥1 recorded occurrences (A, C, E) and ≥2 recorded occurrences (B, D, F).

(A) Observed, ≥1 record

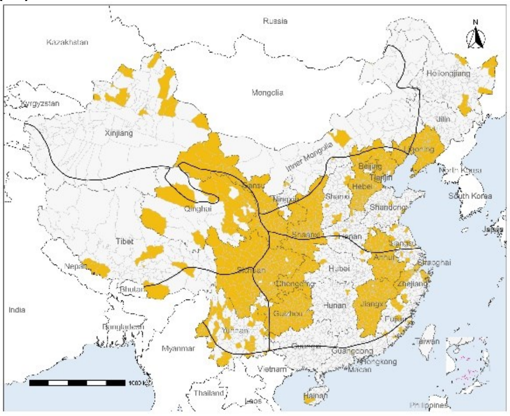

(B) Observed, ≥2 records

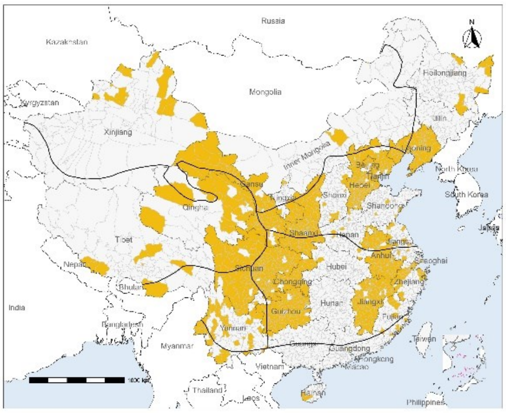

(C) Model-predicted, ≥1 record

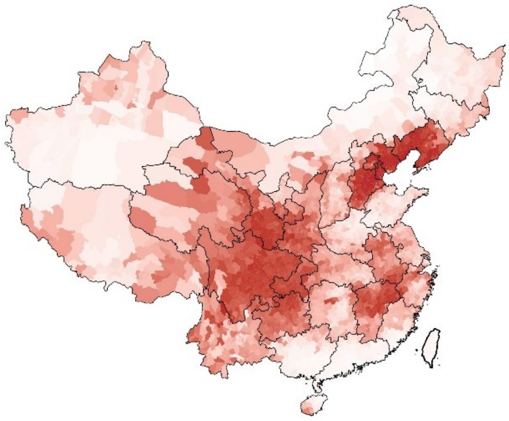

(D) Model-predicted , ≥2 records

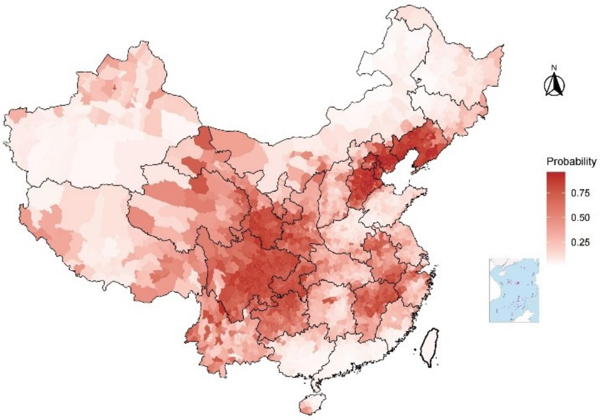

(E) Variable importance, ≥1 records

One record	*Mus musculus*	RC (%)
1	Annual Mean Temperature	18.99
2	Temperature Seasonality	12.06
3	High Coverage Grasslands	8.26
4	Precipitation of Driest Quarter	7.39
5	Elevation	6.05
6	Population Density	5.83

(F) Variable importance, ≥2 records

Two records	*Mus musculus*	RC (%)
1	Temperature Seasonality	24.28
2	Annual Mean Temperature	24.15
3	Precipitation Seasonality	15.66
4	Precipitation of Driest Quarter	11.58
5	Total precipitation	11.72
6	Isothermality	7.54

Appendix Figure S25: Observed (A and B) and model-predicted (C and D) geographic distributions and variable importance (E and F) for *Cricetulus barabensis* based on "case" counties defined by ≥1 recorded occurrences (A, C, E) and ≥2 recorded occurrences (B, D, F).

(A) Observed, ≥1 records

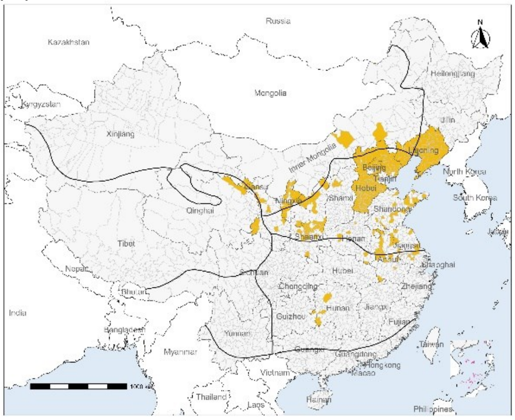

(B) Observed, ≥2 records

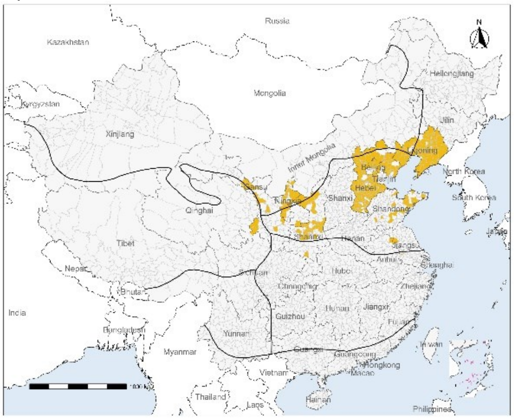

(C) Model-predicted , ≥1 records

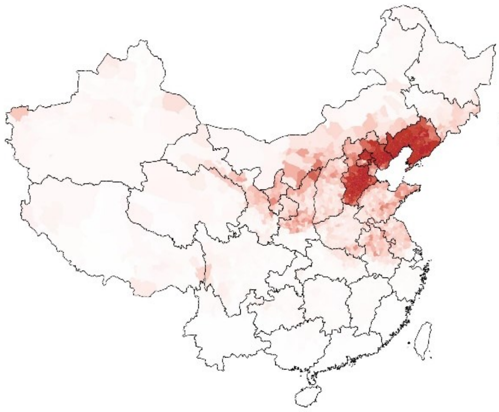

(D) Model-predicted , ≥2 records

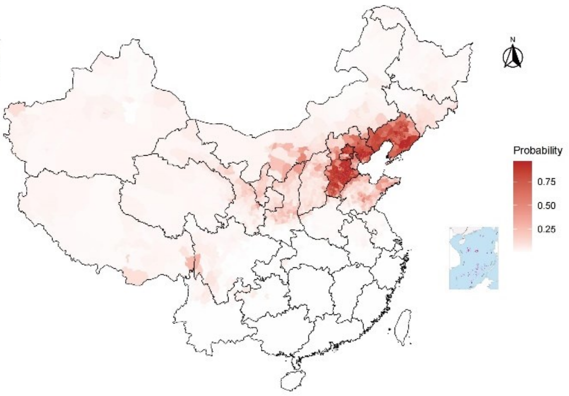

(E) Variable importance, ≥1 records

One records	*Cricetulus barabensis*	RC (%)
1	Temperature seasonality	30.91
2	Precipitation seasonality	22.04
3	Annual Mean Temperature	9.29
4	Elevation	8.42
5	GDP	5.16
6	Population Density	5.07

(F) Variable importance, ≥2 records

Two records	*Cricetulus barabensis*	RC (%)
1	Precipitation Seasonality	36.21
2	Annual Mean Temperature	21.59
3	Temperature Seasonality	14.50
4	Total precipitation	11.96
5	Isothermality	6.04
6	Population Density	5.42
